# ﻿*Primulameishanensis* (Primulaceae), a new species from Sichuan, China

**DOI:** 10.3897/phytokeys.248.127117

**Published:** 2024-10-23

**Authors:** Tingyu Li, Xinyu Chen, Bo Li, Donglai Hua, Can Luo, Huixian Luo, Yun Liang, Jieli Yue, Xiaodan Xi, Ke Huang, Zhixi Fu

**Affiliations:** 1 Ministry of Education, Key Laboratory of Land Resources Evaluation and Monitoring in Southwest (Sichuan Normal University), Chengdu 610101, China; 2 College of Life Sciences, Sichuan Normal University, Chengdu 610101, China; 3 Sichuan Environmental Monitoring Center, Chengdu 610091, China; 4 College of Life Sciences, Mianyang Teachers’ College, Mianyang 621000, China; 5 Meishan Eco-environmental Monitoring Center Station of Sichuan Province, Meishan, 620010, China; 6 Sustainable Development Research Center of Resources and Environment of Western Sichuan, Sichuan Normal University, Chengdu 610101, China

**Keywords:** Morphological characters, new species, *Primula* sect. *Petiolares*, taxonomy

## Abstract

*Primulameishanensis* K.Huang & Z.X.Fu, **sp. nov**., a new species of Primulaceae from Meishan City, Sichuan Province, China, is described and illustrated. The morphological data and phylogenetic analysis, based on the complete chloroplast genome, suggest that *Primulameishanensis* is a separate species closely related to *Primuladejuniana*. The complete chloroplast genome of *Primulameishanensis* was 152,175 bp and the complete chloroplast genome of *Primuladejuniana* was 151,988 bp. The new species differs from the latter by the solitary scape, the length of petiole, acute leaf blade apex and pin flower. The distribution map, morphological comparison of related species and conservation status of the new species are also provided.

## ﻿Introduction

The genus *Primula* L. is one of the most diverse member within the Primulaceae. It consists of 38 sections and more than 500 species worldwide (Hu and Kelso 1996; Richards 2003). It is widely distributed throughout temperate and cold regions of Asia and Europe, alongside the tropical mountains of the Northern Hemisphere. The genus is composed of herbaceous plants with a basal rosette of leaves, flowers on top of a naked scape, gathered in lateral or perpendicular to the axis umbels. China hosts over 340 species, with particular biodiversity hotspots of *Primula* found in south-western China (Hu 1990; Hu 1998; Richards 2003; Ju et al. 2023; Li et al. 2023). In recent years, several new species of *Primula* from China were reported, for example, *Primulalihengiana* C. M. Hu & R. Li (Li and Hu 2009), *Primulawawushanica* G. Hao, C.M. Hu & Y. Xu, *Primulaundulifolia* G. Hao, C. M. Hu & Y. Xu, *Primulapengzhouensis* C.M. Hu, G. Hao & Y. Xu., *Primulasurculos*a Y. Xu & G. Hao (Xu et al. 2016a, b, 2017, 2022), *Primuladongchuanensis* Z.K. Wu & Yuan Huang (Wu et al. 2019), *Primuladujiangyanensis* W. B. Ju, Bo Xu & X. F. Gao (Ju et al. 2021), *Primulalongipilosa* Ze H. Wang & H. Peng (Wang et al. 2022), *Primulawolongensis* W.B.Ju, Bo Xu & X.F.Gao (Li et al. 2023), *Primulasugongii* J.D.Ya, Bin Yang & Y.H. Tan (Yang et al. 2023) and *Primulaxilingensis* K. Huang & Z.X. Fu (Luo et al. 2023).

The Primulasect.Petiolares Pax comprises more than 60 currently recognised species (Hu 1994; Hu and Kelso 1996; Hu and Geng 2003; Rankin 2010; Wei et al. 2022; Zhang et al. 2023) and are well represented in the region of Himalaya-Hengduan Mountains, with only a few members extending into Kashmir, central China and some other regions (Hu and Kelso 1996). One of the most important diagnostic characters of this section is a globose capsule which does not open by valves, but by crumbling at the membrane apex (Hu 1994).

During the botanical expedition of Zhongyan temple in Qingshen County, Meishan City, Sichuan Province from 2022 to 2024, a population of *Primula* was discovered, photographed and collected. Based on the photographs of the flowering taxon, it appears to be closely related to *Primuladejuniana* G. Hao, C.M. Hu & Y. Xu (Xu et al. 2014) at first sight. After consultation with relevant literature and conducting morphological examination of closely-related taxa, it was determined that it represents an unreported taxon from P.sect.Petiolares. The new species could be differentiated from other members of the section by the following combination: scape solitary, the length of petiole 2.5–5 cm, a terminal umbel of (1) 2–3 (4) flowers, leaf blade smooth, oblanceolate, 19.5–25 × 2.5–5 cm, apex acute, corolla pastel violet, 5–6 lobes spreading and stamens inserted at middle of corolla tube, corolla tubes 11.0–12.0 mm long, ca. 2 mm in diam., style 4.0–7.0 mm above base of corolla tube, stamens reaching the corolla tube mouth, 7.0–12.0 mm above base of corolla tube.

In addition, in this study, the molecular data of complete chloroplast genomic data were collected and used to identify its relationship with *Primuladejuniana*. In recent years, many new species have been jointly supported by genetic and morphological data, including *Primulasunhangii* T. Deng, D. G. Zhang & Jiao Sun (Sun et al. 2020), *Asterquanzhouensis* M.Tang, G.J.Yan & W.P.Li (Xiao et al. 2022), *Primulaundulifolia* G. Hao, C. M. Hu & Y. Xu (Xu et al. 2016), *Asteryaoshanensis* K. Qin, Z.X. Fu and P. Li. (Zheng et al. 2024) and *Saussureatalungensis* S.K.Ghimire & H.K.Rana (Rana et al. 2021).

In this study, we provide a detailed description of this new species, based on observations of living plants in the field and specimens in the herbarium.

## ﻿Material and methods

### ﻿Morphological analysis

The observation and collection of both herbarium and living materials of the new species from Qingshen County, Meishan City, Sichuan Province, were conducted in December 2022, January 2023, January 2024 and March 2024. We conducted a morphological comparison using taxonomic literature of closely-related species, i.e. *Primuladejuniana* (description of reference Xu et al. (2014)) and *Primuladavidii* Franch. (holotype: *David, A.1870* (P, image!), as well as the images of specimens from the Global Plants JSTOR database (https://plants.jstor.org), i.e. *Primulaepilosa* Craib (holotype: *Sun231* (KUN, image!) were consulted. Morphological description and measurements of *P.meishanensis* were based on living plants. The taxonomic description followed the terminology used by Beentje (2016). The holotype voucher specimens were stored at the Herbarium of Sichuan Normal University (SCNU). The conservation status of the new species was assessed following the guidelines of the IUCN Red List Categories and Criteria (IUCN 2024).

### ﻿DNA extraction and sequencing

Total genomic DNA was obtained using the CTAB method (Doyle and Doyle 1987). We follow the Illumina DNA Library Construction Guide to complete paired-end DNA library construction (Allen et al. 2006). The complete chloroplast genome was sequenced on the Illumina HiSeq XTen platform (San Diego, CA, USA). In this study, SPAdes v.3.10.1 software was used to assemble high-quality data by default parameters (Bankevich et al. 2012). We further used CPGView (http://47.96.249.172:16085/cpgview/view) to improve annotation, visualise the structure of the cp genome and identify gene structures including cis-splicing and trans-splicing (Liu et al. 2023). The genome sequence of *Primuladejuniana and P.meishanensis* have been deposited in GenBank (accession numbers: PQ213817 and PQ213816).

### ﻿Phylogenetic analysis

The phylogenetic analysis of the complete chloroplast genomic dataset of 18 species was performed using the Maximum Likelihood (ML) method implemented in RAxML. The species *Maesamontana* A. DC. (KU569490) and *Ardisiapolysticta* Migo (KC465962) were selected as outgroups. These chloroplast genome sequences were imported into MAFFT v.7.520 software (Katoh and Standley 2013) for multiple comparisons and the phylogenetic tree was constructed using CIPRES (https://www.phylo.org/), with the ML method, based on the GTRGAMMA model and the bootstrap was set to 1, 000 (Stamatakis et al. 2008) (Table 2) (Fig. 5).

## ﻿Result and discussion

### ﻿Taxonomic treatment

#### ﻿ Primula meishanensis

Taxon classificationPlantaeEricalesPrimulaceae

K.Huang & Z.X.Fu sp. nov.

E91F8418-0885-5E85-89A8-544E22E3190A

urn:lsid:ipni.org:names:77350705-1

##### Type.

**Figure 1.**
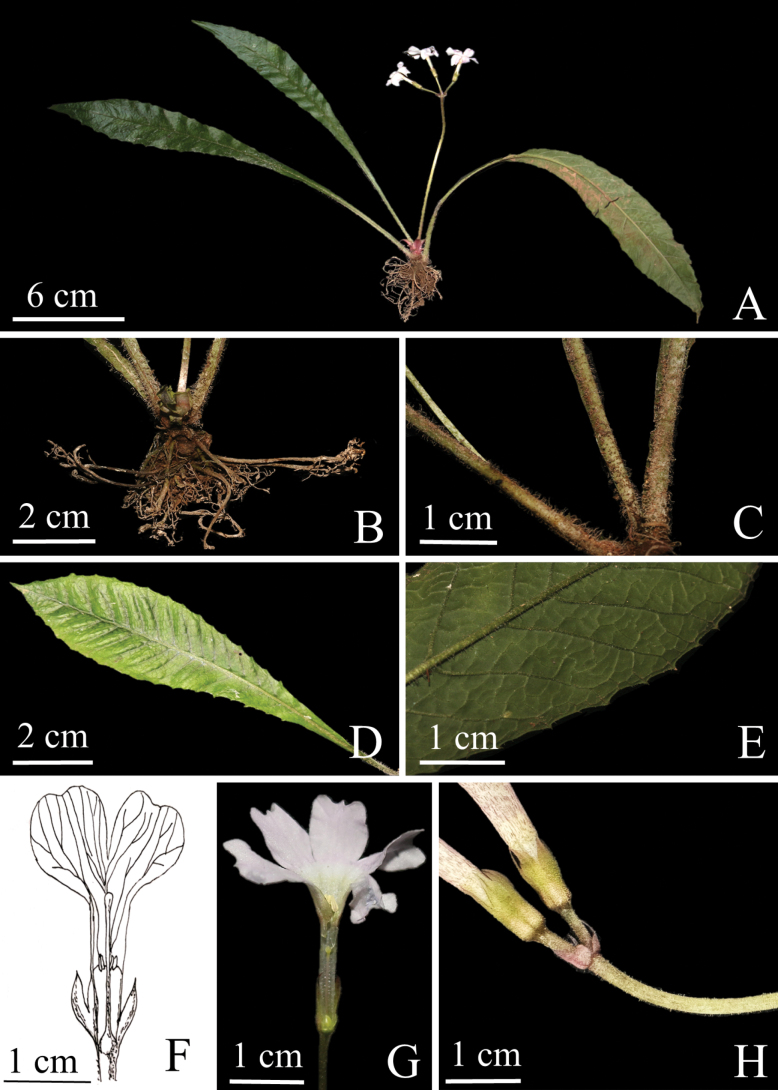
*Primulameishanensis* sp. nov. **A** plant and roots **B** roots **C** petioles **D, E** leaves **F** pin flower **G** thrum flower **H** scape and bract (Photos by XC).

**Figure 2.**
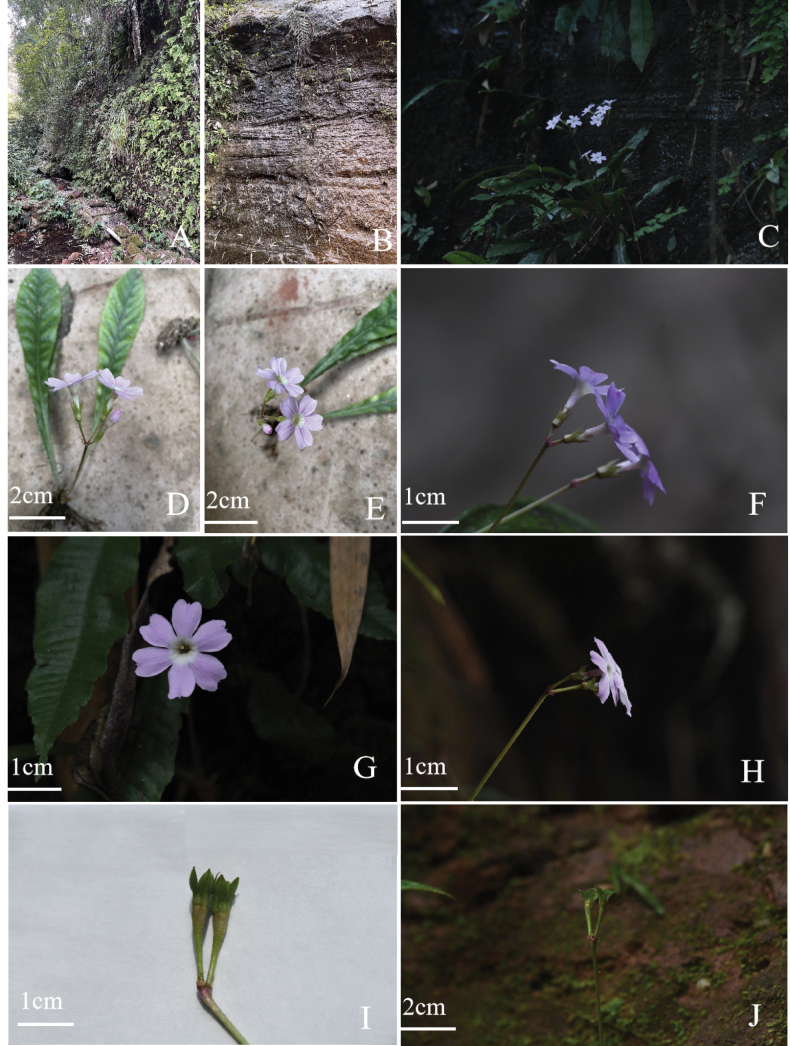
*Primulameishanensis* sp. nov. **A–C** habitat **D–H** flowers **I, J** fruit (Photos **A**, **B**, **D, E** by ZF and **C, F–J** by KH).

**Figure 3.**
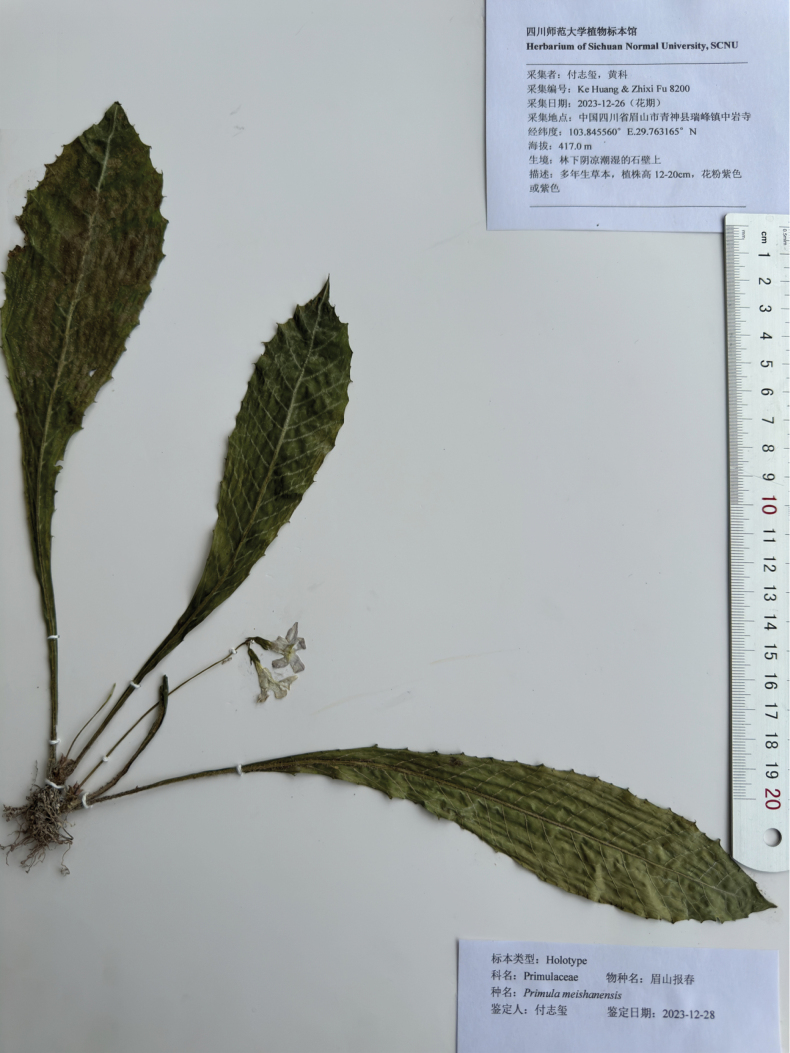
Holotype image of *Primulameishanensis* K.Huang & Z.X.Fu, sp. nov.

China, Sichuan Province, Meishan City, Qingshen County, Zhongyan temple, grows on moist rock surfaces amidst moss under the forest, at elevations of approximately 417 m; 29°45′47.39″N, 103°50′44.017″E; 26 December 2023 (fl.), *Ke Huang & Zhixi Fu 8200* (holotype SCNU!) (Figs 1–3).

##### Diagnosis.

The new species is morphologically similar to *Primuladejuniana* in having ciliate, sharply and remotely dentate leaf blade margin, while it can be easily recognised by the following combination of characters: scape solitary, the length of petiole 2.5–5 cm, a terminal umbel of (1) 2–3 (4) flowers, leaf blade smooth, oblanceolate, 19.5–25 × 2.5–5 cm, apex acute, corolla pastel violet, 5–6 lobes spreading and stamens inserted at middle of corolla tube, corolla tubes 11.0–12.0 mm long, ca. 2 mm in diam., style 4.0–7.0 mm above base of corolla tube, stamens reaching the corolla tube mouth, 7.0–12.0 mm above base of corolla tube. (Figs 1–3).

##### Description.

Perennial herbs, 12.0–20.0 cm tall. ***Roots*** numerous, fibrous, without hairs. ***Leaves*** pilose, forming a spreading rosette, each rosette with only 2−4 leaves of previous year at flowering time; resting bud of rosette clothed by a few small paleaceous scales, basal bud scales ovate to ovate-oblong, ciliolate, rose red, apex acute; petiole 2.5–5 cm long, narrowly winged and densely covered with multicellular hairs; leaf blade oblanceolate, smooth, 19.5–25 × 2.5–5 cm, cuneate at base, abaxial surface densely along mid-vein, sparser on lateral veins covered with multicellular hairs; margin ciliate, sharply and remotely dentate; apex acute; mid-vein dull yellow in fresh state, turning brownish when dry; veins impressed adaxially, prominently raised and subalveolate abaxially. ***Scape*** solitary, 7.2–11.8 cm long, pilose, carrying a terminal umbel of (1) 2–3 (4) flowers, dull yellow in fresh state, turning brownish when dry at base. ***Bracts*** lanceolate, 4–5.5 mm long, minutely ciliate. ***Pedicel*** 0.4–1.4 cm long, shorter than leaf blade, pilose. ***Flowers*** distylous. ***Calyx*** campanulate, 8–9 mm long, parted to 1/2 of its length or slightly below; lobes linear lanceolate to lanceolate, apex acute. ***Corolla*** pastel violet, annulate, 5–6 lobes spreading, lobes broadly elliptic, 8.0–10.0 mm long, emarginate, densely yellow farinose abaxially, smooth adaxially. ***Pin flower***: corolla tubes 9.0–10.0 mm long, stamens inserted at middle of corolla tube, 9.5–10.5 mm long, style slightly exceeding the corolla tube mouth. ***Thrum flower***: corolla tubes 11.0–12.0 mm long, ca. 2 mm in diam., 2 times as long as the calyx, style 4.0–7.0 mm above base of corolla tube, stamens reaching the corolla tube mouth, 7.0–12.0 mm above base of corolla tube (Fig. 1G).

##### Phenology.

The flowering period is from December to February and the fruiting period is March to May.

##### Etymology.

The epithet “meishanensis” is derived from Meishan City, located in Sichuan Province, China.

##### Distribution and habitat.

**Figure 4.**
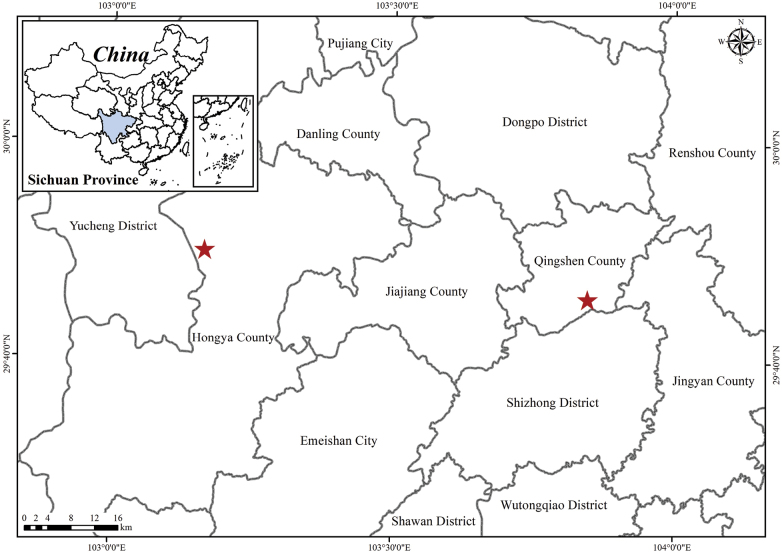
Location of the population of *Primulameishanensis* in Qingshen County and Hongya County, Meishan City and Sichuan Province (red star).

*P.meishanensis* is currently known from its type locality in Zhongyan temple, Ruifeng Town, Qingshen County and roadside of Panjiaozui, forest Hongya Forest Farm, Hongya County, Meishan City, Sichuan Province, China (Fig. 4). This new species probably exists in other localities. It grows on moist rock surfaces amidst moss under the forest, at elevations of approximately ca. 400–1000 m (Fig. 2).

##### Additional specimens examined.

China, Sichuan, Meishan City, Qingshen County, 29°45′47.39″N, 103°50′44.017″E, 26 December 2023 (fl.), Ke Huang & Zhixi Fu 8201 (SCNU!); Hongya County, 29°49'57.23"N, 103°09'50.23"E, 28 May 2015 (fl.), Ya J.D. & Hu X.J. 15CS11038 (KUN!)

##### Conservation status.

Data Deficient (DD). Currently, two populations with more than 100 individuals have been found in the Qingshen and Hongya Counties. The population of *P.meishanensis* inhabits moist rocks. Given its currently limited occurrence near the temple, there is a significant likelihood that the taxon may also be found in other localities. Until we have fully investigated the situation, it would be suggested to assess the conservation status of the species as Data Deficient (DD) following the IUCN Red List Criteria (IUCN 2024).

##### Relationship with related species.

Critical examination of collected specimens, comparison with type material of allied taxa and relevant taxonomic literature revealed that *P.meishanensis* is a new member of the P.sect.Petiolares. Morphologically, *P.meishanensis* shares certain similarities with *P.dejuniana*. However, *P.meishanensis* differs from *P.dejuniana* in featuring the leaf apex acute (vs. the leaf apex acute, but with a small point at tip), corolla pastel violet (vs. the corolla pale rose-purple), scape solitary, 7.2–11.8 cm long, carrying a terminal umbel of (1) 2–3 (4) flowers (vs. the scape usually one per rosette, 8–12 (18) cm long, carrying a terminal umbel of 2–6 flowers), the length of petiole 2.5–5 cm (vs. 1–3 cm), basal bud scales rose red (vs. flesh pink) and flowering period is from December to February (vs. from early February to early March). The number of chloroplast genes etc. is different in the two species (Table 2, Fig. 6, Suppl. material 1). To some extent, *P.meishanensis* also resembles to *P.davidii* as a perennial herb with calyx and corolla. However, it differs from *P.davidii* in leaf blade margin (sharply vs. erose-dentate) and apex (acute vs. rounded). The species of *P.meishanensis* bears similarities to *P.epilosa*, yet the former is readily distinguished by its leaf apex (acute vs. rounded), corolla (pastel violet vs. rose-purple with a yellow eye) and altitude (400–420 m vs. 2000–2900 m). Further morphological comparisons amongst the species of *P.meishanensis*, *P.davidii*, *P.dejuniana* and *P.epilosa* are shown in Table 1.

**Table 1.** Morphological characters comparison amongst *P.meishanensis* and closely-related species of *P.dejuniana*, *P.breviscapa* and *P.epilosa*.

Features	*P.meishanensis*	*P.dejuniana*	*P.epilosa*	*P.davidii*
Roots	numerous, without hairs	numerous, without hairs	few, without hairs	numerous, without hairs
Leaf blade	**smooth**, oblanceolate, 19.5–25 × 2.5–5 cm	rough, oblanceolate, 8–13(22) × 2–3(5.5) cm	rough, oblong-obovate to oblong-oblanceolate, 5–10 × 2–4 cm	rough, oblong to obovate-oblong, (5–)8–18 × 1.5–4 cm
Petiole	**2.5−5 cm long**	1−3 cm long	0.5−2.5 cm long	indistinct to nearly obsolete
Leaf apex	acute	Acute, but with a small point at tip	rounded	rounded
Leaf margin	ciliate, sharply and remotely dentate	ciliate, sharply and remotely dentate	hydathode-dentate	erose-dentate
Scape	solitary, 7.2–11.8 cm long, pilose, carrying a terminal umbel of (1)2–3(4) flowers	usually 1 per rosette, 8–12(–18) cm long, pilose, carrying a terminal umbel of 2–6 flowers	3.5–14 cm, sparsely glandular; umbel solitary, 2–5 flowered	8–20 cm, rust-coloured pilose, umbels 2–10-flowered
Corolla	**pastel violet**	pale rose-purple	rose-purple with a yellow eye	pale rose-purple
Pin flowers	corolla tube 9.0–10.0 mm long, stamens inserted at middle of corolla tube, style slightly exceeding the corolla tube mouth	corolla tube ca. 1.8 cm long, stamens inserted at middle of corolla tube, style reaching annulus	corolla tube ca. 1 cm; stamens ca. 4 mm above base of corolla tube; style slightly exserted	stamens ca. 3.5 mm above base of corolla tube; style ca. as long as tube
Thrum flowers	corolla tubes 11.0–12.0 mm long, ca. 2 mm in diam., style 4.0–7.0 mm above base of corolla tube	corolla tube ca. 2 cm long, style ca. 9 mm long reaching to middle of corolla	corolla tube 1.4–1.7 cm; style 4–5.5 mm	unknown
Altitude	**400–1000 m**	618–979 m	2000–2900 m	ca. 1000 m
Flowering	**December to February**	early February to early March	April to May	April

**Table 2.** Molecular analysis for the species.

Species	Family	Genus	GenBank number
*Ardisiapolysticta* Miq.	Myrsinaceae	*Ardisia* Sw.	KC465962
*Maesamontana* A.DC.	Myrsinaceae	*Maesa* Forssk.	KU569490
*Primulabracteata* Franch.	Primulaceae	*Primula* L.	NC053592
*Primulabulleyana* Forrest	Primulaceae	*Primula* L.	NC046947
*Primulacalliantha* Franch.	Primulaceae	*Primula* L.	ON804895
*Primulachrysochlora* Balf.f. & Kingdon-Ward	Primulaceae	*Primula* L.	KX668178
*Primulachungensis* Balf.f. & Kingdon-Ward	Primulaceae	*Primula* L.	NC050245
*Primuladenticulata* Wight	Primulaceae	*Primula* L.	NC050247
*Primuladryadifolia* Franch.	Primulaceae	*Primula* L.	NC053596
*Primulafilchnerae* R.Knuth	Primulaceae	*Primula* L.	NC051972
*Primulaforrestii* Balf.f.	Primulaceae	*Primula* L.	NC053602
*Primulahandeliana* W.W.Sm. & Forrest	Primulaceae	*Primula* L.	MG181221
*Primulajiugongshanensis* J.W.Shao	Primulaceae	*Primula* L.	NC056335
*Primulaknuthiana* Pax	Primulaceae	*Primula* L.	MG181223
*Primulakwangtungensis* W.W.Sm.	Primulaceae	*Primula* L.	KX774737
*Primulamatthioli* (L.) V.A.Richt.	Primulaceae	*Primula* L.	KY235373
*Primulamoupinensis* Franch.	Primulaceae	*Primula* L.	NC050244
*Primulaobconica* Hance, J. Bot.	Primulaceae	*Primula* L.	NC046415
*Primulaodontocalyx* Pax	Primulaceae	*Primula* L.	NC065386
*Primulaoreodoxa* Franch.	Primulaceae	*Primula* L.	NC050848
*Primulapellucida* Franch.	Primulaceae	*Primula* L.	NC050248
*Primulapersimilis* G.Hao, C.M.Hu & Y.Xu	Primulaceae	*Primula* L.	KX641757
*Primulapoissonii* Franch.	Primulaceae	*Primula* L.	KF753634
*Primulapulchella* Franch.	Primulaceae	*Primula* L.	NC050246
*Primularanunculoides* F.H.Chen	Primulaceae	*Primula* L.	NC056361
*Primulasikkimensis* Hook.	Primulaceae	*Primula* L.	NC050243
*Primulasinensis* Lour.	Primulaceae	*Primula* L.	KU321892
*Primulastenodonta* Balf.f. ex W.W.Sm. & H.R.Fletcher	Primulaceae	*Primula* L.	KX668176
*Primulaszechuanica* Pax	Primulaceae	*Primula* L.	NC080275
*Primulatsiangii* W.W.Sm.	Primulaceae	*Primula* L.	NC046755
*Primulaveris* L.	Primulaceae	*Primula* L.	KX639823
*Primulawilsonii* Dunn	Primulaceae	*Primula* L.	MW442886
*Primulawoodwardii* Balf.f.	Primulaceae	*Primula* L.	MG181222
*Primuladejuniana* G.Hao, C.M.Hu & Yuan Xu	Primulaceae	*Primula* L.	PQ213817
*Primulameishanensis*	Primulaceae	*Primula* L.	PQ213816

### ﻿Molecular phylogeny

**Figure 5.**
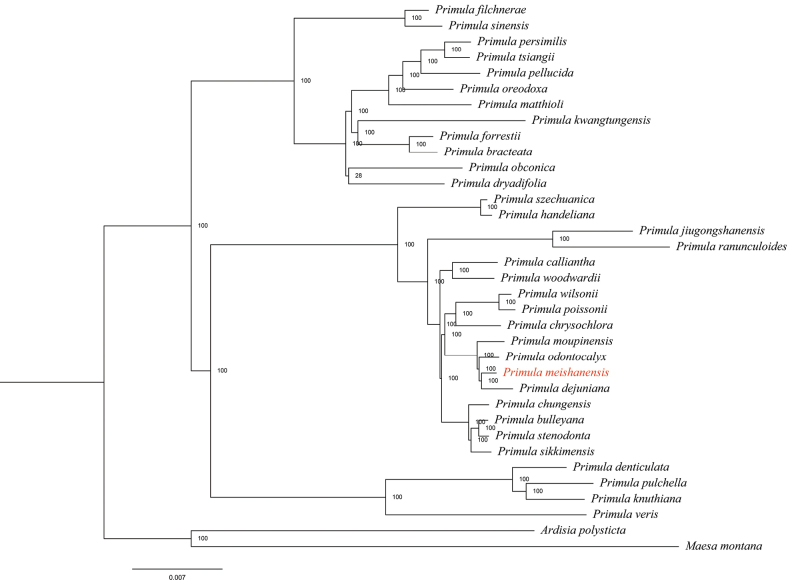
Phylogenetic tree reconstruction of 35 taxa using the ML method, based on complete cp genomes (the phylogenetic position of *Primulameishanensis* is marked with red circle.)

**Figure 6.**
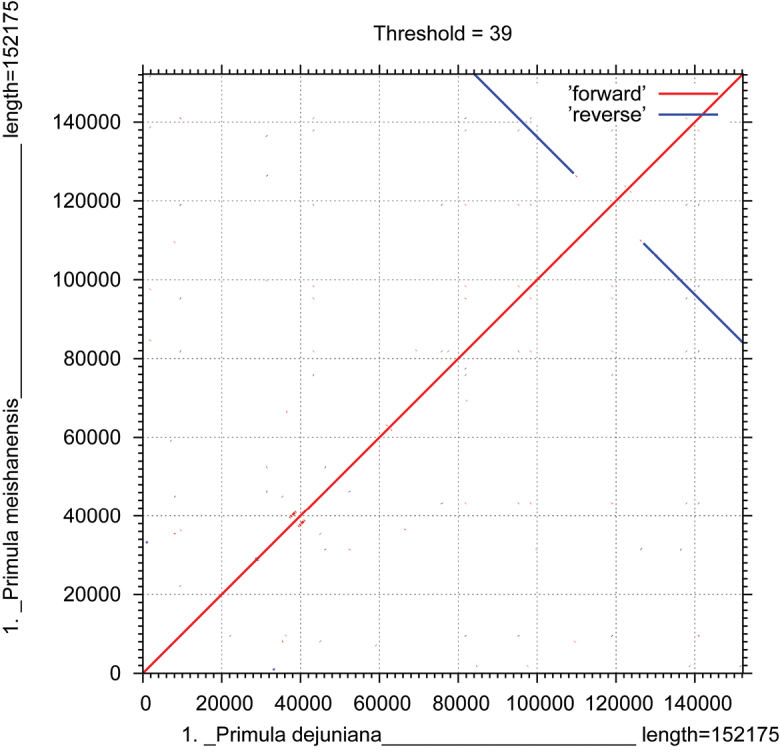
The covariance analyses of two species.

**Figure 7.**
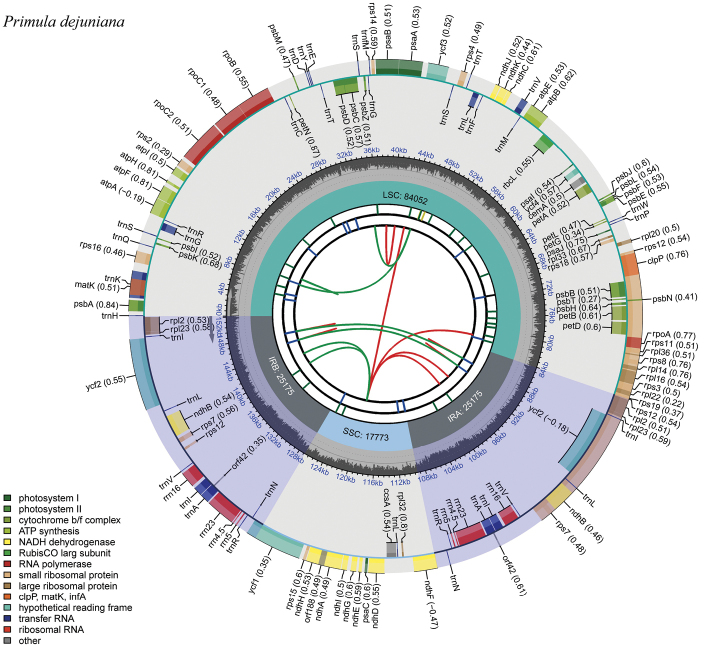
Circular map of *Primuladejuniana*. The map of complete chloroplast genome was generated using CPGView (http://www.1kmpg.cn/cpgview).

**Figure 8.**
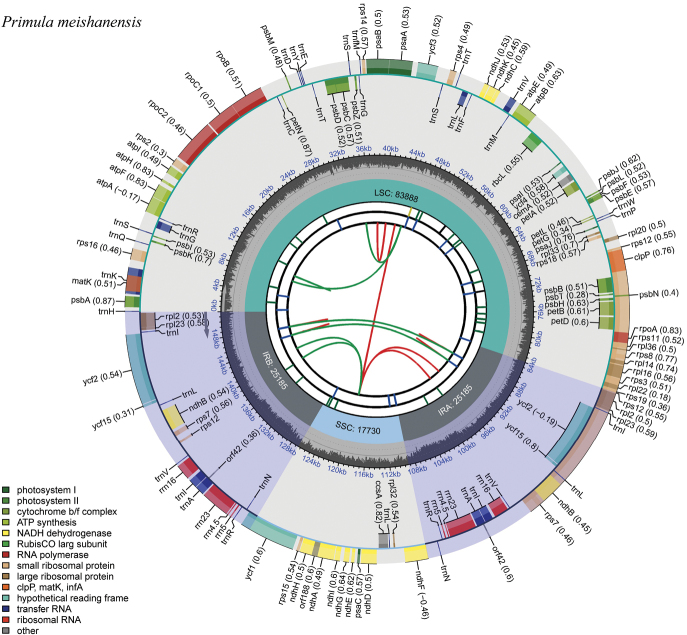
Circular map of *Primulameishanensis*. The map of complete chloroplast genome was generated using CPGView (http://www.1kmpg.cn/cpgview).

**Figure 9.**
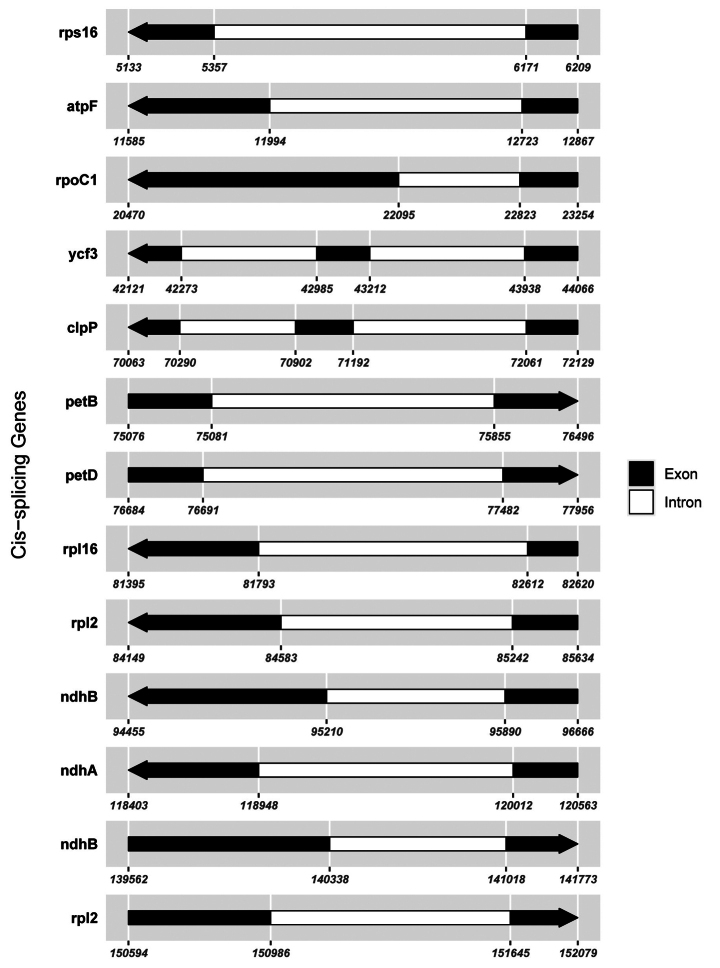
Schematic map of the cis-splicing genes in the chloroplast genome of *Primuladejuniana*.

**Figure 10.**
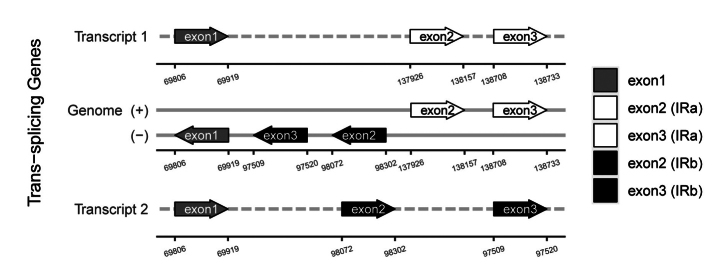
Schematic map of the trans-splicing genes in the chloroplast genome of *Primuladejuniana*.

**Figure 11.**
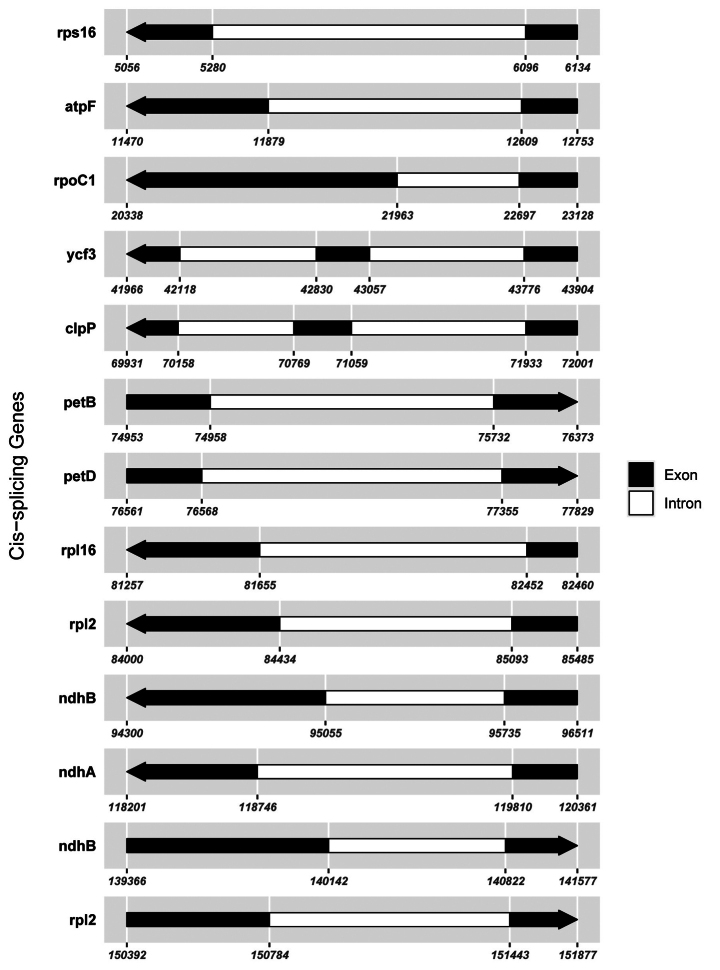
Schematic map of the cis-splicing genes in the chloroplast genome of *Primulameishanensis*.

**Figure 12.**
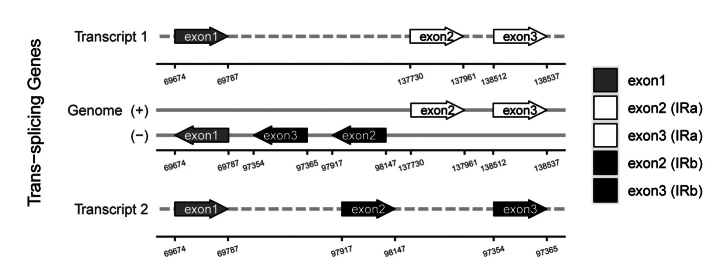
Schematic map of the trans-splicing genes in the chloroplast genome of *Primulameishanensis*.

In this study, 35 chloroplast genome sequences and measured chloroplast genome sequence of *P.meishanensis* were used to construct the ML evolutionary tree (Table 2) (Fig. 5). *Primulameishanensis* is sister to *Primuladejuniana*, forming a highly supported clade. However, the branch lengths of the two species are different, so they should be two species. The number of chloroplast genes etc. is not the same in the two species (Table 3). The complete chloroplast genome of *Primuladejuniana* is 151,988 bp, with the GC content of 37.01% (Fig. 7). The LSC length of *Primuladejuniana* is 83,888 bp, SSC length is 17,730 bp and IR length is 25,185 bp. In the nucleotide sequence of the complete chloroplast gene of the *Primuladejuniana*, the number of A is 47,313, the number of T is 48,431, the number of C is 28,628 and the number of G is 27,616. The complete chloroplast genome of *Primulameishanensis* is 152,175 bp, with the GC content of 36.93% (Fig. 8). The LSC length of *Primulameishanensis* is 84,052 bp, SSC length is 17,773 bp and IR length is 25,175 bp. In the nucleotide sequence of the complete chloroplast gene of the *Primulameishanensis*, the number of A is 47,469, the number of T is 48,508, the number of C is 28,613 and the number of G is 27,585. These complete cp genomes sequences are aligned by MAFFT (Fig. 6). We obtained a fasta file for the comparison of the two species and put it in the additional file (see Suppl. material 1). The cis-splicing genes are the same, but their positions in the sequence are different (Figs 9, 11). The *rps12* is a trans-splicing gene (Figs 10, 12).

**Table 3.** Comparative analyses of cp genomes between the two species.

Species	Genome Size (bp)	LSC (bp)	IR (bp)	SSC (bp)	A	T	C	G	GC Content (%)
*Primuladejuniana*	151,988	83,888	25,185	17,730	47,313	48,431	28,628	27,616	37.01
*Primulameishanensis*	152,175	84,052	25,175	17,773	47,469	48,508	28,613	27,585	36.93

## Supplementary Material

XML Treatment for Primula meishanensis

## ﻿Additional information

### Author ORCIDs

Tingyu Li https://orcid.org/0009-0005-6873-2114

Xinyu Chen https://orcid.org/0009-0008-6347-2490

Bo Li https://orcid.org/0000-0002-5131-8639

Donglai Hua https://orcid.org/0000-0002-7778-2608

Can Luo https://orcid.org/0009-0007-1815-6926

Huixian Luo https://orcid.org/0009-0002-8332-5494

Yun Liang https://orcid.org/0009-0009-9005-4662

Jieli Yue https://orcid.org/0009-0002-8786-2473

Xiaodan Xi https://orcid.org/0009-0008-4917-6345

Ke Huang https://orcid.org/0009-0006-2057-5452

Zhixi Fu https://orcid.org/0000-0002-2789-6287

### Data availability

All of the data that support the findings of this study are available in the main text or Supplementary Information.

## Supplementary materials

Supplementary material 1 Supplementary data Data type docx https://binary.pensoft.net/file/1159701This dataset is made available under the Open Database License (http://opendatacommons.org/licenses/odbl/1.0/). The Open Database License (ODbL) is a license agreement intended to allow users to freely share, modify, and use this Dataset while maintaining this same freedom for others, provided that the original source and author(s) are credited.Tingyu Li, Xinyu Chen, Bo Li, Donglai Hua, Can Luo, Huixian Luo, Yun Liang, Jieli Yue, Xiaodan Xi, Ke Huang, Zhixi Fu
