# Supplementary material for: ﻿Primulameishanensis (Primulaceae), a new species from Sichuan, China
Source: PhytoKeys. 2024 Oct 23;248:73–90. doi: 10.3897/phytokeys.248.127117 (PMC11525204; doi:10.3897/phytokeys.248.127117)
Supplement: Supplementary material 1 — Supplementary data [file phytokeys-248-073_article-127117__-s001.docx]

CLUSTAL format alignment by MAFFT (v7.511)

baochun2 agtgatgggcgaacgacgggaattgaacccgcgcatggtggattcacaatccactgcctt

baochun1 ----atgggcgaacgacgggaattgaacccgcgcatggtggattcacaatccactgcctt

********************************************************

baochun2 aatccacttggctacatccgccccctgaattccactattttatttacattagaaaaggaa

baochun1 aatccacttggctacatccgccccctgaattccactattttatttacattaaaaaaggaa

***************************************************.********

baochun2 aaatagtctattttaacccgtcatcattatttagttcatattttataaaatatgtaaaga

baochun1 aaatagtctattttaacccgtcatcattatttagttcatattttataaaatatgtaaaga

************************************************************

baochun2 aaaagagtcaatgaaacaaaaaaagacaaagtaatataaagtataaattaatacaaaata

baochun1 aaa------aatgaaacaaaaaaagacaaagtaatataaagtataaattaatacagaata

*** **********************************************.****

baochun2 aaaatgaaaagacgatacttaatcatgaactaactcatgacccccttttttatttattag

baochun1 aaaatgaaaagacgatacttaatcatgaactaactcatga-ccccttttttatttattag

**************************************** *******************

baochun2 ttctagtagtcttttatttacatatactttttcattagtaattagtaagaagaaagcttt

baochun1 ttctagtagtcttttatttacatatactttttcatt-------agtaagaagaaagcttt

************************************ *****************

baochun2 ttatgagaaaaaagtaaaggagcaataacgtcctcttgatagaacaagaattttgttatt

baochun1 ttatgagaaaaaagaaaaggagcaataacgtcctcttgatagaacaagaattttgttatt

************** *********************************************

baochun2 gctcctttactttcaaaaactcatatacaccaagaaaaaattcttatccatttgtagatg

baochun1 gctcctttactttcaaaaactcatatacaccaagaaaaaattcttatccatttgtagatg

************************************************************

baochun2 gagcttcaatcgcagctaggtctagagggaagttgtgagcattacgttcatgcataactt

baochun1 gagcttcaatcgcagctaggtctagagggaagttgtgagcattacgttcatgcataactt

************************************************************

baochun2 ccataccaaggttagcacgattaatgatatcagcccaagtattaattacacgaccttgac

baochun1 ccataccaaggttagcacgattaatgatatcagcccaagtattaattacacgaccttgac

************************************************************

baochun2 tatcaactacggattggttgaaattgaaaccatttaggttgaaagccatagtgctgatac

baochun1 tatcaactacggattggttgaaattgaaaccatttaggttgaaagccatagtgctgatac

************************************************************

baochun2 ctaaagcagtgaaccagatacctacgacaggccaagcagctaagaagaaatgtaaagaac

baochun1 ctaaagcagtgaaccagatacctacgacaggccaagcagctaagaagaaatgtaaagaac

************************************************************

baochun2 gagagttattgaaactagcatattggaagattaatcggccaaaataaccatgagcagcta

baochun1 gagagttattgaaactagcatattggaagattaatcggccaaaataaccatgagcagcta

************************************************************

baochun2 cgatattataagtttcttcctcttgaccaaatctgtaaccttcattagcagattcatttt

baochun1 cgatattataagtttcttcctcttgaccaaatctgtaaccttcattagcagattcatttt

************************************************************

baochun2 ctgtggtttccctgatcaaactagaagttactaaggaaccatgcatagcgctgaataggg

baochun1 ctgtggtttccctgatcaaactagaagttaccaaggaaccatgcatagcactgaataggg

*******************************.*****************.**********

baochun2 agccgccgaatacaccagctacacctaacatgtgaaatgggtgcataaggatgttgtgct

baochun1 agccgccgaatacaccagctacacctaacatgtgaaatgggtgcataaggatgttgtgct

************************************************************

baochun2 cagcctggaatacgatcatgaagttgaaagtaccagagattcctagaggcataccatcag

baochun1 cagcctggaatacgatcatgaagttgaaagtaccagagattcctagaggcataccatcag

************************************************************

baochun2 aaaaacttccttgaccaattgggtagatcaagaaaacagcagtcgcagctgcaacaggag

baochun1 aaaaacttccttgaccaattgggtagatcaagaaaacagcagtcgcagctgcaacaggag

************************************************************

baochun2 ctgaatatgcaacagcaatccaaggtcgcatacccaaacggaaactaagttcccactcac

baochun1 ctgaatatgcaacagcaatccaaggtcgcatacccaaacggaaactaagttcccactcac

************************************************************

baochun2 gacccatgtaacaagctacaccaagtaagaagtgtagaacaattagctcataaggaccgc

baochun1 gacccatgtaacaagctacaccaagtaagaagtgtagaacaattagctcataaggaccgc

************************************************************

baochun2 cattgtataaccattcatcaacagatgcagcttcccagattgggtaaaagtgcaaaccaa

baochun1 cattgtataaccattcatcaacagatgcagcttcccagattgggtaaaagtgcaaaccaa

************************************************************

baochun2 tagctgcagaagtaggaataatggcaccggaaataatattgtttccgtaaagtagagatc

baochun1 tagctgcagaagtaggaataatggcaccggaaataatattgtttccgtaaagtagagatc

************************************************************

baochun2 cagaaacaggttcacgaataccatcaatatctactggaggagcagcaatgaaggcgataa

baochun1 cagaaacaggttcacgaataccatcaatatctactggaggagcagcaatgaaggcgataa

************************************************************

baochun2 taaatacagaagttgcggttaataaggtagggatcatcaaaacaccaaaccatccaatgt

baochun1 taaatacagaagttgcggttaataaggtagggatcatcaaaacaccaaaccatccaatgt

************************************************************

baochun2 aaagacggttttcagtgctagttatccagttacagaagcgaccccataggctttcgcttt

baochun1 aaagacggttttcagtgctagttatccagttacagaagcgaccccataggctttcgcttt

************************************************************

baochun2 cgcgtctctctaaaattgcagtcatggtaaaatcttggtttatttaattatcagggactc

baochun1 cgcgtctctctaaaattgcagtcatggtaaaatcttggtttatttaattatcagggactc

************************************************************

baochun2 ccaagcacacgaattttcataaatagaaataaaaaatggacggcttgttattgaacagta

baochun1 ccaagcacacgaattttcataaatagaaataaaaaatggatggcttgttattgaacagta

****************************************.*******************

baochun2 taacatgacttatatgcccgtgtcaaccaatacctatctagatctatcataattttttat

baochun1 taacatgacttatatgcccatgtcaaccaatacctatctagatctatcataattttttat

*******************.****************************************

baochun2 aaatgaatagagttact-aaaaaaaaatagaatacaggtttatatacctatgtgacattg

baochun1 aaatgaatagagttactaaaaaaaaaatagaatacaggtttctatacctatgtgacattg

***************** *********************** ******************

baochun2 ggttgcccgggattcgaacccggaactagtcggatggagtagataaattccttgttaaac

baochun1 ggttgcccgggattcgaacccggaactagtcggatggagtagataaattccttgttaaac

************************************************************

baochun2 aagcaaaaacccctccccaagccgtgcttgcatttttcattgcacacggctttccctatg

baochun1 aagcaaaaacccctccccaagccgtgcttgcatttttcattgcacacggctttccctatg

************************************************************

baochun2 tatacatataaaactccgttctttccttagacaaggaaaaggattgaagactcagtgaat

baochun1 tatacatataaaactccgttctttctttagacaaggaaaaggattgaag---------at

*************************.*********************** **

baochun2 ttaagccttaactagatgatacatgaacatttcataatctaatttatttgttatatttt-

baochun1 ttaagccttaactagatgatacatgaacatttcataatctaatttatttgttatattttt

***********************************************************

baochun2 -----ttattatttaaagttaatgttcatttacacggtctgataacaaatcattcatgat

baochun1 tattattattatttaaaattaattttcatttacacggtctgataacaaatcattcatgat

************.***** ************************************

baochun2 ttgccagatcattgatacaaaaaatatccaaataccaaattcgctttctatataagcctc

baochun1 ttgccagatcattgatacaaaaaatatccaaataccaaattcgctttctatataagcctc

************************************************************

baochun2 ccgaagtagaagaagcttttgggaaggtcaaagaaaaaatgagctcttccgccataaaga

baochun1 ccgaagtagaagaagcttttgggaaggtcaaagaaaaaatgagctcttccgccataaaga

************************************************************

baochun2 attcttccaataattccgggcctattcttttcaaaaaaacacgtaccgtacttttgtgtt

baochun1 attcttccaataattccgggcctattcttttcaaaaaagcacgtaccgtacttttgtgtt

**************************************.*********************

baochun2 tacgagccaaagttcgagcacaagagagtcgaattatatactttattcgatacaaactca

baochun1 tacgagccaaagttcgagcacaagagagtcgaattatatactttattcgatacaaactca

************************************************************

baochun2 ttttttttgaggatccgctataataatgagaaatatttctatatatacgcccaaatcggt

baochun1 ttttttttgaggatccgctataataatgagaaatatttctatatatacgcccaaatcggt

************************************************************

baochun2 caataatatccgaatcggataaatcggcccagactggcttactaatgggatgtcctaata

baochun1 caataatatccgaatcggataaatcggcccagactggcttactaatgggatgtcctaata

************************************************************

baochun2 ggttacaaaatttagctttagacaaggatccaataagaggaataattggaacaatcgtat

baochun1 ggttacaaaatttagctttagacaaggatccaataagaggaataattggaacaatcgtat

************************************************************

baochun2 caaacttcttactaaaattagctattagaaatgcattttctagcatttgattccgtacca

baochun1 cgaacttcttactagaattagctattagaaatgcattttctagcatttgattccgtacca

*.************.*********************************************

baochun2 ttgaagagtttagtgccacatttgaaagatagcccagaaggtcgagggaataatgagata

baochun1 ttgaagagtttagtgccacatttgaaagatagcccagaaggtcgagggaataatgggata

*******************************************************.****

baochun2 atttatttatatggatccttcctggttgaaaccacatgtaaaaataacattgccagaaat

baochun1 atttatttatatggatccttcctggttgaaaccacatgtaaaaataacattgccagaaat

************************************************************

baochun2 ttacaagataatagttccatttattcatcaaaagaaacggtccttttgaagccaaaaggg

baochun1 tgacaagataatagttccatttattcatcaaaagaaacggtccttttgaagccaaaaggg

* **********************************************************

baochun2 cttttccttgatacctaagataatgcatcaaaggatccttgaccgaacataaaatggcct

baochun1 cttttccttgatacctaagataatgcatcaaaggatccttgaccgaacataaaatggcct

************************************************************

baochun2 gaaaatattttgtaaatacttcgaaaaaatagtctcgtttttcatagaaatatattcggt

baochun1 gaaaatattttgtaaatacttcgaaaaaatagtctcgtttttcatagaaatatattcggt

************************************************************

baochun2 caagaaaaggtctataagcttttgattgtaaatgagaagattgattgcggagaaaggcga

baochun1 caagaaaaggtctataagcttttgattgtaaatgagaagattgattgcggagaaaggcga

************************************************************

baochun2 agaccgattcgtattcatatatatgaaaattatataaaaaaaataagaaccgttcctttc

baochun1 agaccgattcgtattcatatatatgaaaattatataaaaaaaataagaaccgttcctttc

************************************************************

baochun2 tttttgaaaaagaaaaactagatttctttatagtaataagattattccaattatgatact

baochun1 tttttgaaaaataaaaactagatttctttatagtaataagattattccaattatgatact

*********** ************************************************

baochun2 tgtgaagaaaggctcgtaataaatgcaaagaagaagcatcttttacccagtaacgaagag

baochun1 tgtgaagaaaggctcgtaataaatgcaaagaagaagcatcttttacccagtaacgaagag

************************************************************

baochun2 tttgaaccaatatttccagatggatggaatggggtataaataaatctaacacataattta

baochun1 tttgaaccaatatttccagatggatggaatggggtataaataaatctaacacataattta

************************************************************

baochun2 aacgtaaaaatttgtcctctaaaaaaggaaatattgaatgaattgatcgtaaattatgag

baochun1 aatgtgaaaatttgtcctctaaaaaaggaaatattgaatgaattgatcgtaaattatgag

**.**.******************************************************

baochun2 attttactatttttttcccttctagggaagatattacttgtatagaaaatggaatttcca

baochun1 attttactatttttttcccttctagggaagatattacttgtatagaaaatggaatttcca

************************************************************

baochun2 taacgactacaaatccttctaatataatttgagagtacaaagttgtgttgtgccccaaaa

baochun1 taacgactacaaatccttctaatataatttgagagtacaaagttgtgttgtgccccaaaa

************************************************************

baochun2 atggattttgattagaatcattaatcaaaaaacgaaaatgattctgtggatccgtttgag

baochun1 atggattttgattagaatcattaatcaaaaaacgaaaatgattctgtggatccgtttgag

************************************************************

baochun2 taattaaatgagtaattaaacgtttcacaatcagtaagctggagttattgtcataacctg

baochun1 taattaaatgagtaattaaacgtttcacaatcagtaagctggagttattgtcataacctg

************************************************************

baochun2 cctcttccaattctaacgaattagatctagttaaactatgattatgagcaagtacataaa

baochun1 cctcttccaattctaacgaactagatctagttaaactatgattatgagcaagtacataaa

********************.***************************************

baochun2 tatactcctgaaatataagtggatataggaagtcgtgttgctgaaatctatctagttcta

baochun1 tatactcctgaaatataagtggatataggaagtcgtgttgctgaaatctatctagttcta

************************************************************

baochun2 aatatcttttgaattcctccatgaattcctccatataaaattaaaatggaaccaaagata

baochun1 aatatcttttgaattcctccatgaattcctccatataaaataaaaatggaaccaaagata

***************************************** ******************

baochun2 gagggtttttggggggtttatcaaatgatacatagtgcgatatagtcaaaacaaggtatt

baochun1 gagggtttttggggggtttatcaaatgatacatagtgcgatatagtcaaaacaaggtatt

************************************************************

baochun2 gtgcgtaagaaaagatacctcggtaaacttattaacagattctctatcctttctttttcc

baochun1 gtgagtaagaaaagatacctcggtaaacttattaacagattctctatcctttctttttcc

*** ********************************************************

baochun2 attgaatttaattaatttatgttcgttataggataaaaagatggttagaaatcctttatt

baochun1 attgaatttaattaatttatgttcgttataggataaaaagatggttagaaatcctttatt

************************************************************

baochun2 ttttttcaacccaatcgctcttttgattttggaaaaaaaaatgatcaatatactatttct

baochun1 ttttttcaacccaatcgctcttttgattttgg-aaaaaaaatgatcaatatgctatttct

******************************** ******************.********

baochun2 tctacacaccaacctccattctataagggagggttcaatagttaggattcattataaaat

baochun1 tctacacaccaacctccattctataagggagggttcaatagttaggattcattataaaat

************************************************************

baochun2 tgataatccacatatgggaaaagtatttctcgcacccacacaagcactaatatattttta

baochun1 tgataatccacatatgggaaaagtatttctcgcacccgcacaagcactaatatattttta

*************************************.**********************

baochun2 acctataattagatggggtaatcattcaaattaagaacagaagctcgtagctctttcttt

baochun1 acctataattagatggggtaatcattcaaattaagaacagaagctcgtagctctttcttt

************************************************************

baochun2 ccctcgaattgattgaagccatagggctctatccatttatttattcgaccaaaggttttt

baochun1 ccctcgaattgattgaagccatagggctctatccatttatttattcgaccaaaggttttt

************************************************************

baochun2 tggttcatttcaagaattcaaacaaagttttgttttgtattgatctgttaagaataaaac

baochun1 tggttcatttcaagaattcaaacaaa-----gttttgtattgatctgttaagaataaaac

************************** *****************************

baochun2 agtttcagaactctccattgatacgacatgctattttttccattcattccccttcaggat

baochun1 agtttcagaactctccattgatacgacatgctattttttccattcattccccttcaggat

************************************************************

baochun2 cagtcgtggtcttccaaactttaccaattgtatggacgaatcccttacttcatccaaatg

baochun1 cagtcgtggtcttccaaactttaccaattgtatggacgaatcccttacttcatccaaatg

************************************************************

baochun2 tgtaaaagatcctagccgcacttaaaagccgagtactctaccattgagttagcaacccga

baochun1 tgtaaaagatcctagccgcacttaaaagccgagtactctaccattgagttagcaacccga

************************************************************

baochun2 ataaaaagaaaaggtgtgtcgaaataatcaaaatcaattaaataaattatgtgatgcaat

baochun1 ataaaaagaaaaggtgtgtcgaaataatcaaaatcaattcaataaattatgtgatgcaat

*************************************** ********************

baochun2 tcaaacattaaactagcagaaaaatttcgaatttattcggaacataaaaatgaactgatc

baochun1 ccaaacattaaactag------------------------------------------tc

.*************** **

baochun2 aaacgaaaatacaagaaattttcaaataaaa-aaaaaagagaaataagtataaaactaaa

baochun1 aaacgaaaatacaagaaattttcaaataaaagaaaaaagagaaataagtataaaactaaa

******************************* ****************************

baochun2 tagaccgcactaaaaaaaacttcttggatcaatcttgtatgaaaatgaattcatcattta

baochun1 tagaccgcactaaaaaaaacttcttggatcaatcttgtatgaaaatgaatccatcattta

**************************************************.*********

baochun2 tctttaagtaaaaaccaaacctatggtacgtaaataaatagatatacttaaaattatatt

baochun1 tctttaagtaaaaaccaaacctatggtacgtaaataaatagatatacttaaaattatatt

************************************************************

baochun2 tgttcgatgcactgttgtcaatataatatgaattttcgaaaataaaacaatagaaaaaaa

baochun1 tgttcgatgcactgttgtcaatataatatgaattttcgaaaataaaacaatag-aaaaaa

***************************************************** ******

baochun2 atatcccatttccattaaattcaaaaatatataaaatagatatttttgaatttaataaaa

baochun1 atatcccatttccattaaattcaaaaatatataaaatagatatttttgaatttaataaaa

************************************************************

baochun2 tattctaaatatttcaaaaaaggacttgtgttggattagcactacatagatacaaaaata

baochun1 tattctaaatatttcaaaaaaggacttgtgttggattagcactacatagatacaaaaata

************************************************************

baochun2 aattaagacattaagcaagtagaaaaagaaccgcaggtttattgaaatagaaatgaaata

baochun1 aattaagacattaagcaagtagaaaaagaaccgcgggtttattgaaatagaaatgaaata

**********************************.*************************

baochun2 agcaaactaaattgcttatttcgtaatttaatatttggtattttttaattttggtattta

baochun1 agcaaactaaattgcttatttcgtaatttaatatttggtat-------ttttggtattta

***************************************** ************

baochun2 tagtaataaagttctgacgaagactagcaaattgaagagaatgtcataatttttctagga

baochun1 tagtaataaagttctgacgaagactagcaaattgaagagaatgtcataatttttctagg-

***********************************************************

baochun2 ttaataaattaataaattctggttttactgttatagaacataggagagtaatgaattggc

baochun1 -------attaataaattctggttttactgttatagaacataggagagtaattaattggc

********************************************* *******

baochun2 atgaatagagtcaaagagataaagaaaaagttacggaagtgataactcctatatagtttt

baochun1 atgaatagagtcaaagagataaagaaaaagttacggaagtgataactcctatatagtttt

************************************************************

baochun2 atacaagccttaa------atctattatttttttttatttccttaattaatttgatttcg

baochun1 atacaagccttaatctattatctattatttttttttatttccttaattaatttgatttcg

************* *****************************************

baochun2 tttgattagagtgaaactccttaaaaacccccgctttctttaaaatatcctgaaccgttt

baochun1 tttgattagagtgaaactccttaaaaacccccgctttctttaaaatatcctgaaccgttt

************************************************************

baochun2 ctgtaggttgagcacctttctcaaggaaatatagaatagcaggaacatttaaataagttt

baochun1 ctgtaggttgagcacctttctcaaggaaatatagaatagcaggaacatttaaataagttt

************************************************************

baochun2 gattctttatcggatcataaaaacccactttccgaagatctcgtccttctcttcgggacc

baochun1 gattctttatcggatcataaaaacccactttccgaagatctcgtccttctcttcgggacc

************************************************************

baochun2 gaacatcaattgcaacgattcgatagacggctcattgggatagatgtaaaaaagtaa---

baochun1 gaacatcaattgcaacgattcgatagacggctcattgggatagatgtaaaaaagtaaccc

*********************************************************

baochun2 ccccccctcgaaacgtataagaagttttctcctcgtacggctcattcaaaataattttta

baochun1 ccccccctcgaaacgtataagaagttttctcctcgtacggctcattcaaaataattttta

************************************************************

baochun2 gttctacttaatgtatagaatcacaaatgggtctataaaaaaatggttgaaatccccctt

baochun1 gttctacttaatgtatagaatcacaaatgggtctataaaaaaatggttgaaatcccccct

**********************************************************.*

baochun2 ttttattcttacttccttaaaaaaaaatcatttgtactcataactcaagttagataactc

baochun1 ttttattcttacttccttaaaaaaaaatcatttgtactcataactcaagttagataactc

************************************************************

baochun2 tccagtggcttaaaggaaaatatttaagcatttcatttattgagtggtcttgaaacctct

baochun1 tccagtggcttaaaggaaaatatttaagcatttcatttattgagtggtcttgaaacctct

************************************************************

baochun2 cttttttgtttcttttatttcaaatctatttgaatttttattatggtacaattatggaaa

baochun1 cttttttgtttcttttatttcaaatctatttgaatttttattatggtacaattatggaaa

************************************************************

baochun2 caatggaaaagatcttttcttgttttgggatcttttaatctttgttttaaatcattgggt

baochun1 caatggaaaagatcttttcttgttttgggatcctttaatctttgttttaaatcattgggt

********************************.***************************

baochun2 ttagacataacttcggtgattcttaatcctttcaaaatggcagcaacatacctttttttg

baochun1 ttagacataacttcggtgattcttaatcctttcaaaatggcagcaacatacctttttttg

************************************************************

baochun2 cgattgatttctaataaagaatcatagaaagggttgattctcatataataaactttgtat

baochun1 cgattgatttctaataaagaatcatagaaagggttgattctcatataataaactttgtat

************************************************************

baochun2 ggaaagaattttttcaattccaacaaattttattttagattgaaaacgcgaaaccctttc

baochun1 ggaaagaattttttcaattccaacaaattttattttagattgaaaacgcgaaaccctttc

************************************************************

baochun2 aatttctctatcaacgatatacttacgaagttcttccaatttattgattggcattaacca

baochun1 aatttctctatcaacgatatacttacgaagttcttccaatttattgattggcattaacca

************************************************************

baochun2 tagacctttgcccctgagaaatgaatcaataatttctactcgagctccatcatcgactat

baochun1 tagacctttgcccctgagaaatggatcaataatttctactcgagctccatcatcgactat

***********************.************************************

baochun2 ttccattacaacccgatgggtttgtagtgaaacagaataaatgatgtcgagtcaagagca

baochun1 ttccattacaacccgatgggtttgtagtgaaacagaataaatgatgtcgagtcaagagca

************************************************************

baochun2 ccttcattcttatataaaatggtggatgtaaaaatccacaatggatcatgtctttccaag

baochun1 ccttcattcttatataaaatggtggatgtaaaaatccacaatggatcatgtcttt-caag

******************************************************* ****

baochun2 tcgcacgttgctttctaccacatcgtttcaaacgaagttttaccataacatttctctaat

baochun1 tcgcgcgttgctttctaccacatcgtttcaaacgaagttttaccataacatttctctaat

****.*******************************************************

baochun2 ttggaaccggtatggaattgattcaattatggaatcgtgaataatcattggttcattcgc

baochun1 ttggaaccggtatggaattgattcaattatggaatcgtgaataatcattggttcattcgc

************************************************************

baochun2 tacatagtactctatcacttttcatctagatagatggatagaaatttttatgaagaggtt

baochun1 tacatagtactctatcacttttcatctagatagatggatagaaatttttctgaagaggtt

************************************************* **********

baochun2 ttagcacgaattcaacgtaactctaattttttttattgtataatataaatacaagatttt

baochun1 ttagcacgaattcaacgtaactccaattttttttattgtatagtataaatacaaga-ttt

***********************.******************.************* ***

baochun2 ttttttaattatcaaaattattatattctaaaaatataaataaaaagggaaaaattgaaa

baochun1 tttttgaattatcaaaattattatattctaaaaatataaataaaaaaggaataatttaaa

***** ****************************************.**** **** ***

baochun2 ttttttgtcgtttatttttatgaaatgaataaaaaagactgatgggagggaagacttgag

baochun1 ttttttgtcgtttatttttatgaaatgaataaaaaagactgatgggagggaagacttgag

************************************************************

baochun2 tccttattatttctattcttattttatcaaatcgctattaaagaatagttcctatcttat

baochun1 tccttattatttctattcttattttctcaaatcgctattaaagaatagttcctatcttat

************************* **********************************

baochun2 agatattctgtgttaaatatggtttaaatattgaaatttgcctttttcaatttaagaaat

baochun1 agatattctgtgttaaatatggtttaaatattgaaatttgcctttttcaatttaagaaat

************************************************************

baochun2 cctaaaatttttgtttcacaacgaaaaacgactctcactgaaatagaactatagagcaat

baochun1 cctaaaatttttgtttcacaacgaaaaacgactctcactgaaatagaactatagagcaat

************************************************************

baochun2 aataatatatacatatagactatgcatttcctagttttatatacgtattttcatattttg

baochun1 aataatatatacatatagactatgcatttcctagttttatatacgtattttcatattttg

************************************************************

baochun2 tttaacttaatatttcagtaatatttcgataaattctctgggaataaaaaaaaataagaa

baochun1 tttaacttaatatttcagtaatatttcgataaattctctgggaataaaaaaaaataagaa

************************************************************

baochun2 ttgtattctattcaatattagacctttaaacataaatagaaagttgttcacaagaatctt

baochun1 ttgtattctattcaatattagacctttaaacataaatagaaagttgttcacaagaatctt

************************************************************

baochun2 attctaatcaaatttagatgatttagaatggaatagggtctgggacggaaggattcgaac

baochun1 attctaatcaaatttagatgatttagaatggaatagggtctgggacggaaggattcgaac

************************************************************

baochun2 ctccgaataccgggatcaaaacccgttgccttaccacttggccacgccccattaaaattt

baochun1 ctccgaataccgggatcaaaacccgttgccttaccacttggccacgccccattaaaattt

************************************************************

baochun2 ctattcaacactaataaagaataatgctggtattagttgatagtcaattctaatacaaat

baochun1 ctattcgacactaataaagaataatgctgatattagttgatagtcaattctaatacaaat

******.**********************.******************************

baochun2 aaatatcgaatttagaaaatatattcttactaagatttttatatgtgtatatatagaata

baochun1 aaatatcgaatttagaaaatatattcttactaagatttttatatgtgtatatatagaata

************************************************************

baochun2 aaatttaatttattgatcattacatataattcaattaagatattgtatgaaagtcgaatt

baochun1 aaatttaatttattgatcattacatataattcaattaagatattgtatgaaagtcgaatt

************************************************************

baochun2 tcttctattccatttgagaatgggagggtttttggttgggtgaattcaaagactaaagaa

baochun1 tcttctattccatttgagaatgggagggtttttggttgggtgaattcaaagactaaagaa

************************************************************

baochun2 gaattttttctaccttactttcttctttttgacttcatatcaataactcaatcaaaattc

baochun1 gaattttttctaccttactttcttctttttgacttcatatcaataactcaatcaaaattc

************************************************************

baochun2 aattatctccaagaacaaaatgtctgttatgcttaatatctttagtttgatctgtattaa

baochun1 aattatctccaagaacaaaatgtctgttatgcttaatatctttagtttgatctgtattaa

************************************************************

baochun2 ttcggctctttattcgagtcattttgtcttcgctaaattgccggaggcctatgctttttt

baochun1 ttcggctctttattcgagtcattttgtcttcgccaaattgccggaggcctatgctttttt

*********************************.**************************

baochun2 gaatccgattgtagatgttatgccagtcatacctctgtttttttttctcttagcctttgt

baochun1 gaatccgattgtagatgttatgccagtcatacctctgtttttttttctcttagcctttgt

************************************************************

baochun2 ttggcaagctgctgtaagttttcgctgagatcttgaatattatatcctataaaattcagg

baochun1 ttggcaagctgctgtaagttttcgctgagatcttgaatattatatcctaaaaaattcagg

************************************************* **********

baochun2 atttatttcgaaaattctatcaattggatcagataagtctcataataatgaaccctcaat

baochun1 atttatttcgaaaattctatcaattggatcagataagtctcataataatgaaccctcaat

************************************************************

baochun2 tcaaagaaactcttgactagacgcaagaaatctaaattatcccattttgtttgccagtga

baochun1 tcaaagaaactcttgactagacgcaagaaatctaaatcatcccattttgtttgccagtga

*************************************.**********************

baochun2 aagacactaatcctattagtatcagagtcttctaaaatagataattttggatatgaaatg

baochun1 aagacactaatcctattagtatcagagtcttctaaaatagataattttggatatgaaatg

************************************************************

baochun2 caattttagtaatgaaagactcttatgacaaaagaattttcataaattctcaattttttc

baochun1 caattttagtaatgaaagactcttatgacaaaagaattttcataaattctccattttttc

*************************************************** ********

baochun2 tatttctagaaggcacttcatttcgtgatgtcaaaataggattaggatatgtggtataaa

baochun1 tatttctagaaggcacttcatttcgtgatgtcaaaataggattaggatatgtggtataaa

************************************************************

baochun2 aaaaagacgatctattcccccttttccccaaaaaatgatcttggagattgtgtaatgctt

baochun1 aaaaagacgatctattcccccttttccccaaaaaatgatcttggagattgtgtaatgctt

************************************************************

baochun2 actctcaaacttttcgtttatacagtagtgatattctttgtttctctcttcatctttgga

baochun1 actctcaaacttttcgtttatacagtagtgatattctttgtttctctcttcatctttgga

************************************************************

baochun2 ttcctatctaatgatccagggcgtaatcctggacgtgaagaataaaatgaaaaaaatgat

baochun1 ttcctatctaatgatccagggcgtaatcctggacgtgaagaataaaatgaaaaaaatgat

************************************************************

baochun2 ttgaaatttttgaaagataaataaaattcaaatatcaaatcatcgagggaggtggaaaga

baochun1 ttgaaatttttgaaagataaataaaattcaaatatcaaatcatcgagggaggtggaaaga

************************************************************

baochun2 gagggattcgaaccctcggtacaaataacttgtacaacggattagcaatccgccgctttc

baochun1 gagggattcgaaccctcggtacaaataacttgtacaacggattagcaatccgccgctttc

************************************************************

baochun2 gtccactcagccatctctcccaattgaaaacaaattcctatgttacattacacataaagt

baochun1 gtccactcagccatctctcccaattgaaaacaaattcctatgttacattacacataaagt

************************************************************

baochun2 aaggattaaaaaaggctttctttctatttttttattatatagattattttattatataga

baochun1 aaggattaaaaaaggctttctttctattt-----------------ttttattatataga

***************************** **************

baochun2 ttagatgtgtataacttttatcaataattcaatttagatttagataaagtaagagctaaa

baochun1 ttagatgtgtataacttttatcagtaattcaatttagatttagataaagtaagagctaaa

***********************.************************************

baochun2 aggatccaattgttttcattttgatcgaaggggcccttttcttttactcccacgccccgg

baochun1 aggatccaattgttttcattttgatcgaaggggcccttttcttttactcccacgccccgg

************************************************************

baochun2 ctggctaggtcaataccaatacctagccaggcccttttttgttacaacgaatgacagata

baochun1 ctggctaggtcaataccaatacctagccaggcccttttttgttacaacgaatgacagata

************************************************************

baochun2 aagataaaaccctttatcggatttaaaaacggaaggacttgttagtaaattaaaacaact

baochun1 aagataaaaccctttatcggatttgaaaacggaaggacttgttagtaaattaaaacaact

************************.***********************************

baochun2 cgaaagtctcatttcttattatttgcttttactaccctttttctattttttgtaataaaa

baochun1 cgaaagtctcatttcttattatttgcttttactaccctttttctatttttt---------

***************************************************

baochun2 agtaataaaaactagatatataataaacaaaagaaactctggactcttacacaacgcatt

baochun1 -gtaataaaaactagatatataataaacaaaagaaactctggactcttacacaacgcatt

***********************************************************

baochun2 tttatgttatgattttagtgcttttagtgaatcgtctctatcaaaattacttcagtaaaa

baochun1 tttatgttatgattttagtgcttttagtgaatcgtctctatcaaaattacttcagtaaaa

************************************************************

baochun2 agaaagaaattcgtattttttttttagttatttaatcttttcctaatttaatccaatccc

baochun1 agaaataaattcgta-tttttttttagttatttaatcttttcctaattt-----aatccc

***** ********* ********************************* ******

baochun2 gcgaacacaaaaattaaagttaacactctaattttcatgattcgtttaggatcctatttt

baochun1 gcgaacacaaaaattaaagttaacactctaattttcatgattcgtttaggatcctatttt

************************************************************

baochun2 gattacgccaaatacctctgttcgacaaaagattcatttgtatacaataatcatattgta

baochun1 gattacgccaaatacctctgttcgacaaaagattcatttgtatacaataatcatattgta

************************************************************

baochun2 gcgggtatagtttagtggtaaaagtgtgattcgttttattaatccccttaatagttaagg

baochun1 gcgggtatagtttagtggtaaaagtgtgattcgttttattaatccccttaatagttaagg

************************************************************

baochun2 ggtccctcggtttaatttatattccgattaaaaactttttttcttaaaaggatgaaatcc

baochun1 ggtccctcggtttaatttatattccgattaaaaac-ttttttcttaaaaggatgaaatcc

*********************************** ************************

baochun2 tttacctctcaatgacttattcgaggaaaaatagaaattctcgtgatttgtatccaaagg

baochun1 tttacctctcaatgacttattcgaggaaaaatagaaattctcgtgatttgtatccaaagg

************************************************************

baochun2 tcaattcgaaattgaaaaattggattctaaaattgtgaaacataatttgtgaattggatt

baochun1 tcaattcgaaattgaaaaattggattctaaaattgcgaaacataatttgtgaattggatt

***********************************.************************

baochun2 cctacttccaattaaataattatgaatcaagatctatggcggaagatataaaagtgtatt

baochun1 cctacttccaattaaataattatgaatcaagatctatggcggaagatataaaagtgtatt

************************************************************

baochun2 tctaaccgtaactaaatcttcaattttgagttgatagagaagaaattgaagcaaaatagc

baochun1 tctaaccgtaactaaatcttcaattttgagttgatagagaagaaattgaagcaaaatagc

************************************************************

baochun2 tatgaaatgatgactttgatttactagagacatcgacatatttttttagctcggtgggaa

baochun1 tatgaaatgatgactttgatttactagagacatcgacatatttttttagctcggtgggaa

************************************************************

baochun2 caaagtactttttctaaggattttaaaaaatagaaatttaaagaacgaaggaactagaaa

baochun1 caaggtacttttcctaaggattttaaaaaatagaaatttaaagaacgaaggaactagaaa

***.********.***********************************************

baochun2 gattgttacaattatcttactctaacgggatcatctagaaagcaagtcttttttaattca

baochun1 gattgttacaattatcttactctaacgggatcatctagaaagcaagtcttttttaattca

************************************************************

baochun2 atatattcagacaaaaagctaacatagatgttatgggtagaattttcattcacatctttt

baochun1 atatattcagacaaaaagctaacatagatgttatgggtagaattttcattcacatctttt

************************************************************

baochun2 agatctcagaatttatgcatcttccataaaggagccgaatgaaaccaaagtttcatgttc

baochun1 agatctcagaatttatgcatcttccataaaggagccgaatgaaaccaaagtttcatgttc

************************************************************

baochun2 ggttttgaattagagacgttaaaaatgttgaattgaatcgacgtcgactataacccctag

baochun1 ggttttgaattagagacgttaaaaatgttgaattgaatcgacgtcgactataacccctag

************************************************************

baochun2 ccttccaagctaacgatgcgggttcgattcccgctacccgctttagaccctctcttatcg

baochun1 ccttccaagctaacgatgcgggttcgattcccgctacccgctttagaccctctcttatcg

************************************************************

baochun2 aatttatagattaattcatatattcattctttatatttctttcttaattaatattaatag

baochun1 aatttatagattaattcatatattcattctttatatttctttcttaattaatattaatag

************************************************************

baochun2 gaagaaatataaagaatgaatatataaaactcttacttatatattcgctattttcattct

baochun1 gaagaaatataaagaatgaatatataaaactcttacttatatattcgctattttcattct

************************************************************

baochun2 ctctatattaattaataattataattaatgcatgattgaattaaatatacaattcctaaa

baochun1 ctctatattaatta------ataattaatgcatgattgaattaaatatacaattcctaaa

************** ****************************************

baochun2 aaaatctcacatacaatccaacaatcctatttttttcaaacaagaagcaaaataaaaatg

baochun1 aaaatatcacatacaatccgacaatcctatttttttcaaacaagaagcaaaataaaaatg

***** *************.****************************************

baochun2 aatgaaaagcgtccattgtctaatggataggacagaggtcttctaaacctttagtatagg

baochun1 aatgaaaagcgtccattgtctaatggataggacagaggtcttctaaacctttagtatagg

************************************************************

baochun2 ttcaaatcctattggacgcaatgtatttccatctatttttttctaaaaaaaataatcaat

baochun1 ttcaaatcctattggacgcaatgtatttccatctatttttttctaaaaaaaataatcaat

************************************************************

baochun2 ttctttatgcttgttcctgaagtataaagcgttccatctgttcctgaatagcttctttca

baochun1 ttctttatgcttgttcctgaagtataaagcgttccatctgttcctgaatagcttctttca

************************************************************

baochun2 aaagggcttccgcttctgcgttaaatgttttgctagaagatatgatttcttggaactggg

baochun1 aaagggcttccgcttctgcgttaaatgttttgctagaagatatgatttcttggaactggg

************************************************************

baochun2 gtttattagttcttaagtaagtacgtaactcaacaagaaatttccttacctgtccaattt

baochun1 gtttattagttcttaagtaagtacgtaactcaacaagaaatttccttacctgtccaattt

************************************************************

baochun2 ctaatgaatcaagataaccatttgttccggtataaatagtcattatctgttcttccaccg

baochun1 ctaatgaatcaagataaccatttgttccggtataaatagtcattatctgttcttccaccg

************************************************************

baochun2 taagaggagctgattgggattgtttgagtaattcacgtaatcgttgacctcttgccaatt

baochun1 taagaggagctgattgggattgtttgagtaattcacgtaatcgttgacctcttgccaatt

************************************************************

baochun2 gattctgagtagctttatcaagatcagaagcgaattgtgcaaaggcttctaattctgcaa

baochun1 gattctgagtagctttatcaagatcagaagcgaattgtgcaaaggcttctaattctgcaa

************************************************************

baochun2 attgtgctaattccaattttaatttaccggctacttgtttcatagctttaatctgagctg

baochun1 attgtgctaattccaattttaatttaccggctacttgtttcatagctttaatctgagctg

************************************************************

baochun2 ccgatcctactctggaaacggaaatacccacattaatagcaggtctaattccagaattaa

baochun1 ccgatcctactctggaaacggaaatacccacattaatagcaggtctaattccagaattaa

************************************************************

baochun2 atagatctgcggataagaatatttgtccatcagtaatggaaattacattagtaggaatat

baochun1 atagatccgcggataagaatatttgtccatcagtaatggaaattacattagtaggaatat

*******.****************************************************

baochun2 aagctgaaacatctcctgattgagtctcaactattggtaaagcggtcatacttccttcac

baochun1 aagctgaaacatctcctgattgagtctcaactattggtaaagcggtcatacttccttcac

************************************************************

baochun2 ctaaactagaacttgatttagcggctctttctaaaaggcgtgaatgcaaataaaaaacat

baochun1 ctaaactagaacttgatttagcggctctttctaaaaggcgtgaatgcaaataaaaaacat

************************************************************

baochun2 cccctggataagcttcgcgacctggtggtcttcgtaatagaagagacatttggcgatatg

baochun1 cccctggataagcttcgcgacctggtggtcttcgtaatagaagagacatttggcgatatg

************************************************************

baochun2 cttgggcttgtttggagagatcatcataaatgattaaagtatgtcgttgacggtacataa

baochun1 cttgggcttgtttggagagatcatcataaatgattaaagtatgtcgttcacggtacataa

************************************************ ***********

baochun2 aatattcagctaaggctgctccggtataaggagcaagatattgtaatgtagccggtgaat

baochun1 aatattcagctaaggctgctccggtataaggagcaagatattgtaatgtagccggtgaat

************************************************************

baochun2 ccgccgtttctgctactacaatagtgtattccattgctcccctttcctggaaattagtaa

baochun1 ccgccgtttctgctactacaatagtgtattccattgctcccctttcctggaaattagtaa

************************************************************

baochun2 ctacctgagccacagaagatgctttttgaccaatagctacataaacacatattacatttt

baochun1 ctacctgagccacagaagatgctttttgaccaatagctacataaacacatattacatttt

************************************************************

baochun2 ggccctgttgattgagaatcgtatctgtggctactgctgttttgccagtctgtctgtccc

baochun1 ggccctgttgattgagaatcgtatctgtggctactgctgttttgccagtctgtctgtccc

************************************************************

baochun2 cgataattaattctcgttgaccacgtcctattggaatcatcgcatcaatagcgataagtc

baochun1 cgataattaattctcgttgaccacgtcctattggaatcatcgcatcaatagcgataagtc

************************************************************

baochun2 ccgtttgaagaggctcatatacggaacgtcgagaaataatacccggggcgggagattcaa

baochun1 ccgtttgaagaggctcatatacggaacgtcgagaaataatacccggggcgggagattcaa

************************************************************

baochun2 ttaaccgagattcagaagctgaaatttcacctctaccatcaataggtttagccaatgcat

baochun1 ttaaccgagattcagaagctgaaatttcacctctaccatcaataggtttagccaatgcat

************************************************************

baochun2 ttataacacgacccaaataggcctcactcacgggtatctgggcaatttttcctgttgctt

baochun1 ttataacacgacccaaataggcctcactcacgggtatctgggcaatttttcctgttgctt

************************************************************

baochun2 ttacagagcttccttcttgtatcagcaaaccgtcacccattaatacaacaccaacattat

baochun1 ttacagagcttccttcttgtatcagcaaaccgtcacccattaatacaacaccaacattat

************************************************************

baochun2 ttgattccaaattcagagcaatgcctattgtaccttcttcaaattctactaactcgcctg

baochun1 ttgattccaaattcagagcaatgcctattgtaccttcttcaaattctactaactcgcctg

************************************************************

baochun2 ccattacttcatcaagaccatgaatacgagcaataccgtcacctacttgaaggacggtac

baochun1 ccattacttcatcaagaccatgaatacgagcaataccgtcacctacttgaaggacggtac

************************************************************

baochun2 cggtatttataatctttacttctctgttatattgctcaatgcgttcacggataatattac

baochun1 cggtatttacaatctttacttctctgttatattgctcaatgcgttcacggataatattac

*********.**************************************************

baochun2 taatttcgtcggctcgaatagttgccatgagtatttcttaattcgtttttg-aaaaaaaa

baochun1 taatttcgtcggctcgaatagttgccatgagtatttcttaattcgtttttgaaaaaaaaa

*************************************************** ********

baochun2 aataatgcctgcagtagaatgactaatcaattatttctttcatcgccccaaacataccaa

baochun1 aataatgcctgcagtagaatgactaatcaattatttctttcatcgccccaaacataccaa

************************************************************

baochun2 tattggcactgatggtacgtaaatgtaattcgttgttcaaacaaccagtcagagttccta

baochun1 tattggcactgatggtacgtaaatgtaattcgttgttcaaacaaccagtcagagttccta

************************************************************

baochun2 aagcgccctctaaggcttgttggaaaacctgttgtcgaacttcattaattgcgctttgtt

baochun1 aagcgccctctaaggcttgttggaaaacctgttgtcgaacttcattaattgcgctttgtt

************************************************************

baochun2 gttcaaaatgaatggtttcatttttgtaattttccaattgttccaaagtcttataagttg

baochun1 gttcaaaatgaatggtttcatttttgtaattttccaattgttccaaagtcttataagttg

************************************************************

baochun2 aattaatcaaattcaatttttctcgctctatctcagaatacccatttactcgaaactcat

baochun1 aattaatcaaattccatttttctcgctctatctcagaatacccatttactcgaaactcat

************** *********************************************

baochun2 ctgcttccatttctactttccgtaagcgagcccgggctttttctagctgttcaatggctc

baochun1 ctgcttccatttctactttccgtaagcgagcccgggctttttctagctgttcaatggctc

************************************************************

baochun2 cctcacgcagttcttctgaatttcgaatagtattcaaaatcttctgttttcgattatcta

baochun1 cctcacgcagttcttctgaatttcgaatagtattcaaaatcttctgttttcgattatcta

************************************************************

baochun2 ataaatcgcttaatgaaagtagattatctttccattcatttcaaaacttccatgatccct

baochun1 ataaatcgcttaatgaaagtagattatctttccattcatttcaaaacttccatgatccct

************************************************************

baochun2 tcccaaaccaaacatgaatctttcaattcatttggctctcacgctcaattacttttggga

baochun1 tcccaaaccaaacatgaatctttcaattcatttggctctcacgctcaattacttttggga

************************************************************

baochun2 aatgcccctatcttttttaatgtaatgagcctatcttctcttctctggtcatattccaaa

baochun1 aatgcccctatcttttttaatgtaatgagcctatcttctcttctctggtcatattccaaa

************************************************************

baochun2 agaaaatcaaaatatcaaaacggattactaatccaagactaaaatattcggaggactctt

baochun1 agaaaatcaaaatatcaaaacggattactaatccaagactaaaatattcggaggactctt

************************************************************

baochun2 ctgaccaaataaaaaagatgtaattgtcagcaaagctgtttcttttttctttaaattcaa

baochun1 ctgaccaaataaaaaatatgtaattgtcagcaaagctgtttcttttttctttaaatccaa

**************** ***************************************.***

baochun2 aaatgcttcttactttatatataaaaatatataaaac-taatacataggttgtcgattca

baochun1 aaatgcttcttactttatatataaaaatatataaaacataatacataggttgtcgattca

************************************* **********************

baochun2 gcattggatgaaaaaaaggacgaaataccacccatttttccaataaatggttcaaatttt

baochun1 gcattggatgaaaaaaaggacgaaataccacccatttttccaataaatggttcaaatttt

************************************************************

baochun2 tttatcaatatgggtgttctatatcgaaaaaaatgtagtttgaaaccatctctatattaa

baochun1 tttatcaatatgggtgttctatatcgaaaaaaatgtagtttgaaaccatctctatattaa

************************************************************

baochun2 catagtggtagaaagagtaccatgctgcgtctggacttcaaacggtttagctttaaccat

baochun1 catagtggtagaaagagtaccatgctgcgtctggacttcaaacggtttagctttaaccat

************************************************************

baochun2 gttaacagtcctacattcttggttcatagagaatcaaagcggatttaccaatgaatcacg

baochun1 gttaacagtcctacattcttggttcatagagaatcaaagcggatttaccaatgaatcacg

************************************************************

baochun2 aaatgctacggttcttgtatataatttcttaatttattcccaagttattcgctagattat

baochun1 aaatgctatggttcttgtatataatttcttaatttattcccaagttattcgctagattat

********.***************************************************

baochun2 gcacctcttactttcctaattataagggcaaaaagtgcagctggttagatccagcctatt

baochun1 gcaccccttactttcctaattataagggcaaaaagtgcagctggttagatccagcctatt

*****.******************************************************

baochun2 cttgaaataaacaactcgcacacaccccctttccaaaaaagatcaatacaccaagtacta

baochun1 cttgaaataaacaactcgcacacaccccctttccaaaaaagatcaatacaccaagtacta

************************************************************

baochun2 cagttagatttattggatttgttgctaaaatatcggtattaaatccgaaactcccggcga

baochun1 cagttagatttattggatttgttgctaaaatatcggtattaaatccgaaactcccggcga

************************************************************

baochun2 atggccagtagcccaaagaaacgaaagaatcggttacatttttcatatgctctcctctta

baochun1 atggccagtagcccaaagaaacgaaagaatcggttacatttttcatatgctctcctctta

************************************************************

baochun2 tagatagactaacaaatcgaacagagttcttttttcatcacttcacccatttttctttta

baochun1 tagatagactaacaaatcgaacagagttcttttttcatcacttcacccatttttctttta

************************************************************

baochun2 tatatatttctaaattcgcaatttaaagtagaataaaaaatatatttgatttcaaaattt

baochun1 tatatatttctaaattcgcaatttaaagtagaataaaaaatatatttgatttcaaaattt

************************************************************

baochun2 ttaactacttggtttgttgcaacacttcctctttctcttgaactacgaatgggaaggaca

baochun1 ttaactacttagtttgttgcaacacttcctctttctcttgaactacgaatgggaaggaca

**********.*************************************************

baochun2 aaagagaatcggtatgctaattcctcatcctcaaatcagtccttcccatcccacggatta

baochun1 aaagagaatcggtatgctaattcctcatcctcaaatcagtccttcccatcccacggatta

************************************************************

baochun2 ttttcttaacgaaaacgaataaataaaatcattttaaattataagagttaaattttgata

baochun1 ttttcttaacgaaaacgaataaataaaatcattttaaattataagagttaaattttgata

************************************************************

baochun2 taataaaaaaaacaaaggacaagtccaaagcaataaaatatgaaaaaaaataggtatttt

baochun1 taataaaaaaaacaaaggacaagtccaaagcaataaaatatgaaaaaaaataggtatttt

************************************************************

baochun2 tcatatttctaggattaaacaaaaggatttgcaaataaaagtgctaatgctacaactagc

baochun1 tcatatttctaggattaaacaaaaggatttgcaaataaaagtgctaatgctacaactagc

************************************************************

baochun2 ccataaattgttaaagcttccatgaaagctaaactaagcaataaggtacctcgtattttt

baochun1 ccataaattgttaaagcttccatgaaagctaaactaagcaataaagtacctcgtattttt

********************************************.***************

baochun2 ccctccgcctcgggctgtctcgcgataccttcgacagcttggcccgcagcagtaccttga

baochun1 ccctccgcctcgggctgtctcgcgataccttcgacagcttggcccgcagcagtaccttga

************************************************************

baochun2 ccaactccgggtccaatagaagcaagccctacggccaatccagcagcaataacggaagcg

baochun1 ccaactccgggtccaatagaagcaagccctacggccaatccagcagcaataacggaagcg

************************************************************

baochun2 gcagaaaccagtggattcatgataagttcctcgcaccaaaaaaaagaaatggttaatgat

baochun1 gcagaaaccagtggattcatgataagttcctcgcaccaaaaaaaagaaatggttaatgat

************************************************************

baochun2 acaatcaactaacgaattatgacttaattattccagcactaaatgcatctagtcgaagta

baochun1 acaatcaactaacgaattatgacttaattattccagcactaaatgcatctagtcgaagta

************************************************************

baochun2 actaagaattgcgaattagagtcaaaaaatattattaaagcatcggaactacttcgatat

baochun1 actaagaattgcgaattagagtcaaaaaatattattaaagcatcggaactacttcgatat

************************************************************

baochun2 cgtctatccacagagtatttgtgaattcatatgactttttttcgttcttccatttcttga

baochun1 cgtctatccacagagtatttgtgaattcatatgactttttttcgttcttccatttcttga

************************************************************

baochun2 ttccgagcggcctgttcggcctttttattgatttcatctctttattaattcaactcaaag

baochun1 ttccgagcggcctgttcggcctttttattgatttcatctctttattaattcaactcaaag

************************************************************

baochun2 tctcaaacgaaacggaagaacgtctattaaaattctcatctaaatttaccatagtaagac

baochun1 tcccaaacgaaacggaagaacgtctattaaaattctcatctaaatttaccatagtaagac

**.*********************************************************

baochun2 aaatctcgagttcatataattagtcaatatctactatcacatatacatgtttttttccat

baochun1 aaatctcgagttcatataattagtcaatatctactatcacatatacatgtttttttccat

************************************************************

baochun2 aacgtaaaccaactattcctctcttagattcaatcggattctagaattgttcatcaaaac

baochun1 aacgtaaaccaactattcctctcttagattcaatcggattctagaattgttcatcaaaac

************************************************************

baochun2 atccaaaagagttagtttgtagccattaaaattatatagacctagttcaaccctttctct

baochun1 atccaaaagagttggtttgtagccattaaaattatatagacctagttcaaccctttctct

*************.**********************************************

baochun2 cctaaccaaaccctccctttctttatcattattccacatggataaaatggagattgggcc

baochun1 cctaaccaaaccctccctttctttatcattattccacatggataaaatggagattgggcc

************************************************************

baochun2 gaatcattgcatataaatatcattatttaattgatcgaagtttaggcaatttattattta

baochun1 gaatcattgcatataaatatcattatttaattgatctaagtttaggcaatttattattta

************************************ ***********************

baochun2 tttcatttttcttttggtcaatcgttggttttattaaattgaaggggaagtcgttctata

baochun1 ttacatttttcttttggtcaatcgttggttttattaaattgaaggggaagtcgttctata

** *********************************************************

baochun2 agaacatactttttttttcgaaaacgagtcaatgatggccctccatggattcacctatat

baochun1 agaacatac-ttttttttcgaaaacgagtcaatgatggccctccatggattcacctatat

********* **************************************************

baochun2 aagcagcggctaaagttgcaaaaataagagcttgaataccgctggtaaataatccaagga

baochun1 aagcagcggctaaagttgcaaaaataagagcttgaataccgctggtaaataatccaagga

************************************************************

baochun2 acatgacaggtataggaaccactaaaggtactaaagaaacaagcacaacaactactaatt

baochun1 acatgacaggtataggaaccactaaaggtactaaagaaacaagaacaacaactactaatt

******************************************* ****************

baochun2 catcggctaatatatttccaaaaagtcgaaaactaagcgataagggttttgtgaaatctt

baochun1 catcggctaatatatttccaaaaagtcgaaaactaagcgataagggttttgtgaaatctt

************************************************************

baochun2 ctaatatgttaatgggtaaaaggattggagtcggttgaatgtatttcccaaaataagaca

baochun1 ctaatatgttaatgggtaaaaggattggagtcggttgaatgtatttcccaaaataagaca

************************************************************

baochun2 atccttttttgctaagacccgcatagaaatatgccattgaggtgagtaaagccaaagcaa

baochun1 atccttttttgctaagacccgcatagaaatatgccattgaggtgagtaaagccaaagcaa

************************************************************

baochun2 cagtagtatttatatcattcgtaggcgcggctaactccccgtggggtaattcaattagtt

baochun1 cagtagtatttatatcattcgtaggcgcggctaactccccgtggggtaattcaattagtt

************************************************************

baochun2 tccaaggtaaaagagctccggaccaatttgaaacaaaaataaatagaaacatagttccaa

baochun1 tccaaggtaaaagagctccagaccaatttgaaacaaaaataaatagaaacatagttccaa

*******************.****************************************

baochun2 taaagggaacccaagggccgtattcttccccaatttgagttttactcacatcacgaatga

baochun1 taaagggaacccaagggccgtattcttccccaatttgagttttactcacatcacgaatga

************************************************************

baochun2 attcaagaacatattcgaagaaattctgaccgccagtcggaatggtttgtgggttccgaa

baochun1 attcaagaacatattcgaagaaattctgaccgccagtcggaatggtttgtgggttccgaa

************************************************************

baochun2 cagctaacgtggctgaacccaataagatagcaattacaacccaagaagtaataagtactt

baochun1 cagctaacgtggctgaacccaataagatagcaattacaacccaagaagtaataagtactt

************************************************************

baochun2 ggccgtggacttggaaacttcctattttccaatagaaatgttgacctacttctacaccgg

baochun1 ggccgtggacttggaaacttcctattttccaatagaaatgttgacctacttctacaccgg

************************************************************

baochun2 atatatcatataaaccctttagtgtgttgatggaacataatagaacattcatattgcccc

baochun1 atatatcatataaaccctttagtgtgttgatggaacataatagaacattcatattgcccc

************************************************************

baochun2 ctgacagaaatagaactttaaacaaaattattttgattcaaccatctcattcttgactta

baochun1 ctgacagaaatagaactttaaacaaaattattttgattcaaccatctcattcttgactta

************************************************************

baochun2 cctattttacttgaatcctatattttgaataccaattaattacataatatcctcagttat

baochun1 cctattttccttgaatcctatattttgaataccaattaattacataatatcctcagttat

******** ***************************************************

baochun2 tctctcttttttgaggttcacaaattgtaaccaattccataaatccttttcgtttcctaa

baochun1 tctctcttttttgaggttcacaaattgtaaccaattccataaatccttttagtttcctaa

************************************************** *********

baochun2 ctaatttatttatcttattattatcaaggattgattatatagctagaacgaccctcacaa

baochun1 ctaatttatttatcttattattatcaaggattgattatatagctagaacgaccctcacaa

************************************************************

baochun2 attgcaaatattaatttgttaagaattaatcggattgaagctatagcgtcatcgttcgct

baochun1 attgcaaatattaatttgttaagaattaatcggattgaagctatagcgtcatcgttcgct

************************************************************

baochun2 ggaatcgaaatatctccaagatcggggtcacaatttgtatcgattaaacaaattgttgga

baochun1 ggaatcgaaatatctccaagatcggggtcacaatttgtatcgattaaacaaattgttgga

************************************************************

baochun2 attcccaaagtaatacattctcgaagagctgtatattcttcgtgctgatcaacaatgatt

baochun1 attcccaaagtaatacattctcgaagagctgtatattcttcgtgctgatcaacaatgatt

************************************************************

baochun2 acaatatcaggtaaccccgtcatatatttaatcccgcccagatatgtttgcaagtgaaat

baochun1 acaatatcaggtaaccccgtcatatatttaatcccgcccagatatgtttgcaagtgagat

*********************************************************.**

baochun2 aattgtcttttcaacatagctgcatctcttttcggaagacgattaaatctgcccatcttt

baochun1 aattgtcttttcaacatagctgcatctcttttcggaagacgattaaatctgcccatcttt

************************************************************

baochun2 tgttccattctcaagtctctaaacttatgaagtctcgtttctgtagtagaccaattcgtt

baochun1 tgttccattctcaagtctctaaacttatgaagtctcgtttctgtagtagaccaattcgtt

************************************************************

baochun2 aacataccaccgagccattttttattaacataatgacatcgagcccttattgcagcccgt

baochun1 aacataccaccgagccattttttattaacataatgacatcgagcccttattgcagcccgt

************************************************************

baochun2 gctactgaatcagctgctttattttttgtaccaacaattaaaaactgttttcccctactt

baochun1 gctactgaatcagctgctttattttttgtaccaacaattaaaaactgttttcccctactt

************************************************************

baochun2 gctgcatcaaaaaccaaatcacaagcttctgataaaaaacgagcagttcgagtaagattt

baochun1 gctgcatcaaaaaccaaatcacaagcttctgataaaaaacgagcagttcgagtaagattt

************************************************************

baochun2 ataatatgaatacctttacgctttgcagaaatataaggtgccattctaggattccatttc

baochun1 ataatatgaatacctttacgctttgcagaaatataaggtgccattctaggattccatttc

************************************************************

baochun2 ctagtaccatggccaaaatgaactcctgcctccatcatctcttccaaattgatgttccaa

baochun1 ctagtaccatggccaaaatgaactcctgcctccatcatctcttccaaattgatgttccaa

************************************************************

baochun2 tatcttcttgtcacgtttccccccactccccctccctttttcacaaaaaaaggggcgagg

baochun1 tatcttcttgtcacgtttccccccactccccctccctttttcacaaaaaaaggggcgagg

************************************************************

baochun2 tgccctgaaaaataaataattgttccgacggaaccttttcttttaccgtaaattaaatta

baochun1 tgccctgaaaaataaataattgttccgacggaaccttttcttttaccgtaaattaaattg

***********************************************************.

baochun2 gacgtagatatacgatccaagctattattcttttctattagttattttcgttattactat

baochun1 gacgtagatatacgatccaagctattattcttttctattagttattttcgttattactat

************************************************************

baochun2 gtattattaccaaattaaacggaccaaatatagataatgaatcaactcttaggcatcttt

baochun1 gtattattaccaaattaaacggaccaaatatagataatgaatcaactcttaggcatcttt

************************************************************

baochun2 taatcctataaatgattgttctgatgaatcgtgaaaattctttgaaatacaagaatcaaa

baochun1 taatcctataaatgattgttctgatgaatcgtgaaaattctttgaaatacaagaatcaaa

************************************************************

baochun2 taattttctgtggtggaacaaaatatctctcatttctccctcaaatagattcttc-tttt

baochun1 taattttctgtggtggaacaaaatatctctcatttctccctcaaatagattcttcttttt

******************************************************* ****

baochun2 tttttccaaaggaatgttattatgttgacttgaaagggacattaatcctttgaaaccggt

baochun1 tttttccaaaggaatgttattatgttgacttgaaagggacattaatcctttgaaaccggt

************************************************************

baochun2 accgacgggaatcattcctcccagaactacattttcttttaagccctttaaccaatcgat

baochun1 accgacgggaatcattcctcccagaactacattttcttttaagccctttaaccaatcgat

************************************************************

baochun2 acgaccccggagagccgcttttgctaaaactcgagcagtttcttgaaaactcgcttcaga

baochun1 acgaccccggagagccgcttttgctaaaactcgagcagtttcttgaaaactcgcttcaga

************************************************************

baochun2 tatgaaactttgagtattcagcgatgctcttgttattcccaataagatagctcggtaaca

baochun1 tatgaaactttgagtattcagcgatgctcttgttattcccaataagatagctcggtaaca

************************************************************

baochun2 aatcgcttcttctaaagcacgccccattcgttccgctctcaacaatccaattagctctcc

baochun1 gatcgcttcttctaaagcacgccccattcgttccgctctcaacaatccaattagctctcc

.***********************************************************

baochun2 gggtgaaaaaacattagacattccatcttctgaaaccagcacttttgatgttatttgacg

baochun1 gggtgaaaaaacattagacattccatcttctgaaaccagcacttttgatgttatttgacg

************************************************************

baochun2 tacaataatttcgatatgcctattatggatttgtaccccctgggatcgataaaccttttg

baochun1 tacaataatttcgatatgcctattatggatttgcaccccctgggatcgataaaccttttg

*********************************.**************************

baochun2 gatcttattaactaaagagatacgactttgcactatagttagctccgcgccaatcaagaa

baochun1 gatcttattaactaaagagatacgactttgcactatagttagctccgcgccaatcaagaa

************************************************************

baochun2 tccccaaggaatgccaagaattcttgttatagattcgttccaaccctcaactctcttttc

baochun1 tccccaaggaatgccaagaattcttgttatagattcgttccaaccctcaactctcttttc

************************************************************

baochun2 tagattcattgatattgactcaattgaacgcacttctaacacctgttcgacttttggaag

baochun1 tagattcattgatattgactcaattgaacgcacttctaacacctgttcgacttttggaag

************************************************************

baochun2 accctgcgttatatcaccagatctcgatttttcatatataaatgtaactaacgtatctcc

baochun1 accctgcgttatatcaccagatctcgatttttcatatataaatgtaactaacgtatctcc

************************************************************

baochun2 ttcataaaagatttcgccataatggccacgaactgttgctcctggagtggccaaataggg

baochun1 ttcataaaagatttcgccataatggccacgaactgttgctcctggagtggccaaataggg

************************************************************

baochun2 cttagccgagcgtattactacagagtcaacttgaacaattagaacttgacccgattttag

baochun1 cttagccgagcgtattactacagagtcaacttgaacaattagaacttgacccgattttag

************************************************************

baochun2 gtgtggttcatttttatctatacatgaattttcacaaataaactgtccaagacttatttt

baochun1 gtgtggttcatttttatctatacatgaattttcacaaataaactgtccaagacttatttt

************************************************************

baochun2 tgtagatgccttttcacaataattgtgatggagaaaataccaattcaaatttaatggatt

baochun1 tgtagatgccttttcacaataattgtgatggagaaaataccaattcaaatttaatggatt

************************************************************

baochun2 caaaagggtgttactacatggatcgggattataaattttcctattttcatccattaaata

baochun1 caaaagggtgttactacatggatcgggattataaattttcctattttcatccattaaata

************************************************************

baochun2 atatttaagtacttgaaaagtctgtttaaaattgtccacttgtaaatatttagttagggg

baochun1 atatttaagtacttgaaaagtctgtttaaaattgtccacttgtaaatatttagttagggg

************************************************************

baochun2 gatctgattataagttatcaaacaggaaaatgcataaaaattcgaaattttaaaagctat

baochun1 gatctgattataagttatcaaacaggaaaatgcataaaaattcgaaattttaaaagctat

************************************************************

baochun2 tcctaaagggcccaccgaatttcgaattggcattagggaattttttttaattgattcttt

baochun1 tcctaaagggcccaccgaatttcgaattggcattagggaattttttttaattg-------

*****************************************************

baochun2 tatcacattgaattcttttatcacattgaatgattttacatcattgaacggacccattcg

baochun1 -----------attcttttatcacattgaatgattttacatcattgaacggacccattcg

*************************************************

baochun2 aaaacagttggatgatgacaagattatcaaaaattggcaatccttatttctattcaataa

baochun1 aaaacagttggatgatgacaagattatcaaagattggcaatccttatttctattcaataa

*******************************.****************************

baochun2 cgtatgaatagttccgtgattttggttaagggattgttgaatctgtgtcctggaatacaa

baochun1 cgtatgaatagttccgtgattttggttaagggattgttgaatctgtgtcctggaatacaa

************************************************************

baochun2 aaaagtggaagaaaatggatttatagtggtgcgatctgattcattatcagggatcaatcc

baochun1 aaaaatggaagaaaatggatttatagtggtgcgatctgattcattatcagggatcaatcc

****.*******************************************************

baochun2 agaatctgaaaggtcatttctttttctagtatacgaaatagaggatttgactaagtcaat

baochun1 agaatctgaaaggtcatttctttttctagtatacgaaatagaggatttgactaagtcaat

************************************************************

baochun2 tcttagaaaatctcgcatcaaaccatttgtccttatttcaacaaaggaagcgcgggcctc

baochun1 tcttagaaaatctcgcatcaaaccatttgtccttatttcaacaaaggaagcgcgggcctc

************************************************************

baochun2 ttcgatagaagaaatttttttgtcttgctcccaattcaatactaaacaagtccgaactaa

baochun1 ttcgatagaagaaatttttttgtcttgctcccaattcaatactaaacaagtccgaactaa

************************************************************

baochun2 ttgaatacacatgtccgaaattccccgaatgggtttgtcgtttccataaagaatataatt

baochun1 ttgaatactcgtgtccgaaattccccgaatgggtttgccgtttccataaagaatataatt

******** *.**************************.**********************

baochun2 gataacttgaagttgcaaattatccctttcctgcaatagatctaggggaaaaagtctttc

baochun1 gataacttgaagttgcaaattatccctttcctgcaatagatctaggggaaaaagtctttc

************************************************************

baochun2 taaatttataccgtccgttatttcatatgtgattacaggccgaaccaaaacaaaagactt

baochun1 taaatttataccgtccgttatttcatatgtgattacaggccgaaccaaaacaaaagactt

************************************************************

baochun2 tttcttggtcggtgtgatccgttgtacatagatccaatttttcaattttttggatgcttt

baochun1 tttcttggtcggtgtgatccgttgtacatagatccaatttttcaattttttggatgcttt

************************************************************

baochun2 gtcatttttttttcctattcttggaggtattaagatgccactgtgtcgagatattttatc

baochun1 gtcatttttttttcctattcttggaggtattaagatgccactgtgtcgagatattttatc

************************************************************

baochun2 tatatctccgggaaaatgtatatctccagaaaagattttcagttcaattctttttttttt

baochun1 tatatctccgggaaaatgtatatctccagaaaagattttcagttcaattctttttttttt

************************************************************

baochun2 tctttccactcggattaacccgcctactctacttcttgtatttaaagtgatttgtgtatc

baochun1 tctttccactcggattaacccgcctactctacttcttgtatttaaagtgatttgtgtatc

************************************************************

baochun2 taccccaatgatactgttattccgtaccattatggtagaagatccaggtaagaaatacac

baochun1 taccccaatgatactgttattccgtaccattatggtagaagatccaggtaagaaatacac

************************************************************

baochun2 ttcttcaggaatgaaaaaaaaccaatttactttcatttgcatttggtattttggcctaaa

baochun1 ttcttcaggaatgaaaaaaaaccaatttactttcatttgcatttggtattttggcctaaa

************************************************************

baochun2 ttctttgactcctcgatactcaatcaaatcctcttttttgaccattgaatgcccttctag

baochun1 ttctttgactcctcgatactcaatcaaatcctcttttttgaccattgaatgcccttctag

************************************************************

baochun2 agtcccatatttagtaattcccgaactctttctgctatatcgagaatcatcgaaataagc

baochun1 agtcccatatttagtaattcccgaactctttctgctatatcgagaatcatcgaaataagc

************************************************************

baochun2 aagaatactatttctacggaaaataccatttataggtatttcaatcaaaatacctgaagg

baochun1 aagaatactatttctacggaaaataccatttataggtatttcaatcaaaatacctgaagg

************************************************************

baochun2 gtagatccgttctttctctcgttcttgatgaatccattggagtggaatggtgaatcgatt

baochun1 gtagatccgttctttctctcgttcttgatgaatccattggagtagaatggtgaatcgatt

*******************************************.****************

baochun2 tcttcgcctctttgccaataaatccgaattctcgtggagaatggcaagatctataagatt

baochun1 tcttcgcctctttgccaataaatccgaattctcgtggagaatggcacgatctataagatt

********************************************** *************

baochun2 cgaatcaccaacgcatatgattcgattcaagtctgaatgatcaagactcctatcttcttt

baochun1 cgaatcaccaatgcatatgattcgattcaagtctgaatgatcaagactcctatcttcttt

***********.************************************************

baochun2 tttatcaaaaaaacccgaactaactaatttatgtctcacttgatcattagtcaatgaggg

baochun1 tttatcaaaaaaacccgaactaactaatttatgtctcacttgatcattagtcaatgaggg

************************************************************

baochun2 attagaaatagatcctcgctttccagtgatagaaatagaatgggcgctcgtttgatcttg

baochun1 attagaaatagatcctcgctttccagtgacagaaagagaatgggcgctcgtttgatcttg

*****************************.***** ************************

baochun2 atccttgtggaacgaaaaggaggctagactggatctgcacggccctccggataatatcca

baochun1 atccttgtggaacgaaaaggaggctagactggatctgcacggccctccggataatatcca

************************************************************

baochun2 taaatgacttgtttttggtaagagatgaacattaccatatgtaaattcaggtgcatgata

baochun1 taaatgacttgtttttggtaagagatgaacattaccatatgtaaattcaggtgcatgata

************************************************************

baochun2 cacatcagtactccaatgcatttctccctctgaatcagaataaatatgttttctaactct

baochun1 cacatcagtactccaatgcatttctccctctgaatcagaataaatatgttttttaactct

****************************************************.*******

baochun2 ctctttaaaattaaaagtggctgttcccgcgcgaatctcagcaattacttgttctgattc

baochun1 ctctttaaaattaaaagtggctgttcccgcgcgaatctcagcaattacttgttctgattc

************************************************************

baochun2 tacatattgatcattttgaactaacagaaaactttttgatggaatagtcacattatgaag

baochun1 tacatattgatcattttgaactaacagaaaactttttgatggaatagtcacattatgaag

************************************************************

baochun2 aatatcttcactttcaatagttacatacaaatctatagaacatagaaaagcgggatgccc

baochun1 aatatcttcactttcaatagttacatacaaatctatagaacatagaaaagcgggatgccc

************************************************************

baochun2 atgacgtgtacgtgttggatgaaccaaattttcattaaattgaatttttccattagaggg

baochun1 atgacgtgtacgtgttggatgaaccaaattttcattaaatttaatttttccattagaggg

***************************************** ******************

baochun2 ggctcgtacatgttctgcagtgcctcctgtgaataccccgccggtatgaaaagttcttaa

baochun1 ggctcgtacatgttctgcagtgcctcctgtgaataccccgccggtatgaaaagttcttaa

************************************************************

baochun2 tgttaattgagtaccgggttctccaatagattgacctgcaataatacctacggcttctcc

baochun1 tgttaattgagtaccgggttctccaatagattgacctgcaataatacctacggcttctcc

************************************************************

baochun2 caattcgaccaggtcaccatgagtcggactgcgtccataacataatcgacagatccaaga

baochun1 caattcgaccaggtcaccatgagtcggactgcgtccataacataatcgacagatccaaga

************************************************************

baochun2 tgtactcctacaagtaaagggggttcgaatatatattggttgtgttcgaaaggttatgaa

baochun1 tgtactcctacaagtaaagggggttcgaatatatattggttgtgttcgaaaggttatgaa

************************************************************

baochun2 tcgattaataagtgcaatcccaatatcttgatttcgagtggcaatgcatcgtgaacccat

baochun1 tcgattaataagtgcaatcccaatatcttgatttcgagtggcaatgcatcgtgaacccat

************************************************************

baochun2 atagatatcatctgctaatacacgaccaattaatgtttggataaaaacgcgttccggcat

baochun1 atagatatcatctgctaatacacgaccaattaatgtttggataaaaacgcgttccggcat

************************************************************

baochun2 catcccattttgaggactcacaaaacaacctcgtatggtgccacaatctgttcgacgcac

baochun1 catcccattttgaggactcacaaaacaacctcgtatggtgccacaatctgttcgacgcac

************************************************************

baochun2 aacaatgtgttgaactacttcaacaagtctccgcgtgagatatccagcatctgacgttcg

baochun1 aacaatgtgttgaactacttcaacaagtctccgcgtgagatatccagcatctgacgttcg

************************************************************

baochun2 tacagcagtatctacaactcctttgcgggctccatagcaagaaattatatattctgttaa

baochun1 tacagcagtatctacaactcctttgcgggctccatagcaagaaattatatattctgttaa

************************************************************

baochun2 agaaagtccttcgcgtaaattgctttgaatgggtaaatcaatcatttgtccttggggatc

baochun1 agaaagtccttcgcgtaaattgctttgaatgggtaaatcaatcatttgtccttggggatc

************************************************************

baochun2 cgacattaatcctctcatacctactaattgatgtacctgagatgcatttcctctagctcc

baochun1 cgacattaatcctctcatacctactaattgatgtacctgagatgcatttcctctagctcc

************************************************************

baochun2 cgaaaaagacattatatgaactggattaaaagggtcagtcattctaaaattaggattcat

baochun1 cgaaaaagacattatatgaactggattaaaagggtcagtcattctaaaattaggattcat

************************************************************

baochun2 ttcttgtcgcaaatattcacttgtagcataccatatctcaatggattgacgtaatttttc

baochun1 ttcttgtcgcaaatattcacttgtagcataccatatctcaatggattgacgtaatttttc

************************************************************

baochun2 gaccgcgtgtacattcccataacggtagtgtttttccaaaagaaaactttgttgttcagc

baochun1 gaccgcgtgtacattcccataacggtagtgtttttccaaaagaaaactttgttgttcagc

************************************************************

baochun2 atcttgaactagccatcctttcgaaggtattgttaaaagatcatcaattcctaatgaaat

baochun1 atcttgaactagccatcctttcgaaggtattgttaaaagatcatcaattcctaatgaaat

************************************************************

baochun2 agatgtagtagtggcttgctggaaacctagggtttttacttgatccaagatgtgtgatgt

baochun1 agatgtagtagtggcttgctggaaacctagggtttttacttgatccaagatgtgtgatgt

************************************************************

baochun2 atatgccattccgaagtgatctattaatcgactaataagtcgtttcatggcagttccgtc

baochun1 atatgccattccgaagtgatctattaatcgactaataagtcgtttcatggcagttccgtc

************************************************************

baochun2 gatcgccttattgtgaaagaccagattggcccgttctgccataagtacctccatatttca

baochun1 gatcgccttattgtgaaagaccagattggcccgttctgccataagtacctccatatttca

************************************************************

baochun2 ctgagtaggattcgacaatgggtttgagtcagtgattgaaaaactcccttttctcgatct

baochun1 ctgagtaggattcgacaatgggtttgagtcagtgattgaaaaactcccttttctcgatct

************************************************************

baochun2 tgattcgcgtaaaaattttgtaactataatcctagttgagccggagagacaaaaatttcc

baochun1 tgattcgcgtaaaaattttgtaactataatcctagttgagccggagagacaaaaatttcc

************************************************************

baochun2 gcaggtctcatagaattggtaccttctgattaggtactatatgagcaagcctgacaaaac

baochun1 acaggtctcatagaattggtaccttctgattaggtactatatgagcaagcctgacaaaac

.***********************************************************

baochun2 ccttgtatagcttcttcgatttctctataaagagaaatatgaccaacagtagttcgaata

baochun1 ccttgtatagcttcttcgatttctctataaagagaaatatgaccaacagtagttcgaata

************************************************************

baochun2 tatgtataaagaatttctttttttatacttcgtactattagatagtgcccataaatctca

baochun1 tatgtataaagaatttctttttttatacttcgtactattagatagtgcccataaatctca

************************************************************

baochun2 taataagtacccaaagattcatagtgaacctcgacgggagcttctcttgaagaaataacg

baochun1 taataagtacccaaagattcatagtgaacctcgacgggagcttctcttgaagaaataacg

************************************************************

baochun2 cgttgatcgagtcgccaccggagccagagaggactatctaaatggattcgtttctgtcga

baochun1 cgttgatcgagtcgccaccggagccagagaggactatctaaatggattcgtttctgtcga

************************************************************

baochun2 taagctccaattgcctcataagaattacaaaaaaggggttctttttcttttggatatttc

baochun1 taagctccaattgcctcataagaattacaaaaaaggggttctttttcttttggatatttc

************************************************************

baochun2 caattattgttgttaattctttccgttttagagtttctgcaattatatggattataccga

baochun1 caattattgttgttaattctttccgttttagagtttctgcaattatatggattataccga

************************************************************

baochun2 tttacagaaataccttgccgagtcccactcgttaatatatagagtcccataagcatatct

baochun1 tttacagaaataccttgccgagtcccactcgttaatatatagagtcccataagcatatct

************************************************************

baochun2 tgagttggtacggaaatgggatcaccaatagccggagacaaaagattaatatgagaaaac

baochun1 tgagttggtacggaaatgggatcaccaatagccggagacaaaagattaatatgagaaaac

************************************************************

baochun2 ataagtaaacgcgcctctgcttgagcctccaaagataaaggtacatgaacagccatttga

baochun1 ataagtaaacgcgcctctgcttgagcctccaaagataaaggtacatgaacagccatttga

************************************************************

baochun2 tccccgtcaaagtctgcattgaatcccttacaaactaatggatgtaaacaaatagcacgt

baochun1 tccccgtcaaagtctgcattgaatcccttacaaactaatggatgtaaacaaatagcacgt

************************************************************

baochun2 ccttccactaaaatgggctggaatgcctgtatgcccaatctatgcagagtaggtgctcta

baochun1 ccttccactaaaatgggctggaatgcctgtatgcccaatctatgcagagtaggtgctcta

************************************************************

baochun2 tttaacaaaacaggatgcccctgcataacttcttgaagtatttgccatacaaccggctct

baochun1 tttaacaaaacaggatgcccctgcataacttcctgaagtatttgccatacaaccggctct

********************************.***************************

baochun2 ttttcccgaatttgactcttcgcaacccctatattggaagcaagatgttgtctaattaga

baochun1 ttttcccgaattttactcttcgcaacccctatattggaagcaagatgttgtctaattaga

************* **********************************************

baochun2 ccccgaattacaaatgtctggaaaagctctattgctatttcgcgaggcaatccacatcga

baochun1 ccccgaattacaaatgtctggaaaagctctattgctatttcgcgaggcaatccacatcga

************************************************************

baochun2 tgtaatgaaagggaaggacccacgacaatgacggaacgccccgaataatcgacccgttta

baochun1 tgtaatgaaagggaaggacccacgacaatgacggaacgccctgaataatcgacccgttta

*****************************************.******************

baochun2 ccaagtagagtctcacgaaatctcccctctttgccttcaattacatctgaaaatgacttg

baochun1 ccaagtagagtctcacgaaatctcccctctttgccttcaattacatctgaaaatgacttg

************************************************************

baochun2 taaactttattatgaccgtccctcattggttgtccgcggattccattatccagaagtgta

baochun1 taaactttattatgaccgtccctcattggttgtccgcggattccattatccagaagtgta

************************************************************

baochun2 tccacggcttcttgtaccaatttctcctgacacattactaattctccaggcgtagatcgg

baochun1 tccacggcttcttgtaccaatttctcctgacacattactaattctccaggcgtagatcgg

************************************************************

baochun2 cttgttgttaatagatcggtaagagtgttgttacgatagataactcttctatagagttca

baochun1 cttgttgttaatagatcggtaagagtgttgttacgatagataactcttctatagagttca

************************************************************

baochun2 ttaatatccgagctcattagtttacccccctcgatctgaatgatcggtctcaactcggga

baochun1 ttaatatccgagctcattagtttacccccctcgatctgaatgatcggtctcaactcggga

************************************************************

baochun2 ggaagaactggtaataggcacaaaaccatccattcaggttctatatttgttcgaataaaa

baochun1 ggaagaactggtaataggcacaaaaccatccattcaggttctatatttgttcgaataaaa

************************************************************

baochun2 tgtttcgccaattccatacgtctaaccaaaaaatcctttcttcttccaacttttcgatct

baochun1 tgtttcgccaattccatacgtctaaccaaaaaatcctttcttcttccaacttttcgatct

************************************************************

baochun2 tcccattcatttcctgtgggcttttcttcccctaattctttccattctactaatgaataa

baochun1 tcccattcatttcctgtgggcttttcttcccctaattctttccattctactaatgaataa

************************************************************

baochun2 tcaataataattcgtaaatctagatccgctaattgttctcgaatagcacttgctccagta

baochun1 tcaataataattcgtaaatctagatccgctaattgttctcgaatagcacttgctccagta

************************************************************

baochun2 gagatctctcgatttcgaaatgtatcgaaaccctgtgtagtaaaaaaaagtgggatgctg

baochun1 gagatctctcgatttcgaaatgtatcgaaaccctgtgtagtaaaaaaaagtgggatgctg

************************************************************

baochun2 tatttccaagattggatttcatattcgaatgaacctcgtaatcgtaagaaagtgggtttt

baochun1 tatttccaagattggatttcatattcgaatgaacctcgtaatcgtaagaaagtgggtttt

************************************************************

baochun2 ttagctattggcctagcaaaagaaaaattaggataggatcctataggatctcccccttcc

baochun1 ttagctattggcctagcaaaagaaaaattaggataggatcctataggatctcccccttcc

************************************************************

baochun2 aaaatcggacatgaaagtttcctttcatccggctcaagtaggtataccaaataaagataa

baochun1 aaaatcggacatgaaagtttcctttcatccggctcaagtaggtataccaaataaagataa

************************************************************

baochun2 acaggggaattatcacttcaaaattatggaaccccccaaaaagatctacttcttactcaa

baochun1 acaggggaattatcacttcaaaattatggaaccccccaaaaagatctacttcttactcaa

************************************************************

baochun2 gttcctagtgaaaaccaagcaagatttcattgtattctaattttatgagttcttt-----

baochun1 gttcctagtgaaaaccaagcaagatttcattgtattctaattttatgagttctttattca

*******************************************************

baochun2 -attcaattacgacataaataaaatgtgaaattcttgagtaatctactgcccttcaaatg

baochun1 aattcaattacgacataaacaaaatgtgaaattcttgagtaatctactgcccttcaaatg

******************.****************************************

baochun2 atgaatccccttaaatacttaattaagagagtcccttggaattcataagagatttacttg

baochun1 atgaatccccttaaatacttaattaagagagtcccttggaattcataagagatttacttg

************************************************************

baochun2 tctatatattgtttcgttcaatcttttaggtctcaacttaacctcgacggttgtatcacg

baochun1 tctatatattgtttcgttcaatcttttaggtctcaacttaacctcgacggttgtatcacg

************************************************************

baochun2 atgcctttaaagcctatatgcgatggatagacttctgtaaccataacatattttctattt

baochun1 atgcctttaaagcctatatgcgatggatagacttctgtaaccataacatattttctattt

************************************************************

baochun2 gctctacgagaaacccaatttctttctaaaagataaggaataattccacaaaagcaaaag

baochun1 gctctacgagaaacccaatttctttctaaaagataaggaataattccacaaaagcaaaag

************************************************************

baochun2 aaagaagtctttttacgaggtacaatttttttatttgttacgaaatcgaccatagatcaa

baochun1 aaagaagtctttttacgaggtacaatttttttatttgttacgaaatcgaccatagatcaa

************************************************************

baochun2 ttcccccctttttgtatttggaagtattaaatacacccattattctgagcttcatgttcc

baochun1 ttcccccctttttgtatttggaagtattaaatacacccattattctgagcttcatgttcc

************************************************************

baochun2 tccttccaagagacatgtcaggtccagggcgggcatcccaattggattgaatgggatgac

baochun1 tccttccaagagacatgtcaggtccagggcgggcatcccaattggattgaatgggatgac

************************************************************

baochun2 agtttctcattcctaatctgtaaaatcaaaatttcgatcaaatcacacatcgcaatatac

baochun1 agtttctcattcctaatctgtaaaatcaaaatttcgatcaaatcacacatcgcaatatac

************************************************************

baochun2 caggccttctaattctttaagaggtttatctaaaatattcgcaatataactaggaagacg

baochun1 caggccttctaattctttaagaggtttatctaaaatattcgcaatataactaggaagacg

************************************************************

baochun2 ttttaaataccatacatgggttactgggcatgccagtttgatgtagcccatttgatatcg

baochun1 ttttaaataccacacatgggttactgggcatgccagtttgatgtagcccatttgatatcg

************.***********************************************

baochun2 tcgtattcgagaatcaacaaattcaactccgcattgttcacaaaatttcgggtcttcttt

baochun1 tcgtattcgagaatcaacaaattcaactccgcattgttcacaaaatttcgggtcttcttt

************************************************************

baochun2 ctcatctccgactactcgataatttccacaagcacaaattccactttttatcggaccaaa

baochun1 ctcatctccgactactcgataatttccacaagcacaaattccactttttatcggaccaaa

************************************************************

baochun2 aattctttcacaaaataatccatctttttccggtttattggttttgtaatgaaaagtata

baochun1 aattctttcacaaaataatccatctttttccggtttattggttttgtaatgaaaagtata

************************************************************

baochun2 gggttttgtcacctccccaactatctctccattaggtaggattttattggcccaagcact

baochun1 gggttttgtcacctccccaactatctctccattaggtaggattttattggcccaagcact

************************************************************

baochun2 tatttgttgaggagaaactgatccaattcggagttgttgatgtttataccggtcgatcat

baochun1 tatttgttgaggagaaactgatccaattcggagttgttgatgtttataccggtcgatcat

************************************************************

baochun2 agaaaaaaagttctgattcattccgattaagcttccttcctattaatctgaaagttcttt

baochun1 agaaaaaaagttctgattcattccgattaagcttccttcctattaatctgaaagttcttt

************************************************************

baochun2 tcagatacaaggaaatgattcagttctagagccaaagatcgtagttctctaactagtaat

baochun1 tcagatacaaggaaatgattcagttctagagccaaagatcgtagttctctaactagtaat

************************************************************

baochun2 cgaaaagattctggagcattctcaggatttggtattgttccgccaatgatcgtagtacca

baochun1 cgaaaagattctggagcattctcaggatttggtattgttccgccaatgatcgtagtacca

************************************************************

baochun2 agtacttcctggcgagcaataatatgatccgatttataagtaagcatctcttgtaaaata

baochun1 agtacttcctggcgagcaataatatgatccgatttataagtaagcatctcttgtaaaata

************************************************************

baochun2 tgggcaacaccaaatccctctaaagcccaaacctccatttcacctactcgttgtccccct

baochun1 tgggcaacaccaaatccctctaaagcccaaacctccatttcacctactcgttgtccccct

************************************************************

baochun2 tgcttagcccttcctctaaggggttgttgtgtaacaagtgcgtaatgcccactggaacgt

baochun1 tgcttagcccttcctctaaggggttgttgtgtaacaagtgcgtaatgcccactggaacgt

************************************************************

baochun2 ccatggattttatcatcaacttgatgaattaatttcaagatataaggctttcctattata

baochun1 ccatggattttatcatcaacttgatgaattaatttcaagatataaggctttcctattata

************************************************************

baochun2 acaggttgttcaaaaggatctcccgttctgccatcaaatattctactttttcctggatac

baochun1 acaggttgttcaaaaggatctcccgttctgccatcaaatattctactttttcctggatac

************************************************************

baochun2 tcgggctcaaatacccatggatttgctgtttgcttactggcttcatataattcagaaaac

baochun1 tcgggctcaaatacccatggatttgctgtttgcttactggcttcatataattcagaaaac

************************************************************

baochun2 acgagttttcgcgaagcttcttgttcatatctctcatcaaaaggcgctattcgataatgt

baochun1 acgagttttcgcgaagcttcttgttcatatctctcatcaaaaggcgctattcgataatgt

************************************************************

baochun2 cggtctagcagacttcccgctaaaccaagtgaacattcaaatatctgtcctacattcatt

baochun1 cggtctagcagacttcccgctaaaccaagtgaacattcaaatatctgtcctacattcatt

************************************************************

baochun2 cgtgagggtactcctaatgggttgaagaccatatcgacgggtcttccatcttgcaaatag

baochun1 cgtgagggtactcctaatgggttgaagaccatatcgacgggtcttccatcttgcaaatag

************************************************************

baochun2 ggcatatcttgtataggcaaaatttttgaaatgatacccttatttccatgtcttccagct

baochun1 ggcatatcttgtataggcaaaatttttgaaatgatacccttatttccatgtcttccagct

************************************************************

baochun2 actttatcacctactttgatttcacgtttctgtaaaatatatatgcgaattgtttctgga

baochun1 actttatcacctactttgatttcacgtttctgtaaaatatatatacgaattgtttctgga

********************************************.***************

baochun2 ttataactagagcctcctttcttctggatccatctcacatcaataacacgacccctacca

baochun1 ttataactagagcctcctttcttctggatccatctcacatcaataacacgacccctacca

************************************************************

baochun2 cctgtaggtagttttaggcaagtttcctttgacatagatacctgaataccaagtatggct

baochun1 cctgtaggtagttttaggcaagtttcctttgacatagatacctgaataccaagtatggct

************************************************************

baochun2 cgtaataatctatcttccggagcataggacgattctttcaccatctggggggttaattta

baochun1 cgtaataatctatcttccggagcataggacgattctttcaccatctggggggttaattta

************************************************************

baochun2 cctactaaaatatcacccgtttccacccaagatcccagcatcacaattccatttttatct

baochun1 cctactaaaatatcacccgtttccacccaagatcccagcatcacaattccatttttgtct

********************************************************.***

baochun2 aaattgcgtaacaaatgggcttctaaatgcggtatttcattagtgaccctttcggggcct

baochun1 aaattgcgtaacaaatgggcttctaaatgcggtatttcattagtgaccctttcggggcct

************************************************************

baochun2 tggcttgtcacatgagtcttaatttcgtatttccttatgttaaaagaagtataaatatct

baochun1 tggcttgtcacatgagtctgaatttcgtatttccttatgttaaaagaagtataaatatct

******************* ****************************************

baochun2 tcatataccaagcgctcgctaatgagtactgcatcttcaaaattgtaaccttcccatggc

baochun1 tcatataccaagcgctcgctaatgagtactgcatcttcaaaattgtaaccttcccatggc

************************************************************

baochun2 atataagccacgactacatttttccccaaagcgagttccccaccaactgtagcagcacca

baochun1 atataagccacgactacatttttccccaaagcgagttccccaccaactgtagcagcacca

************************************************************

baochun2 tccgctacaatttgtccctttttaatgcatcgaccccgttgaacctggggtttttgatgc

baochun1 tccgctacaatttgtccctttttaatgcatcgaccccgttgaacctggggtttttgatgc

************************************************************

baochun2 atacaagtatttttgttggaacgttgatacagaactaatggaatgcttatagtatctcca

baochun1 atacaagtatttttgttggaacgttgatacagaactaatggaatgcttatagtatctcca

************************************************************

baochun2 ttacctgataaaataatcttgtcagtatcggtataaatgatctttccctcatgttcggct

baochun1 ttacctgataaaataatcttgtcagtatcggtataaatgatctttccctcatgttcggct

************************************************************

baochun2 atagaaagagcccctgaatctaaagccgcttgacgttccagcccagttccaacaatgcac

baochun1 atagaaagagcccctgaatctaaagccgcttgacgttccagcccagttccaacaatgcac

************************************************************

baochun2 ttctcggaccgagaaagcggaactgcttgccgttgcatattagaactcattaaagcccga

baochun1 ttctcggaccgagaaagcggaactgcttgccgttgcatattagaactcattaaagcccga

************************************************************

baochun2 ttcgcatcattatgctcgataaaaggaatgagggaagctccaatcgaaaaatattggaag

baochun1 ttcgcatcattatgctcgataaaaggaatgagggaagctccaatcgaaaaatattggaag

************************************************************

baochun2 gggaaaatacttcgaaggtgagcctgttcccatgcaacagtcaagaattcttggcggtat

baochun1 gggaaaatacttcgaaggtgagcctgttcccatgcaacagtcaagaattcttggcggtat

************************************************************

baochun2 cgagctggaacaacctgttcttcctgaatgccccgattcaatgccaacgaatttcctgcc

baochun1 cgagctggaacaacctgttcttcctgaatgccccgattcaatgccaacgaatttcctgcc

************************************************************

baochun2 gctaccatatagtattcatctatacctggtgataaataaa------gtaccttttttgat

baochun1 gctaccatatagtattcatctctacctggtgataaataaagcatccgtaccttttttgat

********************* ****************** **************

baochun2 ttctcagaaatttcataaagtggactttctagaaatccccaatgaccaatcctggcatga

baochun1 ttctcagaaatttcataaagtggactttctagaaatccccaatgaccaatcctggcatga

************************************************************

baochun2 attgctaaggatccaataagcccaacattgattccttccgatgtgtcaattgggcaaata

baochun1 attgctaaggatccaataagcccaacattgattccttccgatgtgtcaattgggcaaata

************************************************************

baochun2 cgtccatagtggctaggatggatatcccgtatccgaaaactagcagttcgcgctgtcaat

baochun1 cgtccatagtggctaggatggatatcccgtatccgaaaactagcagttcgcgctgtcaat

************************************************************

baochun2 cctccagggcccaaataactcaattttctaccatgaactatttgtgtcaatggattagtt

baochun1 cctccagggcccaaataactcaattttctaccatgaactatttgtgtcaatggattagtt

************************************************************

baochun2 cgatccaaaacttgagataatgggtgtaagccgaaaaaagactcataagtagttgttaat

baochun1 cgatccaaaacttgagataatgggtgtaagccgaaaaaagactcataagtagttgttaat

************************************************************

baochun2 ggagttgaagttaccaaattctgaggggtcggtatcaatttatgtctaattgccccacat

baochun1 ggagttgaagttaccaaattctgaggggtcggtatcaatttatgtctaattgccccacat

************************************************************

baochun2 atagtccctcgaattacattttctaaccgaactaaagccaatcccagttgatcctgtaaa

baochun1 atagtccctcgaattacattttctaaccgaactaaagccaatcccagttgatcctgtaaa

************************************************************

baochun2 agatccgccccagaacgaatacgtttatttttcaaatgattcatatcgtcaagtgtaccc

baochun1 agatccgccccagaacgaatacgtttatttttcaaatgattcatatcgtcaagtgtaccc

************************************************************

baochun2 attccaaatttcattccaatcaaatgatccgcggcagccaatatatctcgcggtaataaa

baochun1 attccaaatttcattccaatcaaatgatccgcggcagccaatatatctcgcggtaataaa

************************************************************

baochun2 aatgtattgttcgtaggtatatcaaggttcaatcttcggttcatatttcgtcgaccaatt

baochun1 aatgtattgttcgtaggtatatcaaggttcagtcttcggttcatatttcgtcgaccaatt

*******************************.****************************

baochun2 cttcctaattcacatctttgttgaaagaatttcttttgtaattccttacataaggcttca

baochun1 cttcctaattcacatctttgttgaaagaatttcttttgtaattccttacataaggcttca

************************************************************

baochun2 gaaaataccggatccccacctacacaggcaaattgttgataaaactccaaaatggcattt

baochun1 gaaaataccggatccccacctacacaggcaaattgttgataaaactccaaaatggcattt

************************************************************

baochun2 tcttttgacccaattttttttttctccttatcattcagaaaagataaaaaaatttcagga

baochun1 tcttttgacccaattttttttttctccttatcattcagaaaagataaaaaaatttcagga

************************************************************

baochun2 taagaaacattctctagaatttctcttagattcgaacccatagccgatgatagaactaga

baochun1 taagaaacattctctagaatttctcttagattcgaacccatagccgatgatagaactaga

************************************************************

baochun2 atagatattttttgtttcctactcacacgagcccatatccttgcttttctatcaatctct

baochun1 atagatattttttgtttcctactcacacgagcccatatccttgcttttctatcaatctct

************************************************************

baochun2 aattctgatcttcctccccaatctgatattatggtgccggtatagaccgaaattccgtta

baochun1 aattctgatcttcctccccaatctgatattatggtgccggtatagaccgaaattccgtta

************************************************************

baochun2 tggtctaattctgaccggtaataaataccggggctttgtaatatttgattgatcacaatt

baochun1 tggtctaattctgaccggtaataaataccggggctttgtaatatttgattgatcacaatt

************************************************************

baochun2 ctgtatattccatttattatagaagttcccagagaattcattagaggaatgtttccaata

baochun1 ctgtatattccatttattatagaagttcccagagaattcattagaggaatgtttccaata

************************************************************

baochun2 aaaattgtttgttcttgcatgtctctacccgttttccaaattaatcccgcgggtacatat

baochun1 aaaattgtttgttcttgcatgtctctacccgttttccaaattaatcccgcggatacatat

****************************************************.*******

baochun2 aattcagaagaatatgtgagtgattcatacacagtatctctttcttttatcaagggttct

baochun1 aattcagaagaatatgtgagtgattcatacacagcatctctttcttttatcaagggttct

**********************************.*************************

baochun2 actaattgatatgtttccacaaataattgaaattcaatttcttgatctgtatcttcaatt

baochun1 actaattgatatgtttccacaaataattgaaattcaatttcttgatctgtatcttcaatt

************************************************************

baochun2 tttggaaacttagaaagttcttcggtcaagccctgatcaatgaacctacaaaagccttca

baochun1 tttggaaacttagaaagttcttcggtcaagccctgatcaatgaacctacaaaagccttca

************************************************************

baochun2 aattgtatctgattaaacccaggtattgtagacattccttcatttccatcccggagcatt

baochun1 aattgtatctgattaaacccaggtattgtagacattccttcatttccatcccggagcatt

************************************************************

baochun2 tgcatttcccatttatctaaaaatcccattcttggttcatttttcattgaatcatataga

baochun1 tgcatttcccatttatctaaaaatcccattcttggttcattcttcattgaatcatataga

*****************************************.******************

baochun2 tgatatagcaataatggaatttatattctgtttactgaattacatgaaattttacccaac

baochun1 tgatatagcaataatggaatttatattctgtttactgaattacatgaaattttacccaac

************************************************************

baochun2 tccctat-atggaatatattagatacgtatgaacggaagaataaatagaattttctactc

baochun1 tccctataatggaatatattagatacgtatgaacggaagaataaatagaattttctactc

******* ****************************************************

baochun2 aaattggaatttgtgcaacagataaaaatggaaaggaattgagaaaatatgccctaaaaa

baochun1 aaattggaatttgtgcaacagataaaaatggaaaggaattgagaaaatatgccctaaaaa

************************************************************

baochun2 agaattctgccacttcgacttattatgttatgggattttgtatagaatatcaaaacaaaa

baochun1 agaattctgccacttcgacttattatgttatgggattttgtataaaatatcaaaacaaaa

********************************************.***************

baochun2 agaatgaaatttcgaccatgattatgatattccatatctatttttttcacttaacccctt

baochun1 agaatgaaatttcgaccatgattatgatattccatatctatttttttca--------ctt

************************************************* ***

baochun2 aaccccttagtcacgtatttcattcgtcaaaaaacgattcgcagagaaaatagagatttt

baochun1 aaccccttagtcacgtatttcattcgtcaaaaaacgattcgcagagaaaatagagatttt

************************************************************

baochun2 ttagtagaatttctatagctataagtagaatacgattgtgaagtgtataagaggggttac

baochun1 ttagtagaatttctatagctataagtagaatacgattgtgaagtgtataagaggggttac

************************************************************

baochun2 tatttgtatttattaaacatgtgcagatatagctatctatctgccttctcccctcttatt

baochun1 tatttgtatttattaaacatgtgcagatatagctatctatctgtcttctcccctcttatt

*******************************************.****************

baochun2 gcctttatattcgaaacagtgggggtccaaaatttccatttttttttaatgaaaaataga

baochun1 gcctttatattcgaaacagtgggggtccaaaatttccatttttttttaatgaaaaataga

************************************************************

baochun2 atcatgaaaatttcctgagaattctctatcggcagcttctagaaataaaagactaccatc

baochun1 atcatgaaaatttcctgagaattctctatcggcagcttctagaaataaaagactaccatc

************************************************************

baochun2 tggataacatggggtttacatatagttatatctgttgttataatggaagttgagagggct

baochun1 tggataacatggggtttacatatagttatatctgttgttataatggaagttgagagggct

************************************************************

baochun2 tttttattgaaaagaacccatattcattagttatttataaatttctattttttatgtcat

baochun1 tttttattgaaaagaacccatattcattagttatttataaatttctattttttatgtcat

************************************************************

baochun2 taggaaaacacaaattcaaatccaaaaataattcatgaattcacagttagcaggcaatag

baochun1 tagga------aaattcaaatccaaaaataattcatgaattcacagttagcaggcaatag

***** *************************************************

baochun2 ttaatggttcaaattagttataaattttgtactaaaaatccacactttatttttcatttt

baochun1 ttaatggttcaaattagttataaattttgtactaaaaatccacactttatttttcatttt

************************************************************

baochun2 aaatagaaaaaggagagaaagggaggattttttttaggaaagggattaaggaaaatcgaa

baochun1 aaatagaaaaaggagagaaagggagga-tttttttaggaaagggattaaggaaaatcgaa

*************************** ********************************

baochun2 tgcaaaatacaagtacaataaaaaaaagaagttaaccgacccaaaagaaccaataaaggg

baochun1 tgcaaaatacaagtacaataaaaaaaagaagttaaccgacccaaaagaaccaataaaggg

************************************************************

baochun2 aaaaatttaatctagtttgtattcttttggcgacatggccgagcggtaaggcgggggact

baochun1 aaaaatttaatctagtttgtattcttttggcgacatggccgagcggtaaggcgggggact

************************************************************

baochun2 gcaaatcctttttccccagttcaaatctgggtgtcgcctaatcaaccaaatactcaaaat

baochun1 gcaaatcctttttccccagttcaaatctgggtgtcgcctaatcaaccaaatactcaaaat

************************************************************

baochun2 ctgctattctgttctgctgatttaactcttttgctaatagaactggctccattattcccc

baochun1 ctgctattctgttctgctgatttaactcttttgctaatagaactggctccattattcccc

************************************************************

baochun2 agcataagaaaaggtattttttatacttgtattttttatacttgtttgattcgaagcaag

baochun1 agcataagaaaag---------------gtattttttatacttgtttgattcgaagcaag

************* ********************************

baochun2 catcaggtataggttgctcgtaaaaaattctaaatccctgaagtctaagattatagtgat

baochun1 catcaggtataggttgctcgtaaaaaattctaaatccctgaagtctaagattatagtgat

************************************************************

baochun2 ggaaggggaggaatttcgatacggactcacgagagtctctgaatgctcaggcattgaatc

baochun1 ggaaggggaggaatttcgatacggactcacgagagtctctgaatgctcaggcattgaatc

************************************************************

baochun2 aatattctatatgaatagataattggaattttttataaatacaaatctatttcgttgatt

baochun1 aatattctatatgaatagataattggaattttttataaatacaaatctatttcgttgatt

************************************************************

baochun2 gattcatcaatagtgttgggtcagaatatatttttgactctgcaccattgattccactat

baochun1 gattcatcaatagtattgggtcagaatatatttttgactctgcaccattgattccactat

**************.*********************************************

baochun2 tattagtgagtaataatagaataattccttcatattcatagaaataggggacataattca

baochun1 tattagtgagtaataatagaataattccttcatattcatagaaataggggacataattca

************************************************************

baochun2 catggatatagtaagtctcgcttgggctgctttaatggtagtctttacattttctctttc

baochun1 catggatatagtaagtctcgcttgggctgctttaatggtagtctttacattttctctttc

************************************************************

baochun2 actcgtagtatggggaagaagtggactctagaggtactattttttaattgagttgaggaa

baochun1 actcgtagtatggggaagaagtggactctagaggtactattttttaattgagttgagg-a

********************************************************** *

baochun2 aaaaactctatcaattgttttatagatcattctgaaaagtttttttaacgattttaaata

baochun1 aaaaactctatcaattgttttatagatcattctgaaaagtttttttaacgattttaaata

************************************************************

baochun2 aaaatatatttattttgaaagtccattggattcgagtggaattaatgtattatataaata

baochun1 aaaatatatttattttgaaagtccattggattcgagtggaattaatgtattatataaata

************************************************************

baochun2 atactctttcaatcattcccactaattctttctaaattttcgatttcaacgataggctct

baochun1 atactctttcaatcattcccactaattctttctaaattttcgatttcaacgataggctct

************************************************************

baochun2 tcatagaattataaatagacaatgaggaagatcaaat--------------------ttt

baochun1 tcatagaattataaatagacaatgaggaagatcaaatttttatttgttttactgttcttt

************************************* ***

baochun2 tatttgttttactgttctaaagaaggaaccgttccggtaagtgtctagacacttgttctg

baochun1 tatttgttttactgttctaaagaaggaaccgttccggtaagtgtctagacacttgttctg

************************************************************

baochun2 agaaacaatataaattgtctaacaaaaaactatgtattttgccttaggagagtttcatat

baochun1 agaaacaatataaattgtctaacaaaaaactatgtattttgccttaggagagtttcatat

************************************************************

baochun2 tggccatttaccgcttgttttcttatttttgcggattcccatagaactccagaactcctg

baochun1 tggccatttaccgcttgttttcttatttttgaggattcccatagaactccagaactcctg

******************************* ****************************

baochun2 ttagtgactcaatcaattacctcttttacctctttaaaacgctaaactaaaaaaaaacga

baochun1 ttagtgactcaatcaattacctcttttacctctttaaaacgctaaact-aaaaaaaacga

************************************************ ***********

baochun2 cttgatttttttaatcaaacttccttcattgatcttactttttcgatagatatggagata

baochun1 cttgatttttttaatcaaacttccttcattgatcttactttttcgat--------agata

*********************************************** *****

baochun2 tggagatatggagattcatagtgaaaaaaatacgaaagacaaagaaatagtaagaattag

baochun1 tggagatatggagattcatagtgaaaaaaatacgaaagacaaagaaatagtaagaattag

************************************************************

baochun2 aacaaagttttctttcaattggaacaaatagatgtagcaaagaaatagaattagatacat

baochun1 aacaaagttttctttcaattggaacaaatagatgtagcaaagaaatagaattagatacat

************************************************************

baochun2 tccatatatggaattgatatctacatataggaagatccatcctattgtagtattgtagat

baochun1 tccatatatggaattgatatctacatataggaagatccatcctattgtagtattgtagat

************************************************************

baochun2 taagtttgtattattattagagtacaactttactacagtattttatacactgtagaactt

baochun1 taagtttgtattattattagagtacaactttactacagtattttatacactgtagaactt

************************************************************

baochun2 tttttacacgacaagaaaactacgagaaaggtatggtagaaagaaagatatgaatctttc

baochun1 tttttacacgacaagaaaactacgagaaaggtatggtagaaagaaagatatgaatctttc

************************************************************

baochun2 tttctaccatatactatcggatcgcatagaatactgacaattctagtccgcgcatttcat

baochun1 tttctaccatatactatcggatcgcatagaatactgacaattctagtccgcgcatttcat

************************************************************

baochun2 ttaagacgcggaatttgaatccttttcgtcagaagtaaaatttcggtcaaattttttaat

baochun1 ttaagacgtggaatttgaatccttttcgtcagaagtcaagtttcggtcaaatttattaat

********.*************************** **.************** *****

baochun2 catttttactttgattttggctgactgtttttacgtaaatgataagtagaaaagcagtag

baochun1 catttttactttgattttggctgactgtttttacgtaaatgataagtagaaaagcagtag

************************************************************

baochun2 gcacgagaacgaacaatgcagtagcaataaatgcaagaatattgacttccataatctaat

baochun1 gcacgagaacgaacaatgcagtagcaataaatgcaagaatattgacttccataatctaat

************************************************************

baochun2 cgtttttttttattttaatttcacaataactcgggatttaatcccatagagatgataaat

baochun1 cgtttttttttattttaatttcacaataactcgggatttaatcccatagagatgataaat

************************************************************

baochun2 cttttgcctgtcaattcaatggatgaactccctctcgatggtattgaatcgaatcaatat

baochun1 cttttgcctgtcaattcaatggatgaactccctctcgatggtattgaatcgaatcaatat

************************************************************

baochun2 cataaataacaatagttgagctatcaaataaattcatcgtcgagaattgaatagtatacc

baochun1 cataaataacaatagttgagctatcaaataaattcatcgtcgagaattgaatagtatacc

************************************************************

baochun2 ataaaaagatcttttatccacaccgaatcaaatgaaaaatgggattcttgatccaatcag

baochun1 ataaaaagatcttttatccacaccgaatcaaatgaaaaatgggattcttgatccaatcag

************************************************************

baochun2 gagttattttatttactgttccgttttttcttttcgataaactatcctacgcctttcgtg

baochun1 gagttattttatttactgttccgttttttcttttcgataaactatcctacgcctttcgtg

************************************************************

baochun2 tatcattatccgatgagatgctatttctcgaatgacccttccacttcccttaaccacaaa

baochun1 tatcattatccgatgagatgctatttctcgaacgacccttccacttccattaaccacaaa

********************************.*************** ***********

baochun2 ccaaactaaacaatagaggtgaaatggaaaaagaaagaagttcaattctaaacttttttt

baochun1 ccaaaataaacaatagaggtgaaatggaaaaagaaagaagttcaattctaaac-tttttt

***** *********************************************** ******

baochun2 ttataatgatctaattttctttgaagacaaagaggtgtgataaaaatgaatccgagtcga

baochun1 ttataatgatctaattttctttgaagacaaagagatgtgataaaaatgaatccgagtcga

**********************************.*************************

baochun2 aagtatctaatattttaccattatagtagcgtttttgatgtcgatgagactccgaaaata

baochun1 aagtatctaatattttatatttatagtagcgtttttgatgttgatgagactccgaaaata

*****************. *********************.******************

baochun2 gaataaatagggacgaaactttatgcatttcttcattaaattcattccttctcgaagaaa

baochun1 gaataaatagggacgaaactttatgcatttcttcattaaattcattccttctcgaagaaa

************************************************************

baochun2 ggtgtgattgtgggacgatctttatctttcttttaccctctttcctcagattattatatt

baochun1 ggtgtgattgtgggacgatctttatctttcttttaccctctttcctcagattattatatt

************************************************************

baochun2 aggactaggacacatatatatataaagttcagtgtgcaatttgaatgaaatagatttttc

baochun1 aggactaggacacatatatatataaagttcagtgtgcaatttgaatgaaatagatttttc

************************************************************

baochun2 aaattttaattagtaaatttactaattgaggttacccaaaatcataagcagaaacagaga

baochun1 aaattttaattagtaaatttactaattgaggttacccaaaatcataagcagaaacagaga

************************************************************

baochun2 aaatttaatttggaaacgtagctcatggggggaattcgattccctttattttgaatttgg

baochun1 aaatttaatttggaaacgtagctcatggggggaattcgattccctttattttgaatttgg

************************************************************

baochun2 ttcattataataaatgaattctatttgtgtatgtgctaccaagaaccctgcgatattctc

baochun1 ttcattataataaatgaattccatttgtgtatgtgctaccaagaaccctgcgatattctc

*********************.**************************************

baochun2 tgtacatattatattccattatgaacaatcgaaaaagaaaactcgatatgtcttggaatc

baochun1 tgtacatattatattccattatgaacaatcgaaaaagaaaactcgatacgtcttggaatc

************************************************.***********

baochun2 ctgaatataatgctaccaacaattgtactaatccaaatatatctttataccaccaatcaa

baochun1 ctgaatataatgctaccaacaattgtactaatccaaatatatctttataccaccaatcaa

************************************************************

baochun2 tactttttgtttgatgataaaaaatgcaaaaggaaagaaaaaaaagagagtttaattgtt

baochun1 tactttttgtttgatgataaaaaatgcaaaaggaaagaaaaaaaagagagtttaattgtt

************************************************************

baochun2 aattgatggatttattggattcgtcgggactgacggggctcgaacccgtagcttccgcct

baochun1 aattgatggatttattggattcgtcgggactgacggggctcgaacccgtagcttccgcct

************************************************************

baochun2 tgacagggcggtgctctaaccaattgaactacaatcccagggaaattaagggatatagaa

baochun1 tgacagggcggtgctctaaccaattgaactacaatcccagggaaattaagggatatagaa

************************************************************

baochun2 gaaaatttgactcctacgtatcgggtagttcggaaggacaagagtttataccatctcatg

baochun1 gaaaatttgactcctacgtatcgggtagttcggaaggacaagagtttataccatctcatg

************************************************************

baochun2 gtagattggcgaattattgggccgagctggatttgaaccagcgtagacatattgccaacg

baochun1 gtagattggcgaattattgggccgagctggatttgaaccagcgtagacatattgccaacg

************************************************************

baochun2 aatttacagtccgtccccattaaccgctcgggcatcgacccaggaagaatccattttaga

baochun1 aatttacagtccgtccccattaaccgctcgggcatcgacccaggaagaatccattttaga

************************************************************

baochun2 cttattggtaatccatgatatgatcaacttcctttcgtagtaccctacccccaggggaag

baochun1 cttattggtaatccatgatatgatcaacttcctttcgtagtaccctacccccaggggaag

************************************************************

baochun2 tcgaatccccgctgcctccttgaaagagagatgtcctgaaccactagacgatgggggcat

baochun1 tcgaatccccgctgcctccttgaaagagagatgtcctgaaccactagacgatgggggcat

************************************************************

baochun2 acttgatcgaccgccatcatactatgatcataatatcaacagttttttgaaattgtc---

baochun1 acttgatcgaccgccatcatactatgatcataatatcaacagttttttgaaattgtcaat

*********************************************************

baochun2 ----aatataaatataatggaatgacatgatttgatccaagctctattttcattatttca

baochun1 ataaaatataaatataatggaatgacatgatttgatccaagctctattttcattatttca

********************************************************

baochun2 taatttttttttattcgttatttatgaattattcattttacttcgattatatatattatt

baochun1 taatttttttttattcgttatttatgaattattcattttacttcgattatatatattatt

************************************************************

baochun2 atagaacgaatttctaattattttaatttatattaaataataataacatagaattaaata

baochun1 atagaacgaatttataattattttaatttat---------------------attaaata

************* ***************** ********

baochun2 ataataacatagaaaaattttagtataaaaaatatgatttggcctggaatctaagaattt

baochun1 ataataacatagaaaaattttagtataaaaaatatgatttggcctggaatctaagaattt

************************************************************

baochun2 gtcgaaaaagagagagtttaattctattttttacaatggattcatttattgttaagatat

baochun1 gtcgaaaaagagagagtttaattctattttttacaatggattcatttattgttaagatat

************************************************************

baochun2 gcctggcttacactaagctaggagattcataaatgagaaatctgagaagagggatcaaga

baochun1 gcctggcttacactaagctaggagattcataaatgagaaatctgagaagagggatcaaga

************************************************************

baochun2 taagttatcgaaaatttgttttctctaattcgaatagaattactcgaattctaatttagt

baochun1 taagttatcgaaaatttgttttctctaattcgaatagaattactcgaattataatttagt

************************************************** *********

baochun2 tcaaaggtcgtccttgaaacaagtcattgcatttttctatacttgctatggaaatccttt

baochun1 tcaaaggtcgtccttgaaacaagtcattgcatttttctatacttgctatggaaatccttt

************************************************************

baochun2 ttcttattcaagaataagctactaggactctactgcatgtacttcgtgtatataatatat

baochun1 ttcttattcaagaataagctactaggactctactgcatgtacttcgtgtatataatatat

************************************************************

baochun2 gtacagaaatctattttatccacatagcgactcattcaagaattgaattcaatgggccct

baochun1 gtacagaaatctattttatccacatagcgactcattcaagaattgaattcaatgggccct

************************************************************

baochun2 tttaactcagcggtagagtaacgccatggtaaggcgtaagtcatcggttcaaatccgata

baochun1 tttaactcagcggtagagtaacgccatggtaaggcgtaagtcatcggttcaaatccgata

************************************************************

baochun2 aggggctttagtccttttttcataaaactccagtcataactttttttggaagggagaata

baochun1 aggggctttagtccttttttcataaaactccagtcataactttttttggaagggagaata

************************************************************

baochun2 aaagtatttttaatatttgtatgtgtaataaaaaaagtaaccaactgtattgtataataa

baochun1 aaagtatttttaatatttgtatgtgtaataaaaaaagtaaccaactgtattgtataataa

************************************************************

baochun2 attatcattctaatgagttagagtatattataatgaattttagtagagtagtgaacattt

baochun1 attatcattctaatgagttagagtatattataatgaattttagtagagtagtgaacattt

************************************************************

baochun2 tttagtaaattctcattatcatgaataatgataagtcgtctcttgaatcgctaaataccc

baochun1 tttagtaaattctcattatcatgaataatgataagtcgtctcttgaatcgccaaataccc

***************************************************.********

baochun2 ctactttctatatctgccaatttaatctattgtaaaaatgagaaattaacaaacaaaaga

baochun1 ctactttctatatctgccaatttaatctattgtaaaaatgagaaattaacaaacaaaaga

************************************************************

baochun2 aaaagtaggtggacctgacccattgaatcatgactatatctgctattctgatattaaaat

baochun1 aaaagtaggtggacctgacccattgaatcatgactatatctgctattctgatattaaaat

************************************************************

baochun2 tcgatagagatgaaattggaccgattgactcctttttttgactccacaagaatttgtcga

baochun1 tcgatagagatgaaattggaccggttgactcctttttttgactccacaagaatttgttga

***********************.*********************************.**

baochun2 tatttccgattcaatcgacttgttcctagacgtttcatacgaataaatagttattccgct

baochun1 tatttccgattcaatctacttgttcctagacgtttcatacgaataaatggttattccgct

**************** *******************************.***********

baochun2 actccacggaaaaatttttagtttattacaagtcacaacataaaagccctttttaatttc

baochun1 actccacggaaaaatttttagtttattacaagtcacaacataaaagccctttttaatttc

************************************************************

baochun2 tttctttgattccagatcaagattcagttttattcccagtttcctatttttatttaatat

baochun1 tttctttgattccagatcaagattcagttttattcccagtttcctatttttatttaatat

************************************************************

baochun2 tttattgaatttgagttcgaatttaatgaaaaaatagaaacttatcccttttaatctaac

baochun1 tttattgaatttgagttctaatttaatgaaaaaatagaaacttatcccttttaatctaac

****************** *****************************************

baochun2 atataaaaagaaatagaaaaatagtcgaagtccctttcttcgacttgcttgtggaaagag

baochun1 atataaaaagaaatagaaaaatagtcgaagtccctttcttcgacttgcttgtggaaagag

************************************************************

baochun2 atactttggaggtttctattcatcgaaaagaaaaggaaacataccaaaaa-agtcaattt

baochun1 atactttggaggtttctattcatcgaaaagaaaaggaaacataccaaaaaaagtaaattt

************************************************** *** *****

baochun2 atagagaattaataaataaaagtctgatactgtagtagtttagtatatagataaattaga

baochun1 atagagaattaataaataaaagtctgatactgtagtagtttagtatatagataaattaga

************************************************************

baochun2 agaagataaaaaaatatttttattttttcaatagcacgaacaagatccaaataataagat

baochun1 agaagataaaaaaatatttttattttttcaatagcacgaacaagatccaaataataagat

************************************************************

baochun2 taagattaattgatagaacaaggggataagtctgaggatcaaccgctagcgagagagggt

baochun1 taagattaattgatagaacaaggggataagtctgaggatcaaccgctagcgagagagggt

************************************************************

baochun2 gccgcttgttccgtgcaaagttctttcaaaaaatgcatcgatttgatgaatatattatta

baochun1 gccgcttgttccgtgcaaagttctttcaaaaaatgcatcgatttgatgaatatattatta

************************************************************

baochun2 agttaataggttataagaagaaaattcatagttccgacgcttggacttagtaataataag

baochun1 agttaataggttataagaagaaaattcatagttccgacgcttggacttagtaataataag

************************************************************

baochun2 aagaatcgaattgagttcatgaatttacctaggtcaggttatagaccaatataatataag

baochun1 aagaatcgaattgagttcatggatttacctaggtcaggttatagaccaatataatataag

*********************.**************************************

baochun2 gattttttctcttcgaaacccactacactaaatggggcagtgcgcgagaaatcatacaca

baochun1 gattttttctcttcgaaacccactacactaaatggggcagtgcgcgagaaatcatacaca

************************************************************

baochun2 aataatagaatcttcgggtgccctgaaaatgctatgaggtgttcggaaatggtcgaagta

baochun1 aataatagaatcttcgggtgccctgaaaatgctatgaggtgttcggaaatggtcgaagta

************************************************************

baochun2 gttgaataggaggattactatgactatagcccttggtaaatttaccaaagatgaaaaaga

baochun1 gttgaataggaggattactatgactatagcccttggtaaatttaccaaagatgaaaaaga

************************************************************

baochun2 tttatttgatcttatggatgactggttacggagggaccgtttcgtttttgtaggctggtc

baochun1 tttatttgatcttatggatgactggttacggagggaccgtttcgtttttgtaggctggtc

************************************************************

baochun2 cggtttattgctctttccttgtgcatatttcgctttagggggttggtttacaggtacaac

baochun1 cggtttattgctctttccttgtgcatatttcgctttagggggttggtttacaggtacaac

************************************************************

baochun2 ctttgtaacttcatggtatactcatggattggccagttcctatttggagggctgcaattt

baochun1 ctttgtaacttcatggtatactcatggattggccagttcctatttggagggctgcaattt

************************************************************

baochun2 cttaacggctgcagtttctacacctgctaatagtttagcacattctttgttgttactctg

baochun1 cttaacggctgcagtttctacacctgctaatagtttagcacattctttgttgttactctg

************************************************************

baochun2 gggtcctgaagcacaaggagattttactcgttggtgtcaattgggcggtctgtggacttt

baochun1 gggtcctgaagcacaaggagattttactcgttggtgtcaattgggcggtctgtggacttt

************************************************************

baochun2 tgttgctctccacggtgctttcggactaataggtttcatgttacgtcaatttgaacttgc

baochun1 tgttgctctccacggtgctttcggactaataggtttcatgttacgtcaatttgaacttgc

************************************************************

baochun2 tcgatctgtgcaattacgcccttataatgcaattgcattttctggcccaattgctgtttt

baochun1 tcgatctgtgcaattacgcccttataatgcaattgcattttctggcccaattgctgtttt

************************************************************

baochun2 tgtttctgtattcttgatttatccactaggtcagtcgggttggttttttgcgcctagctt

baochun1 tgtttctgtattcttgatttatccactaggtcagtcgggttggttttttgcgcctagctt

************************************************************

baochun2 tggtgtagcagctatatttcggttcatcctcttttttcaagggtttcataattggacgtt

baochun1 tggtgtagcagctatatttcggttcatcctcttttttcaagggtttcataattggacgtt

************************************************************

baochun2 gaacccatttcatatgatgggagttgccggtgtattgggggcagctttgctatgtgctat

baochun1 gaacccatttcatatgatgggagttgccggtgtattgggggcagctttgctatgtgctat

************************************************************

baochun2 tcatggtgctaccgtagaaaatactttatttgaagatggtgatggtgcaaatacattccg

baochun1 tcatggtgctaccgtagaaaatactttatttgaagatggtgatggtgcaaatacattccg

************************************************************

baochun2 tgcttttaacccaactcaagccgaagaaacttattcaatggttaccgctaaccgcttttg

baochun1 tgcttttaacccaactcaagccgaagaaacttattcaatggttaccgctaaccgcttttg

************************************************************

baochun2 gtcccaaatctttggggttgctttttctaataaacgttggttacatttctttatgttatt

baochun1 gtcccaaatctttggggttgctttttctaataaacgttggttacatttctttatgttatt

************************************************************

baochun2 tgtaccagtaaccggtttatggatgagtgctcttggagtagtcggactagctctgaactt

baochun1 tgtaccagtaaccggtttatggatgagtgctcttggagtagtcggactagctctgaactt

************************************************************

baochun2 acgtgcttatgactttgtttctcaggaaattcgtgcagcggaagatcctgaatttgagac

baochun1 acgtgcttatgactttgtttctcaggaaattcgtgcagcggaagatcctgaatttgagac

************************************************************

baochun2 tttctacaccaaaaatattctcttaaacgaaggtattcgtgcttggatggcggctcaaga

baochun1 tttctacaccaaaaatattctcttaaacgagggtattcgtgcttggatggcggctcaaga

******************************.*****************************

baochun2 tcagcctcatgaaaaccttatattccctgaggaggttctaccccgtggaaacgctcttta

baochun1 tcagcctcatgaaaaccttatattccctgaggaggttttaccccgtggaaacgctcttta

*************************************.**********************

baochun2 atggaactttagctttagcaggtcgtgaccaagaaaccaccggtttcgcttggtgggccg

baochun1 atggaactttagctttagcaggtcgtgaccaagaaaccaccggtttcgcttggtgggccg

************************************************************

baochun2 gaaatgcccgacttatcaatttatctggtaaactactaggggctcatgtagcccatgctg

baochun1 gaaatgcccgacttatcaatttatctggtaaactactaggggctcatgtagcccatgctg

************************************************************

baochun2 gattaatagtattctgggccggcgcaatgaacctatttgaagtggctcattttgtcccag

baochun1 gattaatcgtattctgggccggcgcaatgaacctatttgaagtggctcattttgtcccag

******* ****************************************************

baochun2 agaagcctatgtatgaacaaggattaattttacttccccacctagctaccctaggctggg

baochun1 agaagcctatgtatgaacaaggattaattttacttccccacctagctaccctaggctggg

************************************************************

baochun2 gggtaggtgctggtggggaagttatagacacctttccctactttgtctctggagtacttc

baochun1 gggtaggtgctggtggggaagttatagacacctttccctactttgtctctggagtacttc

************************************************************

baochun2 acttaatttcttctgcagtattgggttttggcggtatttatcatgcacttctgggacctg

baochun1 acttaatttcttctgcagtattgggttttggcggtatttatcatgcacttctgggacctg

************************************************************

baochun2 agactcttgaagaatcttttccattcttcggttatgtatggaaagatagaaataaaatga

baochun1 agactcttgaagaatcttttccattcttcggttatgtatggaaagatagaaataaaatga

************************************************************

baochun2 ccacaattttaggtattcatttaatcttgttaggtataggtgcttttcttctagtattta

baochun1 ccacaattttaggtattcatttaatcttgttaggtataggtgcttttcttctagtattta

************************************************************

baochun2 aggctctttattttgggggcgtatatgatacctgggctcccggagggggagatgtaagaa

baochun1 aggctctttattttgggggcgtatatgatacctgggctcccggagggggagatgtaagaa

************************************************************

baochun2 aaattaccaacttgacacttagcccaagtattatatttggttatttactaaaatcgccct

baochun1 aaattaccaacttgacacttagcccaagtattatatttggttatttactaaaatcgccct

************************************************************

baochun2 ttggaggggaaggatggattgttagtgtagacgatttggaagatattatcggaggacatg

baochun1 ttggaggggaaggatggattgttagtgtagacgatttggaagatattatcggaggacatg

************************************************************

baochun2 tatggttaggttccatttgtatatttggtggaatctggcatatcttaaccaaacccttcg

baochun1 tatggttaggttccatttgtatatttggtggaatctggcatatcttaaccaaacccttcg

************************************************************

baochun2 cctgggcgcgccgtgcgcttgtatggtctggagaggcttacttgtcttatagtttagctg

baochun1 cctgggcgcgacgtgcgcttgtatggtctggagaggcttacttgtcttatagtttagctg

********** *************************************************

baochun2 ctttatctctttttggtttcattgcttgttgttttgtctggttcaataataccgcttatc

baochun1 ctttatctctttttggtttcattgcttgttgttttgtctggttcaataataccgcttatc

************************************************************

baochun2 ctagtgagttttatgggcctactggtccagaagcttctcaagctcaagcatttacttttc

baochun1 ctagtgagttttatgggcctactggtccagaagcttctcaagctcaagcatttacttttc

************************************************************

baochun2 tagttagagaccaacgtcttggggctaacgtggggtccgctcaaggacctaccgggttag

baochun1 tagttagagaccaacgtcttggggctaacgtggggtccgctcaaggacctaccgggttag

************************************************************

baochun2 gtaaatatctaatgcgttccccgactggcgaagtcatttttggcggagaaactatgcgtt

baochun1 gtaaatatctaatgcgttccccgactggcgaagtcatttttggcggagaaactatgcgtt

************************************************************

baochun2 tttgggatttgcgtgctccgtggttagaacctctaagaggtccaaacggattggacttaa

baochun1 tttgggatttgcgtgctccgtggttagaacctctaagaggtccaaacggattggacttaa

************************************************************

baochun2 gtaggctgaaaaaagacatacaaccttggcaagaacggcgttccgcagaatatatgaccc

baochun1 gtaggctgaaaaaagacatacaaccttggcaagaacggcgttccgcagaatatatgaccc

************************************************************

baochun2 atgctcctttaggttctttaaattccgtcggtggtgtagctactgagatcaatgcagtca

baochun1 atgctcctttaggttctttaaattccgtcggtggtgtagctactgagatcaatgcagtca

************************************************************

baochun2 attatgtctctcctagaagttggttatctacctcgcattttgttctaggattcttcttat

baochun1 attatgtctctcctagaagttggttatctacctcgcattttgttctaggattcttcttat

************************************************************

baochun2 tcgtaggtcatttgtggcacgcgggaagggctcgtgcagctgcagcgggctttgaaaaag

baochun1 tcgtaggtcatttgtggcacgcgggaagggctcgtgcagctgcagcgggctttgaaaaag

************************************************************

baochun2 gaattgatcgtgatttggaacctgttctttccatgacccctcttaattgagacaagagat

baochun1 gaattgatcgtgatttggaacctgttctttccatgacccctcttaattgagacaagagat

************************************************************

baochun2 cccacgtttgaagtagaaattaatttgtttccaccatacatctttggatcagggcatacc

baochun1 cccacgtttgaagtagaaattaatttgtttccaccatacatctttggatcagggcatacc

************************************************************

baochun2 taaaaagtattcctttttctttttttttcaattaatttcgatctaatttcttttttagaa

baochun1 taaaaagtattcctttttctttttttttcaattaatttcgatctaatttcttttttagaa

************************************************************

baochun2 tctatttttttatggcttggctaggtgggatagccgagccattcccctttgtttatatta

baochun1 tctatttttttatggcttggctaggtgggatagccgagccattcccctttgtttatatta

************************************************************

baochun2 tcggtcagagaaaaaccaataatgattaaaaggagagagagggattcgaaccctcgatag

baochun1 tcggtcagagaaaaaccaataatgattaaaaggagagagagggattcgaaccctcgatag

************************************************************

baochun2 ttctttggtcagaactataccggttttcaagaccggggctatcaaccactcagccatctc

baochun1 ttctttggtcagaactataccggttttcaagaccggggctatcaaccactcagccatctc

************************************************************

baochun2 tccgaaagaccataagatcatttttattttattcctccgaatcgaacatagctatataag

baochun1 tccgaaagaccataagatcatttttattttattcctccgaatcgaacatagctatataag

************************************************************

baochun2 tggatgcgaccactatcggtaaaaagatatcatatcgggtgtgactctacaggtcgatct

baochun1 tggatgcgaccactatcggtaaaaagatatcatatcgggtgtgactctacaggtcgatct

************************************************************

baochun2 atttatccatataaatagatgcataacccatcctacccatttatttgtgaaatacaaaaa

baochun1 atttatccatataaatagatgcatgacccatcctacccatttgtttgtgaaatacaaaaa

************************.*****************.*****************

baochun2 aatttcattccactcgactacatgaataaagtactaaaaaaattagtatactaattagtt

baochun1 aatttcattccactcgactacatgaataaagtactaaaaaaatgagtatactaattagtt

******************************************* ****************

baochun2 atatagagtcaatgtattcatgataaaatcccccacgatggagtttattccaaagataga

baochun1 atatagagtcaatgtattcatgataaaatcccccacgatggagtttattccaaagataga

************************************************************

baochun2 gggattaaatggtatagttcatttgttggtagtttggaggattaaaagtatgactattgc

baochun1 gggattaaatggtatagttcatttgttggtagtttggaggattaaaagtatgactattgc

************************************************************

baochun2 tttccaattagctgtttttgcattaattgctacgtcatcaatcttattgattagcgtacc

baochun1 tttccaattagctgtttttgcattaattgctacgtcatcaatcttattgattagcgtacc

************************************************************

baochun2 ccttgtatttgcttctcctgatggttggtcgagtaacaaaaatgttgtattttctggtac

baochun1 ccttgtatttgcttctcctgatggttggtcgagtaacaaaaatgttgtattttctggtac

************************************************************

baochun2 atctttatggattggattagtttttctggtgggtatccttaattctctcatctcttgaac

baochun1 atctttatggattggattagtttttctggtgggtatccttaattctctcatctcttgaac

************************************************************

baochun2 ttatttataccagctccaaaaatcaaatgacccccctgaattcttttcggttgtgagaca

baochun1 ttattcataccagctccaaaaatcaaatgacccccctgaattcttttcggttgtgagaca

*****.******************************************************

baochun2 cattcaaatttaatattaatataagtcccccaaatacaaataaagaaaaaaatgataggg

baochun1 cattcaaatttaatattaatataagtcccccaaatacaaataaagaaaaaaatgataggg

************************************************************

baochun2 gggtcaaaaaaaaatccttgcaatccccctctgaaaaaagtctttggcttggcactacat

baochun1 gggtcaaaaaaaaatccttgcaatccccctctgaaaaaagtctttggcttggcactacat

************************************************************

baochun2 acacaaatataatccagacatatatatcatatatgtgtgatacatattatgtatcaagaa

baochun1 acacaaatataatccagacatatatatcatatatgtgtgatacatattatgtatcaagaa

************************************************************

baochun2 ctaaaaaaatgcggatatagtcgaatggtaaaatttctctttgccaaggagaagacgcgg

baochun1 ctaaaaaaatgcggatatagtcgaatggtaaaatttctctttgccaaggagaagacgcgg

************************************************************

baochun2 gttcgattcccgctatccgccactagagtgaagtcattcctttttaatatgattaaaggg

baochun1 gttcgattcccgctatccgccactagagtgaagtcattcctttttaatatgattaaaggg

************************************************************

baochun2 ttcggtatagttgactgtgatagaccccccccaaaaaaaaaaaggtaattaattactagt

baochun1 ttcggtatagttgactgtgatagattcccccc-cccaaaaaaaggtaattaattactagt

************************..****** ************************

baochun2 taacagagttaaacctaattttaaaaagatgttgcggagacaggatttgaacccgtgacc

baochun1 taacagagttaaacctaattttaaaaagatgttgcggagacaggatttgaacccgtgacc

************************************************************

baochun2 tcaaggttatgagccttgcgagctaccaaactgctctaccccgcgctaaactaaagagaa

baochun1 tcaaggttatgagccttgcgagctaccaaactgctctaccccgcgctaaactaaagagaa

************************************************************

baochun2 gaactgaaaactaataaaaaacaagtattgaatgcgaccctctaccatatctgtacaaat

baochun1 gaactgaaaactaataaaaaacaagtattgaatgcgaccctctaccatatctgtacaaat

************************************************************

baochun2 agaatagcccatttatacagaatggtaaaggggctctctatgatcgatgatcatagaaat

baochun1 agaatagcccatttatacagaatggtaaaggggctctctatgatcgatgatcatagaaat

************************************************************

baochun2 gaagggagatttttatcttaccaacttgatcttgttgcccctggcaacaaacatgcctga

baochun1 gaagggagatttttatcttaccaacttgatcttgttgcccctggcaacaaacatgcctga

************************************************************

baochun2 accatttcacgaagtatgtgtccggataggccgaagtctcgaaagttagctcggggcctt

baochun1 accatttcacgaagtatgtgtccggataggccgaagtctcgaaagttagctcggggcctt

************************************************************

baochun2 ccggtcgaaaaacaacgccgatgaaggcgtgtaggtgcgctattccgcggtagggattgt

baochun1 ccggtcgaaaaacaacgccgatgaaggcgtgtaggtgcgctattccgcggtagggattgt

************************************************************

baochun2 aactttccgtgaatttcccacttgtcactcaatgactgaactttgcttatttcttttttt

baochun1 aactttccgtgaatttcccacttgtcactcaatgactgaactttgcttatttcttttttt

************************************************************

baochun2 gaggatcgacgaatcaaatgatatttctgttccaaatttttccttttcttttccctctga

baochun1 gaggatcgacgaatcaaatgatatttctgttccaaatttttccttttcttttccctctga

************************************************************

baochun2 atcaaacttttttttgccataatggttcaatttctattcgtatccatgatacaagtcgga

baochun1 atcaaacttttttttgccataatggttcaatttctattcgtatccatgatacaagtcgga

************************************************************

baochun2 tcctagatgtaaaaataaaagaagttggactacttccccattaaaagaaatgagattctc

baochun1 tcctagatgtaaaaataaaagaagttggactacttccccatcaaaagaaatgagattctc

*****************************************.******************

baochun2 ccagatatgcgatactaaaaaaattaaccaaatttacccgacgtagaggcaatcaagaaa

baochun1 ccagatatgcgatactaaaaaaattaaccaaatttacccgacgtagaggcaatcaagaaa

************************************************************

baochun2 gctgcataagtgaatatataacctacagaaaagtgggctaatccaaccaatcttgcttgc

baochun1 gctgcataagtgaatatataacctacagaaaagtgggctaatccaaccaatcttgcttgc

************************************************************

baochun2 acaatggaaagtgccaccggtttatctctccatcgaatcaaattggccaaaggtgtacgt

baochun1 acaatggaaagtgccaccggtttatctctccatcgaatcaaattggccaaaggtgtacgt

************************************************************

baochun2 tcatgagcccatgctaaagtttcaatcaattcctgccaatagccacgccaggaaattaag

baochun1 tcatgagcccatgctaaagtttcaatcaattcctgccaatagccacgccaggaaattaag

************************************************************

baochun2 aacataaatccagtagcccaaacaagatgtccaaataagaacatccacgcccagaccgat

baochun1 aacataaatccagtagcccaaacaagatgtccaaataagaacatccacgcccagaccgat

************************************************************

baochun2 aaactattcataccaaaaggattatatccattgataagttgggaagagtttaaccataaa

baochun1 aaactattcataccaaaaggattatatccattgataagttgggaagagtttaaccataaa

************************************************************

baochun2 taatctcttaaccatcccatcaaataagtggacgattcattaaactgcgaaacgttaccc

baochun1 taatctcttaaccatcccatcaaataagtggacgattcattaaactgcgaaacgttaccc

************************************************************

baochun2 tgccataatgtgatgtgtttccaatgccaataaaaagtaacccatccaatggtatttaac

baochun1 tgccataatgtgatgtgtttccaatgccaataaaaagtaacccatccaatggtatttaac

************************************************************

baochun2 atccaaaaaaccgccaaataaaatgcgtcccaagctgaaatatcacaagtaccacctcgc

baochun1 atccaaaaaaccgccaaataaaatgcgtcccaagctgaaatatcacaagtaccacctcgc

************************************************************

baochun2 cccgggccatcgcaaggaaaactataaccgaaatcctttttatctggcattaacttggaa

baochun1 cccgggccatcgcaaggaaaactataaccgaaatcctttttatctggcattaacttggaa

************************************************************

baochun2 ccgcgtgcatctaaagcaccttttactaagatcaatgtagttgtatgtaaacctagagca

baochun1 ccgcgtgcatctaaagcgccttttactaagatcaatgtagttgtatgtaaacctagagca

*****************.******************************************

baochun2 atagcatgatgaaccaaaaagtctccaggacctattgttaagaagagtgaattactattt

baochun1 atagcatgatgaaccaaaaagtctccaggacctattgttaagaagagtgaattactattt

************************************************************

baochun2 tcattaacagcatttaaccaaccgggcaaccatatgcttcgacccgcattgaacgctgga

baochun1 tcattaacagcatttaaccaaccgggcaaccatatgcttcgacccgcattgaacgctggg

***********************************************************.

baochun2 ccactcgttgaagataaaagtacatcgaacccatacgaagttttaccatgagcagattgg

baochun1 ccactcgttgaagataaaagtacatcgaacccatatgaagttttaccatgagcagattgg

***********************************.************************

baochun2 atccattgagcaaatataggttcgatcaagatttgtttttccggagtaccaaaagcaagc

baochun1 atccattgagcaaatataggttcgatcaggatttgtttttccggagtaccaaaagcaagc

****************************.*******************************

baochun2 atgacatcattatgaacataaagtcccaaggtatggaaccccagaaagagactggcccaa

baochun1 atgacatcattatgaacataaagtcccaaggtatggaaccccagaaagagactggcccaa

************************************************************

baochun2 cttaaatgggatatgatagcttctttatggtctaacattcttgccaatacattgtcctca

baochun1 cttaaatgggatatgatagcttctttatggtctaacattcttgccaatacattgtcctca

************************************************************

baochun2 ttctgttccggattataatctctaatgaaaaatatagctccgtgagcaaaagctcctgtc

baochun1 ttctgttccggattataatctctaatgaaaaatatagctccgtgagcaaaagctcctgtc

************************************************************

baochun2 atgatgaaccctgcgatgtattggtgatgcgtatataacgcagcttgagtagtaaagtct

baochun1 atgatgaaccctgcgatgtattggtgatgcgtatataacgcagcttgagtagtaaagtct

************************************************************

baochun2 tgtgctatgaatgcataagcaggtaaagaatacatgtgttgagctaccaaagaagtaata

baochun1 tgtgctatgaatgcataagcaggtaaagaatacatgtgttgagctaccaaagaagtaata

************************************************************

baochun2 acccctaaagaagctagagccaaacctaattgaaaatgaattgaattattaattgtgtca

baochun1 acccctaaagaagctagagccagacctaattgaaaatgaattgaattattaattgtgtca

**********************.*************************************

baochun2 taaagacccttatggccacgtcccaaccgtccccccgggggaacgtgtgcatctaaaaga

baochun1 taaagacccttatggccacgtcccaaccgtcccccggggggaacgtgtgcatctaaaaga

*********************************** ************************

baochun2 tctttcatactgtgtccaattccgaagttcgttctatacatatgaccggcaacgagaaaa

baochun1 tctttcatactgtgtccaattccgaagttcgttctatacatatgaccggcaacgagaaaa

************************************************************

baochun2 ataaatgcaatagctaaatgatgatgagcaatatcagtcagccataaactttgcgtttgt

baochun1 ataaatgcaatagctaaatgatgatgagcaatatcagtcagccataaactttgcgtttgt

************************************************************

baochun2 ggatggaatcctccaagaagggttagaatggcagttccggcgccttgggaggtactaaat

baochun1 ggatggaatcctccaagaagggttagaatggcagttccggcgccttgggaggtactaaat

************************************************************

baochun2 aaatgactacttgaatcggggttttgagcataaagattccactgacccgtaaacagaggg

baochun1 aaatgactacttgaatcggggttttgagcataaagattccactgacccgtaaacagaggg

************************************************************

baochun2 cctaacccttggggatgcggtaatacatttaagaaattattccatcggacgtactctcct

baochun1 cctaacccttggggatgcggtaatacatttaagaaattattccatcggacgtactctcct

************************************************************

baochun2 ctagatgcaggaatagcgacatgaactaaatgccccgtccaagccaaggaacttactccg

baochun1 ctagatgcaggaatagcgacatgaactaaatgccccgtccaagccaaggaacttactccg

************************************************************

baochun2 aaaagtcctgacaaatgatgattgagacgagattcggcatttttgaaccacgaaacactt

baochun1 aaaagtcctgacaaatgatgattgagacgagattcggcatttttgaaccacgaaacactt

************************************************************

baochun2 ggtttccatttcggttgtagatgtaaccaacccgctattaaagatatagcagaaagaaat

baochun1 ggtttccatttcggttgtagatgtaaccaacccgctattaaagatatagcagaaagaaat

************************************************************

baochun2 aatagaaaaagagctccagtataaagatcttcattagtacgtaaaccaattgtataccac

baochun1 aatagaaaaagagctccagtataaagatcttcattagtacgtaaaccaattgtataccac

************************************************************

baochun2 cactgataaacaccagaatacgcgatattcactgggccaagagcaccccctcgagtaaag

baochun1 cactgataaacaccagaatacgcgatattcactgggccaagagcaccccctcgagtaaag

************************************************************

baochun2 gcttctacagccggttgaccaaaatgaggatcccaaattgcatgagcaataggccttaca

baochun1 gcttctacagccggttgaccaaaatgaggatcccaaattgcatgagcaataggccttaca

************************************************************

baochun2 tgtaaagggtcctgtacccatgactcaaaattcccttgccaagctacatgaaacagattt

baochun1 tgtaaagggtcctgtacccatgactcaaaattcccttgccaagctacatgaaacagattt

************************************************************

baochun2 ccggaagtccacaaaaaaattattgctaattgtccgaagtgagaagcaaaaatattctga

baochun1 ccggaagtccacaaaaaaattattgctaattgtccgaagtgagaagcaaaaatattctga

************************************************************

baochun2 taaagacgttcttcagtaatatcatcatgactctcgaagtcatgtgcggtagcaatacca

baochun1 taaagacgttcctcagtaatatcatcatgactctcgaagtcatgtgcggtagcaatacca

***********.************************************************

baochun2 aaccaaatacgacgagtagtggggtcctgagctaagccttggctaaaccttggaaatctt

baochun1 aaccaaatacgacgagtagtggggtcctgagctaagccttggctaaaccttggaaatctt

************************************************************

baochun2 aatgccataatgcttttcaaatcctcctagccattatcctactgcaataattcttgctaa

baochun1 aatgccataatgcttttcaaatcctcctagccattatcctactgcaataattcttgctaa

************************************************************

baochun2 gaagaatgcccatgttgtggcaattccacccagaaggtaatgagctactcctacagcacg

baochun1 gaagaatgcccatgttgtggcaattccacccagaaggtaatgagctactcctacagcacg

************************************************************

baochun2 tccttgtacaatgctcaaggctctaggctgagtagcaggagcaacttttaatttattatg

baochun1 tccttgtacaatgctcaaggctctaggctgagtagcaggagcaacttttaatttattatg

************************************************************

baochun2 agcccaaacgatggattcaataagttcttgccaataaccacgtccactgaatagaaacat

baochun1 agcccaaacgatggattcaataagttcttgccaataaccacgtccactgaatagaaacat

************************************************************

baochun2 taaactaaaagcccatacaaaatgagcacctaggaaaaaaaggccatatgctgataatga

baochun1 taaactaaaagcccatacaaaatgagcacctaggaaaaaaaggccatatgctgataatga

************************************************************

baochun2 agaaccataagactgaattacttgggatgcctgtgcccataagaaatctcggagccaccc

baochun1 agaaccataagactgaattacttgggatgcctgtgcccataagaaatctcggagccaccc

************************************************************

baochun2 attaatagtaatggaactctgtgcaaagtttcctcctgtaatatgagttactattccctg

baochun1 attaatagtaatggaactctgtgcaaagtttcctcctgtaatatgagttactattccctg

************************************************************

baochun2 atcacttatactaccccaaacatctgactgcattttccaactgaaatggaatattactac

baochun1 atcacttatactaccccaaacatctgactgcattttccaactgaaatggaatattactac

************************************************************

baochun2 ggaaattgcattgtacatccaaaatagtcctaagaagacatgatcccaagccgatacctg

baochun1 ggaaattgcattgtacatccaaaatagtcctaagaagacatgatcccaagccgatacctg

************************************************************

baochun2 acatgttccacctcttccaggtccatcacaaggaaaacgaaaacccagatttgctttatc

baochun1 acatgttccacctcttccaggtccatcacaaggaaaacgaaaacccagatttgctttatc

************************************************************

baochun2 cggtatcaaacgggagctacgagcaaatagaacgcctttcaggagtatcaataccgttac

baochun1 cggtatcaaacgggagctacgagcaaatagaacgcctttcaggagtatcaataccgtcac

*********************************************************.**

baochun2 atgaatcgtaaatgcatgaatgtggtggaccaaaaaatccgcggttcctaatggaatagg

baochun1 atgaatcgtaaatgcatgaatgtggtggaccaaaaaatccgcggttcctaatggaatagg

************************************************************

baochun2 taacaaagctactttgccgcccactgctattaaatcaccacctccccaagttaaactggt

baochun1 taacaaagctactttgccgcccactgctattaaatcaccacctccccaagttaaactggt

************************************************************

baochun2 gcttgttgttgcaccaggagccgttgcgccaggcgccaaagcatgggtattttggatcca

baochun1 gcttgttgttgcaccaggagccgttgcgccaggcgccaaagcatgggtattttggatcca

************************************************************

baochun2 ttgagcaaagacgggttgtaattggatagcggtatctgaaaacatatcttggggacgccc

baochun1 ttgagcaaagacgggttgtaattggatagcggtatctgaaaacatatcttggggacgccc

************************************************************

baochun2 taaagcgctcatggtatcattatgaatatacaaaccaaaactgtgaaatcctagaaaaat

baochun1 taaagcgctcatggtatcattatgaatatacaaaccaaaactgtgaaatcctagaaaaat

************************************************************

baochun2 acatgcccagttgagatgtgatatgattgcatcacgatgtctaaggacacgatctaatag

baochun1 acatgcccagttgagatgtgatatgattgcatcacgatgtctaaggacacgatctaatag

************************************************************

baochun2 atcgttgtatcgagtagttggatcatagtctcttaccataaaaatggctgcatgcgcagc

baochun1 atcgttgtatcgagtagttggatcatagtctcttaccataaaaatggctgcatgcgcagc

************************************************************

baochun2 agcaccaactattagaaatccaccaatccacatatgatgtgtaaacaatgacagttgtgt

baochun1 agcaccaactattagaaatccaccaatccacatatgatgtgtaaacaatgacagttgtgt

************************************************************

baochun2 accatagtcagtagctagatatggataaggcggcatggaatacatatggtgagctacaac

baochun1 accatagtcagtagctagatatggataaggcggcatggaatacatatggtgagctacaac

************************************************************

baochun2 aatggttaaagagcctaacatagctaggttaagagataattgagcatgccatgacgttgt

baochun1 aatggttaaagagcctaacatagctaggttaagagataattgagcatgccatgacgttgt

************************************************************

baochun2 taggatctcatataggcctttatgaccctggcctgtaaatggacctttatgggcttctaa

baochun1 taggatctcatataggcctttatgaccctggcctgtaaatggacctttatgggcttctaa

************************************************************

baochun2 aatctcttttaggccatgaccaataccccagttcgttctatacatgtgacccgctaccag

baochun1 aatctcttttaggccatgaccaataccccagttcgttctatacatgtgacccgctaccag

************************************************************

baochun2 gaaaagaattgcaatagctaaatgatggtgtgcaatatcagtcaaccatagacccccagt

baochun1 gaaaagaattgcaatagctaaatgatggtgtgcaatatcagtcaaccatagacccccagt

************************************************************

baochun2 tactggatctaatcctccgcgaaaagtaagaaagtctgcatattttgaccaattcaaggt

baochun1 tactggatctaatcctccgcgaaaagtaagaaagtctgcatattttgaccaattcaaggt

************************************************************

baochun2 gaagaatggggttgctccctcagcaaaacttggataaagttgagccaaaagatcccgatt

baochun1 gaagaatggggttgctccctcagcaaaacttggataaagttgagccaaaagatcccgatt

************************************************************

baochun2 caagataaattcatgaggaagtggtatctctttaggatctactccagcgtttagaaattg

baochun1 caagataaattcatgaggaagtggtatctctttaggatctactccagcgtttagaaattg

************************************************************

baochun2 gttaatgggtaaagatacatgtacttgatggcctgcccaagaaagagacccaagtcctag

baochun1 gttaatgggtaaagatacatgtacttgatggcctgcccaagaaagagacccaagtcctag

************************************************************

baochun2 taaccccgctaaatggtggttcaacatagattccacatcttgaaaccaagctaattttgg

baochun1 taaccccgctaaatggtggtttaacatagattccacatcttgaaaccaagctaattttgg

*********************.**************************************

baochun2 agcagctttatgataatggaaccaaccagcaaaaagcattaacgctgcaaagatcaatgc

baochun1 agcagctttatgataatggaaccaaccagcaaaaagcattaacgctgcaaagatcaatgc

************************************************************

baochun2 cccaattgcggtacaatagagttgtaattcgctagttattccagatgctcgccaaatctg

baochun1 cccaattgcggtacaatagagttgtaattcgctagttattccagatgctcgccaaatctg

************************************************************

baochun2 aaaaaaaccagaggttatttgtattcctcggaaaccaccgcccacatcaccattcaagat

baochun1 aaaaaaaccagaggttatttgtattcctcggaaaccaccgcccacatcaccattcaagat

************************************************************

baochun2 ttcttgacccactattggccaaaccacttgggcactaggcccaatgtgagtaggatcgct

baochun1 ttcttgacccactattggccaaaccacttgggcactaggcccaatgtgagtaggatcgct

************************************************************

baochun2 taaccatgcttcatagttggaaaaacgagcaccatggaaatacatgccactcaaccaaag

baochun1 taaccatgcttcatagttggaaaaacgagcaccatggaaatacatgccactcaaccaaag

************************************************************

baochun2 aaagatgatggagagttgcccaaaatgggcactaaatacttttcgagaaatctcctccaa

baochun1 aaagatgatggagagttgcccaaaatgggcactaaatacttttcgagaaatctcctccaa

************************************************************

baochun2 atcaccagtatggctatcgaaatcgtgagcatcggcatgtaggttccagatccaagtggt

baochun1 atcaccagtatggctatcgaaatcgtgagcatcggcatgtaggttccagatccaagtggt

************************************************************

baochun2 agtctcaggacctttagctattgttcttgagaaatgacccggtttggcccattcctcaaa

baochun1 agtctcaggacctttagctattgttcttgagaaatgacccggtttggcccattcctcaaa

************************************************************

baochun2 agaagtttttatgggatccctatctaccaaaatttttacttctggttccggcgaacgaat

baochun1 agaagtttttatgggatccctatctaccaaaatttttacttctggttccggcgaacgaat

************************************************************

baochun2 aatcattgagtcctcctctttccggacaacacatacaaagagacttgccaactatgaacc

baochun1 aatcattgagtcctcctctttccggacaacacatacaaagagacttgccaactatgaacc

************************************************************

baochun2 tctgagatatttttttttctattttatattctatct-atttcctttagttattcactaga

baochun1 tctgagata-ttttttttctattttatattctatctaatttcctttagttattcactaga

********* ************************** ***********************

baochun2 gtaattatgatttgaaagtcaatccggggcaagtgttcgaatctattatgacatagccct

baochun1 gcaattatgatttgaaagtcaatccggggcaagtgttcgaatctattatgacatagccct

*.**********************************************************

baochun2 gaggcgctcaacggaccttttttttactttaaaacttttttgagggcgttggattgatcc

baochun1 gaggcgctcaacggaccttttttttactttaaaacttttttgagggcgttggattgatcc

************************************************************

baochun2 aaaactttttttctgcaacctatagtgtgtattcatactgcacttagaagtacttgctta

baochun1 aaaac-ttttttctgcaacctatagtgtgtattcatactgcacttagaagtacttgctta

***** ******************************************************

baochun2 taaggatatacgttatattttactatagaacatagactaaataagagacatcccaatata

baochun1 taaggatatacgttatattttactatagaacatagactaagtaagagacatcccaatata

****************************************.*******************

baochun2 ttatcgatatttcgtctcttttttattatattagttttaacattatcaatagaaaaaaaa

baochun1 ttatcgatatttcgtctcttttttattatattagttttaacattatcaatagaaaaaaaa

************************************************************

baochun2 gaaagatattttgactccatctgtctatcctttatccctatgaaataccagacgaatatg

baochun1 gaaagatattttgactccatctgtctatcctttatccctatgaaataccagacgaatatg

************************************************************

baochun2 gaatgatcttagaagaaatataatgaaattctttataataaataaataaacagagttttc

baochun1 gaatgatcttagaagagatataatgaaattctttataataaataaataaacagagtcttc

****************.***************************************.***

baochun2 ttttattcgaaacgtctagtgatctttaaccaattatgtgcttcaatataattacccgga

baochun1 ttttattcgaaacgtctagtgatctttaaccaattatgtgcttcaatataattacccgga

************************************************************

baochun2 gtaagtgctatagcttgcttccaatactcagcggcttgatcgaaccaagcttctgcaatt

baochun1 gtaagtgctatagcttgcttccaatactcagcggcttgatcgaaccaagcttctgcaatt

************************************************************

baochun2 tcagaatttccctgtcgaatggcctgttctccccggtcggaataggctggtaaattcctt

baochun1 tcagaatctccctgtcgaatggcctgttctccccggtcggaataggctggtaaattcctt

*******.****************************************************

baochun2 cccttagaaccgtacttgagagtttcctacctcatacggctcaaccctcaatcttttttt

baochun1 cccttagaaccgtacttgagagtttcctacctcatacggctcaaccctcaatcttttttt

************************************************************

baochun2 tggtatcccatttgaatctaccatatctaagtgaatgagattcctcatagatctatccca

baochun1 tggtatcccatttgaatctaccatatctaagtgaatgagattcctcatagatctatccca

************************************************************

baochun2 ctgcttcgggctaaccaaaagaggttaattacataagtttcaaactttaatttagattaa

baochun1 ctgcttcgggctaaccaaaagaggttaattacataagtttcaaactttaatttagattaa

************************************************************

baochun2 taatcggtttttgttttatcttttctcccaccttcagaataataaaacataggcatttca

baochun1 taatcggttttcgttttatcttttctcccaccttcagaataataaaacataggcatttca

***********.************************************************

baochun2 cctatcgttagaattttctgaaaggtaactatctcggtttcatatcgaaattgatataga

baochun1 cctatcgttagaattttctgaaaggtaactatctcggtttcatatcaaaattgatataga

**********************************************.*************

baochun2 atctttgaaaaagactttccttcagaagaaaagacttactatctttgggatctgatccta

baochun1 atctttgaaaaagactttccttcagaagaaaagacttactatctttgggatctgatccta

************************************************************

baochun2 caccgctgcccaataccttagtggatcgactttattacataagttgattcctaactttta

baochun1 caccgctgcccaataccttagtggatcgactttattacataagttgattcctaactttta

************************************************************

baochun2 tctgacatcatgaaaaagtaagcagttattattgtctcggcctaaaacctcgctaattga

baochun1 tctgacatcatgaaaaagtaagcagttattattgtctcggcctaaaacctcgctaattga

************************************************************

baochun2 tctttacggtgcttcctttatcaatttgatccttttatccatagaataaaatagctaggc

baochun1 tctttacggtgcttcctttatcaatttgatccttttatccatagaataaaatagctaggc

************************************************************

baochun2 atatttatttctttagatttggcttctataaagtttctttcttttctacagctgataaaa

baochun1 atatttatttcttcagatttggcttctataaagtttctttcttttctacagctgataaaa

*************.**********************************************

baochun2 atcgttgttttagacaatacatatgtagaaagcctattttttttctagtattttcctttt

baochun1 atcgttgttttagacaatacatatgtagaaagcctattttttttctagtattttcctttt

************************************************************

baochun2 tctttctatagtggggatagtcgcacgtaatgacagatcacggccatattattaaaagct

baochun1 tctttctatagtggggatagtcgcacgtaatgacagatcacggccatattattaaaagct

************************************************************

baochun2 tgtggtaagaatggatttcgttctaatgctcgaaaataatattccaaagctttcgtatgt

baochun1 tgtggtaagaatggatttcgttctaatgctcgaaaataatattccaaagctttcgtatgt

************************************************************

baochun2 tctccattacttgtgtggataagaccgatgttataaagtatataacttcgatcataggga

baochun1 tctccattacttgtgtggataagaccgatgttataaagtatataacttcgatcataggga

************************************************************

baochun2 tcaatttctaatcgcatagcttcgtaataattctgtaaagcttccgcataatttccttcg

baochun1 tcaatttctaatcgcatagcttcgtaataattctgtaaagcttccgcataatttccttcg

************************************************************

baochun2 gattgggctgacatccgttacggtcgtcattcaagtcaaagaatccccgttacagaaccg

baochun1 gattgggctgacatccgttacggtcgtcattcaagtcaaagaatccccgttacagaaccg

************************************************************

baochun2 tacgtgagatgttcatctcatacggctcctcccttatgtgcctaataagaatagaataat

baochun1 tacgtgagatgttcatctcatacggctcctcccttatgtgcctaata-----agaataat

*********************************************** ********

baochun2 acatggaataaaaaaagattaaactattctcattattaactgagtggagctagtgttttt

baochun1 acatggaataaaaaaagattaaactattctcattattaactgagtggagctagtgttttt

************************************************************

baochun2 acatgaaatctctagccaaccttcctgcaagagatcttttcttaacctcaagcgtattgt

baochun1 acatgaaatctctagccaaccttcctgcaagagattttttcttaacctcaagcgtattgt

***********************************.************************

baochun2 taggatatagaaatgataactcaaccatttttttgttctcaacgcctcctaatttataga

baochun1 taggatatagaaatgataactcaaccatttttttgttctcaacgcctcctaatttataga

************************************************************

baochun2 aattagtcacttcaacagccttcgatggttatacgggtatacaaaatacgaacgagatgg

baochun1 aattagtcacttcaacagccttcgatggttatacgggtatacaaaatacgaacgagatgg

************************************************************

baochun2 atgtttgttgtcccaaccattcttgttagtcccgagcccgagacagacaagggtaattta

baochun1 atgtttgttgtcccaaccattcttgttagtcccgagcccgagacagacaagggtaattta

************************************************************

baochun2 taacaaagttttcgtgttgttgatttctaggtgtagtgcttcttcctctatgccgcctag

baochun1 taacaaagttttcgtgttgttgatttctaggtgtagtgcttcttcctctatgccgcctag

************************************************************

baochun2 tggtactagtagagtagtgttgatctgtaatatgtaatacagaacctctaggtgtatcct

baochun1 tggtactagtagagtagtgttgatctgtaatatgtaatacagaacctctaggtgtatcct

************************************************************

baochun2 ttcgcttaatactataatcgacagttgaagtatagcatctaaggctgcattaatcgagga

baochun1 ttcgcttaatactataatcgacagttgaagtatagcatctaaggctgcattaatcgagga

************************************************************

baochun2 tacacgacagaaggaattgttttatttccaaacttcaccttcaagaagcgtagatttttt

baochun1 tacacgacagaaggaattgttttatttccaaacttcaccttcaagaagcgtaga--tttt

****************************************************** ****

baochun2 tttttcaaaaattttctttttatatcgaatcatgtgtctttctcgtaagactgagagcag

baochun1 tttttcaaaaattttctttttatatcgaatcatgtgtctttctcgtaagactgagagcag

************************************************************

baochun2 taacaaaaaataatcaaatcacaccatctctgtaataactaaatgcctctttttctcctg

baochun1 taacaaaaaataatcaaatcacaccatctctgtaataactaaatgcctctttttctcctg

************************************************************

baochun2 aagttgtcggaataattcgtaataagatattggctacaattgaaaaggtcttatcaataa

baochun1 aagttgtcggaattattcgtaataagatattggctacaattgaaaaggtcttatcaataa

************* **********************************************

baochun2 aatttccatttattcgtgatctaggcataggtagcaatccattctatcattcttctgatt

baochun1 aatttccatttattcgtgatctaggcataggtagcaatccattctatcattcttctgatt

************************************************************

baochun2 ccttttcgtgggaaaattatcccacaaagaaaagaattttaattatacagtacaaaataa

baochun1 ccttttcgtgggaaaatgatcccacaaagaaaagaattttaattatacagtacaaaataa

***************** ******************************************

baochun2 cataaaaacagattcatttcaaaaaataaaagaaagggggctttctaccccaattgttcg

baochun1 cataaaaacagattcatttcaaaaaataaaagaaagggggctttctaccccaattattcg

*******************************************************.****

baochun2 ttaagaatcgctaagaaatatttgaacctcatttcattagatttcaagatttcacccaaa

baochun1 ttaggaatcgctaagaaatatttgaacctcatttcattagatttcaagatttcacccaaa

***.********************************************************

baochun2 tctagtaatataaaaatgaaagcaactaattcgatttcagtaaagttagagattagaata

baochun1 tctagtaatatatcaatgaaagcaactaattcgatttcagtaaagttagagattagaata

************ **********************************************

baochun2 acttgataagtttggattgagttaggattcaaagaagaaaaaaaggatcgactaatcatt

baochun1 acttgataagtttggattgagttaggattcaaagaagaaaaaaaggatcgactaatcatt

************************************************************

baochun2 ctatgatgaaaaaagaataaccgtctcttttgtgttgtgtgcatcgcgggtatacgctat

baochun1 ctatgatgaaaaaagaataaccgtctcttttgtgttgtgtgcatcgcgggtatacgctat

************************************************************

baochun2 aaaatcaaaatattcacattcctagaatgttttcaaaatttgtaatagatccacgtggta

baochun1 aaaatcaaaatattaacattcctagaatgttttcaaaatttgtaatagatccacgtggta

************** *********************************************

baochun2 aaggatttgttggtccgaactttactttaagactggaaacgaaaaaagtttgataatttt

baochun1 aaggatttgttggtccgaactttactttaagactggaaacgaaaaaagtttgataatttt

************************************************************

baochun2 tatggtatgatgaaatcatagtctaaatccaattatgatcgaaaggtatccattaaccat

baochun1 tatggtatgatgaaatcatagtctaaatccaattatgatcgaaaggtatccattaaccat

************************************************************

baochun2 agtctacaaaatctagatccattaataataattggattccgtctaattcattgcattgat

baochun1 agtctacaaaatctagatccattaataataattggattccgtctaattcattgcattgat

************************************************************

baochun2 ttaatctagtataaatatcagaataaagaaaccagcatcgtcttaccaaatagaaataga

baochun1 ttaatctagtataaatatcagaataaagaaaccagcatcgtcttaccaaatataaataga

**************************************************** *******

baochun2 tactttttaccttttcacctaacccttttttcacaataaaaatttttttatttattcccg

baochun1 tactttttaccttttcacctaacccttttttcacaataaaaatttttctatttattcccg

***********************************************.************

baochun2 aagactcgaagttcttttcagaatcgattggtcgcacctatccaataattaaatatgact

baochun1 aagactcgaagttcttttcagaatcgattggtcgcacctatccaataattaaatatgact

************************************************************

baochun2 cgctattcattcgggttttgggtcataatcattgtgtaggagagatggccgagtggttca

baochun1 cgctattcattcgggttttgggtcataatcattgtgtaggagagatggccgagtggttca

************************************************************

baochun2 aggcgtagcattggaactgctatgtaagcttttgtttaccgagggttcgaatccctctct

baochun1 aggcgtagcattggaactgctatgtaagcttttgtttaccgagggttcgaatccctctct

************************************************************

baochun2 ttccgtacctttcctgaatgggctgatattaccgaccctaatgtatcaaatagcaagaga

baochun1 ttccgtacctttcctgaatgggctgatattaccgaccctaatgtatcaaatagcaagaga

************************************************************

baochun2 taccatatatttgatatttgaacagttaaatctttctttgatatatattatctatttcac

baochun1 taccatatatttgatatttgaacagttaaatctttctttgatatatattctctatttcac

************************************************* **********

baochun2 atcgctgtggcatggaaaataggaaaactagaaagagtagacaaggaagtaaaatattct

baochun1 atcgctgtggcatggaaaataggaaaactagaaagagtagacaaggaagtaaaatattct

************************************************************

baochun2 attatacatacgtgggagaaaatctgagtcaaagctttattgatcttaactttaggtgaa

baochun1 attatacatacgtgggagaaaatctgagtcaaagctttattgatcttaactttaggtgaa

************************************************************

baochun2 ttgactttgctgaaaggtaccaaaattgcccgaacgcttattatgttagttaggtttaag

baochun1 ttgactttgctgaaaggtaccaaaattgcccgaacgcttattatgttagttaggtttaag

************************************************************

baochun2 tctgacgagaataatattctacgactagcaactcatttattttcaaaccgacccatttac

baochun1 tctgacgagaataatattctacgactagcaactcatttattttcaaaccgacccatttac

************************************************************

baochun2 tatctattatttgattgaccaatcctttatattgaaatgggtaaagagtcaaatgttttg

baochun1 tatctattatttgattgaccaatcctttatattgaaatgggtaaagagtcaaatgttttg

************************************************************

baochun2 gcaattcctcctgaggggaggaatcaagagaattctgaatcagagctctggatttttgtt

baochun1 gcaattcctcctgaggggaggaatcaagagaattctgaatcagagctctggatttttgtt

************************************************************

baochun2 catcccttgctgtaataatatctcggggtttacaacgataacttggtatatctactatac

baochun1 catcccttgctgtaataatatctcggggtttgcaacgataacttggtatatctactatac

*******************************.****************************

baochun2 gaccattaacgacaatatgtcgatggttaactaattggcgagctccgggaatagttgaag

baochun1 gaccattaacgacaatatgtcgatggttaactaattggcgagctccgggaatagttgaag

************************************************************

baochun2 ccatacccaatcgaaaaaggatgttatccaaacgcatttcaagtaattgtaataaaacct

baochun1 ccatacccaatcgaaaaaggatgttatccaaacgcatttcaagtaattgtaataaaacct

************************************************************

baochun2 gacctgttgaccccttggcttttccggcaatacgaacatatttaagtaattgtcgttctg

baochun1 gacctgttgaccccttggcttttccggcaatacgaacatatttaagtaattgtcgttctg

************************************************************

baochun2 taagaccataatgaaaacgcaatttttgtttttcttctagacgaatacgatattgagatt

baochun1 taagaccataatgaaaacgcaatttttgtttttcttctagacgaatacgatattgagatt

************************************************************

baochun2 ttttcccggaacgcaattggtttctaagatcatttacggccctgggccttttattcgtaa

baochun1 ttttcccggaacgcgattggtttctaagatcatttacggccctgggccttttattcgtaa

**************.*********************************************

baochun2 gtcctggtaaagctcccaggcgacgtatttttttgaaacgaggtcctcggtaacgcgaca

baochun1 gtcctggtaaagctcccaggcgacgtatttttttgaaacgaggtcctcggtaacgtgaca

*******************************************************.****

baochun2 taaaaactccttgattcttgatttatatttatttgaaatttcattttacagaataaatct

baochun1 taaaaactccttgattcttgatttatatttatttgaaatttcattttacagaataaatct

************************************************************

baochun2 aaaataaaactgaactaaatgaaatgaaatttacagaagtattgtactactaaagataaa

baochun1 aaaataaaactgaactaaatgaaatgaaatttacagaagtattgtactac-----ataaa

************************************************** *****

baochun2 gaataatgagatgaat----tatatatatataaatatccaaaactgcttctattattata

baochun1 gaataatgagatgaattatatatatatatataaatatccaaaactgcttctattattata

**************** ****************************************

baochun2 tagaga--------atatagaa------------tttcgtgacatagttggacgttccta

baochun1 tagagaaatatattatatagagaaatagaacacttttcgtgacatagttggacgttccta

****** *******. **************************

baochun2 taacacaattaatatactaaatcaagaatttttgaaaaaaaaaagggggagcagtttttc

baochun1 taacacaattaatatactaaatcaagaatttttg--aaaaaaaagggggggcagtttttc

********************************** *************.**********

baochun2 aatattttttattttcaaagggacattatcaatcatggaaaaaatcgaataaatagcgcg

baochun1 aatattttttattttcaaagggacattatcaatcatggaaaaaatcgaataaatagcgcg

************************************************************

baochun2 aaaagccggctatcggaatcgaaccgatgaccatcgcattacaaatgcgatgctctaacc

baochun1 aaaagccggctatcggaatcgaaccgatgaccatcgcattacaaatgcgatgctctaacc

************************************************************

baochun2 tctgagctaagcaggcggatctcatagaatttttacatgaataagaagttcataaactat

baochun1 tctgagctaagcaggcggatctcatagaatttttacatgaataagaagttcataaactat

************************************************************

baochun2 tggaatcttagctattaactattcgatttctattattttaattcttagctatttaattcc

baochun1 tggaatcttagctattaactattcgatttctattattttaattcttagctatttaattcg

***********************************************************

baochun2 gaattaaattaatatcaatatagaaaaaactataatttcaactaaatcttcatatcgaat

baochun1 gaattaaattaatatcaatatagaaaaaactataatttcaactaaatcttcatatcgaat

************************************************************

baochun2 attatagaacataacaattaatatagcgatacgctatattaggatagaaaattttattta

baochun1 attatagaacataacaattaatatagcgatacgctatattaggatagaaaattttattta

************************************************************

baochun2 aattcttcacaagaatttattagattctaaatttaaatgcatttgaaattcgttttatta

baochun1 aattcttcacaagaatttattagattctaaatttaaatgcatttgaaattcgttttatta

************************************************************

baochun2 cacttagctcttttcttccatatcatagatttcgaattctaattgcataatagatttaat

baochun1 cacttagctcttttcttccatatcatagatttcgaattctaattgcataatagatttaat

************************************************************

baochun2 ctaagttactaattatttaaatttgttaattaatgaactaattcattataacgaataaga

baochun1 ctaagttactaattatttaaatttgttaattaatgaactaattcattataacgaataaga

************************************************************

baochun2 cattcttccgctttcattcagacagatagaagttacgaaaaaaaaagattcgaccgttca

baochun1 cattcttccgctttcattcagacagatagaagttacg-aaaaaaaagattcgaccgttca

************************************* **********************

baochun2 agtattcaaaattatatgacaaaaaggataaaagtagatatatatatgtagtatatatcc

baochun1 agtattcaaaattatatgacaaaaaggataaaagtagatatatatatgtagtatatatcc

************************************************************

baochun2 atctataattgaattgtggatacagaaatgatagaatcttttttgattgtttgattgaac

baochun1 atctat-attgaattgtggatacagaaatgatagaatcttttttgattgtttgattgaac

****** *****************************************************

baochun2 caatatagggttctcctcgaatattagatggcctactatatatatatagtagatgaaaga

baochun1 taatatagggttctcctcgaatattagatggcctac--tatatatatagtagatgaaaga

.*********************************** **********************

baochun2 aaataggcaagaaatccaagacgagaaaacacctttcgagatagggattggtatcgaatt

baochun1 aaataggcaagaaatccaagacgagaaaacacctttcgagatagggattggtatcgaatt

************************************************************

baochun2 cattcgacgagtccagtataaaggaaaaacgaaaaagggaacgacatcacaatgagatcc

baochun1 cattcgacgagtccagtataaaggaaaaacgaaaaagggaacgacatcacaatgagatcc

************************************************************

baochun2 taatctcaaatcaaaagggggatatggcgaaattggtagacgctacggacttaattggat

baochun1 taatctcaaatcaaaagggggatatggcgaaattggtagacgctacggacttaattggat

************************************************************

baochun2 tgagccttagtatggaaacctactaagtgagaactttcaaattcagagaagccctggaat

baochun1 tgagccttagtatggaaacctactaagtgagaactttcaaattcagagaagccctggaat

************************************************************

baochun2 taataaaaatgggtaatcctgagccaaatcctcgttttcgaaaacaaaggttaaaggaaa

baochun1 taataaaaatgggtaatcctgagccaaatcctcgttttcgaaaacaaaggttaaaggaaa

************************************************************

baochun2 ataaaaaagggggataggtgcagagactcaatggaagatgttctaacaaatggagttggt

baochun1 ataaaaaagggggataggtgcagagactcaatggaagatgttctaacaaatggagttggt

************************************************************

baochun2 cgcgttgatagaggaatcctttcatgaaaatttcagaaaggaaagtatgaaagataaatg

baochun1 cgcgttggtagaggaatcctttcatgaaaatttcagaaaggaaagtatgaaagataaatg

*******.****************************************************

baochun2 tatatatatacgcatatgtactgacaatactatactatatcaaaataaaatgtttatttt

baochun1 tatatatatacgcatatgtactgacaatactatactatatcaaaataaaatgtttatttt

************************************************************

baochun2 tctatgaaaaaaaatagaagaattgttgcgaatcgattctacattcgacaatgactaaag

baochun1 tctatg-aaaaaaatagaagaattgttgcgaatcgattctacattccacaatgactaaag

****** *************************************** *************

baochun2 aatttcatattaattgatcaaagcatttactccattccatagtctgatagatcttgtgaa

baochun1 aatttcatattaattgatcaaagcatttactccattccatagtctgatagatcttgtgaa

************************************************************

baochun2 taactgattaatcaggcgagaataaagatagagtcccattctacatgtcaatacagacaa

baochun1 taactgattaatcaggcgagaataaagatagagtcccattctacatgtcaatacagacaa

************************************************************

baochun2 caataatgcaatttatagtacgaggaaaatccgtcgactttagaaatcgtgagggttcaa

baochun1 caataatgcaatttatagtacgaggaaaatccgtcgactttagaaatcgtgagggttcaa

************************************************************

baochun2 gtccctctatccccaaaagtttatttgactccctaactatatatttttcgttagcagttc

baochun1 gtccctctatccccaaaagtttatttgactccctaactatatatttttcgttagcagttc

************************************************************

baochun2 gaaattcggtatctttctgattcactttactctttgacaaatggatccgggtagaaatgc

baochun1 gaaattcggtatctttctgattcactttactctttgacaaatggatccgggtagaaatgc

************************************************************

baochun2 tttctcttattatcagttttgtggtattatgctatttaatacatgtacaaacgaacatct

baochun1 tttctcttattatcagttttgtggtattatgctatttaatacatgtacaaacgaacatct

************************************************************

baochun2 ttgagcaaagaatccccatgggaatgattcacggtccatatcattattcgtactgaaact

baochun1 ttgagcaaagaatccccatgggaatgattcacggtccatatcattattcgtactgaaact

************************************************************

baochun2 tacacaattttccttttttt--tcaaaatccaagaaattacagggcctgcataagacttt

baochun1 tacacaattttccttttttttttcaaaatccaagaaattacagggcctgcataagacttt

******************** **************************************

baochun2 atttaataatacctttttatttttaattgacatagacccgagtcgtctagtaaaatgagg

baochun1 atttaagaatacctttttatttttaattgacatagacccaagtcgtctagtaaaatgagg

****** ********************************.********************

baochun2 atgatgcatcgggagcggtcgggatagctcagctggtagagcagaggactgaaaatcctc

baochun1 atgatgcatcgggagcggtcgggatagctcagctggtagagcagaggactgaaaatcctc

************************************************************

baochun2 gtgtcaccagttcaaatctggttcctggcacattattcatttggataaggatctattcga

baochun1 gtgtcaccagttcaaatctggttcctggcacattattcatttggataaggatctattcga

************************************************************

baochun2 caaattaattgatatgggtatgggttaacatccatatttagtaatgggctagattagaat

baochun1 caaattaattgatatgggtatgggttaacatccatatttagtaatgggctagattagaat

************************************************************

baochun2 ctatattttgatccatctaagtgcttagagatataccccatctctaaaatagatgggtaa

baochun1 ctatattttgatccatctaagtgcttagagatataccccatctctaaaatagatgggtaa

************************************************************

baochun2 ataatattctttt---------------------------atataaagaataaagtatat

baochun1 ataatattcttttatatcaaagaataaagtatataaaaaaatataaagaataaagtatat

************* ********************

baochun2 aaaaaaatttactctagttcctgctccttgttatttctcttcttgttaaaaaagagggta

baochun1 aaaaaaattgactctagttcctgctccttgttatttctcttcttgttaaaaaagagggta

********* **************************************************

baochun2 aattagttggttaaaaaaccaactaatttagtctataatttagtctagaggagttgaagc

baochun1 aattagttggttaaaaaaccaactaatttagtctataatttagtctagaggagttgaagc

************************************************************

baochun2 atgggaataaacaagattaagttcaaatcaaatacaaataaaaaaattagaccccctttt

baochun1 atgggaataaacaagattaagt-----tcaaatacaaataaaaaaattagaccccctttt

********************** *********************************

baochun2 tctttgtattttggttcagattctatttcttcactccctctgacatgacttgaatttcta

baochun1 tctttgtattttggttcagattctatttcttcactccctctgacatgacttgaatttcta

************************************************************

baochun2 caacttgtttaagtcatgcgcacagcacaaagttcatgatgcagaattaattttggtact

baochun1 caacttgtttaagtcatgcgcacagcacaaagttcatgatgcagaattcattttggtact

************************************************ ***********

baochun2 aacttaacccaagacatacgaaaaaaatatgttataaattttcgaattccaatggtattg

baochun1 aacttaacccaagacatacgaaaaaaatatgttataaattttcgaattccaatggtattg

************************************************************

baochun2 ta-----ttttttttttttttagctcatccatatgataagacaaatgaaacttcaattac

baochun1 tatttttttttttttttttttagctcatccatatgataagacaaatgaaacttcaattac

** *****************************************************

baochun2 tttgtttagaatgtacaatcctttactatctggaattgtctaagtgaggattagtttctt

baochun1 tttgtttagaatgtacaatcctttactatctggaattgtctaagtgaggattagtttctt

************************************************************

baochun2 atcattcaatgagcatcttgtatttcataaaaattgggagcaatataatccttacgtaaa

baochun1 atcattcaatgagcatcctgtatttcataaaaattgggagcaatataatccttacgtaag

*****************.*****************************************.

baochun2 ggccagcctatccaactttcaggcattaagatacgtttcaagcgcggatgattatcatat

baochun1 ggccagcctatccaactttcaggcattaagatacgtttcaagcgcggatgattatcatat

************************************************************

baochun2 aagatccccaacatatcatgagattctcgttcttgaaaatccacacttttccaaacccaa

baochun1 aagatccccaacatatcatgagattctcgttcttgaaaatccacacttttccaaacccaa

************************************************************

baochun2 aaaacagatggaattcgaggatttctccttggggtaaatacttttatgcacacctcctcc

baochun1 aaaacagatggaattcgaggatttctccttggggtaaatacttttatgcacacctcctcc

************************************************************

baochun2 ggttgatctacaccatactctagtcttgtaagatgatatacactagctaatagcccgccg

baochun1 ggttgatctacaccatactctagtcttgtaagatgatatacactagctaatagcccgccg

************************************************************

baochun2 ggtgctacatcataagcacattgggaacgtagataattgtaaccatatacatataaaata

baochun1 ggtgctacatcataagcacattgggaacgtagataattgtaaccatatacatataaaata

************************************************************

baochun2 acagcaatggaatgccaatcctctggctttatttgtaaagtctctattccttggtaatca

baochun1 acagcaatggaatgccaatcctctggctttatttgtaaagtctctattccttggtaatca

************************************************************

baochun2 aaacctaaggatctatgaattatcccatgcttgactagccaagcagacaaacgaccctgc

baochun1 aaacctaaggatctatgaattatcccatgcttgattagccaagcagacaaacgaccctgc

**********************************.*************************

baochun2 atcttttttctctctcccgcatttttatttggaataagtatttatgatttacgtacaatg

baochun1 atcttttttctctctcccgcatttttatttggaataagtatttctgatttacgtacaatg

******************************************* ****************

baochun2 aaatttatgaagattgacctgcgattattctgtacaaaagaattctttctaattcactaa

baochun1 aaatttatgaagattgacctgcgattattctgtacaaaagaattctttctaattcactaa

************************************************************

baochun2 ttcgtgggaagattgggaagatactgaacttttgtatttaaaaaatatttcacgaggggt

baochun1 ttcgtgggaagattgggaagatactgaacttttgtatttaaaaaatatttcacgaggggt

************************************************************

baochun2 ctttgaactagatggtggttgataaagtaatccttgattataatttccagtattcagact

baochun1 ctttgaactagatggtggttgataaagtaatccttgattataatttccagtattcagact

************************************************************

baochun2 gcgtgcaacatgaaacttgtgattggtagtaaaacagcgattctcctgttgatacttaat

baochun1 gcgtgcaacatgaaacttgtgattggtagtaaaacagcgattctcctgttgatacttaat

************************************************************

baochun2 tcgatcttcatagatttctcgagatattttcttacgaagttttgttatagcatctataac

baochun1 tcgatcttcatagatttcttgagatattttcttacgaagttttgttatagcatctataac

*******************.****************************************

baochun2 tgcttcgggtttaggtgggcagcctggcaaataaacatctacaggaattagcttatccac

baochun1 tgcttcgggtttaggtgggcagcctggcaaataaacatctacaggaattagcttatccac

************************************************************

baochun2 tccccgaacagtactataagaatcggtactgaacatcccccctgtaattgtacatgctcc

baochun1 tccccgaacagtactataagaatcggtactgaacatcccccctgtaattgtacatgctcc

************************************************************

baochun2 catagcaataacatattttggttcgggcatttgttcatataatctcactaaagaaggacc

baochun1 catagcaataacatattttggttcgggcatttgttcatataatctcactaaagaaggacc

************************************************************

baochun2 cattttcattgttactgttccggccgttaaaatgaggtctgcttgtctaggactcgatct

baochun1 cattttcattgttactgttccggccgttaaaatgaggtctgcttgtctaggactcgatct

************************************************************

baochun2 tggtaccagtccatatcgatcaaagtcgaagcgcgagcctattagtgaagcaaattcaat

baochun1 tggtaccagtccatatcgatcaaagtcgaagcgcgagcctattagtgaagcaaattcaat

************************************************************

baochun2 gaagcaacaactagtaccatagagaaggggccataaactagaaagtcttgaccaatttga

baochun1 gaagcaacaactagtaccatagagaaggggccataaactagaaagtcttgaccaatttga

************************************************************

baochun2 aagatcatttaatgtagttgaaataactgaattttgggttggtcgattaagtaaaggaaa

baochun1 aagatcatttaatgtagttgaaataactgaattttgggttggtcgattaagtaaaggaaa

************************************************************

baochun2 ctcaatggaattcataactacctcaaaaatttttctttttattgtctgaatattcaggag

baochun1 ctcaatggaattcataactacctcaaaaatttttctttttattgtctgaatattcaggag

************************************************************

baochun2 ctaagaccattccaatgccccctttcgccatgcataaactaaaccaacaattaagataag

baochun1 ctaagaccattccaatgccccctttcgccatgcataaactaaaccaacaattaagataag

************************************************************

baochun2 cacaaaaatgaaagcttctataaatacagatagacccaatacatcaaaactcattgccca

baochun1 cacaaaaatgaaagcttctataaatacagatagacccaatacatcaaaactcattgccca

************************************************************

baochun2 tggataaagaaaaacggtttcaacatcaaaaataacaaaaactagagcaaacatataata

baochun1 tggataaagaaaaacggtttcaacatcaaaaataacaaaaactagagcaaacatataata

************************************************************

baochun2 acgaattctaaattgtaaccaagcgtcgcccattggttctatacccgattcataactaga

baochun1 acgaattctaaattgtaaccaagcgtcgcccattggttctatacccgattcataactaga

************************************************************

baochun2 aaatttctctggtccttggctaatcggggctaaaactccggaaataaaaaatgccaaaat

baochun1 aaatttctctggtccttggctaatcggggctaaaactccggaaatcaaaaatgccaaaat

********************************************* **************

baochun2 cggaataagacttgatattattagaaatgcccaaaaaatatcatattcataaagcagaaa

baochun1 cggaataagacttgatattattagaaatgcccaaaaaatatcatattcataaagcagaaa

************************************************************

baochun2 cataaacgcactcctgttaatgtggaaaatataccggattaatcgatttgaattcgaatc

baochun1 cataaacgcactcctgttaatgtggaaaatataccggattagtcgatttgaattcgaatc

*****************************************.******************

baochun2 gttaagtcgcccataactctaattcttgagtcaaaacaaagaaaaatgaattttgctcga

baochun1 gttaagtcgcccataactctaattcttgagtcaaaacaaacaaaaatgaattttgctcga

**************************************** *******************

baochun2 aacgactagtttcgtttttttac---tgttgggctcgggcaaaattcatttcacgattca

baochun1 aacgactagtttcgtttttttactgttgttgggctcgggcaaaattcatttcacgattca

*********************** **********************************

baochun2 tcgaatgaaatcctatttccattaaacctattttttgatataattttatttcaatatagt

baochun1 tcgaatgaaatcctatttccattaaacctattttttgatataattttatttcaatatagt

************************************************************

baochun2 tagtataaaaagatagtgcttttatattatacaaataaaaatttcttgttacctaggatt

baochun1 tagtataaaaagatagtgcttttatattatacaaataaaaatttcttgttacctaggatt

************************************************************

baochun2 ctctctaaagaaaagaaattcgaaatatatttttttctttctaaaaatacttagaaaatt

baochun1 ctctctaaagaaaagaaattcgaaatatatttttttctttctaaaaatgcttagaaaatt

************************************************.***********

baochun2 ggatgtttctgaatagaattaagctatgaataaaattaggcgggttttttgtctcttttt

baochun1 ggatgtttctgaatagaattaagctatgaataaaattaggcgggttttttgtctcttttt

************************************************************

baochun2 tagagtttagagatagaatagagcaagtagttaaatcaaagagaatgtctaaaaattccc

baochun1 tagagtttagagatagaatagagcaagtagttaaatcaaagagaatgtctaaaaattccc

************************************************************

baochun2 atttacgcatttagaatatattggtgctggtatattctttttattagaatacaagcggaa

baochun1 atttacgcatttagaatatattagtgctggtatattctttttattagaatacaagcggaa

**********************.*************************************

baochun2 gggtttctattgattggatattagatacggaaaaagaagacggaccccttttgtgctttg

baochun1 gggtttctattgattggatattagatacggaaaaagaagacggaccccttttgtgctttg

************************************************************

baochun2 ctagggaaggtatacgaagaaaaggttatgtaacaatggttccaatgaaactttccaaag

baochun1 ctagagaaggtatacgaagaaaaggttatgtaacaatggttccaatgaaactttccaaag

****.*******************************************************

baochun2 ctatttgtttttcaaactttagcttgtgtacaaatctagcactagatccctgtaaattac

baochun1 ctatttgtttttcaaactttagcttgtgtacaaatctagcactagatccctgtaaattac

************************************************************

baochun2 tattaaatttgattgggttggattagattgaagtttttaacggcgcgcatgtcatgattt

baochun1 tattaaatttgattgggttggattagattgaagtttttaacggcgcgcatgtcatgattt

************************************************************

baochun2 ttacttcaaaaatgcaagagtattaggtttaccaaagaaaaagagtatcactaactcttt

baochun1 ttacttcaaaaatgcaagagtattaggtttaccaaagaaaaagagtatcactaactcttt

************************************************************

baochun2 tattataaacaagaaaaatagaaaaattcctatttttatagaattcacctcatgaagaaa

baochun1 tattataaacaggaaaaatagaaaaattcctatttttatagaattcacctcatgaagaaa

***********.************************************************

baochun2 ggttggtttgtttagccgatatttttccatctatttcattggatccattggaatatgttt

baochun1 ggttggtttgtttagccgatatttttccatctatttcattggatccattggaatatgttt

************************************************************

baochun2 tgctttccgttttttttattttcgactctaaaggatccttgtgagtgagcttctaagaat

baochun1 tgctttccgttttttttattttcgactctaaaggatccttgtgagtgagcttcgaagaat

***************************************************** ******

baochun2 acttttttcgattaactgacctccgagttagttacaaacaacaaacaagaataaggaaat

baochun1 gcttttttcgattaactgacctccgagttagttacaaacaacaaacaagaataaggaaat

.***********************************************************

baochun2 aaagcatttttagaattttaatttgattttatagggtttatttatagggctatacggact

baochun1 aaagcatttttataattttcatttgattttatagggtttatttatagggctatacggact

************ ****** ****************************************

baochun2 cgaaccgtagaccttctcggtaaaacagatcaaacttattattatcaaaatgatccgaac

baochun1 cgaaccgtagaccttctcggtaaaacagatcaaacttattattatcaaaatgatccgaac

************************************************************

baochun2 tgtttcaaagacccaacatgca-tttttttgcattgggctctttcattaactgatataaa

baochun1 tgtttcaaagacccaacatgcattttttttgcattgggctctttcattaactgatataaa

********************** *************************************

baochun2 tatcagctaatccaccatattttttcttgacagaaaaataaagagatggcgccccgcgct

baochun1 tatcagctaatccgccatattttttcttgacagaaaaataaagagatggctccccgcgct

*************.************************************ *********

baochun2 ctgattcattatttgaaatttgattcagtagcactaccaaagtgtttcaaaaaagggtta

baochun1 ctgattcattatttgaaatttgattcagtagcactaccaaagtgtttcaaaaaagggtta

************************************************************

baochun2 tcttgacgtaggtctgcctctggcctagatcaacctaagttaaataaagtctctatcgct

baochun1 tcttgacgtaggtctgcctctggcctagatcaacctaagttaaataaagtctctatcgct

************************************************************

baochun2 ttgtctaaaaaaatatgaaactttatacaccttaaagttcataggacgaaaagagaaatt

baochun1 ttgtctaaaaaaatatgaaactttatacaccttaaagttcataggacgaaaagagaaatt

************************************************************

baochun2 ttggaggtccttagactcattatgcctagcattgaatagactgggtattcaccttatcaa

baochun1 ttggaggtccttatactcattatgcctagcattgaatagactgggtattcaccttatcaa

************* **********************************************

baochun2 tatctcaaatcaatgatgggttctttttggaacctaaatggaataggcacctgaatcaga

baochun1 tatctcaaatcaatgatgggttctttttggaacctaaatggaataggcacctgaatcaga

************************************************************

baochun2 ccggaccatttgtcaggctattgttccctcaaagttattggagtaagacatcgatttttc

baochun1 ccggaccatttgtcaggctattgttccctcaaagttattggagtaagacatcgatttttc

************************************************************

baochun2 cctaagatcaattcttttgattacatgatggactcccctgaaaaacattggcgcacgtgt

baochun1 cctaagatcaattcttttgattacatgctggactcccctgaaaaacattggcgcacgtgt

*************************** ********************************

baochun2 aaacgaggtgctctacctaactgagctatagcccttaattcttgtaatacctattttatc

baochun1 aaacgaggtgctctacctaactgagctatagcccttaattcttgtaatacctattttatc

************************************************************

baochun2 atgtagacaatttcttgtcaagataactattccacaatccaacatcatagaggtttgatt

baochun1 atgtagacaatttcttgtcaagataactattccacaatccaacatcatagaggtttgatt

************************************************************

baochun2 catattgcttattaagtaatatgatatttataatccatcgatgcgataggtcccgtttgc

baochun1 catattgcttattaagtaatatgatatttataatccatcgatgcgataggtcccgtttgc

************************************************************

baochun2 ttcttgctgataaatggcctacttaactcagtggttagagtattgctttcatacggcagg

baochun1 ttcttgctgataaatggcctacttaactcagtggttagagtattgctttcatacggcagg

************************************************************

baochun2 agtcattggttcaaatccaatagtaggtagaacttattagataccattgactcgggtatc

baochun1 agtcattggttcaaatccaatagtaggtagaacttattagataccattgactcgggtatc

************************************************************

baochun2 taataagttttttattttattgtgatatttttttctattttattttttcattgcattaaa

baochun1 taataagttttttattttattgtgatatttttttctattttattttttcattgcattaaa

************************************************************

baochun2 atcggattctgattgagtgtagttgattatattcaatcgacagaataaacttaaatttaa

baochun1 atcggattctgattgagtgtagttgattatattcaatcgacagaataaac------tgaa

************************************************** * **

baochun2 atttaaatatagggtggataacacagaacttctttattagtattccaacttaccaactag

baochun1 atttaaatatagggtggataacacagaacttctttattagtattccaacttaccaactag

************************************************************

baochun2 acttcttacgaaataccattgatagcttcgactcgggtcctagctcgtctgagagctaga

baochun1 acttcttacgaaatatcattgatagcttcgactcgggtcctagctcgtctgagagctaga

***************.********************************************

baochun2 tttgcctcaattagttgtcttttaccctcagctttcttcaaattagcttctgctatttca

baochun1 tttgcctcaattagttgccttttaccctcagctttcttcaaattagcttctgctatttca

*****************.******************************************

baochun2 agagtttgctgcgcttcttgtggatcaatgtcactacccttctccgcatcatttactaaa

baochun1 agagtttgctgcgcttcttgtggatcaatgtcactacccttctccgcatcatttactaaa

************************************************************

baochun2 acagtgatctcattattgcctattctagcaaaaccgcccatcagagccattgttaaccat

baochun1 acagtgatctcattattgcctattctagcaaaaccgcccatcagagccattgttaaccat

************************************************************

baochun2 tgatcgttaagccgtattcttaaaatacctatatctacagctgtggcaataggggcgtga

baochun1 tgatcgttaagccgtattcttaaaatacctatatctacagctgtggcaataggggcgtga

************************************************************

baochun2 tttggtaatacgccaatttggccactattagtggataaaattatttctttcacttcagaa

baochun1 tttggtaatacgccaatttggccactattagtggataaaattatttctttcacttcggaa

********************************************************.***

baochun2 tcccaaacaattcgattaggggtcagtacacaaagatttaaggtcatttcttcaaattgc

baochun1 tcccaaacaattcgattaggggtcagtacacaaagatttaaggtcatttcttcaaattgc

************************************************************

baochun2 tctccatttctaatttcatagccttcgcggtagcttcatcgatgttccctaccaaataaa

baochun1 tctccatttctaatttcatagccttcgcggtagcttcatcgatgttccctaccaaataaa

************************************************************

baochun2 aggcctgttcaggaagaccatctaattctccggaaaggatcaattgaaaccctcgaattg

baochun1 aggcctgttcaggaagaccatctaattctccggaaaggatcaattgaaaccctcgaattg

************************************************************

baochun2 tttctgctagaccaacatatttccctggagaaccagtaaatacttctgcaacgaaaaagg

baochun1 tttctgctagaccaacatatttccctggagaaccagtaaatacttctgcaacaaaaaagg

****************************************************.*******

baochun2 gttgtgataaaaaacgctcaatttttcttgctcttgctacggttaaacgatcctcttcag

baochun1 gttgtgataaaaaacgctcaatttttcttgctcttgctacggttaaacgatcctcttcag

************************************************************

baochun2 ataattcgtccaatccaagtatagctataatgtcctgaagttctttgtaacgttgtaaag

baochun1 ataattcgtccaatccaagtatagctataatatcctgaagttctttgtaacgttgtaaag

*******************************.****************************

baochun2 tttccttaactctttgcgcagtttcataatgttcctcaccaacgatccggggttgaagca

baochun1 tttccttaactctttgcgcagtttcataatgttcctcaccaacgatccggggttgaagca

************************************************************

baochun2 tagttgacgttgaatctaaaggatctactgctggatagatacctttggaagccaatcctc

baochun1 tagttgacgttgaatctaaaggatctactgctggatagatacctttggaagccaatcctc

************************************************************

baochun2 ttgatagtacggtagtcgcatctaaatgggcaaatgtcgtcgcaggagccgggtcggtca

baochun1 ttgatagtacggtagtcgcatctaaatgggcaaatgtcgtcgcaggagccgggtcggtca

************************************************************

baochun2 aatcgtctgccggtacataaactgcttgaatagaagttatggacccttctttagtagaag

baochun1 aatcgtctgccggtacataaactgcttgaatagaagttatggacccttctttagtagaag

************************************************************

baochun2 taattctttcttgtaacgaacccatttcagtactaagagtgggttgatagcccacagcgg

baochun1 taattctttcttgtaacgaacccatttcagtactaagagtgggttgatagcccacagcgg

************************************************************

baochun2 aaggcattctacccaataaagcagatacttcggatcccgcttggacaaaacggaagatat

baochun1 aaggcattctacccaataaagcagatacttcggatcccgcttggacaaaacggaagatat

************************************************************

baochun2 tgtcgataaatagaagtacgtcttgttcattaacatctcggaaatattccgccatagtta

baochun1 tgtcgataaatagaagtacgtcttgttcattaacatctcggaaatattccgccatagtta

************************************************************

baochun2 aggcagtcaaaccaactctcatacgagctcccggcggttcattcatttgaccataaacta

baochun1 aggcagtcaaaccaactctcatacgagctcccggcggttcattcatctgaccataaacta

**********************************************.*************

baochun2 gagcaacttttgattctgcaatattttcttcattaattaccccagattctttcatttcca

baochun1 gagcaacttttgattctgcaatattttcttcattaattaccccagattctttcatttcca

************************************************************

baochun2 tgtaaagatcgtttccttcacgagtacgctcccccactccaccaaatacggatacacctc

baochun1 tgtaaagatcgtttccttcacgagtacgctcccccactccgccaaatacggatacacctc

****************************************.*******************

baochun2 catgagctttggcaatattgttaatcaattccataataagtactgttttacccactccag

baochun1 catgagctttggcaatattgttaatcaattccataataagtactgttttacccactccag

************************************************************

baochun2 ctcccccgaataatcctattttccctccgcggcgataaggcgccaaaagatcgactacct

baochun1 ctcccccgaataatcctatttttcctccgcggcgataaggcgccaaaagatcgactacct

**********************.*************************************

baochun2 taattcctgtttcaaaaatagataatttcgtatctaactgtataaaggcaggcgcagatc

baochun1 taattcctgtttcaaaaatagataatttcgtatctaactgtataaaggcaggcgcagatc

************************************************************

baochun2 tatgaataggagatgttgtgcgagtatctacagggcctaaattatcaacaggttctccaa

baochun1 tatgaataggagatgttgtgcgagtatctacagggcctaaattatcaacaggttctccaa

************************************************************

baochun2 ggacgttgaaaattcgtccgagagttgctccaccaactggaacacttagagcagctcccg

baochun1 ggacgttgaaaattcgtccgagagttgctccaccaactggaacacttagagcagctcccg

************************************************************

baochun2 tgtcaatcacttccattcctctcgttagaccatctgtagcactcatagctacagccctaa

baochun1 tgtcaatcacttccattcctctcgttagaccatctgtagcactcatagctacagccctaa

************************************************************

baochun2 ctcgattgttccctaataattgttgtacctcacaagtaacattaattggttgaccggcag

baochun1 ctcgattgttccctaataattgttgtacctcacaagtcacattaattggttgaccggcag

************************************* **********************

baochun2 tatctcgacccttaactaccagagcattataaatattaggcatcttacccggcggaaagg

baochun1 tatctcgacccttaactaccagagcattataaatattaggcatcttacccggcggaaagg

************************************************************

baochun2 atacatctagtaccggaccaatgatttgaatgatacgtcccaggttttttttttcaagca

baochun1 atacatctagtaccgggccaatgatttgaatgatacgtcccaggttttttttttcaagca

****************.*******************************************

baochun2 cagaagtcccagaacctgaagtagtaggatttattctcataataataaataaagt-aaaa

baochun1 cagaagtcccagaacctgaagtagtaggatttattctcataataataaataaagtaaaaa

******************************************************* ****

baochun2 aaaaatatatgtcgaaattttttgtaaaaattatcgaattc-aaaaaaatatcccatagc

baochun1 aaaaatatatgtcgaaattttttgtaaaaattatcgaattcaaaaaaaatatcccatagc

***************************************** ******************

baochun2 gaagttaatcggttaattcaatatgaaatcgggattagtactccattttgttggtatcat

baochun1 gaagttaatcggttaattcaatatgaaatcgggattagtactccattttgttggtatcat

************************************************************

baochun2 tgatccgaatccaattcaatttgttacttaatttaataagataagttcattttcaaattc

baochun1 tgatccgaatccaattcaatttgttacttaatttaataagataagttcattttcaaattc

************************************************************

baochun2 aaccaaacccttttcaaaatatcaagtgaattaataagaatcttgataaagtctttacat

baochun1 aaccaaacctttttcaaaatatcaagtgaattaataagaatcttgataaagtctttacat

*********.**************************************************

baochun2 ttgtttatcattataaacaatcccatctatatgaattatctctagatttcgaacctaaac

baochun1 ttgtttatcattataaacaatcccatctatatgaattatctctagatttcgaacctaaac

************************************************************

baochun2 tctatttacgattcattatttctatctttttctttctatcaa-tttttcctagcccattc

baochun1 tctatttacgattcattatttctatctttttctttctatcaattttttcctagcccattc

****************************************** *****************

baochun2 tttcttttttttatataccttttcataggtgaagtaccccatattttcacatctaggatt

baochun1 tttcttttttttatataccttttcataggtgaagtaccccatattttcacatctaggatt

************************************************************

baochun2 tacatatacaacatataccactttcgcgaggtaatttcttattatttaggtattttgatt

baochun1 tacatatacaacatataccactttcgcgagataatttcttattatttaggtatttcgatt

******************************.************************.****

baochun2 caaaataagggagggggtcgtaaaaaaaagaattgggttgcgccatatatatgaaagagt

baochun1 caaaataagggagggggtcgtaaaaaaaagaattgggttgcgccatatatatgaaagagt

************************************************************

baochun2 atacaataatgatgtatttggcgaatcaaatacgatggtataataagagagcactcggat

baochun1 atacaataatgatgtatttggcgaatcaaatacgatggtataataagagagcactcggat

************************************************************

baochun2 tagttgataatattaatattaagttaaaattttttagaaggattcctgtgaaaagtatca

baochun1 tagttgataatattaatattaagttaaaattttttagaaggattcctgtgaaaagtatca

************************************************************

baochun2 ttaacgtttaatttatgtcgagtagaccttgttgttgtgagaattcttaaatttattaat

baochun1 ttaacgtttaatttatgtcgagtagaccttgttgttgtgagaattcttaaatttattaat

************************************************************

baochun2 tatagggagggatttatgtcaccacaaacagagactaaagcaggtgttggattcaaagct

baochun1 tatagggagggatttatgtcaccacaaacagagactaaagcaggtgttggattcaaagct

************************************************************

baochun2 ggtgttaaagattacaaattgacttattatactcctgaatatgaaaccttggatactgat

baochun1 ggtgttaaagattacaaattgacttattatactcctgaatatgaaaccttggatactgat

************************************************************

baochun2 atcttggcagcatttcgagtaactcctcaacctggagtcccgccggaagaagcaggggcc

baochun1 atcttggcagcatttcgagtaactcctcaacctggagtcccgccggaagaagcaggggcc

************************************************************

baochun2 gcggtagctgccgaatcttctactggtacatggacaaccgtgtggaccgatggacttact

baochun1 gcggtagctgccgaatcttctactggtacatggacaaccgtgtggaccgatggacttact

************************************************************

baochun2 aaccttgatcgttacaaagggcggtgctaccacatcgagcccgttgctggagaagaaaat

baochun1 aaccttgatcgttacaaagggcggtgctaccacatcgagcccgttgctggagaagaaaat

************************************************************

baochun2 caatttattgcttatgtagcttatcctttagacctttttgaagaaggttctgttactaac

baochun1 caatttattgcttatgtagcttatcctttagacctttttgaagaaggttctgttactaac

************************************************************

baochun2 atgtttacttctatcgtgggtaatgtatttggattcaaagccctgcgcgctctacgtctg

baochun1 atgtttacttctatcgtgggtaatgtatttggattcaaagccctgcgcgctctacgtctg

************************************************************

baochun2 gaagatctgcgaatcccggttgcgtatgttaaaactttccaaggaccgcctcatggtatc

baochun1 gaagatctgcgaatcccggttgcgtatgttaaaactttccaaggaccgcctcatggtatc

************************************************************

baochun2 caagttgaaagagataaattgaataagtatggtcgtcccctgttgggatgtactattaaa

baochun1 caagttgaaagagataaattgaataagtatggtcgtcctctgttgggatgtactattaaa

**************************************.*********************

baochun2 cctaaattgggattatctgctaaaaactacggcagagctgtttatgaatgtcttcgtggt

baochun1 cctaaattgggattatctgctaaaaactacggcagagctgtttatgaatgtcttcgtggt

************************************************************

baochun2 ggacttgattttaccaaagatgatgagaacataaactcccaaccatttatgcgttggcga

baochun1 ggacttgattttaccaaagatgatgagaacgtgaactcccaaccatttatgcgttggcga

******************************.*.***************************

baochun2 gaccgtttcttattttgtgccgaagctctttataaagcacaggctgaaacaggtgaaatc

baochun1 gaccgtttcttattttgtgccgaagctatttataaagcgcaggctgaaacaggtgaaatc

*************************** **********.*********************

baochun2 aaaggtcattacttgaacgctactgcaggtacatgtgaagaaatgataaaaagagctgta

baochun1 aaaggtcattacttgaacgctactgcaggtacatgtgaagaaatgataaaaagagctgta

************************************************************

baochun2 tttgccagagaattaggagttcctattgtaatgcatgactatttaacagggggattcact

baochun1 tttgccagagaattaggagttcctattgtaatgcatgactatttaacagggggattcact

************************************************************

baochun2 gcaaatactagtctggctcattattgccgagataatggcttacttcttcacatccaccgt

baochun1 gcaaatactagtctggctcattattgccgagataatggcttacttcttcacatccaccgc

***********************************************************.

baochun2 gcaatgcatgcagttattgatagacagaaaaatcatggtatacacttccgtgtactagcg

baochun1 gcaatgcatgcagttattgatagacagaaaaatcatggtatacacttccgtgtactagcg

************************************************************

baochun2 aaagccttacgtatgtctggtggagatcatattcactctggtactgtagtaggtaaactt

baochun1 aaagccttacgtatgtctggtggagatcatattcacgctggtactgtagtaggtaaactt

************************************ ***********************

baochun2 gaaggggaaagggaaatcactttgggctttgttgatttactgcgtgatgattatattgaa

baochun1 gaaggggaaagggaaatcactttgggctttgttgatttactgcgtgatgattatattgaa

************************************************************

baochun2 aaagatcgaagccgcggtatttatttcactcaagattgggtctcgctaccaggtgttctg

baochun1 aaagatcgaagccgcggtatttatttcactcaagattgggtctcgctaccaggtgttctg

************************************************************

baochun2 cccgtggcttcagggggtattcacgtttggcatatgcctgctctgaccgagatctttgga

baochun1 cccgtggcttcagggggtattcacgtttggcatatgcctgctctgaccgagatctttgga

************************************************************

baochun2 gatgattctgtactacaattcggtggcggaactttaggacacccttggggaaatgcaccg

baochun1 gatgattctgtactacaattcggtggcggaactttaggacacccttggggaaatgcaccg

************************************************************

baochun2 ggtgctgtagctaaccgagtagctctagaagcatgtgtacaagctcgtaatgagggacgt

baochun1 ggtgctgtagctaaccgagtagctctagaagcatgtgtacaagctcgtaatgagggacgt

************************************************************

baochun2 gatcttgctcgtgaaggtaatgaaattatccgtgaggcttgcaaatggagtcctgaatta

baochun1 gatcttgctcgtgaaggtaatgaaattatccgtgaggcttgcaaatggagtcctgaatta

************************************************************

baochun2 gctgctgcttgtgaggtatggaaggagatcaaatttgaattcgaagcaatggatactttg

baochun1 gctgctgcttgtgaggtatggaaggagatcaaatttgaattcgaagcaatggatactttg

************************************************************

baochun2 taattcaataattactgttcggtctgttaattgaattggaattaaactcggcccaatctt

baochun1 taattcaataattactgttcggtctgttaattgaattggaattaaactcggcccaatctt

************************************************************

baochun2 ttactaaaaggattgagccgaatacaaaaattctattgtatccacgttggctagagaatc

baochun1 ttactaaaaggattgagccgaatacaaaaattctattgtatccactttggctagagaatc

********************************************* **************

baochun2 cattttgggtagatatatacttatcaagatatacaagatttaaaattcaataaaaaataa

baochun1 cattttgggtagatatatacttatcaagatatacaagattgaaaattc-------aataa

**************************************** ******* *****

baochun2 aataagattcaaaaacacaaatatttctattcttgtattggatccacaatgaatcctctg

baochun1 aataagattcaaaaacacaaatatttctattcttgtattggatccacaatgaatcctctg

************************************************************

baochun2 gatccttaggattggtgtattcttttatatcttctagtttctctggatagaactaagtat

baochun1 gatccttaggattggtgtattcttttatatcttctagtttctctggatagaactaagtat

************************************************************

baochun2 caaaacccttttttacccatcctgtatattgtccttttcgttttgtgttgcaatcgaaac

baochun1 caaaacctttttttacccatcctgtatattgtccttttcgttttgtgttgcaatcgaaac

*******.****************************************************

baochun2 tttacttaattagtggattcgttattagacgagattttacaaaaaaaagtcct-------

baochun1 tttacttaattagtggattcgttattagacgagattttacaaaaaaagtccttattagta

***********************************************. .*.*

baochun2 tattagtagagacgaaattagattagaagagaataaatatttc-ttttttttttcaatac

baochun1 gattagtagagacgaaattagattagaagagaataaatatttctttttttttttcaatac

****************************************** ****************

baochun2 ttacactcctcattagttaataatcctagtgattcgatttctatgtttattctgatagta

baochun1 ttacactcctcattagttaataatcctagtgattcgatttctatgtttattttgatagta

***************************************************.********

baochun2 aataggatatttaaaaaaattctttttcatcgaatgactattcatttattgtattttcat

baochun1 aataggatattgaaaaaaattctttttcatcgaatgactattcatttattgtattttcat

*********** ************************************************

baochun2 gtaaataaaataggggtcaaaaaaactctatggaaaaatggcagttcaatttgattttgt

baochun1 gtaaataaaataggggtcaaaaaaactctatggaaaaatggcagttcaatttgatcttgt

*******************************************************.****

baochun2 ctaacaaggagttagagttagaacacaggtgtagattaagtaaatcaatggacagtcgta

baochun1 ctaacaaggagttagagttagaacacaggtgtagattaagtaaatcaatggacagtcgta

************************************************************

baochun2 gttttattaatgaacatattactgaaagtgaagatccaaatagaaatgataggcatcatc

baochun1 gttttattaatgaacatattactgaaagtgaagatccaaatagaaatgataggcatcatc

************************************************************

baochun2 atagttggagttatagtaacagttccacttacagtaatgttgatcatttatttggcgtca

baochun1 atagttggagttatagtaacagttccacttacagtaatgttgatcatttatttggcgtca

************************************************************

baochun2 aggacattggtaatttcatctctgatgacacttttatagttagggataggaatggggaca

baochun1 aggacattggtaatttcatctctgatgacacttttatagttagggataggaatggggaca

************************************************************

baochun2 gctattccatatattttgatattgaaaataagctttttgagattaacaaggatcattctt

baochun1 gctattccatatattttgatattgaaaataagctttttgagattaacaaggatcattctt

************************************************************

baochun2 ttctgagtgaattcgaaagacctttttctagttatccgaattctgattatctgactaatg

baochun1 ttctgagtgaactcgaaagacctttttctagttatccgaattctgattatctgaataatg

***********.****************************************** *****

baochun2 gatctaatagtgatgatccttactatgatcgttacatagatgatactcaatatagttgga

baochun1 gatctaatagtgatgatccttactatgatcgttacatagatgatactcaatatagttgga

************************************************************

baochun2 ctaatcatattaatagttgtattgacagttatcttgattctcaactacgcattaatgctt

baochun1 ctaatcatattaatagttgtattgacagttatcttgattctcaactacgcattaatgctt

************************************************************

baochun2 acgcttacattgtaagtagtagtgacaattatagtgacagttacatttatggtgaaaatc

baochun1 acgcttacattgtaagtagtagtgacaattatagtgacagttacatttatggtgaaaatc

************************************************************

baochun2 gaaatagtagtgaaagcgagagttccagtataaggaatacgggtgatttaactataagag

baochun1 gaaatagtagtgaaagcgagagttccagtataaggaatacgggtgatttaactataagag

************************************************************

baochun2 aaagttataataatctcgatgtaactcaaaaatacaggcatttgtgggttcaatgcgaaa

baochun1 aaagttataataatctcgatgtaactcaaaaatacaggcatttgtgggttcaatgcgaaa

************************************************************

baochun2 agtgttatggattaaattataagaaaattttgaagtcaaaaatgaatatttgtgaacaat

baochun1 agtgttatggattaaattataagaaaattttgaagtcaaaaatgaatatttgtgaacaat

************************************************************

baochun2 gtggatattatttgaaaatgagtagttcagatagaatcgaacttttgattgatccaggca

baochun1 gtggatattatttgaaaatgagtagttcagatagaatcgaacttttgattgatccaggca

************************************************************

baochun2 cttgggatcctatgaatgaagacatggtatctctggatcccattgaatttcattcggagg

baochun1 cttgggatcctatgaatgaagacatggtatctctggatcccattgaatttcattcggagg

************************************************************

baochun2 aggagccgtataaagatcgtattgattcttatcaaagaaagacagggttaactgaggctg

baochun1 aggagccgtataaagatcgtattgattcttatcaaagaaagacagggttaactgaggctg

************************************************************

baochun2 ttcaaacaggtataggccaactaaatggtattcccatcgcaattggggttatggattttc

baochun1 ttcaaacaggtataggccaactaaatggtattcccgtcgcaattggggttatggattttc

***********************************.************************

baochun2 agtttatggggggtagtatgggatctgtagtcggagagaaaattacccgtttggtcgagt

baochun1 agtttatggggggtagtatgggatctgtagtcggagagaaaattacccgtttggtcgagt

************************************************************

baochun2 atgctaccaaaaaatttatacctcttgttatagtgtgtgcttctgggggtgcacgtatgc

baochun1 atgctaccaaaaaatttatacctcttgttatagtgtgtgcttctgggggtgcacgtatgc

************************************************************

baochun2 aagaaggaagtttgagcttgatgcaaatggctaaaatatcttctgctttatacgattatc

baochun1 aagaaggaagtttgagcttgatgcaaatggctaaaatatcttctgctttatacgattatc

************************************************************

baochun2 aatcaaataaaaaactattttatgtaccaattcttacatctcccactacgggtggggtga

baochun1 aatcaaataaaaaactattttatgtaccaattcttacatctcccactacgggtggggtga

************************************************************

baochun2 cagctagttttggtatgttgggggatatcattattgccgaacccaacgcctacattgcat

baochun1 cagctagttttggtatgttgggggatatcattattgccgaacccaacgcctacattgcat

************************************************************

baochun2 ttgcgggtaaaagagtaattgaacaaacattgaataaaacaatacccgaagggtcacaag

baochun1 ttgcgggtaaaagagtaattgaacaaacattgaataaaacagtacccgaagggtcacaag

*****************************************.******************

baochun2 cggctgaatatttattccagaagggcttattcgatctaattgtaccacgtaatcttttaa

baochun1 cggctgaatatttattccagaagggcttattcgatctaattgtaccacgtaatcttttaa

************************************************************

baochun2 aaaacgttctgagtgagttatttcaactccacgctttctttcctttgaatcaaaattaaa

baochun1 aaaacgttctgagtgagttatttcaactccacgctttctttcctttgaatcaaaattaaa

************************************************************

baochun2 agactattccgtttaattcgttttttgtagtaaacacgtagttagcttatcggaatcaaa

baochun1 agactattccgtttaattcgttttttgtagtaaacgcgtagttagcttatcggaatcaaa

***********************************.************************

baochun2 ataaaaataagaagaacagacttttttttggtgacttaagatctataagatcgaattcta

baochun1 ataaaaataagaagaacagacttttttttggtgacttaagatctataagatcaaattcta

****************************************************.*******

baochun2 gaaagaatcaccagttgcatataatttttc---tttttttttactaatattcatgattac

baochun1 gaaagaatcaccagttgcatataatttttcttttttttttttactaatattcatgattac

****************************** ***************************

baochun2 gaatcagcaagcctctataaaagaatgaattcttgcttttgtgaaattaggcgaagttct

baochun1 gaatcagcaagcctctataaaagaataaattcttgcttttgtgaaattaggcgaagttct

**************************.*********************************

baochun2 tcttacgtatgtattatataataaattcaaatatagaaaaatacacaattatatattttc

baochun1 tcttacgtatgtattatataataaattcaaatatagaaaaatacacaattatatattttc

************************************************************

baochun2 tatatttgaattttgactaagaagtctagaaatctttcagtaatttgtaaaaaattatta

baochun1 tatatttgaattttgactaagaagtctagaaatctttcagtaatttgtaaaaaattatta

************************************************************

baochun2 aattagaagtagaagacaagaacaaacgaagaagaataaataataaactaaattttcata

baochun1 aattagaagtagaagacaagaacaaacgaagaagaataaataataaactaaattttcata

************************************************************

baochun2 catattcttcgtgtcgaaagatgaataagtccatttatttagttctacattccttgcact

baochun1 catatccttcgtgtcgaaagatgaataagtccatttatttagttctacattccttgcact

*****.******************************************************

baochun2 tattatatatactcatttagatatatacttcgatctataattacttaaaatgaaagtttc

baochun1 tattatatatactcatttagatatatacttcgatctataattacttaaaatgaaagtttc

************************************************************

baochun2 ataattaaaaaaatagtaattagaatcgcattaataaggaattttcattttataattaaa

baochun1 ataattcaaaaaatagtaattagaatcgcattaataaggaattttcattttataattaaa

****** *****************************************************

baochun2 attattacaagatatctttataacatattacaagatatctttataacaatagaaacaata

baochun1 attattacaagatatctttataacatattacaagatatctttataacaatagaaacaata

************************************************************

baochun2 taataataacaggtacaaatagtaaattaaatcgaggtattcattctatgataactttca

baochun1 taataataacaggtacaaatagtaaattaaatcgaggtattcattctatgataactttca

************************************************************

baochun2 actttccctctatttttgtgcctttagtgggcctagtatttccggccatggcaatggctt

baochun1 actttccctctatttttgtgcctttagtgggcctagtatttccggccatggcaatggctt

************************************************************

baochun2 ctttatttctttatgttcaaaaaaacaagattgtttagatctcggataggactaaatctc

baochun1 ctttatttctttatgttcaaaaaaacaagattgtttagatctcggataggactaaatctc

************************************************************

baochun2 attatct--tttttttttgaacttagataaaataaataaatagatatttatttgagtatg

baochun1 attatcttgtttttttttttacttagataaaataaataaatagatatttatttgagtatg

******* ********* ****************************************

baochun2 gtataacatggggtttctgcttaacagaaatagaacatacataaatcaaagagttcttat

baochun1 gtataacatggggtttctgcttaacagaaatagaacatacataaatcaaagagttcttat

************************************************************

baochun2 acatacagaaaatgatatatgggtaaatttattctagctattttcaactagaataaggat

baochun1 acatacagaaaatgatatatgggtaaatttattctagctattttcaactagaataaggat

************************************************************

baochun2 tggcggatgtcggaaatggaaagtctatgtattaatatgtctgccgatatccttagtagg

baochun1 tggcggatgtcggaaatggaaagtctatgtattaatatgtatgccgatatccttagtagg

**************************************** *******************

baochun2 gccataaagtgaagaggcatttttccatctaacccgttttatgaattattcctaagggtt

baochun1 gccataaagtgaagaggcatttttccatctaacccgttttatgaattattcctaagggtt

************************************************************

baochun2 cacatcaaaatagtgctagttgatgagagttatttcggaaacaaaatacagtaaagtcaa

baochun1 catatcaaaatagtgctagttgatgagagttatttcggaaacaaaatacagtaaagtcaa

**.*********************************************************

baochun2 attcattaggctatgctctcaattccaataaaatgaaatcagatcaagtatgagttggcg

baochun1 attcattgggctatgctctcaattccaataaaatgaaatcagatcaagtatgagttggcg

*******.****************************************************

baochun2 atcagaacatatatggatagaacttataagggggtctcggaaaataagtaatttttcctg

baochun1 atcagaacatatatggatagaacttataagggggtctcgaaaaataagtaatttttcctg

***************************************.********************

baochun2 ggccattatcctttttttaggttcatcaggcttcttattggttggaacttccagttatct

baochun1 ggccattatcctttttttaggttcatcaggcttcttattggttggaacttccagttatct

************************************************************

baochun2 tgggaaaaatttgatatctttatttccgtctcaacaaatcattttttttccacaagggct

baochun1 tggtaaaaatttgatatctttatttccgtctcaacaaatcattttttttccacaagggct

*** ********************************************************

baochun2 cgtaatgtctttttatgggttcgcgggtctttttattagcgcctacttatggtgtacaat

baochun1 cgtaatgtctttttatgggttcgcgggtctttttattagcgcctacttatggtgtacaat

************************************************************

baochun2 ttcgtggaatgtaggtagtggttatgatcgattcgatagaaaagaaggaatagtgtgtat

baochun1 ttcgtggaatgtaggtagtggttatgatcgattcgatagaaaagaaggaatagtgtgtat

************************************************************

baochun2 ttttcgttggggatttcccggaaaaaatcgtcgtgtcttcctacgatttcttataaaaga

baochun1 ttttcgttggggatttcccggaaaaaatcgtcgtgtcttcctacgatttcttataaaaga

************************************************************

baochun2 tattcagtctgtgcgaatagaagttaaagagggtatttatgctcgccgtgttctttatat

baochun1 tattcagtctgtgcgaatagaagttaaagagggtatttatgctcgccgtgttctttatat

************************************************************

baochun2 ggaaatccgaggccagggggccattcccttgacgcgcactgatgagaatttgacgccacg

baochun1 ggaaatccgaggccagggggccattcccttgacgcgcactgatgagaatttgacgccacg

************************************************************

baochun2 agaaattgaacaaaaagctgccgaattggcctattttttgcgtgtcccaattgaagtttt

baochun1 agaaattgaacaaaaagctgccgaattggcctattttttgcgtgtcccaattgaagtttt

************************************************************

baochun2 ttgagaaataaagaatgaacgccttatcagcagttaaagaaaaagtaaagaatcctcttt

baochun1 ttgagaaataaagaatgaacgccttatcagcagttaaagaaaaagtaaagaatcctcttt

************************************************************

baochun2 tttctctaatataacttcactgaagtttcttcagaacgcagatttgagccaaagcaagac

baochun1 tttctctaatataacttcactgaagtttcttcagaacgcagatttgagccaaagcaagac

************************************************************

baochun2 gtaacagccctaaaacgaaactctttttggggtcttttatgcatatggaaatccattcaa

baochun1 gtaacagccctaaaacgaaactctttttggggtcttttatgcatatggaaatccattcaa

************************************************************

baochun2 ttcagcaacaaaacaagtaacgaatcgaaatattggaattgatactttagatccagatgg

baochun1 ttcagcaacaaaacaagtaacgaatcgaaatattggaattgatactttagatccagatgg

************************************************************

baochun2 atcatatcttgttaattttttattttattacttcattcgaattcgaagtggatttttatt

baochun1 atcatatcttgttaattttttattttattacttcattcgaattcgaagtggatttttatt

************************************************************

baochun2 cgatatttttattttttatagattcaaggactaatcccggaatcccccaaaaagagtgaa

baochun1 cgatatttttattttttatagattcaaggactaatcccggaatcccccaaaaagagtgaa

************************************************************

baochun2 tagactccttcgacaccttgcaatagaacttatgcttcatagaaatattagattgcggag

baochun1 tagactccttcgacaccttgcaatagaacttatgcttcatagaaatattagattgcggag

************************************************************

baochun2 agtcagcgaatgaggtaagttcattcacaattcacaaattcaaatggcaaaaaagaacgc

baochun1 agtcagcgaatgaggtaagttcattcacaattcacaaattcaaatggcaaaaaagaaagc

********************************************************* **

baochun2 atttactcctcttatatatcttgcatctgtagtagttttgccctggtggatttctaataa

baochun1 atttactcctcttatatatcttgcatctgtagtagttttgccctggtggatttctaataa

************************************************************

baochun2 aagtctggaatcttgggttattaattggtggaatactaaacaatccgaaacttttttgaa

baochun1 aagtctggaatcttgggttattaattggtggaatactaaacaatccgaaacttttttgaa

************************************************************

baochun2 tgatattcaagaaaagaatattctagaaaaattcatagaagtagaagaactggtcctctt

baochun1 tgatattcaagaaaagaatattctagaaaaattcatagaagtagaagaactggtcctctt

************************************************************

baochun2 ggatgaaatgatcaaggaatactcggagacacatctacaaaagcttcgtataggaatgca

baochun1 ggatgaaatgatcaaggaatactcggagacacatctacaaaagcttcgtataggaatgca

************************************************************

baochun2 caaagaaacgatccaattaatcaagatatacaatgaggattatatccatacgattttgca

baochun1 caaagaaacgatccaattaatcaagatatacaatgaggattatatccatacgattttgca

************************************************************

baochun2 tttctcgacaaatataatctctttcattattctaagtagttattctattctgggtaatga

baochun1 tttctcgacaaatataatctctttcattattctaagtggttattctattctgggtaatga

*************************************.**********************

baochun2 agaacttgttattcttaactcttgggcgcaagaattcttatataatttaagcgatacaat

baochun1 agaacttgttattcttaactcttgggcgcaagaattcttatataatttaagcgatacaat

************************************************************

baochun2 aaaagctttttctattcttttattaactgatttatgtatcggattccattctccccatgg

baochun1 aaaagctttttctattcttttattaactgatttatgtatcggattccattctccccatgg

************************************************************

baochun2 gtgggaactaatgattggttctgtttacaaagattttggatttgttcataacgatcagat

baochun1 gtgggaactaatgattggttctgtttacaaagattttggatttgttcataacgatcaaat

*********************************************************.**

baochun2 tatatctggtcttgtttctacctttccagttattctagatacaatttttaaatattggat

baochun1 tatatctggtcttgtttctacctttccagttattctagatacaatttttaaatattggat

************************************************************

baochun2 tttccgttatttaaatcggctatctccgtcacttgtagtgatttatcattcaatgaatga

baochun1 tttccgttatttaaatcggctatctccgtcacttgtagtgatttatcattcaatgaatga

************************************************************

baochun2 ttgacaaataatccactcatattaatccaattcgaatctttgttacttttgagttgtaca

baochun1 ttgacaaataatccactcatattaatccaattcgaatctttgttacttttgagttgtaca

************************************************************

baochun2 gaaaaaagaatttcaaattatatttactctttctatttctacttatctcggggattcatc

baochun1 gaaaaaagaatttcaaattatatttactctttctatttctacttatctcggggattcatc

************************************************************

baochun2 ctatattctatattattccagtaaataccagaatcgcggatagggaactatactagtgac

baochun1 ctatattctatattattccagtaaataccagaatcgcggatagggaactatactagtgac

************************************************************

baochun2 ctatcctaatttattgtagaaattttcgggatcaatgattggaccatgcaaactagaaat

baochun1 ctatcctaatttattgtagaaattttcgggatcaatgattggaccatgcaaactagaaat

************************************************************

baochun2 actttttcttggataaaggaacagattactcgatccatttccgcatcgctcatgatatat

baochun1 actttttcttggataaaggaacagattactcgatccatttccgcatcgctcatgatatat

************************************************************

baochun2 atcataacccgtacatccatttcaggtgcatatcccatttttgcacagaaggcttatgaa

baochun1 atcataacccgtacatccatttcaggtgcatatcccatttttgcacagaaggcttatgaa

************************************************************

baochun2 aatccacgagaagcaactgggcgtattgtatgcgctaattgccatttagctaataagccc

baochun1 aatccacgagaagcaactgggcgtattgtatgcgctaattgccatttagctaataagccc

************************************************************

baochun2 gtggatattgaggttccacaagcggtacttcctgatactgtatttgaagcagttgttcga

baochun1 gtggatattgaggttccacaagcggtacttcctgatactgtatttgaagcagttgttcga

************************************************************

baochun2 attccttatgatatgcaattgaaacaagttcttgctaatggtaaaaagggggctttgaat

baochun1 attccttatgatatgcaattgaaacaagttcttgctaatggtaaaaagggggctttgaat

************************************************************

baochun2 gtcggggctgttcttattttacctgagggttttgaattagcccctgctgatcgtatttct

baochun1 gtcggggctgttcttattttacctgagggttttgaattagcccctgctgatcgtatttct

************************************************************

baochun2 cccgagataaaagaaaagataggcaatttgtcttttcagagctatcgccccaataacaaa

baochun1 cccgagataaaagaaaagataggcaatttgtcttttcagagctatcgccccaataacaaa

************************************************************

baochun2 aatattcttgtgataggccctgttcctggtcagaaatatagtgaaatcatctttcctatt

baochun1 aatattcttgtgataggccctgttcctggtcagaaatatagtgaaatcacctttcctatt

*************************************************.**********

baochun2 ctttcaccggaccctgctactaagaaagatgttcactttttaaaatatcctatatacgta

baochun1 ctttcaccggaccctgctactaagaaagatgttcactttttaaaatatcctatatacgta

************************************************************

baochun2 ggtggaaacaggggaaggggtcagatttatcccgacgggaacaagagtaacaatacagtt

baochun1 ggtggaaacaggggaaggggtcagatttatcccgacgggaacaagagtaacaatacagtt

************************************************************

baochun2 tataatgctacaacaacgggaatagtaagcaaaatcatacgaaaagaaaaagggggatac

baochun1 tataatgctacaacaacgggaatagtaagcaaaatcatacgaaaagaaaaagggggatac

************************************************************

baochun2 gaaataacgatagcagatgcatcggatggacgtcaagtagttgatattatccccccaggc

baochun1 gaaataacgatagcagatgcatcggatggacgtcaagtagttgatattatccccccaggc

************************************************************

baochun2 ccggaacttcttgtttcggagggcgaatctatcaaattggatcaaccattaacgagtaat

baochun1 ccggaacttcttgtttcggagggcgaatctatcaaattggatcaaccattaacgagtaat

************************************************************

baochun2 cctaatgtaggcgggtttggtcaaggagatgcagaaatagtacttcaagatccattacgc

baochun1 cctaatgtaggcgggtttggtcaaggagatgcagaaatagtacttcaagatccattacgc

************************************************************

baochun2 attcaaggccttttggtctttttagcatctgttgttttggcacaaatttttttggttctg

baochun1 attcaaggccttttggtctttttagcatctgttgttttggcacaaatttttttggttctg

************************************************************

baochun2 aaaaagaaacagttcgaaaaggttcaattgtccgaaatgaatttctagacttcgtgaatt

baochun1 aaaaagaaacagttcgaaaaggttcaattgtccgaaatgaatttctagacttcgtgaatt

************************************************************

baochun2 tatcagcatcaagtttataaaaataaccaaaaaattattagggattattaaaaaaaaatg

baochun1 tatcagcatcaagtttataaaaataaccaaaaaattattagggattatt-aaaaaaaatg

************************************************* **********

baochun2 aaattctaaaattttttattctttctttttctatgagatgtcaagaagtccgtgtatcgt

baochun1 aaattctaaaattttttattctttctttttctatgagatgccaagaagtccgtgtatcgt

****************************************.*******************

baochun2 gttccgagtcatagtatgtagaatactttttgcctttaccttttctttttttatccaaat

baochun1 gttccgagtcatagtatgtagaatactttttgcctttcccttttctttttttatccaaat

************************************* **********************

baochun2 caaatttggatggtgtgactatattattcttgccagattaaaatatcataatcaataggt

baochun1 caaatttggatggtgtgactatattattcttgccagattaaaatatcataatcaataggt

************************************************************

baochun2 ttttattctaaatagaaatagaagagaatcaaatgaaactagatacaaataaataggaaa

baochun1 ttttattct------aaatagaagagaatcaaatgaaactagatacaaataaataggaaa

********* *********************************************

baochun2 tagagaagggataaacgcgggggaagaaacaattctaggaaggattatttgtttcttagt

baochun1 tagagaagggataaacgcgggggaagaaacaattctaggaaggattatttgtttcttagt

************************************************************

baochun2 cttcgacacaagaaaaggtattttcaagatccctttcttgtgtcgacatcataatgattt

baochun1 cttcgacacaagaaaaggtattttcaagatccctttcttgtgtcgacatcataatgattt

************************************************************

baochun2 ttcatcccgctcgtcaaagatttctatttttagtttttttatagatgtattaatattaga

baochun1 ttcatcccgctcgtcaaagatttctatttttagtttttttatagatgtattaatattaga

************************************************************

baochun2 atgtatttagaactcttttttctatttattttttttaagctttacgtagaatatcgaatc

baochun1 atgtatttagaactcttttttctattta-tttttttaagctttacgtagaatatcgaatc

**************************** *******************************

baochun2 taacaacaaagtgcacaaatacaaaaagatagggagattttagttattgagtaaatataa

baochun1 taacaacaaagtgcacaaatacaaaaagatagggagattttagttattgagtaaatataa

************************************************************

baochun2 cttcttcaatgaacttataaaaaaatatcaaaatggatgggatactaataaatagagtca

baochun1 cttcttcaatgaacttataaaaaaatatcaaaatggatgggatactaataaatagagtca

************************************************************

baochun2 aattctatatcttagggaaaggatttgcataatatctaatttacagaaatgcatattatt

baochun1 aattctatatcttagggaaaggatttgcataatatctaatttacagaaatgcatattatt

************************************************************

baochun2 ttatccaggaaattgacgaattccttctttacttctaaaaattgtaaaagtatattaatg

baochun1 ttatccaggaaattgacgaattccttctttatttctaaaaattgtaaaagtatattaatg

*******************************.****************************

baochun2 actttctagcaaggaatggtcgttcttaatcaattaatttagttcttttttcaaaaatat

baochun1 actttctagcaaggaatggtcgttcttaatcaattaatttagttcttttttcaaaaatat

************************************************************

baochun2 cattagcattag-aaaaaaaatgttttgtcatttaatttatataaaaaaagtttgattag

baochun1 cattagcattagaaaaaaaaatgttttgtc-----atttatataaaaaaagtttgattag

************ ***************** *************************

baochun2 tattaagaactcaacggggcctttcctttcccctcgaatcatcctaaagaaggaagggaa

baochun1 tattaagaactcaacggggcctttcctttcccctcgaatcatactaaagaaggaagggaa

****************************************** *****************

baochun2 tcccgttgaattcttacgctttcgtgtttacaactcaatttatccaattactacttacta

baochun1 tcccgttgagttcttacgctttcg----tacaactcaatttatccaattactacttacta

*********.************** ********************************

baochun2 cagggatgaacctaatccagaataggaaccataaaagaaaatgcctattaaaccgatcac

baochun1 cagggatgaacctaatccagaataggaaccataaaagaaaatgcctattaaaccgatcac

************************************************************

baochun2 aagaataccagctacagtacctattagccaaagaggaatccttccagtagtatcggccat

baochun1 aagaataccagctacagtacctattagccaaagaggaatccttccagtagtatcggccat

************************************************************

baochun2 ttaacccaattccctccacatttcatcaaatggtcatgctagagacataaacagtcatag

baochun1 ttaacccaattccctccacatttcatcaaatggtcatgctagagacataaacagtcatag

************************************************************

baochun2 ataattatgtgatgagatccttccgaatgggctaagagaattcctagagtttttattcta

baochun1 ataattatgtgatgagatccttccgaatgggctaagagaattcctagagtttttattcta

************************************************************

baochun2 taattctattttg-tttttttaattgaaaaaataattggaaaataaaacagcaaggacaa

baochun1 taattctattttgttttttttaattgaaaaaataattggaaaataaaacagcaaggacaa

************* **********************************************

baochun2 aaatgagtaataacccccagtagagactggtacgattcaattcaacactttgttcgtttg

baochun1 aaatgagtaataacccccagtagagactggtacgattcaattcaacactttgttcgtttg

************************************************************

baochun2 ggtttgattgtgtcatagctctataattcggattaggtttatcgttggatgaactgcatt

baochun1 ggtttgattgtgtcatagctctataattcggattaggtttatcgttggatgaactgcatt

************************************************************

baochun2 gctgagattgaccccaaaaaagaaacggtaggtacagctaatccgtgaatagccaaccat

baochun1 gctgagattgaccccaaaaaagaaacggtaggtacagctaatccgtgaatagccaaccat

************************************************************

baochun2 cgcactgtgaaaattggataagttcgatctatggtcattggggcctcctaaaaggatcta

baochun1 cgcactgtgaaaattggataagttcgatctatggtcattggggcctcctaaaaggatcta

************************************************************

baochun2 ctaaattcatcgagttgttccaaaggatcaaaacggccagttattaatggaattccttgt

baochun1 ctaaattcatcgagttgttccaaaggatcaaaacggccagttattaatggaattccttgt

************************************************************

baochun2 cggctctctgtaaaatattcgtttggccgagggcttccaaacacatcgtaagctaagcct

baochun1 cggctctctgtaaaatattcgtttggacgagggcttccaaacacatcgtaagctaagcct

************************** *********************************

baochun2 gtgctgacgaataaccaacccgcaatgaatagggaaggtatagtaatgctatgaatgacc

baochun1 gtgctgacgaataaccaacccgcaatgaatagggaaggtatagtaatgctatgaatgacc

************************************************************

baochun2 cagtatcgaatactggtaataatatcagcaaaagaacgttctcctgtgcttccagacatg

baochun1 cagtatcgaatactggtaataatatcagcaaaagaacgttctcctgtgcttccagacatg

************************************************************

baochun2 ccgagctccacatattcttgtacagtcaaaaggggatcgattccgtaaaagatgagatca

baochun1 ccgagctccacatattcttgtacagtcaaaaggggatcgattccgtaaaagatgagatca

************************************************************

baochun2 gtaaatttccatttactaaaattgaatctttgtgagatcgtcaatattgtaccgagggcg

baochun1 gtaaatttccatttactaaaattgaatctttgtgagatcgtcaatattgtaccgagggcg

************************************************************

baochun2 tctttagagtctaccgaatcagtatagctatccttcttctgacacagcaatgcaatttca

baochun1 tctttagagtctaccgaatcagtatagctatccttcttctgacacagcaatgcaatttca

************************************************************

baochun2 attaatagtcaaataaaatgctagataatttatttctttcgttgttgcttctccatataa

baochun1 attaatagttaaataaaatgctagataatttatttctttccttgttgcttctccatataa

*********.****************************** *******************

baochun2 aaaaaggtgccactcaatactcaataaaaaattatactatttgtataacatttatccctt

baochun1 aaaagggtgccactcaatactcaat-aaaaattatactatttgtataacatttatccctt

****.******************** **********************************

baochun2 caaagttttgagctataaactgaagattcaataactaataaaaagtgaatcgaacaagac

baochun1 caaagttttgggctataaactgaagattcaataactaataaaaagtgaatcgaacaagac

**********.*************************************************

baochun2 aagttggttttaaataattgatctaagggggttatctattattatgttcctagaatttaa

baochun1 aagttggttttaaataattgatctaagggggttatctattattatgttcctagaatttaa

************************************************************

baochun2 gtagatgcaagatctagtaatcttcgtttcgtagtagaatattttgttcgaattcccttt

baochun1 gtagatgcaagatctagtaatcttcgtttcgtagtagaatattttgttcgaattcccttt

************************************************************

baochun2 tgttattttaaggaattgctttgtcatccagtaacaagtaagatcaataccaagtaataa

baochun1 tgttattttaaggaatttctttgtcatccagtaacaagtaagatcaataccaagtaataa

***************** ******************************************

baochun2 tactaaaataatattagctgaaaaaaatagaactaaagaataaaagaattggaatcaata

baochun1 tactaaaataatattagctgaaaaaaatagaactaaagaataaaagaattggaatcaata

************************************************************

baochun2 tttaatttatgatgtatgcattgtttagtagatcgaggttctttcttaacctaactacaa

baochun1 tttaatttatgatgtatgcattgtttagtagatcgaggttctttcttaacctaactacaa

************************************************************

baochun2 ggatgatcttttagataatataatcattaatcatatggaaaaagaaaatggaatctattg

baochun1 ggatgatcttttagataatataatcattaatcatatggaaaaagaaaatggaatctattg

************************************************************

baochun2 agtgaaaataagcgtttaaagtaaaataaaatgttcatttttaaatagaaaatttgatac

baochun1 agtgaaaataagcgtttaaagtaaaataaaatgttcatttttaaatagaaaatttgatac

************************************************************

baochun2 aattaaaagatacaagaaatgatcaaacaaactagaaatcattttccaattttctagtga

baochun1 aattaaaagatacaagaaatgatcaaacaaactagaaatcattttccaattttctagtga

************************************************************

baochun2 ttgatt-aaaaaaaggagtttctttttttaattaaagttacacaacatagtactttttct

baochun1 ttgattaaaaaaaaggagtttctttttttaattaaagttacacaacatagtac-------

****** **********************************************

baochun2 agttttttttctaatttattcaagaattgctcacgaaatccgttggttggaattacggta

baochun1 -----tttttctaatttattcaagaattgctcacgaaatccgttggttggaattacggta

*******************************************************

baochun2 acggtattaggtagatgtcacaaatgatgaataaatttctttttatacacccgtcacttt

baochun1 acggtattaggtagatgtcacaaatgatgaataaatttctttttatacacccgtcacttt

************************************************************

baochun2 ctctttgtttcgttaatgctatctatagtgattgaggaatcaaaaagtttcaattaaact

baochun1 ctctttgtttcgttaatgctatctatagtgattgaggaatcaaaaagtttcaattaaact

************************************************************

baochun2 tattatttcaaaatagtatttttgcttatcctacgatcttgcaaaaatggaaactcactt

baochun1 tattatttcaaaatagtatttttgcttatcctacgatcttgcaaaaatggaaactcactt

************************************************************

baochun2 aggtaagtgctttagaaacctatgtataaaacaatatatttcattatttatccccttcat

baochun1 aggtaagtgctttagaaacctatgtataaaacaatatatttc---atttatccccttcat

****************************************** ***************

baochun2 gcttacgataactagttatttcggttttttactagcagctttaactataacctcggctct

baochun1 gcttacgataactagttatttcggttttttactagcagctttaactataacctcggctct

************************************************************

baochun2 ttttattggtctgagcaaggtacgacttatttgaaattcttatgtgggattccatgtatt

baochun1 ttttattggtctgagcaaggtacgacttatttgaaattcttatgtgggattccgtgtatt

*****************************************************.******

baochun2 ctatagttacttaccagtccatttgccaattcttgatcattgagattcgtggcaattcgc

baochun1 ctatagttacttaccagtccatttgccaattcttgatcattgagattcgtggcaattcgc

************************************************************

baochun2 attaatatttaggtatagatattaccta-tttttttctttcaaacaaatttaataaatga

baochun1 attaatatttaggtatagatattacctattttttttctttcaaacaaatttaataaatga

**************************** *******************************

baochun2 ttgaagtttttctatttggaattgtgttaggtctcattcctattactttggctggattat

baochun1 ttgaagtttttctatttggaattgtgttaggtctcattcctattactttggctggattat

************************************************************

baochun2 ttgtaactgcatatttacaatacagacgtggtgatcagttagacctttaattaacatcta

baochun1 ttgtaactgcatatttacaatacagacgtggtgatcagttagacctttaattaacatcta

************************************************************

baochun2 tctattttttgattgacctcttcctttcttttatccataggaggtaaaagtcagcttgct

baochun1 tctattttttgattgacctcctcctttcttttatccataggaggtcaaagtcagcttgct

********************.************************ **************

baochun2 gttcaagttaatgaagctttgtcagtctaatggacctatgaaaaatggaatcacgctctg

baochun1 gttcaagttaatgaagctttgtcagtctaatggacctatgaaaaatggaatcacgctctg

************************************************************

baochun2 taggatttgaacctacgacatcgggttttggagacccgcgttctaccgaactgaactaag

baochun1 taggatttgaacctacgacatcgggttttggagacccgcgttctaccgaactgaactaag

************************************************************

baochun2 agcgctttcttatcagaatagataagataagactttaatcaaaattaaagaaaaataata

baochun1 agcgctttcttatcagaatagataagataagactttaatcaaaa-taaagaaaaataata

******************************************** ***************

baochun2 tttttgtaactctaatatagcgtggatgcatttactataaaaaatattctagatataata

baochun1 tttttgtaactctaatatagcgtggatgcatttactataaaaaatattatagatataata

************************************************ ***********

baochun2 tattgccaaactgaaaatttattcatttcaattgatccctcgttactgctcaaaggagaa

baochun1 tattgccaaactgaaaatttattcatttcaattgatccctcgttactgctcaaaggagaa

************************************************************

baochun2 gtaataggtagggatgacaggatttgaacccgtgacattttgtacccaaaacaaacgcgc

baochun1 gtaataggtagggatgacaggatttgaacccgtgacattttgtacccaaaacaaacgcgc

************************************************************

baochun2 taccaagctgcgctacatcccttctaatttgtctacagtgtcattgtatagaattcttat

baochun1 taccaagctgcgctacatcccttctaatttgtctacagtgtcattgtatagaattcctat

********************************************************.***

baochun2 cttgttttccacatacttatttcttacattgaaataaactttccatttattctttttggt

baochun1 cttgttttccacatacttatttcttacattgaaataaactttccatttattctttttggt

************************************************************

baochun2 ctcatataacatataataatagtaaaaaaccttactatataggaaatacaaaatctaccg

baochun1 ctcatataacatataataatagtaaaaaaccttactatataggaaatacaaaatctaccg

************************************************************

baochun2 gaagtaaaaaaaaaaattggaatacttagtaagaaagaaatgcattttttcttttatttt

baochun1 gaagtaaaaaaaaaaattggaatacttagtaagaaagaaatgcattttttcttttatttt

************************************************************

baochun2 aagcaaaagaaaatcctctcgactggatcattgtacatttcaattagaattagagattcc

baochun1 aagcaaaagaaaatcctctcgactggatcattgtacatttcaattagaattagagattcc

************************************************************

baochun2 gcgcacaactacaagtgtaccttaactacatatgcatttgatcatatatggattaccatt

baochun1 gcgcacaactacaagtgtaccttaactacatatgcatttgatcatatatggattaccatt

************************************************************

baochun2 atttattaataataaaataacgaaggaggtttttgaatgcgagatttcaaaacatatctc

baochun1 atttattaataataaaataacgaaggaggtttttgaatgcgagatttcaaaacatatctc

************************************************************

baochun2 tctgtggcaccggtactaagtactctatggttcgggtctttagcaggtctattgatcgag

baochun1 tctgtggcaccggtactaagtactctatggttcgggtctttagcaggtctattgatcgag

************************************************************

baochun2 attaatcgtttttttccggatgcgttgacattcccgtttttttcattctagttatttgca

baochun1 attaatcgtttttttccggatgcgttgacattcccgtttttttcattctagttatttgca

************************************************************

baochun2 cgggaaggaatgaagaagattctagatagaataaaatatctatgtgactaatccctttcc

baochun1 cgggaaggaatgaagaagattatagatagaataaaatatctatgtgactaatccctttcc

********************* **************************************

baochun2 ccctcttttctcttttttcccttttttataaaaaaaacaaaataagggacgaaagagaaa

baochun1 ccctcttttctcttttttcccttttttataaaaaaaacaaaataagggacgaaagagaaa

************************************************************

baochun2 aaataaagtgaattcaacattttattattttatttgagaaattcgagtttgaatgacatt

baochun1 aaataaagtgaattcaacattttattattttatttgagaaattcgagtttgaatgacatt

************************************************************

baochun2 catatataattatatatttatagaagaattggggggagagcataaaagtttggtttagtc

baochun1 catatataattatata-ttatagaagaattggggggagagcataaaagtttggtttagtc

**************** *******************************************

baochun2 taggccaaaagtattatacaaaatactgtactacgattcgaaatatgttttgaaatcatt

baochun1 taggacaaaagtattatacaaaatactgtactacgattcgaaatatgttttgaaatcatt

**** *******************************************************

baochun2 gtattacttattatttactattacttgcttgattccaagcaaatttttcctaattggatt

baochun1 gtattacttattatttactattacttgcttgattccaagcaaatttttcctaattggatt

************************************************************

baochun2 tcaaataagtaacttatattgtttttccttcctttcttcttcgtttaggatcaaaaatag

baochun1 tcaaataagtaacttatattgtttttccttcctttcttcttcgtttaggatcaaaaatag

************************************************************

baochun2 acgagtgtagcaaataaaaaaatccaaaggaggttcatggccaaggggaaagatgtccga

baochun1 acgagtgtagcaaataaaaaaatccaaaggaggttcatggccaaggggaaagatgtccga

************************************************************

baochun2 gtaactgttattttggaatgtaccagttgtgtccgaaacggtgttaataagtcagcaacg

baochun1 gtaactgttattttggaatgtaccagttgtgtccgaaacggtgttaataagtcagcaacg

************************************************************

baochun2 gggatttccagatatattactcaaaagaatcgacacaatacgtccaatcgattggaattg

baochun1 gggatttccagatatattactcaaaagaatcgacacaatacgcccaatcgattggaattg

******************************************.*****************

baochun2 agaaaattctgtccctattgttacaaacatacaattcatggggagataaaaaaatagatc

baochun1 agaaaattctgtccctattgttacaaacatacaattcatggggagataaaaaaatagatc

************************************************************

baochun2 gaatcaagtacttgcgtgtcacctttccaagatacaaggtaggggaaaaatgacattata

baochun1 gaatcgagtacttgcgtgtcacctttccaagatacaaggtaggggaaaaatgacattata

*****.******************************************************

baochun2 tataataacatatttaataaacaaagcaaatcctatttgaattcatttttgatgtgacca

baochun1 tataataacatatttaataaacaaagcaaatcctatttgaattcatttttgatgcgacca

******************************************************.*****

baochun2 taatagggttttcacgataagaactaaactaaacaaaccatggataaatccaagcgaccc

baochun1 taatagggttttcacgataagaactaaactaaacaaaccatggataaatccaagcgaccc

************************************************************

baochun2 tttcttaaatccaagcgatcttttcgtaggcgtttacccccgatccaatcgggggatcaa

baochun1 tttcttaaatccaagcgatcttttcgtaggcgtttacccccgatccaatcgggggatcaa

************************************************************

baochun2 attgattatagaaacatgagtttaattagtcgatttattagtgaacaaggaaaaatacta

baochun1 attgattatagaaacatgagtttaattagtcgatttattagtgaacaaggaaaaatacta

************************************************************

baochun2 tctagacgagtgaatagattgaccttgaaacaacaacgattaattactattgctataaaa

baochun1 tctagacgagtgaatagattgaccttgaaacaacaacgattaattactattgctataaaa

************************************************************

baochun2 caagctcgtattttatctttgttaccttttcttaataatgagaaacaatttgaaaaaagc

baochun1 caagctcgtattttatctttgttaccttttcttaataatgagaaacaatttgaaaaaagc

************************************************************

baochun2 gagtcgaccgctagaactgccggtcttagaactagaattaaataagcttattatttcttc

baochun1 gagtcgaccgctagaactgccggtcttagaactagaattaaataagcttattatttcttc

************************************************************

baochun2 aattgaattaaaattccaatcgatactcaaacgcattttgttcaaaaacccgaaaattca

baochun1 aattgaattaaaattccaatcgacactcaaacgcattttgttcaaaaacccgaaaatcca

***********************.*********************************.**

baochun2 gattttggtatcgtgtcataagaataagaaaaaactcgggtaaagcgaagaaatcttttt

baochun1 gattttggtatcgtgtcataagaataagaaaaaactcgggtaaagcgaagaaatcttttt

************************************************************

baochun2 ttattgaacgcggttatttattatttatgttgactactttctcatatcataataattttt

baochun1 ttattgaacgcggttatttattatttatgttgactactttctcatatcataataattttt

************************************************************

baochun2 tctcatagtcatttttactctatcctcccggagtttattctccggggaattcgattctag

baochun1 tctcatagtcatttttactctatcctcccggagtttattctccggggaattcgattctag

************************************************************

baochun2 tggatttcttagaatcgacttattttatgatctcgttggaaatcatataaagactttttt

baochun1 tggatttcttagaatcgacttattttatgatctcgttggaaatcatataaagactttttt

************************************************************

baochun2 tatttaatatagctatttgcgcaagtatttttcgattaagaagcactcggctcttgtaca

baochun1 tatttaatatagctatttgcgcaagtatttttcgattaagaagcactcggctcttgtaca

************************************************************

baochun2 aatcatgtattaatttactataactataggatacccctttttcgcgaattactgcgttta

baochun1 aatcatgtattaatttactataactataggatacccctttttcgcgaattactgcgttta

************************************************************

baochun2 tacgagtgatccacaaacgacgaaaggttatcttttgtttatctctatcccgatgagccg

baochun1 tacgagtgatccacaaacgacgaaaggttatcttttgtttatctctatcccgatgagccg

************************************************************

baochun2 aaaccaaagatcttattttctgttgagtaatagttcgagtaagtcttgaatgagcccctc

baochun1 aaaccaaagatcttattttctgttgagtaatagttcgagtaagtcttgaatgagcccctc

************************************************************

baochun2 gaaagcttgaggcaaataaacgaatttttgttcgacgtctccgagctatatatccccgtc

baochun1 gaaagcttgaggcaaataaacgaatttttgttcgacgtctccgagctatatatccccgtc

************************************************************

baochun2 taattctggtcattgaataaatgaaactttgataaatagaataactaatggatttacttt

baochun1 taattctggtcattgaataaatgaaactttgatgaatagaataactaatggatttacttt

*********************************.**************************

baochun2 ctttaagtttttctattcccctttctcagtctattaataacaaaacggatttttccaatg

baochun1 ctttaagtttttctattcccctttctcagtctattaataacaaaacggatttttccaatg

************************************************************

baochun2 tataaaataaaaattccaacggctttagctactataaccttcccgaccacaattttgtat

baochun1 tataaaataaaaattccaacggctttagctactataaccttcccgaccacaattttttat

******************************************************** ***

baochun2 ctcgaaaaataaattgtattcgactaggtatcaaaaacataataaataaaaaactagtaa

baochun1 ctcgaaaaataaattgtattcgactaggtatcaaaaacatagtaaataaaaaactagtaa

*****************************************.******************

baochun2 atggataaataaatagtgggtttcatcgtttctatggttaattcgtaaacggtgaggtct

baochun1 atggataaataaatagtgggtttcatcgtttctatggttaattcgtaaacggtgaggtct

************************************************************

baochun2 tctctatacaccggagcctatctaattta----------gtaaccttatgagtaacttgt

baochun1 tctctatacaccggagcctatctaatttatttaattaacgtaaccttatgagtaacttgt

***************************** *********************

baochun2 acagttcatattctttggctcgacccataaattagtcagtaatcggtctttcacaagtag

baochun1 acagttcatattctttggctcgacccataaattagccagtaatcggtctttcacaagtag

***********************************.************************

baochun2 atccacctatacagtaacggtatttaattatgaaagttagctgggtagctgaccctctta

baochun1 atccacctatacagtaacggtatttaattatgaaagttagctgggtagctgaccctctta

************************************************************

baochun2 gtccgttcttgcaagaatagaagtctaatctttcttttttaaaataagattgtccccgct

baochun1 gtccgttcttgcaagaatagaagtctaatctttcttttttaaaataagattgtccccgct

************************************************************

baochun2 taatggataaccatttaataccaatgggg---catttttttttcatcttaaattaaattg

baochun1 taatggataaccatttaataccaatgggggcatttttttttttcatcttaaattaaattg

***************************** . **************************

baochun2 aggtaattggatttacaccaatggaaaccataaatttcatacacaatagaaggatagatt

baochun1 aggtaattggatttacaccaatggaaaccataaatttcatacacaatagaaggatagatt

************************************************************

baochun2 ttattatttttcgaaagtgaatggaatagagttcttccattttatcctattaatcggtac

baochun1 ttattatttttcgaaagtgaatggaatagagttcttccattttatcctattaatcggtac

************************************************************

baochun2 tgatcattgatactggaaatttgttttcttgtgctccagcctatgatctgaacgagtcgc

baochun1 tgatcattgatactggaaatttgttttcttgtgctccagcctatgatctgaacgagtcgc

************************************************************

baochun2 acatacacccgagtacatgttcctcgacgctgagggcatccccgaagagcgggggatttc

baochun1 acatacacccgagtacatgttcctcgacgctgagggcatccccgaagagcgggggatttc

************************************************************

baochun2 gtaacatttctgattggctgtcttgtatttctaataagttgtttaatcgttggcatgttg

baochun1 gtaacatttctgattggctgtcttgtatttctaataagttgtttaatcgttggcatgttg

************************************************************

baochun2 aatgatatacataatgagctggtttagatcgatcctaaccggatgattataaattattaa

baochun1 aatgatatacataatgagctggtttagatcgatcctaaccggatgattataaattattaa

************************************************************

baochun2 tataataaaatatttaactcggtaagaagataaaataataaatcaccaatttgcgctaaa

baochun1 tataataaaatatttaactcggtaagaagataaaataataaatcaccaatttgcgctaaa

************************************************************

baochun2 tccatcctattttctattttcattcaactgctacaagatcaacaattccgtaagcttgtg

baochun1 tccatcctattttctattttcattcaactgctacaagatcaacaattccataagcttgtg

*************************************************.**********

baochun2 cttctgttgctgacataaaaacatctctttccatgtcttcggatacaacccataggggtt

baochun1 cttctgttgctgacataaaaacatctctttccatgtcttcggatacaacccataggggtt

************************************************************

baochun2 ttcccgttctttgtacataaacccttgtgatggtttcacgcagttttagcagctcctccg

baochun1 ttcccgttctttgtacataaacccttgtgatggtttcacgcagttttagcagctcctccg

************************************************************

baochun2 cttccaagatagcttctcccgtttgtgcttcataaaaagcactagcgggttgatgaatca

baochun1 cttccaagatagcttctcccgtttgtgcttcataaaaagcactagcgggttgatgaatca

************************************************************

baochun2 ttaccctgatgatataacataataaagtttcttctatctagatgatggagtgaagagaaa

baochun1 ttaccctgatgatataacataataaagtttcttctatctagatgatggagtgaagagaaa

************************************************************

baochun2 ataaataaagataaaaaaaatagaataaccgtacgggcattttttatgtattgcatacgg

baochun1 ataaataaagataaaaaaaatagaataaccgtacgggcattttttatgtattgcatacgg

************************************************************

baochun2 ctgtacaataaaattgatctttaccttccatcacagaaagaaacaaataggatctataag

baochun1 ctgtacaataaaattgatctttaccttccatcacagaaagaaacaaataggatctataag

************************************************************

baochun2 actcatattggtaaatgatcaaattaccacccttctttttggaggagttaaaaaatacta

baochun1 actcatattggtaaatgatcaaattaccacccttctttttggaggagttaaaaaatacta

************************************************************

baochun2 tgatggttccgttgctttatatatttcttattgattttttggtatttatttgtctgttat

baochun1 tgatggttccgttgctttatatatttcttattgattttttggtatttatttgtctgttat

************************************************************

baochun2 tcagcaatcccaaagtttctttttgatccgatcaaataaaaaaaatattttttatttata

baochun1 tcagcaatcccaaagtttctttttgatccgatcaaat-aaaaaaatattttttatttata

************************************* **********************

baochun2 caataaatattattaagagatctagggtatgaaaaagtttgtgacgctgaacagtattcc

baochun1 caataaatattattaagagatctagggtatgaaaaagtttgtgacgctgaacagtattcc

************************************************************

baochun2 cgatggataagataaaatcagaaataccctttatttcatactactctctcgatatataat

baochun1 cgatggataagataaaatcagaaataccctttatttcatactactctctcgatatataat

************************************************************

baochun2 ataattttttgttttgaaaaataatcaaaattttctcatatcgaattctaaatgccatgc

baochun1 ataattttttgttttgaaaaataatcaaaattttctcatatcgaattctaaatgccatgc

************************************************************

baochun2 tattttgacttaaatattcctatttcatatggcgaaggcatagtcttttgtttctctcaa

baochun1 tatttttacttaaatattcctatttcatatggcgaaggcatagtcttttgtttctctcaa

****** *****************************************************

baochun2 aaaactgcattggcgccaagcgtgagggaatgctagacgtttggtaatttcccctccaac

baochun1 aaaactgcattggcgccaagcgtgagggaatgctagacgtttggtaatttcccctccaac

************************************************************

baochun2 caggataaaagatcccattgaagcagctaatcccatgcatattgtatgtacatttggtcg

baochun1 caggataaaagatcccattgaagcagctaatcccatgcatattgtatgtacatttggtcg

************************************************************

baochun2 cacaaattgcatagtatcaaaaatagctactccaggtattacccatccaccaggagagtt

baochun1 cacaaattgcatagtatcaaaaatagctactccaggtattacccatccaccaggagagtt

************************************************************

baochun2 tataaacaaataaagatctttggtatcatcctcaatactgagatataccataagaccaat

baochun1 tataaacaaataaagatctttggtatcatcctcaatactgagatataccataagaccaat

************************************************************

baochun2 aagctgatttgagatctcgctatcaactgcttggcctaaaaaaagtaatctctctcgata

baochun1 aagctgatttgagatctcgctatcaactgcttggcctaaaaaaagtaatctctctcgata

************************************************************

baochun2 aagtcggttgattagggtaaagttgtatcccttagaaaccgtacatgcacttttttctgc

baochun1 aagtcggttgattagggtaaagttgtatcccttagaaaccgtacatgcacttttttctgc

************************************************************

baochun2 atacggttcaaaaaatttttcgaaaaaatcaatgtgtagattccagccctctttcgtttt

baochun1 atacggttcaaaaaatttttcgaaaaaatcaatgtgtagattccagccctctttcgtttt

************************************************************

baochun2 gtcatatcatacatagcatagtcatagtaagctctttctaacttttaacgaaagggcttt

baochun1 gtcatatcatacatagcatagtcatagtaggctctttttaacttttaacgaaagggcttt

*****************************.*******.**********************

baochun2 ttcttcgatttttcaataaagattttttttaactccttttcttatgttagaatataaaaa

baochun1 ttcttcgatttttcaataaagattttttttgactccttttcttatgttagaatataaaaa

******************************.*****************************

baochun2 aagaatttaaaacttatcgaattaacttatcattgatgtatttttttcattgagatccaa

baochun1 aagaatttaaaacttatcgaattaacttatcattgatgta-ttttttcattgagatccaa

**************************************** *******************

baochun2 atcgcgatgtcattttcttgttcctgaatgggtttcttaaatctttttaggtttatgctc

baochun1 atcgcgatgtcattttcttgttcctgaatgggtttcttaaatctttttaggtttatgctc

************************************************************

baochun2 tactccgggtaaagatctgccctattttgatttgcacatataggacaaatgcttccctta

baochun1 tactccgggtaaagatctgccctattttgatttgcacatataggacaaatgcttccctta

************************************************************

baochun2 ccacttctttattgctatgatttcttaatta------attattaatttcatgccttctac

baochun1 ccacttctttattgctatgatttcttaattaattattattattaatttcatgccttctac

******************************* ***********************

baochun2 caaaccaaatatttgatgggattcatccatattacttgattgattaacgatttgagataa

baochun1 caaaccaaatatttgatgggattcatccatattactcgattgattaacgatttaagataa

************************************.****************.******

baochun2 gttttctttataaataggaatcgaatcatttatgatacaagccgtaatcatatataatta

baochun1 gttttctttataaataggaatcgaatcatttataatacaagccgtaatcatatataatta

*********************************.**************************

baochun2 ctaattggattgttttttaaacggagcctgaaatacttaattttattagtccaacgaaga

baochun1 ctaattggattgttttttaaacggagcctgaaatacttaattttattagtccaacgaaga

************************************************************

baochun2 taaccataaattattcaaattgataataataataatatgaattccctccaaaaaaaaggg

baochun1 taaccataaattattcaaattgataataataataatatgaattccctccaaaaaaaaggg

************************************************************

baochun2 ggggatcgagttgcattgcacttcactctcctaatttttacttcacgctcccaatttttt

baochun1 ggggatcgagttgcattgcacttcactctcctaatttttacttcacgctcccaatttttt

************************************************************

baochun2 atgattcaattaatatttcttttgggcaaaatggaggatatctcgatcgggggagaaaac

baochun1 atgattcaattaatatttcttttgggcaaaatggaggatatctcgatcgggggagaaaac

************************************************************

baochun2 gggtaaatcccatatgacccaatatatctgacaagtcgcactagacgtcaatccaagatg

baochun1 gggtaaatcccatatgacccaatatatctgacaagtcgcactatacgtcaatccaagatg

******************************************* ****************

baochun2 catcttcctctccaggactccgaaaaggtacttttggaacaccaataggcattaaataaa

baochun1 catcttcctctccaggacttcgaaaaggtacttttggaacaccaataggcattaaataaa

*******************.****************************************

baochun2 ataaattaaagaactaaatactatatttcactttgatgtggaaacgtaagaatggattta

baochun1 ataaa-taaagaactaaatactatatttcactttgatgtggaaacgtaagaatggattta

***** ******************************************************

baochun2 ttgtctttctactaatattagccctt-gtattagtttatccatagattagaaaacttcat

baochun1 ttgtctttctactaatattagcccttagtattagtttatccatagattagaaaacttcat

************************** *********************************

baochun2 acataatataataaaacgaagacagaatgaataaagaaaaattattaaaaataggtcttt

baochun1 acataatataataaaacgaagacagaatgaataaagaaaaattattaaaaataggtcttt

************************************************************

baochun2 ctaacgatgaagtagtatgcgcatattcccccataaggaggagtttcaacccccattgcg

baochun1 ctaacgatgaagtagtatgcgcatattccctcataaggaggagtttcaacccccattgcg

******************************.*****************************

baochun2 tattggtacttattgagtatagaataaatttgcttcgccttgttcctacgaacataattg

baochun1 tattggtacttattgagtatagaataaatttgcttcgccttgttcctacgaacataattg

************************************************************

baochun2 ttctatatattatcaacaaaatagaacaaccccccttattctaagagaatccactgaaca

baochun1 ttctatatattatcaacaaaatagaacaaccccccttattctaagagaatccactgaaca

************************************************************

baochun2 aaggggatctatagcatagtctatagtagagtctttttcaatgcaataaagttacgtagt

baochun1 aaggggatctatagcatagtctatagtagagtctttttcaatgcaataaagttacgtagt

************************************************************

baochun2 gtctacttttgtttgataaaggggtatttccatgggtttaccttggtatcgtgttcatac

baochun1 gtctacttttgtttgataaaggggtatttccatgggtttaccttggtatcgtgttcatac

************************************************************

baochun2 tgttgtgttgaatgatcccggtcggttacttgctgtccatataatgcatacagctttggt

baochun1 tgttgtgttgaatgatcccggtcggttacttgctgtccatataatgcatacagctttggt

************************************************************

baochun2 ttctggttgggccggttcaatggctctgtatgaattagcagtttttgatccctctgaccc

baochun1 ttctggttgggctggttcaatggctctgtatgaattagcagtttttgatccctctgaccc

************.***********************************************

baochun2 tgttcttgatccaatgtggagacagggtatgtttgttattcctttcatgactcgtttagg

baochun1 tgttcttgatccaatgtggagacagggtatgtttgttattcctttcatgactcgtttagg

************************************************************

baochun2 aataaccaattcatggggcggttggagtatcacaggagggactggcacgaatccgggtat

baochun1 aataaccaattcatggggcggttggagtatcacaggagggactggcacgaatccgggtat

************************************************************

baochun2 ttggagttacgaaggtgtagccggggcacatattgtattttctggcttgtgcttcttggc

baochun1 ttggagttacgaaggtgtagccggggcacatattgtattttctggcttgtgcttcttggc

************************************************************

baochun2 agctatctggcattgggtgtattgggatcttgaaatttttcgtgatgaacgtacaggaaa

baochun1 agctatctggcattgggtgtattgggatcttgaaatttttcgtgatgaacgtacaggaaa

************************************************************

baochun2 acccgttttggatttgcctaaaatctttggaattcatttatttctcgcagggttagcttg

baochun1 acccgttttggatttgcctaaaatctttggaattcatttatttctcgcagggttagcttg

************************************************************

baochun2 ttttggttttggtgtatttcatgtaacaggcttatatggtcctggaatatgggtgtccga

baochun1 ttttggttttggtgtatttcatgtaacaggcttatatggtcctggaatatgggtgtccga

************************************************************

baochun2 tccttatggactaacaggaaaagtacaacctgtaaatccggcgtggggggtggaaggttt

baochun1 tccttatggactaacaggaaaagtacaacctgtaaatccggcgtggggggtggaaggttt

************************************************************

baochun2 tgatccgtttgttccgggaggaatagcctctcatcatattgcagcaggaacattgggcat

baochun1 tgatccgtttgttccgggaggaatagcctctcatcatattgcagcaggaacattgggcat

************************************************************

baochun2 attagcgggcctatttcatcttagcgtccgcccgccccaacgtctatacaaaggcttacg

baochun1 attagcgggcctatttcatcttagcgtccgcccgccccaacgtctatacaaaggcttacg

************************************************************

baochun2 tatgggcaatattgaaaccgtcctttctagcagtatcgccgctgtattttttgcagcttt

baochun1 tatgggcaatattgaaaccgtcctttctagcagtatcgccgctgtattttttgcagcttt

************************************************************

baochun2 tgttgttgccggaactatgtggtatggttcggcaactacccccatcgaattatttgggcc

baochun1 tgttgttgccggaactatgtggtatggttcggcaactacccccatcgaattatttgggcc

************************************************************

baochun2 cactcgttatcaatgggatcaggggtacttccagcaagaaatatatcgaagagttggcgc

baochun1 cactcgttatcaatgggatcaggggtacttccagcaagaaatatatcgaagagttggcgc

************************************************************

baochun2 tggactggccgaaaatcaaagtttatcagaagcttggtctaaaattcctgaaaaattagc

baochun1 tggactggccgaaaatcaaagtttatcagaagcttggtctaaaattcctgaaaaattagc

************************************************************

baochun2 tttttatgattacataggtaataatccagcaaaaggcggattattcagggcgggttcaat

baochun1 tttttatgattacataggtaataatccagcaaaaggcggattattcagggcgggttcaat

************************************************************

baochun2 ggataacggggatggaatagcggttggatggttaggacatcctatctttagagataaaga

baochun1 ggataacggggacggaatagcggttggatggttaggacatcctatctttagagataaaga

************.***********************************************

baochun2 agggcgtgagctttttgtacgccgtatgcctaccttttttgaaacatttccggttgtttt

baochun1 agggcgtgagctttttgtacgccgtatgcctaccttttttgaaacatttccggttgtttt

************************************************************

baochun2 ggtagacggtgatggaattgttagagctgacgttccttttagaagagcagaatcgaagta

baochun1 ggtagacggtgatggaattgttagagctgacgttccttttagaagagcagaatcgaagta

************************************************************

baochun2 tagtgtcgaacaagtaggtgtaactgttgagttctacggcggggaacttaatggagtcag

baochun1 tagtgtcgaacaagtaggtgtaactgttgagttctacggcggggaacttaatggagtcag

************************************************************

baochun2 ttataatgaccccgctactgtaaaaaaatatgctagacgtgctcaattaggtgaaatttt

baochun1 ttataatgaccccgctactgtaaaaaaatatgctagacgtgctcaattaggtgaaatttt

************************************************************

baochun2 tgaattagatcgtgctactttgaaatccgatggtgtttttcgtagtagtccaaggggttg

baochun1 tgaattagatcgtgctactttgaaatccgacggtgtttttcgtagtagtccaaggggttg

******************************.*****************************

baochun2 gtttacttttgggcatgtttcatttgctttgctcttcttcttcggtcacatttggcatgg

baochun1 gtttacttttgggcatgtttcatttgctttgctcttcttcttcggtcacatttggcatgg

************************************************************

baochun2 tgctagaactttgttccgagatgtttttgccggtattgacccagatttggacgctcaagt

baochun1 tgctagaactttgttccgagatgtttttgccggtattgacccagatttggacgctcaagt

************************************************************

baochun2 ggaatttggggcattccaaaaacttggcgatccaactacaagaagacagggagtttgata

baochun1 ggaatttggggcattccaaaaacttggagatccaactacaagaagacagggagtttgata

*************************** ********************************

baochun2 caacattacttcggtaccgttcacctttattttctttttatgatttgacatagaatacca

baochun1 caacattacttcggtaccgttcacctttattttctttttatgatttgacatagaatacca

************************************************************

baochun2 gagaaatcttaattacaatcattacttttttattcttattttttttgtttatacgtgcga

baochun1 gagaaatcttaattacaatcattacttttttattcttatttttgttgtttatacgtgaga

******************************************* ************* **

baochun2 tgagtctaaataaaataaaaaagaaataggtatggaagctataattgtaaaccacgattg

baochun1 tgagtctaaataaaataaaaaagaaataggtatggaagctataattgtaaaccacgattg

************************************************************

baochun2 aatctatggaagctttggtttatacatttctcttagtctcgactctagggataatttttt

baochun1 aatctatggaagctttggtttatacatttctcttagtctcgactctagggataatttttt

************************************************************

baochun2 tcgctatcttttttcgagaaccgcctaaagttccaactaaaaagagtaaataatttttca

baochun1 tcgctatcttttttcgagaaccgcctaaagttccaactaaaaagagtaaataatttttca

************************************************************

baochun2 ttatatcaattgaagtaaagatcctcccgatat-----tgggaggatctttacttcaatt

baochun1 ttatatcaattgaagtaaagatcctcccaatattgggatgggaggatctttacttcaatt

****************************.**** **********************

baochun2 agtctccatgttcctcgaatggatctcgtagttgttgagaaggttgcccaaaagcggtat

baochun1 agtctccatgttcctcgaatggatctcgtagttgttgagaaggttgcccaaaagcggtat

************************************************************

baochun2 ataaggcatacccagtaaaacttacaagtaaaccagatataaagatggcgactagggttg

baochun1 ataaggcatacccagtaaaacttacaagtaaaccagatataaagatggtgactagggttg

************************************************.***********

baochun2 ctgtttccattattatataattttaagatcaaaatggatctatgataagatcgtttattt

baochun1 ctgtttccattattatataattttaagatcaaaatggatctatgataagatcgtttattt

************************************************************

baochun2 acaacggaatagtatacaaagtcaacagatctcaatgaatataataggatttatggctac

baochun1 acaacggaatagtatacaaagtcaacagatctcaatgaatataataggatttatggctac

************************************************************

baochun2 aaaaactgttgagaccgtttctagacccggtcttggtccaagaccaaaacgaactaatct

baochun1 aaaaactgttgagaccgtttctagacccggtcttggtccaagaccaaaacgaactaatct

************************************************************

baochun2 agggggtttattgaaaccattgaattcggaatatggtaaagtagctcctggatggggaac

baochun1 agggggtttattgaaaccattgaattcggaatatggtaaagtagctcctggatggggaac

************************************************************

baochun2 aactccttttatgggtgttgcaatggctttatttgcaatatttctatctattattttgga

baochun1 aactccttttatgggtgttgtaatggctttatttgcaatatttctatctattattttgga

********************.***************************************

baochun2 gatttataattcttctgttttattggatggaatttcaatgaattagatatattaagagct

baochun1 gatttataattcttctgttttattggatggaatttcaatgaattagatatattaagaact

*********************************************************.**

baochun2 gggcagtcctatctttcgataaaatttcaataaaaaaaatcctttagagctcggatttct

baochun1 gggcagtcctatctttcgataaaatttcaataaaaaaaatcctttagagctcggatttct

************************************************************

baochun2 atttctagcccatctttggtggcagtttgaccgcgaaatttctttgtttctgtatttccg

baochun1 atttctagcccatctttggtggcagtttgaccgcgaaatttctttgtttctgtatttccg

************************************************************

baochun2 gaatatgagtgtgtgacttgttataattgatcctattgatagtacagaaaatggatctgt

baochun1 gaatatgagtgtgtgacttgttataattgatcctattgatagtacagaaaatggatctgt

************************************************************

baochun2 catcttaatagagatggttttacttcgtcggatatttattctagtatctggaacacggaa

baochun1 catcttaatagagatggttttacttcgtcggatatttattctagtatctggaacacggaa

************************************************************

baochun2 taggtgaaatcggttaagaaatttttgaactatgatttatacttaatattcagaccctgc

baochun1 taggtgaaatcggttaagaaatttttgaactatgatttatacttaatattcagaccctgc

************************************************************

baochun2 ggccgtattaaaaaaaaatttcaaggaattctaatttataaattgaaatatttttattct

baochun1 ggccgtattaaaaaaaaatttcaaggaattctaatttataaattgaaatatttttattct

************************************************************

baochun2 ttcaaacccatatttatgcttattttgacttaaagccaaaggttttttattttgagtcat

baochun1 ttcaaacccatatttatgcttattttgacttaaagccaaaggttttttattttgagtcat

************************************************************

baochun2 tctatctatttaagcaataagcaatgatccaaaagttcatactcatggaattcttgggct

baochun1 tctatctattcaagcaataagcaatgatccaaaagttcatactcatggaattcttgggct

**********.*************************************************

baochun2 tagtttagggtttttattgaatcattgtggttctagtatgaatctcaggttttaattgat

baochun1 tagtttagggtttttattgaatcattgtggttctagtatgaatctcaggttttaattgat

************************************************************

baochun2 ttatagggtcttaacaagagaattcctatcaataataaataaaaaaagagtaaaactgca

baochun1 tcatagggtcttaacaagagaattcctatcaataataaataaaaaaagagtaaaactgca

*.**********************************************************

baochun2 ttatgcacaaattaagaatcgaagaaaaaaaaaaataggaaaaaagagaattcaagaggc

baochun1 ttatgcacaaattcagaatcgaagaaaaaaaaaaataggaaaaaagagaattcaagaggc

************* **********************************************

baochun2 ctgtaaggatcaagataaagacaaccgagccaacttgatattttggcattaccacactcc

baochun1 ctataaggatcaagataaagacaaccgagccaacttgatattttggcattaccacactcc

**.*********************************************************

baochun2 aaaaatagctttcggatttttcttatttcatatcttcggagaagattgaatcagagatta

baochun1 aaaaatagctttcggatttttcttatttcatatcttcggagaagattgaatcagagatta

************************************************************

baochun2 aaaagttttaaacttgctattccatacccgttgcaaccagtatttgggtgtttttctgct

baochun1 aaaagtttaaaacttgctattccatatccgttgcaaccagtatttgggtgtttttctgct

******** *****************.*********************************

baochun2 tgagctgtacgagatgaaattctcatatacagttctcagagggggggtcttacctatctc

baochun1 tgagctgtacgagatgaaattctcatatacagttctcagagggggggtcttacctatctc

************************************************************

baochun2 aataaagtttatgattggttcgaagaacgtctcgaaattcaagcgattgcagatgatata

baochun1 aataaagtttatgattggttcgaagaacgtctcgaaattcaagcgattgcagatgatata

************************************************************

baochun2 actagtaaatatgttcctcctcatgtcaatatattttattgtctaggaggaattacgctt

baochun1 actagtaaatatgttcctcctcatgtcaatatattttattgtctaggaggaattacgctt

************************************************************

baochun2 acttgttttttagtacaagtagctacagggtttgctatgactttttactatcgtccaact

baochun1 acttgttttttagtacaagtagctacagggtttgctatgactttttactatcgtccaact

************************************************************

baochun2 gttagtgaggcttttgcgtctgttcaatacataatgactgaagcgaattttggttggtta

baochun1 gttagtgaggcttttgcgtctgttcaatacataatgactgaagcgaattttggttggtta

************************************************************

baochun2 attcgatcagttcatcgatggtcggcaagtatgatggttctaatgatgattttgcatgta

baochun1 attcgatcagttcatcgatggtcggcaagtatgatggttctaatgatgattttgcatgta

************************************************************

baochun2 tttcgtgtgtatctcactggtggatttaaaaaaccccgtgaattgacttgggttacgggt

baochun1 tttcgtgtgtatctcactggtggatttaaaaaaccccgtgaattgacttgggttacgggt

************************************************************

baochun2 gtggttctaggggtattgaccgcatcatttggtgtaactggttattccttgccttgggat

baochun1 gtggttctaggggtattgaccgcatcatttggtgtaactggttattccttgccttgggat

************************************************************

baochun2 caaattggttattgggcagtaaaaattgtaacaggggtacctgacgctattcctgtaata

baochun1 caaattggttattgggcagtaaaaattgtaacaggggtacctgacgctattcctgtaata

************************************************************

baochun2 ggcgcgcctttggtagaattattgcggggaagcgctagtgtgggacaatccactttgact

baochun1 ggcgcgcctttggtagaattattgcggggaagcgctagtgtgggacaatccactttgact

************************************************************

baochun2 cggttttatagtttacacacttttgtattgcctcttcttactgccgtatttatgttaatg

baochun1 cggttttatagtttacacacttttgtattgcctcttcttactgccgtatttatgttaatg

************************************************************

baochun2 cacttcccaatgatacgtaaacaaggcatttctggtcctttatagagagggtatatcata

baochun1 cacttcccaatgatacgtaaacaaggcatttctggtcctttatagagagggtatatcata

************************************************************

baochun2 aatatttttaatcaatcatttatcacttttgggaggaacaatactattttattgctacaa

baochun1 aatatttttaatcaatcatttatcacttttgggaggaacaatactattttattgctacaa

************************************************************

baochun2 gtatggattattgaaaagaataatacatgtatttggatatttcccttcaaccccacaata

baochun1 gtatggattattgaaaagaataatacatgtatttggatatttcccttcaaccccacaata

************************************************************

baochun2 ttacttagttgattgaatagttgaagagaattctccgaagagaaaatggattatgggagt

baochun1 ttacttagttgattgaatagttgaagagaattctccgaagagaaaatggattatgggagt

************************************************************

baochun2 gtgtgacttgaactattgattggtccgtgcagaaatgtccttatctgccacattgaaatt

baochun1 gtgtgacttgaactattgattggtccgtgcagaaatgtccttatctgccacattagaatt

******************************************************..****

baochun2 aacaagcaaatgtgtctttgttccaaccaccgtgtaagccccatacagaggataggctgg

baochun1 aacaagcaaatgtgtctttgttccaaccaccgtgtaagccccatacagaggataggctgg

************************************************************

baochun2 ttcagttgaagagaatcttttctatgatcagatccgaatcatgtcgtacatgagcaggct

baochun1 ttcagttgaagagaatcttttctatgatcagatccgaatcatgtcgtacatgagcaggct

************************************************************

baochun2 ccgtaagacccagtagaataagtgaaatagataacccaaaactttgggttatctatttca

baochun1 ccgtaagatccagtagaataagtgaaatagataaccccaagttttgggttatctatttca

********.**************************** **..******************

baochun2 cttaatttaagattgaatagtatggaaatgcattcatttcctatgcattgacacgatcta

baochun1 cttaatttaagattgaatagtatggaaatgcattcatttcctatgcattaacacgatcta

*************************************************.**********

baochun2 tgatactatcggagtgaaacacgggatctaaagaaaaaaataaagctagactatattaat

baochun1 tgatactatcggagtgaaacacgggatctaaagaaaaaaataaagctagactatattaat

************************************************************

baochun2 aacaagtaaactctttgtatgttgtgtagggaaaaggtatttcccatttttttttggggg

baochun1 aacaagtaaactctttgtatgttgtgtagggaaaaggtatttcccattttttttt--tgg

******************************************************* **

baochun2 gggggggtataagcatcagttgcaaggtctgagacgacccagaaagcacttgatcatgat

baochun1 gggggggtataagcatcagttgcaaggtctgagacgacccagaaagcacttgatcatgat

************************************************************

baochun2 atgatcaattttgtaagcctacttgggtattgagtatttatttgtaagaacttcattttt

baochun1 atgatcaattttgtaagcctacttgggtattgagtatttatttgtaagaacttcattttt

************************************************************

baochun2 tacaataaaataaaatctggtaaaatgtttcttattatggaaacatttatttatatatat

baochun1 tacaataaaataaaatctggtaaaatgtttcttattatggaaacatttatt--tatatat

*************************************************** *******

baochun2 ttatatattatattatgtgcatatatgcgtgcacacgcatatatgtgtagatatcatata

baochun1 ttatatattatattatgtgcatatatgcgtgcacacgcatatatgtgtagataccatata

*****************************************************.******

baochun2 tattttctaacatatttattttatatggatccatttagttctttctagtttttgctcgag

baochun1 gattttctaacatatttattttatatggatccatttagttctttctagtttttgctcgag

***********************************************************

baochun2 ccggatgatgaaaaattatcatgtccggttccgtcgggggatggatctataagaattcac

baochun1 ccggatgatgaaaaattatcatgtccggttccgtcgggggatggatctataagaattcac

************************************************************

baochun2 ctatcccaataacaaaaaaacctgacttgaatgatcctgtattaagagctaaattggcta

baochun1 ctatcccaataacaaaaaaacctgacttgaatgatcctgtattaagagctaaattggcta

************************************************************

baochun2 aaggcatgggtcataattattacggagagcccgcatggcccaacgatcttttatatattt

baochun1 aaggcatgggtcataattattacggagagcccgcatggcccaacgatcttttatatattt

************************************************************

baochun2 ttccagtagtcattttcggtactattgcgtgtaacgtaggtttagcggttctcgaaccat

baochun1 ttccagtagtcattttcggtactattgcgtgtaacgtaggtttagcggttctcgaaccat

************************************************************

baochun2 caatgattggtgaaccagcggatccatttgcaactcccctggaaatattacctgaatggt

baochun1 caatgattggtgaaccagcggatccatttgcaactcccctggaaatattacctgaatggt

************************************************************

baochun2 atttctttcccgtatttcaaatacttcgaacagtacccaataaattattgggtgttcttt

baochun1 atttctttcccgtatttcaaatacttcgaacagtacccaataaattattgggtgttcttt

************************************************************

baochun2 taatggcttcagtacctgctggattattaacagttccttttttagagaatgttaataaat

baochun1 taatggcttcagtacctgctggattattaacagttccttttttagagaatgttaataaat

************************************************************

baochun2 ttcaaaatccatttcgtcgtccagtagcgacaactgtctttttgattggtacggcggcgg

baochun1 ttcaaaatccatttcgtcgtccagtagcgacaactgtctttttgattggtacggcggcgg

************************************************************

baochun2 ccctttggttgggtattggtgcaacattacctattgataaatccctaactttaggtcttt

baochun1 ccctttggttgggtattggtgcaacattacctattgataaatccctaactttaggtcttt

************************************************************

baochun2 tttaaattgattcattaattgtgaaataaaatattacgacgtgtgtatctagggaataat

baochun1 tttaaattgattcattaattgtgaaataaaatattacgacgtgtgtatctagggaataat

************************************************************

baochun2 ctcttcaaagtgacttatccctagatacacctaacgttcgagtacaaatttatatatagg

baochun1 ctcttcaaagtgacttatccctagatacacctaacgttcgagtacaaatttatatatagg

************************************************************

baochun2 ttgtgtaaagatcgaaaaaaacattttcatcttgttttgtaaacaaaaagatgaaataaa

baochun1 ttgtgtaaagatctaaaaaaacattttcatcttgttttgtaaacaaaaagatgaaataaa

************* **********************************************

baochun2 tccaacggattaaaaactaatttttcagtaaatccatttcaaactgcttttctagaatgc

baochun1 tccaacggattaaaaactaatttttcagtaaatccatttcaaactgcttttctagaatgc

************************************************************

baochun2 ccaatatctgttttacatcttctatacgaaaatgttctattttcataagatcctcttgag

baochun1 ccaatatctgttttacatcttctatacgaaaatgttcaattttcataagatcctcttgag

************************************* **********************

baochun2 ttttattcaaaagatctaataatgtatatatattggatcttttgaggcaattatagattc

baochun1 tgttattcaaaagatctaataatgtatatatattggatcttttgaggcaattatagattc

* **********************************************************

baochun2 tgggaggcaattttaattggtcaataaaaatatatttcaatgctattttttttttgtttt

baochun1 tgggaggcaattttaattggtcaataaaaatatatttcaatgctattttttttttgtttt

************************************************************

baochun2 tctttagtttaaccaatttatcatgaaaggtaaaaagaggtaaagtaaccttgtgtttat

baochun1 tctttagtttaaccaatttatcatgaaaggtaaaaagaggtaaagtaaccttgtgtttat

************************************************************

baochun2 tgtcgtctaaatttaagttttcttcttcttctataaatataaagggaataaataaatcaa

baochun1 tgtcgtctaaatttaagtt---ttcttcttctatatggaaaaagggaataaataaatcaa

******************* ************* . * ********************

baochun2 tcaaattccgggacgcttcatgaagcgcttctttaggagttaaacttccatttgtccata

baochun1 tcaaattccgggacgcttcatgaagcgcttctttaggagttaaacttccatttgtccata

************************************************************

baochun2 tttcgagaaaaagcatctcttgtttttcatttccattcccataagaatgaatactatgat

baochun1 tttcgagaaaaagcatctcttgtttttcatttccattcccataagaatgaatactatgat

************************************************************

baochun2 ttgcatttcgaacaggcatgaatacagcatcgataggataacttccgccttgaaagttag

baochun1 ttgcatttcgcacaggcatgaatacagcatcgataggataacttccgccttgaaagttag

********** *************************************************

baochun2 tttcactttttatacgatatcctcgactcctctcaatttgtaatccaatataaaaatcaa

baochun1 tttcactttttatacgatatcctcgactcctctcaatttgtaatccaatataaaaatcaa

************************************************************

baochun2 taggttctgtcaagttagctatatgttgtgtattatcaacgatttccaaataaggtggtg

baochun1 taggttctgtcaagttagctatatgttgtgtattatcaacgatttccaaataaggtggtg

************************************************************

baochun2 aaattatatcttgagctgttacatacccaggacccttaacacaaatagacgcatcacaag

baochun1 agattatatcttgagctgttacatacccaggacccttaacacaaatagacgcatcacaag

*.**********************************************************

baochun2 ttccatagaaattacttctcaatactatttctttcaaattcattaaaatttcatgtactg

baochun1 ttccatataaattacttctcaatactatttctttcaaattcattaaaatttcatgtactg

******* ****************************************************

baochun2 attcttgaatacctactatggtagaatattcgtggggtattttctcagattttgcgcgtg

baochun1 attcttgaatacccactatggtagaatattcgtggggtattttctcagattttgcgcgtg

*************.**********************************************

baochun2 tgatacatgttccttctatttctccaagtaaagctcttcgcatcgcaatgcctacggtgt

baochun1 tgatacatgttccttctatttctccaagtaaagctcttcgcatcgcaatgcctatggtgt

******************************************************.*****

baochun2 cggcttgacctttcataagtggagacaaaagaaagcgtccataataaagacgcttactgt

baochun1 cggcttgacctttcataagtggagacaaaagaaagcgtccataataaagacgcttactgt

************************************************************

baochun2 ctgtccttgattcaacacacttccactgtagtgtccgagtagatactgttactttctctc

baochun1 ctgtccttgattcaacacacttccactgtagtgtccgagtagatactgttactttctctc

************************************************************

baochun2 gaaccatagtaatataatattatttgatcgaatcgtttatttctcttgaaatttctttaa

baochun1 gaaccatagtaatataatattatttgatcgaatcgtttatttctcttgaaatttctttaa

************************************************************

baochun2 tattttttttttacacgcgtctttttttaggaggtctacagccattatgtggcataggag

baochun1 tattttttttttacacgcgtctttttttaggaggtctacagccattatgtggcataggag

************************************************************

baochun2 ttacatcccggacgaaagttaatagtataccacttcgacgaatagcccgtaatgctgcat

baochun1 ttacatcccggacgaaagttaatagtataccacttcgacgaatagcccgtaatgctgcat

************************************************************

baochun2 ctcttccgagaccgggtccttttatcatgacttctgctcgttgcataccttgatcgacta

baochun1 ctcttccgagaccgggtccttttatcatgacttctgctcgttgcataccttgatcgacta

************************************************************

baochun2 ctgtacgaatagcatttccagctgccgtttgagcagcaaaaggtgtccctcttcttgtac

baochun1 ctgtacgaatagcatttccagctgccgtttgagcagcaaaaggtgtccctcttcttgtac

************************************************************

baochun2 cccgaaatccacaagtaccggccgaagaccaagaaaccacccgacctcttatatctgtaa

baochun1 cccgaaatccacaagtaccggccgaagaccaagaaaccacccgacctcttatatctgtaa

************************************************************

baochun2 cagtcacaatggtattattgaaacttgcttgaacatgaataactccctttggtattctac

baochun1 cagtcacaatggtattattgaaacttgcttgaacatgaataactccctttggtattctac

************************************************************

baochun2 gtgtattcttacgtgaaccaatacgtccattcctacgcgaacccatttttggtatagttt

baochun1 gtgtattcttacgtgaaccaatacgtccattcctacgcgaacccatttttggtatagttt

************************************************************

baochun2 ttgccatattttatcatctcataaatataagtcatatagatatatggatatatccatttc

baochun1 ttgccatattttatcatctcataaatataagtcatatagatatatggatatatccatttc

************************************************************

baochun2 ttgtcaaaataaatcttttttatttgtacatcgggtcttttagagagtcttttttacact

baochun1 ttgtcaaaacaaatcttttttatttgtacatcgggtcttttagagagtcttttttacact

*********.**************************************************

baochun2 atccctgtctttgtttatgtctcgggttggaacaaattactataattcgtccccgtctac

baochun1 atccctgtctttgtttatgtctcgggttggaacaaattactataattcgtccccgtctac

************************************************************

baochun2 gaattagtcgacatttttcacaaattttacgaacagaagctcttattttcatatttttaa

baochun1 gaattagtcgacatttttcacaaattttacgaacagaagctcttattttcatatttttaa

************************************************************

baochun2 ttccttcaacttaattttgaatcgtagtcccacggaaactaatgttaaagttgaaaaaga

baochun1 ttccttaaacttcattttgaatcgtagtcccacggaaactaatgttaaagttgaaaaaga

****** ***** ***********************************************

baochun2 aaaaccaccgaatccctcgaatctttgttggggagttgataaatgatacgccctctggtt

baochun1 aaaaccaccgaatccctcgaatctttgttggggagttgataaatgatacgccctctggtt

************************************************************

baochun2 gaatcagaatgccttactttaattttgactctacctcctggtagtatctgtataaaacta

baochun1 gaatcagaatgccttactttaattttgactctacctcctggcagtatttgtataaaacta

*****************************************.*****.************

baochun2 tgccggatctttcctaaaatataacctagattaagatcttcattatctaaaggagctcgg

baochun1 tgccggatctttcctgaaatataacctagattaagatcttcattatctaaaggagctcgg

***************.********************************************

baochun2 aacatatcatttggaagcatttccgtaattaaaccctcatgaatctatttttgttcttta

baochun1 aacataccatttggaagcatttccgtaattaaaccctcatgaatctatttttgttcttta

******.*****************************************************

baochun2 tattccaggtaaaacccccttaaattagtaattaatgtaggaggaacaagattatatact

baochun1 tattccaggtaaaacccccttaaattagtaattaatgtaggaggaacaagattatatact

************************************************************

baochun2 ccgcatttttttttgtttttttcacaacaaatagaaagtttcggatccaatgcagatata

baochun1 ccgcatttttttttgtttttttcacaacaaatagaaagtttcggatccaatgcagatata

************************************************************

baochun2 agaaggattaccatatataacacaaaatttctccgcctattccttttagtcgagcctccc

baochun1 agaaggattaccatatataacacaaaatttctccgcctattccttttagtcgagcctccc

************************************************************

baochun2 gatctgtcattataccatgagaagtagaaagaatgacaacgcccattccacccaaaattc

baochun1 gatctgtcattataccatgagaagtagaaagaatgacaacgcccattccacccaaaattc

************************************************************

baochun2 taggaattctttgatagttcgaatagattcgtagaccaggtctactgatccgttttaaat

baochun1 taggaattctttgatagttagaatagattcgtagaccaggtctactgatccgttttaaat

******************* ****************************************

baochun2 ttaaaagatttctataaggcccctttctatttcttgtatgtcgcagggttgaaaccaaaa

baochun1 ttaaaagatttctatagggcccctttctatttcttgtatgtcgcagggttgaaaccaaaa

****************.*******************************************

baochun2 aatatttgtcgctttctcgatgtttcctcacattttcgataaaaccttctcgtaaaagga

baochun1 agtatttgtcgctttctcgatgtttcctcacattttcgataaaaccttctcgtaaaagga

*.**********************************************************

baochun2 ttttcacaatgttttcagtgatattagtagatgctattcgaactactctttttttatcca

baochun1 ttttcacaatgttttcagtgatattagtagatgctattcgaactactctttttttatcca

************************************************************

baochun2 tatccgcattgcgtatagaggttagtatatcagcaatagtgtccctactcatgatggact

baochun1 tatccgcattgcgtatagaggttagtatatcagcaatagtgtccctactcatgatggact

************************************************************

baochun2 aaaattcttgttgcccccgaatttttatataatcaacatgtttttttacttaattttttt

baochun1 aaaattcttgttgcccccgaatttttatacaatcaacatgtttttttacttaattttttt

*****************************.******************************

baochun2 tgtttatgaaaaaaaattatattattaaattaaaggtatatgcgtaaaacacaatctact

baochun1 tgtttatg-aaaaaaattatattattaaattaaaggtatatgcgtgaaacacaatctact

******** ************************************.**************

baochun2 aaattaatttgaatctatttctttccaatacccgactataattatatcatagtctcatct

baochun1 aaattaatttgaatctatttctttccaatacccgactataactatatcatagtctcatct

*****************************************.******************

baochun2 tatattttaaggctattataatacctcgggcgccaatgaaactattttagtaaaatttaa

baochun1 tatattttaaggctattataatacctcgggcgccaatgaaactattttagtaaaatttaa

************************************************************

baochun2 atgtctcaattcccgggcgatcgcaccaaaaacgcgagttcctttaggatttccttcttg

baochun1 atgtctcaattcccgggcgatcgcaccaaaaacgcgagttcctttaggatttccttcttg

************************************************************

baochun2 atcaataacaactgcggcattgtcatcatatcgtattagcataccattgtcgcgtttaag

baochun1 atcaatgacaactgcggcattgtcatcatatcgtattagcataccattgtcgcgtttaag

******.*****************************************************

baochun2 ttctttgcaggtacgtacaattacagctctgaccacttctgatctttctaggggcatgtt

baochun1 ttctttgcaggtacgtacaattacagctctgaccacttctgatctttctaggggcatgtt

************************************************************

baochun2 tggtactgcttctttaatcacagcaacaataacatcaccgatataagcatatcggcggtt

baochun1 tggtactgcttctttaatcacagcaacaataacatcaccgatataagcatatcggcggtt

************************************************************

baochun2 actagctcctatgattcgaatacacatcaattctcgagccccactgttatctgctacatt

baochun1 actagctcctatgattcgaatacacatcaattctcgagccccactgttatctgctacatt

************************************************************

baochun2 caaacgtgtctggggttgaatcatttttttatttgttctttcaatgcaaagggcgaagaa

baochun1 caaacgtgtctggggttgaatcatttttttatttgttctttcaatgcaaagggcgaagaa

************************************************************

baochun2 aaagaaagaaatattttttgtcaaaaaaaaaataaaccttgcattttttattcccaggat

baochun1 aaagaaagaaatattttttgtcaaaaaaaaaataaaccttgc-------attcccaggat

****************************************** ***********

baochun2 ttattttctttgattctacattcttatcccaaaataataaattgagttttgataggcatt

baochun1 ttattttctttgattctacattcttatcccaaaataataaattgagttttgataggcatt

************************************************************

baochun2 ttggatgctgctattgaaatggcttttctggctatattttctgcgactccacccatttca

baochun1 ttggatgctgctattgaaatggcttttctggctatattttctgcgactccacccatttca

************************************************************

baochun2 taaagtattcggcctggtttaacaacagctacccaatattctggagagcctttacctgaa

baochun1 taaagtattcggcctggtttaacaacagctacccaatattctggagagcctttacctgaa

************************************************************

baochun2 cccatacgtgtttctgtgggtcttactgtaactggtttgtcgggaaatatacgtacccat

baochun1 cccatacgtgtttctgtgggtcttactgtaactggtttgtcgggaaatatacgtacccat

************************************************************

baochun2 atttttccaccccgacgtgcatttcgtgtcattgcccggcgccccgcttctatttgtcta

baochun1 atttttccaccccgacgtgcatttcgtgtcattgcccggcgccccgcttctatttgtcta

************************************************************

baochun2 gatgtgatccaagcgggttcaagtgcttgaagagcatatttaccgaaactaatatgatta

baochun1 gatgtgatccaagcgggttcaagcgcttgaagagcatatttaccgaaactaatatgatta

***********************.************************************

baochun2 cctcgataagacattcccttcattcgtcctctatgttgtttacggaatctggttcttttg

baochun1 cctcgataagacattcccttcattcgtcctctatgttgtttacggaatctggttcttttg

************************************************************

baochun2 gggttatagttgatggttctttctgaattccacctctactacagaaccggacatgagaat

baochun1 gggttatagttgatggttctttctgaattccacctctactacagaaccggacgtgagagt

****************************************************.*****.*

baochun2 ttcgtctcatccagctcctcgcgaaaaggggattcaaaatatttaaaatgtagcaatcca

baochun1 ttcgtctcatccagctcctcgcgaaaaggggattcaaaatatttaaaatgtagcaatcca

************************************************************

baochun2 aaaaagaatgttttcgcgggcgaatatttactctactatctatttcagttgtaaggttaa

baochun1 aaaaagaatgttttcgcgggcgaatatttactctactatctatttcagttgtaaggttaa

************************************************************

baochun2 ttcattacgtctcagatcagaatagatgaattctttctcggttccttccgccatcccgac

baochun1 ttcattacgtctcagatcagaatagatgaattctttctcggttccttccgccatcctgac

********************************************************.***

baochun2 caatgaatcgttaggatttggtttcaataaaatcttctgtattcatgggttccatcgttc

baochun1 caatgaatcgttaggatttggtttcaataaaatcttctgcattcatgggttccctcgttc

***************************************.************* ******

baochun2 ccatcgcttcttgtttaatggttaggtcttaatgttacaacggagctcttaatgaaattt

baochun1 ccatcgcttcttgtttaatggttaggtcttaatgttacaacggagctcttaatgaaattt

************************************************************

baochun2 gttcttgagtcaattttctcagtctttattggctaaaggctcctggttttttgttctata

baochun1 gttcttgagtcaattttctcagtctttattggctaaaggctcctggttttttgttctata

************************************************************

baochun2 atgtatcatttaattatgtatgaatcagtattgatgctttattacattgtcttttatgag

baochun1 atgtatcataaaattatgtatgaatcagtattgatgctttattacattgtcttttatgag

********* *************************************************

baochun2 atgactaaatgactcatagaccttacatattggaattttatatcatttata-tttttttc

baochun1 atgactaaatgactcatagaccttacatattggaattctatatcatttatattttttttc

*************************************.************* ********

baochun2 tctctttctctcacccctctatccacatctttttctatattccggttcacaccttagaat

baochun1 tctctttctctcacccctctatccacatctttttctatattccggttcacaccttagaat

************************************************************

baochun2 aatattttttgcttttttagtttatgcaaaacaaatttttagtttatgcaaaacaaattt

baochun1 aatattttttgct----------------------tttttagtttatgcaaaacaaattt

************* *************************

baochun2 cagttgctacaatgatatgaaaaatttatcatatcttgactgctttgttggatctagata

baochun1 cagttgctacaatgatatgaaaaatttatcatatcttgactgctttgttggatctagata

************************************************************

baochun2 atgtgaagtgatgagttggttcttagttttatagttattagttcatattagggaggcttt

baochun1 atgtgaagtgatgagttggttcttagttctatagttattagttcatattagggaggc-tt

****************************.**************************** **

baochun2 tttttttgaaccttattcctaaaaaaaccaacgagtcacacactaagcatagcaattata

baochun1 tttttttgaaccttattcctaaaaaaaccaacgagtcacacactaagcatagcaattatc

***********************************************************

baochun2 tcaaacatttatttaatcgaatttttattaaaccctatagaattaaagaattacgagctc

baochun1 tcaaacatttatttaatcgaatttttattgaaccctatagaattaaagaattacaagctc

*****************************.************************.*****

baochun2 ttttttttattcttcatctataaatatccaaattttgatacctaatacgccatagatagt

baochun1 ttttttttattcttcatctataaatatccaaattttgatacctaatacgccatagatagt

************************************************************

baochun2 tcgaactgtataggcacaataatcaattttagcccgaatggtttgtaggggaaccctacc

baochun1 tcgaactgtataggcacaataatcaattttagcccgaatggtttgtaggggaaccctgcc

*********************************************************.**

baochun2 ttctctgatccattcgacgcgcgcaatttcttttccatcaatgcgccctgcaatttgtac

baochun1 ttctctgatccattcgacgcgtgcaatttcttttccatcaatgcgccctgcaatttgtac

*********************.**************************************

baochun2 ttgaattccttttgtatttgcttgttcggttaattcaatagcctttttcattgctttgcg

baochun1 ttgaattccttttgtatttgcttgttcggttaattcaatagcctttttcattgctttgcg

************************************************************

baochun2 acaagaaactctattttttaattgtccggctataaattctgcaagaatattaggatttcc

baochun1 acaagaaactctattttttaattgtccggctataaattctgcaagaatattaggatttcc

************************************************************

baochun2 ataaggctttgcaattcttgtgatagtaatattgagttttcggtttacacagttaaactc

baochun1 ataaggctttgcaattcttgtgatagtaatattgagttttcggtttacacagttaaactc

************************************************************

baochun2 tttttgtagattcgtctgtaattcttcgattccttgtggccgattttcgattaataattt

baochun1 tttttgtagattcgtctgtaattcttcgattccttgtggccgattttcgattaataattt

************************************************************

baochun2 aggaaatcccatatagattatgacctggatcagatcgattcttttttgaatctctatacg

baochun1 aggaaatcccatatagattatgatctggatcagatcgattcttttttgaatctctatacg

***********************.************************************

baochun2 tgcaattccctcgatgctggaggatattctcatattcttttgtacataatttttgataca

baochun1 tgcaattccctcgatgccggaggatattctcatattcttttgtacataatttttgataca

*****************.******************************************

baochun2 atctcttatttttttatcttcttctaaaccttcggaatagttttttggttgtgaaaacca

baochun1 atctcttatttttttatcttcttctaaaccttcggaatagttttttggttgtgaaaacca

************************************************************

baochun2 aacggaatgatgtctttgggttgtaccaagtcggaaaccaagtggatttattttttgccc

baochun1 aacggaatgatgtctttgggttgtaccaagtcggaaaccaagtggatttattttttgccc

************************************************************

baochun2 catattcccccgcgacttttattatacacactctgatgtttcatcgtcatatcttcatat

baochun1 catattcccccgcgacttttattatacacactctgatgtttcatcgtcatatctttatat

*******************************************************.****

baochun2 tcatctaaagatatatctttcactccaatagttatatgacaagtaggtctttttattgga

baochun1 tcatctaaagatatatctttcactccaatagttatatgacaagtaggtctttttattgga

************************************************************

baochun2 aaactacgtcctcgagctcgaggacgtagtttcttcacggtagtgccttcgttgacttca

baochun1 aaactacgtcctcgagctcgaggacgtagtttcttcacggtagtgccttcgttgacttca

************************************************************

baochun2 gctttactaattattaaattagtttcgttggaacccatgttgtaactagcatttgctgct

baochun1 gctttactaattattaaattagtttcgttggaacccatgttgtaactagcatttgctgct

************************************************************

baochun2 gcagaataaaccaatttaaaaatagaataacatgctcgatagggcatcagttctagtatc

baochun1 gcagaataaaccaatttaaaaatagaataacatgctcgatagggcatcagttctagtatc

************************************************************

baochun2 ataagtgtttcctcataggaacgtccacgaatttgatcaataactcttcgcgctttatga

baochun1 ataagtgtttcctcataggaacgtccacgaatttgatcaatgactcttcgcgctttatga

*****************************************.******************

baochun2 gccgacatagatatatgttgacctaaagcatatatttcggtttttttcttctttatcata

baochun1 gccgacatagatatatgttgacctaaagcatatatttcggtttttttcttctttatcata

************************************************************

baochun2 agattcgcctcctactaatgaatgataagtatctatattttttattaacgacgagagcga

baochun1 agattcgcctcctactaatgaatgataagtatctatattttttattaacgacgagagcgg

***********************************************************.

baochun2 ttatctccttttgtatgccctcgaaaatttaaagtaggtgaaaattctcccaatttgtga

baochun1 ttatctccttttgcatgccctcgaaaatttaaagtaggtgaaaattctcccaatttgtga

*************.**********************************************

baochun2 cctatcatacggtctgttatataaataggtacatgctcttttccattatggatcccaata

baochun1 cctatcatacggtctgttatataaataggtacatgctcttttccattatggatcccaata

************************************************************

baochun2 gtatggccgatcattgtaggtataatggtcgatgctcgggaccaagttactattatttct

baochun1 gtatggccgatcattgtaggtataatggtagatgctcgggaccaagttactattatttct

***************************** ******************************

baochun2 ttttctgcttttgtgttaagcttctctatttttcttaacaaatgctttgctacaaaaggg

baochun1 ttttctgcttttgtgttaagcttctctatttttcttaacaaatgctttgctacaaaaggg

************************************************************

baochun2 ttttttttgagtgaacgtgtcacaacttactccttttttcttttggtaaagacgaagaaa

baochun1 ttttttttgagtgaacgtgtcacaacttactccttttttcttttggtaaagacgaagaaa

************************************************************

baochun2 gaaattcgat-----------tttctctcctatttactacggcgacgaagaatcaaatta

baochun1 gaaattcgattttctctcctatttctctcctatttactacggcgacgaagaatcaaatta

********** ***************************************

baochun2 tcgctatatttattcctttttctacttcttcttccaagtgcaggataaccccaaggggtt

baochun1 tcgctatatttattcctttttctacttcttcttccaagtgcaggataaccccaaggggtt

************************************************************

baochun2 gtgggtttttttctaccaattggggctctcccttcaccacccccatggggatggtctaca

baochun1 gtgggtttttttctaccaattggggctctcccttcaccacccccatggggatggtctaca

************************************************************

baochun2 gggttcataactactcctcttactacaggacgcttacctagccaacgcttcgatccggct

baochun1 gggttcataactactcctcttactacaggacgcttacctagccaacgcttcgatccggct

************************************************************

baochun2 ctacccagacttttctggttcaccccaacattccccacttgtccgactgttgctgagcag

baochun1 ctacccagacttttctggttcaccccaacattccccacttgtccgactgttgctgagcag

************************************************************

baochun2 tttttggatatcaaacggacctccccagaaggtaattttaatgtggccgatttcccctct

baochun1 tttttggatatcaaacggacctccccagaaggtaattttaatgtggccgatttcccctct

************************************************************

baochun2 tttgcaatcagtttcgctacagcaccagctgctctaactaattgtccaccctttccaagt

baochun1 tttgcaatcagtttcgctacagcaccagctgctctaactaattgtccaccctttccaagt

************************************************************

baochun2 gtgatttctatgttatgtatggccgtgcctaagggcatatcggttgaagtagattcttct

baochun1 gtgatttctatgttatgtatggccgtgcctaagggcatatcggttgaagtagattcttct

************************************************************

baochun2 ttttgatcaatcaaaaccccttcccaaactgtacaagcttcttccaaagcatacggcttt

baochun1 ttttgatcaatcaaaaccccttcccaaactgtacaagcttcttccaaagcatacggcttt

************************************************************

baochun2 ctggatgtagatgatgatatctatacagatggattttctatatatggtacactgaagtac

baochun1 ctggatgtagatgatgatatctatacagatggattttctatatatggtacactgaagtac

************************************************************

baochun2 cacatgagtggatatataggaatcaaaatatgccgaatcactcatgttatgatcttctaa

baochun1 cacatgagtggatatataggaatcaaaatatgccgaatcactcatgttatgatcttctaa

************************************************************

baochun2 atcctaggttttcccgttccgtcatctggcttatgttcttcatgtagcattcagaccgaa

baochun1 atcctaggttttcccgttccgtcatctggcttatgttcttcatgtagcattcagaccgaa

************************************************************

baochun2 tgactctatgaaattacgtcgatacttccacatattattgtattggtaacgtaggagact

baochun1 tgactctatgaaattacgtcgatacttccacatattattgtattggtaacgtaggagact

************************************************************

baochun2 ttccccgggggaatctttagaattaccactgcttagctttcaattcgcctctgaccatca

baochun1 ttccccgggggaatctttagaattaccactgcttagctttcaattcgcctctgaccatca

************************************************************

baochun2 aatgaaatgtgaataacccgtcctcctctctttgaaacaaggggcgcttccggttctgtc

baochun1 aatgaaatgtgaataacccgtcctcctctctttgaaacaaggggcgcttccggttctgtc

************************************************************

baochun2 ggtgcttgaaacaattttgtcttctccatattactatatctctagagtcaataattttat

baochun1 ggtgcttgaaacaattttgtcttctccatattactatatctctagagtcaataattttat

************************************************************

baochun2 atgaggaactactgaactcaatcacttgctgccgttactcttcagttttctgttgaggtc

baochun1 atgaggaactactgaactcaatcacttgctgccgttactcttcagttttctgttgaggtc

************************************************************

baochun2 tatcctgtagaggtactcaaattggatcagtgatcgatttctaggtttcgtcgtaaacct

baochun1 tatcctgtagaggtactcaaattggatcagtgatcgatttctaggtttcgtcgtaaacct

************************************************************

baochun2 aattggttacttccaattacgtaaatcaatagttcaaaccgcactcaaaggtagggcatt

baochun1 aattggttacttccaattacgtaaatcaatagttcaaaccgcactcaaaggtagggcatt

************************************************************

baochun2 tcccatttttataggaacttctgtaccagaaacaatggtatctccaattatagcccctct

baochun1 tcccatttttataggaacttctgtaccagaaacaatggtatctccaattatagcccctct

************************************************************

baochun2 gggatgtaaaatatatctcttctcaccatccccatagtgtatgagacaaatgtatgcatt

baochun1 gggatgtaaaatatatctcttctcaccatccccatagtgtatgagacaaatgtatgcatt

************************************************************

baochun2 tcgattagggtcgtattctatggttacgattctaccatatatgtttttttcattccgtcg

baochun1 tcgattagggtcgtattctatggttacgattctaccatatatgtttttttcattccgtcg

************************************************************

baochun2 aaaatcgattttacggtatagacgcttatgacctccccctctatgccctgcggtaatgat

baochun1 aaaatcgattttacggtatagacgcttatgacctccccctctatgccctgcggtaatgat

************************************************************

baochun2 tcctctggaattacgacctttaccacaatgatgctgtccatagatcaaattatttcgtgg

baochun1 tcctctggaattacgacctttaccacaatgatgctgtccatagatcaaattatttcgtgg

************************************************************

baochun2 attggatttcacttgactgtctacggttccattgcgtatgctgcggttagaagttttgta

baochun1 attggatttcacttgactgtctacggttccattgcgtatgctgcggttagaagttttgta

************************************************************

baochun2 taaatgtatcgccatgctattaagtattttgatttaagttcttttctttctaagaggtgg

baochun1 taaatgtatcgccatgctattaagtattttgatttaagttcttttctttctaagaggtgg

************************************************************

baochun2 aatagaataacccggttgaagcgtaatgatcatacgtctgtaatgcattgtatgccctct

baochun1 aatagaataacccggttgaagcgtaatgatcatacgtctgtaatgcattgtatgccctct

************************************************************

baochun2 aataggtcccattcttctcccctttcccggaagtcgatgactattcatagctattacctt

baochun1 aataggtcccattcttctcccctttcccggaagtcgatgactattcatagctattacctt

************************************************************

baochun2 gacaccaaagaagagttcgacccaatgctttatttctgtcctagttgatcctgattcgac

baochun1 gacaccaaagaagagttcgacccaatgctttatttctgtcctagttgatcctgattcgac

************************************************************

baochun2 attagaagtatattgatttttccccaataaccgaatacttttgtctgtaaatactgcata

baochun1 attagaagtatattgatttttccccaataaccgaatacttttgtctgtaaatactgcata

************************************************************

baochun2 tttgattccatccataaatctattttcttccctatgagttctagtctcaataagaatgct

baochun1 tttgattccatccataaatctattttcttccctatgagttctagtctcaataagaatgct

************************************************************

baochun2 agttcttactgttcatatattatgttatgatatgaatataccacacctattcgttatgta

baochun1 agttcttactgttcatata-----ttatgatatgaatataccacacctattcgttatgta

******************* ************************************

baochun2 tggatgatgagattccattgatacagagccaattccaatagacttattggagggtcccat

baochun1 tggatgatgagattccattgatacagagccaattccaatagacttattggagggtcccat

************************************************************

baochun2 tggcgtgcatccagtaggaattgaacctacgaattcgccaattatgagttgggcgcttta

baochun1 tggcgtgcatccagtaggaattgaacctacgaattcgccaattatgagttgggcgcttta

************************************************************

baochun2 accattcagccatggatgcttagcggggatcctcgtacatggtgaataaccaaattccaa

baochun1 accattcagccatggatgcttagcggggatcctcgtacatggtgaataaccaaattccaa

************************************************************

baochun2 ttgaaatgaaatctttaggataaatcaatgcaatttaggaggaatcaatgaaaggacatc

baochun1 ttgaaatgaaatctttaggataaatcaatgcaatttaggaggaatcaatgaaaggacatc

************************************************************

baochun2 aattcaaatcctggattttcgaattgagagagatattgagagagatcaagaattctcact

baochun1 aattcaaatcctggattttcgaattgagagagatattgagagagatcaagaattctcact

************************************************************

baochun2 atttcttagattcatggacccaattcaatttagtgggatctttcattcacgtttttttcc

baochun1 atttcttagattcatggacccaattcaatttagtgggatctttcattcacgtttttttcc

************************************************************

baochun2 accaagaacgttttataaaactctttgacccccgaatttggagtatcctactttcacgca

baochun1 accaagaacgttttataaaactctttgacccccgaatttggagtatcctactttcacgca

************************************************************

baochun2 attcacagggttcaacaagcaatcgatatttcacgatcaagggtgtaatactctttgtcg

baochun1 attcacagggttcaacaagcaatcgatatttcacgatcaagggtgtaatactctttgtcg

************************************************************

baochun2 tagcggtccttctatatcgtattaacaatcgaaatatggtcgaaagaaaaaatctctatt

baochun1 tagcggtccttctatatcgtattaacaatcgaaatatggtcgaaagaaaaaatctctatt

************************************************************

baochun2 tgatagggcttcttcctatacctatgaattccattggacccagaaatgatacattggaag

baochun1 tgatagggcttcttcctatacctatgaattccattggacccagaaatgatacattggaag

************************************************************

baochun2 acccaaaggagtcttccaatatcaataggttgattctttccctcctctatcttccaaaag

baochun1 acccaaaggagtcttccaatatcaataggttgattctttccctcctctatcttccaaaag

************************************************************

baochun2 gaaaaaagatctctgagagttgtttcctgaatccgaaagagagtacttgggttctcccaa

baochun1 gaaaaaagatctctgagagttgtttcctgaatccgaaagagagtacttgggttctcccaa

************************************************************

baochun2 taactaaaaagtgtagcatgcctgaatctaactggggttcgcggtggtggaggaactgga

baochun1 taactaaaaagtgtagcatgcctgaatctaactggggttcgcggtggtggaggaactgga

************************************************************

baochun2 tcggaaaaaatagggattcgagttgtaagatatctaatgaaaccgtcgctggaattgaga

baochun1 tcggaaaaaatagggattcgagttgtaagatatctaatgaaaccgtcgctggaattgaga

************************************************************

baochun2 tcttattcaaagagaaagatatcaaatatctggaatttctttttgtatattatatggatg

baochun1 tcttattcaaagagaaagatatcaaatatctggaatttctttttgtatattatatggatg

************************************************************

baochun2 atccgattcgcaaggaccatgattgggaattgtttgatcatctttctctgaggaagaggc

baochun1 atccgattcgcaaggaccatgattgggaattgtttgatcatctttctctgaggaagaggc

************************************************************

baochun2 gaaatagaatcaacttgaattcgggaccgctatttgaaatcttagtgaaacactggattt

baochun1 gaaatagaatcaacttgaattcgggaccgctatttgaaatcttagtgaaacactggattt

************************************************************

baochun2 gttatctcatgtctgcttttcgtgaaaaaataccaatggaagtggagggtttcttcaaac

baochun1 gttatctcatgtctgcttttcgtgaaaaaataccaatggaagtggagggtttcttcaaac

************************************************************

baochun2 aacaaggggctggattaacgattcaatcaaatgatattgagcatgtttcccatctctttt

baochun1 aacaaggggctggattaacgattcaatcaaatgatattgagcatgtttcccatctctttt

************************************************************

baochun2 cgagaaacaagcggtctttttctttacaaaattgtgctcaatttcatatgtggcaattcc

baochun1 cgagaaacaagcggtctttttctttacaaaattgtgctcaatttcatatgtggcaattcc

************************************************************

baochun2 gccaagatctcttcgttagttgggggaagaatctgcacgaatttgattttttgaggaacg

baochun1 gccaagatctcttcgttagttgggggaagaatctgcacgaatttgattttttgaggaacg

************************************************************

baochun2 tatcgagagagaatcggatttggttagacaatgtgtggttggtaaacaaggatcggtttt

baochun1 tatcgagagagaatcggatttggttagacaatgtgtggttggtaaacaaggatcggtttt

************************************************************

baochun2 ttagcaaggtacggaatgtatcgtcaaatattcaatatgattccacaaaatcgagtttcg

baochun1 ttagcaaggtacggaatgtatcgtcaaatattcaatatgattccacaaaatcgagtttcg

************************************************************

baochun2 ttcaattaagggattctaaccaattgaaaggatcttcggatcaatccagagatcatttgg

baochun1 ttcaattaagggattctaaccaattgaaaggatcttcggatcaatccagagatcatttgg

************************************************************

baochun2 attccatgagtaatgaggattcggaatatcacacattgatcaatcaaagagagattcaac

baochun1 attccatgagtaatgaggattcggaatatcacacattgatcaatcaaagagagattcaac

************************************************************

baochun2 aactaaaagaaagatcgattctttgggatccttcctttcttcaaacggaacgaacaaaga

baochun1 aactaaaagaaagatcgattctttgggatccttcctttcttcaaacggaacgaacagaga

********************************************************.***

baochun2 tagaagcagaccgattaccgaaatgcccttctggatattcctcaatggcccggctattca

baochun1 tagaagcagaccgattaccgaaatgcccttctggatattcctcaatggcccggctattca

************************************************************

baochun2 cggaacgtgagaagcagatgattaatcatctgcttccggaagaaatagaagaatttcttg

baochun1 cggaacgtgagaagcagatgattaatcatctgcttccggaagaaatagaagaatttcttg

************************************************************

baochun2 ggaatcctacaagatccatttgttcttttttctctgacagatggtcagagcttcatctgg

baochun1 ggaatcctacaagatccatttgttcttttttctctgacagatggtcagagcttcatctgg

************************************************************

baochun2 gttcgaatcctactgaaaggtccactagagatcagaaattgttgaagaaacaacaagatc

baochun1 gttcgaatcctactgaaaggtccactagagatcagaaattgttgaagaaacaacaagatc

************************************************************

baochun2 tttcttttgtcccttccaggcgatcggaaaataaagaaatggttaatatattcaagataa

baochun1 tttcttttgtcccttccaggcgatcggaaaataaagaaatggttaatatattcaagataa

************************************************************

baochun2 ttacgtatttacaaaataccgtctcaattcatcctatttcatcagatccgggatgtgata

baochun1 ttacgtatttacaaaataccgtctcaattcatcctatttcatcagatccgggatgtgata

************************************************************

baochun2 tggttccgaaggatgaaccggatatggacagttccaataagatttcattcttgaacaaaa

baochun1 tggttccgaaggatgaaccggatatggacagttccaataagatttcattcttgaacaaaa

************************************************************

baochun2 atgatttatttcatctattccatgactggaacaggggaggatacacgttccaccacgatt

baochun1 atgatttatttcatctattccatgactggaacaggggaggatacacgttccaccacgatt

************************************************************

baochun2 ttgaatcagaagagagatttcaagaaatggcagatcgattcactctatcaataactgagc

baochun1 ttgaatcagaagagagatttcaagaaatggcagatcgattcactctatcaataactgagc

************************************************************

baochun2 cgaatctggtgtatcataagggatttgccttttctattgattcctgcggattgaatcaaa

baochun1 cgaatctggtgtatcataagggatttgccttttctattgattcctgcggattgaatcaaa

************************************************************

baochun2 aaaaattcttgaatgaggtattcaacttcagggatgaatcgaaaaagaaatttttattgg

baochun1 aaaaattcttgaatgaggtattcaacttcagggatgaatcgaaaaagaaatttttattgg

************************************************************

baochun2 ctctacctcctattttttatgaagagaatgaatctttttatcgaaggatcaaaaaaaaat

baochun1 ctctacctcctattttttatgaagagaatgaatctttttatcgaaggatcaaaaaaaaat

************************************************************

baochun2 gggcccggatctcctgcgggaatgatttggaagatccaaaaccaaagagagtggtatttg

baochun1 gggcccggatctcctgcgggaatgatttggaagatccaaaaccaaagagagtggtatttg

************************************************************

baochun2 ctagcaacaacataatggaggcagtcaatcaatatagattgatccgaaatctgattgaaa

baochun1 ctagcaacaacataatggaggcagtcaatcaatatagattgatccgaaatctgattgaaa

************************************************************

baochun2 tccaatattgcacctatgggtatataagaaatgtattgaatctattctttttaatgaatc

baochun1 tccaatattgcacctatgggtatataagaaatgtattgaatctattctttttaatgaatc

************************************************************

baochun2 gatccgatcgcaacttcgaatatggaattcaaaggtatcaaataggaaaggatactctga

baochun1 gatccgatcgcaacttcgaatatggaattcaaaggtatcaaataggaaaggatactctga

************************************************************

baochun2 atcatagaactataatgaaatatacgatcaaccaacatttatcgaatttgaaaaagagtc

baochun1 atcatagaactataatgaaatatacgatcaaccaacatttatcgaatttgaaaaagagtc

************************************************************

baochun2 agaagaaatggtttgatcctcttatttttatttctcgaaccgggagatccatgaattggg

baochun1 agaagaaatggtttgatcctcttatttttatttctcgaaccgggagatccatgaattggg

************************************************************

baochun2 atcctgatgcatatagatacaaatggtccaatgggagcaagaatttccaggaacatttgg

baochun1 atcctgatgcatatagatacaaatggtccaatgggagcaagaatttccaggaacatttgg

************************************************************

baochun2 aacatttcggttctgagcagaagagccattttcaagtagtgttcgatcgattacgtatta

baochun1 aacatttcggttctgagcagaagagccattttcaagtagtgttcgatcgattacgtatta

************************************************************

baochun2 atcaatattcgattgattggtctgaagttatcgacaaaaaagatttgtctaagccacttc

baochun1 atcaatattcgattgattggtctgaagttatcgacaaaaaagatttgtctaagccacttc

************************************************************

baochun2 gtttctttttgtccaagtcacttctttttttgtccaagttgcttttctttttgtctaact

baochun1 gtttctttttgtccaagtcacttctttttttgtccaagttgcttttctttttgtctaact

************************************************************

baochun2 cacttccttttttctttttgagtttcgggaatatccccattcataggtccgagatctaca

baochun1 cacttccttttttctttttgagtttcgggaatatccccattcataggtccgagatctaca

************************************************************

baochun2 tctatgaattgaaaggtccgaatgatcaactctgcaatcagttgttagaatcaataggtc

baochun1 tctatgaattgaaaggtccgaatgatcaactctgcaatcagttgttagaatcaataggtc

************************************************************

baochun2 ttcaaatcattcatttgaaaaaatggaaagccttcttattggatgatcatgatacttccc

baochun1 ttcaaatcattcatttgaaaaaatggaaagccttcttattggatgatcatgatacttccc

************************************************************

baochun2 caaaatcgaaattattgatcaacggaggaagaatatcaccattttcgttcaataagatac

baochun1 aaaaatcgaaattattgatcaacggaggaagaatatcaccattttcgttcaataagatac

***********************************************************

baochun2 caaagtggatgattgactcattccatactcgaaagaatcgcaggaaatcctttgataaca

baochun1 caaagtggatgattgactcattccatactcgaaagaatcgcaggaaatcctttgataaca

************************************************************

baochun2 cggattcctatttctcaatgatattccacgatcaagacaattggctgaatcccgtgaaac

baochun1 cggattcctatttctcaatgatattccacgatcaagacaattggctgaatcccgtgaaac

************************************************************

baochun2 tgtttcatagaagttcattgatatcttctttttataaagcaaatcgacttcgattcttga

baochun1 tgtttcatagaagttcattgatatcttctttttataaagcaaatcgacttcgattcttga

************************************************************

baochun2 ataatccacataacttctccttctattgtaacagaagattccctttttatgtggaaaagg

baochun1 ataatccacataacttctccttctattgtaacagaagattccctttttatgtggaaaagg

************************************************************

baochun2 cccgtatcaataattatgattttacgtatggacaattcctcaatatcttgttcattcgca

baochun1 cccgtatcaataattatgattttacgtatggacaattcctcaatatcttgttcattcgca

************************************************************

baochun2 acaaaatattttctttgtgcgtcggtaaaaaaaaacatgcttttgggaggagagatacta

baochun1 acaaaatattttctttgtgcgtcggtaaaaaaaaacatgcttttgggaggagagatacta

************************************************************

baochun2 tttcaccaatcgagtcacaggtatctaacctactcttacctaatgattttccacaaagtg

baochun1 tttcaccaatcgagtcacaggtatctaacctactcctacctaatgattttccacaaagtg

***********************************.************************

baochun2 gtgacgaaatgtataacttggacagatctttccattttccaagtcgatccgatcgtagag

baochun1 gtgacgaaatgtataacttggacagatctttccattttccaagtcgatccgatcgtagag

************************************************************

baochun2 ctatttactcgatcgcagatatttatggaacacctctaacagagggacaaatcgtcaatt

baochun1 ctatttactcgatcgcagatatttctggaacacctctaacagagggacaaatcgtcaatt

************************ ***********************************

baochun2 ttgaaagaacttactgtcaacctctttccgatctgaatctatctgattcagaagggaaga

baochun1 ttgaaagaacttactgtcaacctctttccgatctgaatctatctgattcagaagggaaga

************************************************************

baochun2 acttgcatcagtatctcaatttcaattcaaacatggatttgattcacattccatgttctg

baochun1 acttgcatcagtatctcaatttcaattcaaacatggatttgattcacattccatgttctg

************************************************************

baochun2 agaagaaatatttaccatccgaaaagaggaaaaaacggagtctttgtctaaagaaattcg

baochun1 agaagaaatatttaccatccgaaaagaggaaaaaacggagtctttgtctaaagaaattcg

************************************************************

baochun2 ttgagaaagtgcagatggataaaacctttcaacgagatagtgccttttcaactctctcaa

baochun1 ttgagaaagtgcagatggataaaacctttcaacgagatagtgccttttcaactctctcaa

************************************************************

baochun2 aatggaatctattccaaacatatatgccatggttccttacttcgacaggatacaaatatc

baochun1 aatggaatctattccaaacatatatgccatggttccttacttcgacaggatacaaatatc

************************************************************

baochun2 taaatttgatatttttagatactttttcagacctattgccgatactaagtagcagtcaaa

baochun1 taaatttgatatttttagatactttttcagacctattgccgatactaagtagcagtcaaa

************************************************************

baochun2 aatttgtatccatttttcatgatattatgcatggatcagatatatcatggcgaattcttc

baochun1 aatttgtatccatttttcatgatattatgcatggatcagatatatcatggcgaattcttc

************************************************************

baochun2 agaaaaaattgtgtcttccacaatggaatctgataagtgagatttcgagtaagtgtttac

baochun1 agaaaaaattgtgtcttccacaatggaatctgataagtgagatttcgagtaagtgtttac

************************************************************

baochun2 ataatatccttctgtccgaagaaatgattcatcaaaaaaatgagtcaccatcgacacatc

baochun1 ataatatccttctgtccgaagaaatgattcatcaaaaaaatgagtcaccatcgacacatc

************************************************************

baochun2 tgagatcgccaaatgttcgggagttcctctattcaatccttttccttcttcttgttgctg

baochun1 tgagatcgccaaatgttcgggagttcctctattcaatccttttccttcttcttgttgctg

************************************************************

baochun2 gatatctcgttcgtacacatcttctctttgtttcccgggcctctagtgagttacagacag

baochun1 gatatctcgttcgtacacatcttctctttgtttcccgggcctctagtgagttacagacag

************************************************************

baochun2 agttcgaaaaggtaaaatctttgatgattccatcatctatgattgagttgcgaaaacttc

baochun1 agttcgaaaaggtaaaatctttgatgattccatcatctatgattgagttgcgaaaacttc

************************************************************

baochun2 tggataggtatcctacatctgaaccgaattctttctggttaaagaatctctttctagttt

baochun1 tggataggtatcctacatctgaaccgaattctttctggttaaagaatctctttctagttt

************************************************************

baochun2 ctctggaacaattaggaaattctctagaagaaatacggggttctgcttatggcggcaaca

baochun1 ctctggaacaattaggaaattctctagaagaaatacggggttctgcttatggcggcaaca

************************************************************

baochun2 tgctattggggggtggtcccctttatggggtcaactcaatacgttctaagaagaaatatt

baochun1 tgctattggggggtggtcccctttatggggtcaactcaatacgttctaagaagaaatatt

************************************************************

baochun2 tgaatataaatctcatcgatctcataagtatcataccaaatcccatcaatcgaatcactt

baochun1 tgaatataaatctcatcgatctcataagtatcataccaaatcccatcaatcgaatcactt

************************************************************

baochun2 tttcgagaaatacgagacatctaagtcatacaagtaaagagatctattcattgataagaa

baochun1 tttcgagaaatacgagacatctaagtcatacaagtaaagagatctattcattgataagaa

************************************************************

baochun2 aaagaaaaaacgtgaacggggattggattgatgataaaatagaatcctgggtcgcgaaca

baochun1 aaagaaaaaacgtgaacggggattggattgatgataaaatagaatcctgggtcgcgaaca

************************************************************

baochun2 gtgattcgattgatgatgaagaaagagaattcttggttcagttctccaccttaacgacag

baochun1 gtgattcgattgatgatgaagaaagagaattcttggttcagttctccaccttaacgacag

************************************************************

baochun2 aaaaaaggattgatcaaattctattgagtctgactcatagtgatcatttatcaaagaatg

baochun1 aaaaaaggattgatcaaattctattgagtctgactcatagtgatcatttatcaaagaatg

************************************************************

baochun2 gctctggttttgaaatgattgaacaaccgggagcaatttacttacgatacttagttgaca

baochun1 gctctggttttgaaatgattgaacaaccgggagcaatttacttacgatacttagttgaca

************************************************************

baochun2 ttcagaaaaagtatctaatgaattatgagttcaatacatcctgtttagcagaaagacgga

baochun1 ttcagaaaaagtatctaatgaattatgagttcaatacatcctgtttagcagaaagacgga

************************************************************

baochun2 tatctcttgctcattatcagacaatcacttattcacaaacctcgtgtggggctaatagtt

baochun1 tatctcttgctcattatcagacaatcacttattcacaaacctcgtgtggggctaatagtt

************************************************************

baochun2 ttcattttccatctcatgaaaaacccttttcgctccgcttagccttatccccctctaggg

baochun1 ttcattttccatctcatgaaaaacccttttcgctccgcttagccttatccccctctaggg

************************************************************

baochun2 gtattttagtgataggttctataggaactggacgatcctatttggtcaaatacctagcga

baochun1 gtattttagtgataggttctataggaactggacgatcctatttggtcaaatacctagcga

************************************************************

baochun2 caaactcctatgttcctttgattacggtatttctgaacaagttcctggataacaagacta

baochun1 caaactcctatgttcctttgattacggtatttctgaacaagttcctggataacaagacta

************************************************************

baochun2 aaggtttttttattgatgatatcgacattgatgatagtgacgatattaatgctagtgacg

baochun1 aaggtttttttattgatgatatcgacattgatgatagtgacgatattaatgctagtgacg

************************************************************

baochun2 atattaatgctaatgacgatatcgatcgtgaccttgatacagagctggagcggctaactc

baochun1 atattaatgctaatgacgatatcgatcgtgaccttgatacagagctggagcggctaactc

************************************************************

baochun2 tgatgaatgctactatggatatgatgtcggaaatagaccgattttatatcacccttcaat

baochun1 tgatgaatgctactatggatatgatgtcggaaatagaccgattttatatcacccttcaat

************************************************************

baochun2 tcgaattagcaaaagcaatgtctccttgcataatatggattccaaacattcatgatctgg

baochun1 tcgaattagcaaaagcaatgtctccttgcataatatggattccaaacattcatgatctgg

************************************************************

baochun2 atgtgaatgagtcgaattacttatccctcggtttattagtgaatcgtctctccagggatt

baochun1 atgtgaatgagtcgaattacttatccctcggtttattagtgaatcgtctctccagggatt

************************************************************

baochun2 gtgaaagatgttccactcgaaatattcttgttattgcttcgactcatattccccaaaaag

baochun1 gtgaaagatgttccactcgaaatattcttgttattgcttcgactcatattccccaaaaag

************************************************************

baochun2 tggatcccgctttaatagctccgaataaattaaatacgtgcattaagatacgaaggcttc

baochun1 tggatcccgctttaatagctccgaataaattaaatacgtgcattaagatacgaaggcttc

************************************************************

baochun2 ttattccacaacaacgaaagcactttttcactctttcatatactaggggatttcacttgg

baochun1 ttattccacaacaacgaaagcactttttcactctttcatatactaggggatttcacttgg

************************************************************

baochun2 aaaagaaaaagtgccatactaatggattcgggtccataaccatgggttccaatgcacgag

baochun1 aaaagaaaaagtgccatactaatggattcgggtccataaccatgggttccaatgcacgag

************************************************************

baochun2 atcttgtagcacttaccaatgaggccctatcgattagtattacacagaagaaatcaattc

baochun1 atcttgtagcacttaccaatgaggccctatcgattagtattacacagaagaaatcaattc

************************************************************

baochun2 tagacactaatacaattagatccgctcttcataaacaaacttgggatttgcgatcccagg

baochun1 tagacactaatacaattagatccgctcttcataaacaaacttgggatttgcgatcccagg

************************************************************

baochun2 taagatcggttcaggatcacgggctccttttctatcagataggaagggctgttgcgcaaa

baochun1 taagatcggttcaggatcacgggctccttttctatcagataggaagggctgttgcgcaaa

************************************************************

baochun2 atgtacttctaagtaattgccccatagatcctatatctatctatatgaagaagaaatcat

baochun1 atgtacttctaagtaattgccccatagatcctatatctatctatatgaagaagaaatcat

************************************************************

baochun2 gtaacgagggggattcttatttgtacaaatggtacttcgaacttggaacgagcatgaaga

baochun1 gtaacgaaggggattcttatttgtacaaatggtacttcgaacttggaacgagcatgaaga

*******.****************************************************

baochun2 aattaacgatacttctttatcttttgagttgttctgccggatcggtcgctcaagatcttt

baochun1 aattaacgatacttctttatcttttgagttgttctgccggatcggtcgctcaagatcttt

************************************************************

baochun2 ggtctctacccggacccgatgaaaaaaatgggatcacttcttatggactcgttgagaatg

baochun1 ggtctctacccggacccgatgaaaaaaatgggatcacttcttatggactcgttgagaatg

************************************************************

baochun2 attctgatctagttcatggcctattagaagtagaaagcgctctggtgggatcctcacgga

baochun1 attctgatctagttcatggcctattagaagtagaaagcgctctggtgggatcctcacgga

************************************************************

baochun2 cagaaaaagattgcagtcagtttgataatgatcgagtgatattgcttcgtcggcccgaac

baochun1 cagaaaaagattgcagtcagtttgataatgatcgagtgatattgcttcgtcggcccgaac

************************************************************

baochun2 caaggaatcccttagatatgatgcaaaacggatcttgttctatccttgatcagagatttc

baochun1 caaggaatcccttagatatgatgcaaaacggatcttgttctatccttgatcagagatttc

************************************************************

baochun2 tctatgaaaaatacgaatcggagtttgaagaaggggaaggagcccttgacccgcaacaga

baochun1 tctatgaaaaatacgaatcggagtttgaagaaggggaaggagcccttgacccgcaacaga

************************************************************

baochun2 tagaggaggatttattcaatcacatagtttgggctcctagaatatggcgcccttggggct

baochun1 tagaggaggatttattcaatcacatagtttgggctcctagaatatggcgcccttggggct

************************************************************

baochun2 ttctatttgattggatcgaaaggcccaatgaattgggatttccctattggtccaggtcat

baochun1 ttctatttgattggatcgaaaggcccaatgaattgggatttccctattggtccaggtcat

************************************************************

baochun2 ttcggggcaagcggatgatttatgatgaagaggatgagcttcaagagaatgattcggagt

baochun1 ttcggggcaagcggatgatttatgatgaagaggatgagcttcaagagaatgattcggagt

************************************************************

baochun2 tcttgcagagtggaaccatgcagtaccagacacgagatagatcttccagggaacaaggcc

baochun1 tcttgcagagtggaaccatgcagtaccagacacgagatagatcttccagggaacaaggcc

************************************************************

baochun2 tttttcgaataagccaattcatttgggaccctgcagatccactctttttcctattcaaag

baochun1 tttttcgaataagccaattcatttgggaccctgcagatccactctttttcctattcaaag

************************************************************

baochun2 atcagccctttgtctctgtgttttcacatcgagaattatttgcagatgaagagatgtcaa

baochun1 atcagccctttgtctctgtgttttcacatcgagaattatttgcagatgaagagatgtcaa

************************************************************

baochun2 aggggcttcttacttcccaaacagatcctcctacatctatatataaaggctggtttatca

baochun1 aggggcttcttacttcccaaacagatcctcctacatctatatataaaggctggtttatca

************************************************************

baochun2 agaatacgcaagaaaagcactttgaattgttgattaatcgccagagatggcttagaaaca

baochun1 agaatacgcaagaaaagcactttgaattgttgattaatcgccagagatggcttagaaaca

************************************************************

baochun2 atagttcattatctaatggatctttccgttctaatactctatccgagagttatcagtatt

baochun1 atagttcattatctaatggatctttccgttctaatactctatccgagagttatcagtatt

************************************************************

baochun2 tatcaaatctcttcctatctaacggaaggttattggatcaaatgacaacgacattgttga

baochun1 tatcaaatctcttcctatctaacggaaggttattggatcaaatgacaacgacattgttga

************************************************************

baochun2 gaaaaagatggcttttcccggatgaaatgcaaattggattcatgtaacaggagaaagatt

baochun1 gaaaaagatggcttttcccggatgaaatgcaaattggattcatgtaacaggagaaagatt

************************************************************

baochun2 tcccattccttagccggaaagatatgtagccatgaaagagggattaagtggaacagaatt

baochun1 tcccattccttagccggaaagatatgtagccatgaaagagggattaagtggaacagaatt

************************************************************

baochun2 gactagtcatggaaacacctttttcttctttattcttccatattttggaccttatctcca

baochun1 gactagtcatggaaacacctttttcttcttt-ttcttccatattttggaccttatctcca

******************************* ****************************

baochun2 tggaacaacatactactgctgaaacatggaagaattgaaatattcgatcaaaacactatg

baochun1 tggaacaacatactactgctgaaacatggaagaattgaaatattcgatcaaaacactatg

************************************************************

baochun2 tatggatggtatgaactgcctaaacaagaattcttgaacagcgaacaaccagttcagata

baochun1 tatggatggtatgaactgcctaaacaagaattcttgaacagcgaacaaccagttcagata

************************************************************

baochun2 ttcacgaccaagaagtattggattctttttcggataggccccgaaaggagaaggaaggct

baochun1 ttcacgaccaagaagtattggattctttttcggataggccccgaaaggagaaggaaggct

************************************************************

baochun2 agaatgccagcaggcgtctattattgaattcaccccacccgatagtacccattttgggaa

baochun1 agaatgccagcaggcgtctattattgaattcgccccacccgatagtacccattttgggaa

*******************************.****************************

baochun2 cgtccagtgccaaagtcactgaatgggtaagtcgccaatccctaaaacggactatgtaat

baochun1 cgtccagtgccaaagtcactgaatgggtaagtcgccaatccctaaaacggactatgtaat

************************************************************

baochun2 gtactttatctgcggggttaggaggaggcatttcattttcccagaggctacctttgtgtg

baochun1 gtactttatctgcggggttaggaggaggcatttcattttcccagaggctacctttgtgtg

************************************************************

baochun2 attcctgttgaagcgtatactcggggggtgggtgcagggcggaccttttttttaaagcag

baochun1 attcctgttgaagcgtatactcggggggtgggtgcagggcggaccttttttttaaagcag

************************************************************

baochun2 actccccattcattagatagagaagatccccaagatttcgtaatccgctgccgaacttat

baochun1 actccccattcattagatagagaagatccccaagatttcgtaatccgctgccgaacttat

************************************************************

baochun2 tccaattccaagagctcggatcaatcggtattgatagaccgattgatctgagctctctta

baochun1 tccaattccaagagctcggatcaatcggtctatcaataccgattgatccgagctctctta

***************************** * * ***********.***********

baochun2 ttgaattgctcattcaatgagcattctcaatattatgccttgaagaggactcgaacctcc

baochun1 ttgaattgctcattcaatgagcattctcaatattatgccttgaagaggactcgaacctcc

************************************************************

baochun2 acgctctttagcacgagattttgagtctcgcgtgtctaccatttcaccaccaaggcatct

baochun1 acgctctttagcacgagattttgagtctcgcgtgtctaccatttcaccaccaaggcatct

************************************************************

baochun2 tgaaagtgaatcgtattccatcaatatgataacttatctatctagtgtgatgtatggaat

baochun1 tgaaagtgaatcgtattccatcaatatgataacttatctatctagtgtgatgtatggaat

************************************************************

baochun2 atatgacaaaggtggggtgttggagtattgctattgatcggtcatgtcatataggcccga

baochun1 atatgacaaaggtggggtgttggagtattgctattgatcggtcatgtcatataggcccga

************************************************************

baochun2 gttggacatccaattgcttcaatttgaattatccggaggatgccttatatatcaaaaaga

baochun1 gttggacatccaattgcttcaatttgaattatccggaggatgccttatatatcaaaaaga

************************************************************

baochun2 tggacaatcaaacctatttctcgattcactagaagcccaaagaggtgaatagggtccaaa

baochun1 tggacaatcaaacctatttctcgattcactagaagcccaaagaggtgaatagggtccaaa

************************************************************

baochun2 ataaggagagatatgtaaaaagcaagtccgactacgtctcttccgaatcctaaatggaat

baochun1 ataaggagagatatgtaaaaagcaagtccgactacgtctcttccgaatcctaaatggaat

************************************************************

baochun2 gtagggattcatatgtaaacatagtatctatttagatacgctcgaataaccccttctcat

baochun1 gtagggattcatatgtaaacatagtatctatttagatacgctcgaataaccccttctcat

************************************************************

baochun2 aatgagaatgtatatccctatttcggtctggtccggtatggaatgaacttataatcatgg

baochun1 aatgagaatgtatatccctatttcggtctggtccggtatggaatgaacttataatcatgg

************************************************************

baochun2 aatcgactcgatcatcagattatagattataagttcataaccccagcccattcccgtttt

baochun1 aatcgactcgatcatcagattatagattataagttcataaccccagcccattcccgtttt

************************************************************

baochun2 ggacggaacagatctactaattctttgattccaggtagtgagagggatcttgaactaaga

baochun1 ggacggaacagatctactaattctttgattccaggtagtgagagggatcttgaactaaga

************************************************************

baochun2 aatagaccctagaagctaaaaaagggtatcctgcgcaattgcaataatcgggttcattga

baochun1 aatagaccctagaagctaaaaaagggtatcctgcgcaattgcaataatcgggttcattga

************************************************************

baochun2 tattcctggtatagtagatgctattacacatacaatcatactaaattcgatggaattgtt

baochun1 tattcctggtatagtagatgctattacacatacaatcatactaaattcgatggaattgtt

************************************************************

baochun2 tgatcttaaaggagatcttctataatttcgcacgtaaggggttatttcttgggttcgtcc

baochun1 tgatcttaaaggagatcttctataatttcgcacgtaaggggttatttcttgggttcgtcc

************************************************************

baochun2 agtcattaataacttgattatttttagataatagtagatagaaacaacgcttgtaaggag

baochun1 agtcattaataacttgattatttttagataatagtagatagaaacaacgcttgtaaggag

************************************************************

baochun2 tcctattaaaaccaagaaatataggcctgcctgccatccacaccagaataaatagagttt

baochun1 tcctattaaaaccaagaaatataggcctgcctgccatccacaccagaataaatagagttt

************************************************************

baochun2 tccgaaaaaacctgctagtgggggaagaccccctagggataagagacatagggctaaaga

baochun1 tccgaaaaaacctgctagtgggggaagaccccctagggataagagacatagggctaaaga

************************************************************

baochun2 gagagccaaaaaaggatctttcgtgtataatcctgcataatctcgaatgttatcagttcc

baochun1 gagagccaaaaaaggatctttcgtgtataatcctgcataatctcgaatgttatcagttcc

************************************************************

baochun2 agtacgtagaccaaataatacaatgcaagcaaaagttcctagattcatggagatatagaa

baochun1 agtacgtagaccaaataatacaatgcaagcaaaagttcctagattcatggagatatagaa

************************************************************

baochun2 cagcatataagttatcatgcttgcatatccatcatttgagtctccaacaattattccaat

baochun1 cagcatataagttatcatgcttgcatatccatcatttgagtctccaacaattattccaat

************************************************************

baochun2 aattacatatccgatttgacctatggacgaatatgcaagcatacgtttcatgcttgtttg

baochun1 aattacatatccgatttgacctatggacgaatatgcaagcatacgtttcatgcttgtttg

************************************************************

baochun2 agtaatagcaatgagatttcccaatatcatgctaagaatagctaggatttccagaagaag

baochun1 agtaatagcaatgagatttcccaatatcatgctaagaatagctaggatttccagaagaag

************************************************************

baochun2 atgccattcgtttgatgagaaataaaaaggaatatcgaaaattcgagtggctaaagctga

baochun1 atgccattcgtttgatgagaaataaaaaggaatatcgaaaattcgagtggctaaagctga

************************************************************

baochun2 agcagctactttcgaagtaacagaaagaaaagcaacgactggagtgggagagtcagagtc

baochun1 agcagctactttcgaagtaacagaaagaaaagcaacgactggagtgggagagtcagagtc

************************************************************

baochun2 gaaaagaggattcctcacttctttctctcattcaaaaccgtgcatgagactttcatctca

baochun1 gaaaagaggattcctcacttctttctctcattcaaaaccgtgcatgagactttcatctca

************************************************************

baochun2 cacggctcctaagtgataaaagaaagaagaactcatcttctttcttttttgattaccttc

baochun1 cacggctcctaagtgataaaagaaagaagaactcatcttctttcttttttgattaccttc

************************************************************

baochun2 ctcgcgtatgtataagaccgaatccattcgatttcgaaaaaggattactaatccttaact

baochun1 ctcgcgtatgtataagaccgaatccattcgatttcgaaaaaggattactaatccttaact

************************************************************

baochun2 tttcgaggaatccttcatcagtggttgtgaatgactgattttttcaatcttttcaacctt

baochun1 tttcgaggaatccttcatcagtggttgtgaatgactgattttttcaatcttttcaacctt

************************************************************

baochun2 gtttccgtaggagcaagtcagaaagattgagaaatagaaccatctgatttgattcgttct

baochun1 gtttccgtaggagcaagtcagaaagattgagaaatagaaccatctgatttgattcgttct

************************************************************

baochun2 caatagccatgaaatgatcatattagggtgatccttttgtcgacggatgctcctattata

baochun1 caatagccatgaaatgatcatattagggtgatccttttgtcgacggatgctcctattata

************************************************************

baochun2 ctcgtagtctctgaaggatgagaaccaactatgtagcatctacatcgagaattcaagtat

baochun1 ctcgtagtctctgaaggatgagaaccaactatgtagcatctacatcgagaattcaagtat

************************************************************

baochun2 tgtatacgtcattagtccgatcctttgtaggaactacccgtaataacgaacttgcaaaat

baochun1 tgtatacgtcattagtccgatcctttgtaggaactacccgtaataacgaacttgcaaaat

************************************************************

baochun2 gtatctgtttatcataaagagatttgtcgttcctgaccctgcttcaccttaattattatt

baochun1 gtatctgtttatcataaagagatttgtcgttcctgaccctgcttcaccttaattattatt

************************************************************

baochun2 tgaacaagtaaaagttctgtcgtggtccgagtggggatagcatttctcttctgcatgtcc

baochun1 tgaacaagtaaaagttctgtcgtggtccgagtggggatagcatttctcttctgcatgtcc

************************************************************

baochun2 atagagttttgaaaaatccaaacatctcagagatagatagagaggtaggaatttatcgaa

baochun1 atagagttttgaaaaatccaaacatctcagagatagatagagaggtaggaatttatcgaa

************************************************************

baochun2 cgaaccgcactccttcgtatacgtcaggagtccattgatgagaaggggctagggaaagct

baochun1 cgaaccgcactccttcgtatacgtcaggagtccattgatgagaaggggctagggaaagct

************************************************************

baochun2 tgaacccaattcctacggtgatgaatatgagtgaaattgaaattcccggggagttataca

baochun1 tgaacccaattcctacggtgatgaatatgagtgaaattgaaattcccggggagttataca

************************************************************

baochun2 tttgtgtattgataagaccattcactatttcttgaagctcgacctctcccccggataaac

baochun1 tttgtgtattgataagaccattcactatttcttgaagctcgacctctcccccggataaac

************************************************************

baochun2 catatagccaagagaaaccatgaaccagaatagaagagcttgccccacccatgagtaaat

baochun1 catatagccaagagaaaccatgaaccagaatagaagagcttgccccacccatgagtaaat

************************************************************

baochun2 atttcatagtagcctcattagaccgtacatctttcttggtatatccagataataggtagg

baochun1 atttcatagtagcctcattagaccgtacatctttcttggtatatccagataataggtagg

************************************************************

baochun2 agcataaactgaaacattctggagctacaaagatagttattaaatcgttagcaccgcata

baochun1 agcataaactgaaacattctggagctacaaagatagttattaaatcgttagcaccgcata

************************************************************

baochun2 aaaacattcctcctagagtagctgttaagacgaataagagaaactctgttatagccattt

baochun1 aaaacattcctcctagagtagctgttaagacgaataagagaaactctgttatagccattt

************************************************************

baochun2 ctgtacattcaacgtactctacggatagaggaatacatagagttgaacatagtaaaataa

baochun1 ctgtacattcaacgtactctacggatagaggaatacatagagttgaacatagtaaaataa

************************************************************

baochun2 gaaattgaaagatttcgttgaaattgttcgtttggaaatttcccgaaaagctaatcatag

baochun1 gaaattgaaagatttcgttgaaattgttcgtttggaaatttcccgaaaagctaatcatag

************************************************************

baochun2 gttcttctctccatcggaacaatagggccgttatgctcattactaaacttgttgaagaga

baochun1 gttcttctctccatcggaacaatagggccgttatgctcattactaaacttgttgaagaga

************************************************************

baochun2 tgaaatataaccaaggtatatctttttgatcagaggttgaatcgaccatcagaagaagaa

baochun1 tgaaatataaccaaggtatatctttttgatcagaggttgaatcgaccatcagaagaagaa

************************************************************

baochun2 ttaggccaaaaattaggatacactctgggaaaataaaacttccatcgaagagaagcaaat

baochun1 ttaggccaaaaattaggatacactctgggaaaataaaacttccatcgaagagaagcaaat

************************************************************

baochun2 gaaaggctttcataaaaattctcgtagaatcgagaatgaagttttcattctgtacatgcc

baochun1 gaaaggctttcataaaaattctcgtagaatcgagaatgaagttttcattctgtacatgcc

************************************************************

baochun2 agatcatgaattagtaactgcatccaatttccaaaaaaatcccaattgtttcgaactttc

baochun1 agatcatgaattagtaactgcatccaatttccaaaaaaatcccaattgtttcgaactttc

************************************************************

baochun2 tatttttggaatggaatctccatgaataggatcaaaccttatttcatggtatttacatga

baochun1 tatttttggaatggaatctccatgaataggatcaaaccttatttcatggtatttacatga

************************************************************

baochun2 ggttcctctttcttattcttaagcaaatcctcgagagggcttagttgatccatgatttat

baochun1 ggttcctctttcttattcttaagcaaatcctcgagagggcttagttgatccatgatttat

************************************************************

baochun2 ctttcatctttggtttccttttcgtttgtttcgagaaatagatcgatcaatttcgattct

baochun1 ctttcatctttggtttccttttcgtttgtttcgagaaatagatcgatcaatttcgattct

************************************************************

baochun2 ttctttattctcttgattcttttccgatcgagatgtatggatctatgtgtctatatatag

baochun1 ttctttattctcttgattcttttccgatcgagatgtatggatctatgtgtctatatatag

************************************************************

baochun2 atcctgtttatggattaacgaaaatgtgcaaaagctctatttgcctctgccattctatga

baochun1 atcctgtttatggattaacgaaaatgtgcaaaagctctatttgcctctgccattctatga

************************************************************

baochun2 gtctcttcctttttgcgtatggcctcgccactccctttggcagcatccactaattcggaa

baochun1 gtctcttcctttttgcgtatggcctcgccactccctttggcagcatccactaattcggaa

************************************************************

baochun2 cttaatttgaaagccatatttcgacccggacgttttcgggatgccgataataaccaacga

baochun1 cttaatttgaaagccatatttcgacccggacgttttcgggatgccgataataaccaacga

************************************************************

baochun2 atggcaagtgcttttccttgtgtggatcctatttcaatgggaacttgatgagtcgatcca

baochun1 atggcaagtgcttttccttgtgtggatcctatttcaatgggaacttgatgagtcgatcca

************************************************************

baochun2 cctacacgtcttgcttttactgctatatcgggagttactccacgtattgcttgacgtaaa

baochun1 cctacacgtcttgcttttactgctatatcgggagttactccacgtattgcttgacgtaaa

************************************************************

baochun2 acagatagtggatttgtttctgtcttttgttgaatctttttcatggctcgatagataatt

baochun1 acagatagtggatttgtttctgtcttttgttgaatctttttcatggctcgatagataatt

************************************************************

baochun2 tgataagccaatgatttttttccgtgtttcagaatacggttaaccaacatattaactaat

baochun1 tgataagccaatgatttttttccgtgtttcagaatacggttaaccaacatattaactaat

************************************************************

baochun2 cgattacgataaattggatccgattttacagttttttcttttgcagtacctcgacgtgac

baochun1 cgattacgataaattggatccgattttacagttttttcttttgcagtacctcgacgtgac

************************************************************

baochun2 atgagtgtgaaagaggttcaagaatcagttttatttttataagggctaaaatcacttatt

baochun1 atgagtgtgaaagaggttcaagaatcagttttatttttataagggctaaaatcacttatt

************************************************************

baochun2 ttggcttttttaccccatattgtagggtggatctcgaaagatatgaaagatctccctcca

baochun1 ttggcttttttaccccatattgtagggtggatctcgaaagatatgaaagatctccctcca

************************************************************

baochun2 agccgtacatacgactttcatcgaatacggctttccacaaaattctatatgtctctatga

baochun1 agccgtacatacgactttcatcgaatacggctttccacaaaattctatatgtctctatga

************************************************************

baochun2 gatcgagtatggaattctgtttactcactttattaaattgagtatccgtttccctccttt

baochun1 gatcgagtatggaattctgtttactcactttattaaattgagtatccgtttccctccttt

************************************************************

baochun2 tcttgctaggatgggaaatcctgtattttacatatccatacgattgagtccttgggtttc

baochun1 tcttgctaggatgggaaatcctgtattttacatatccatacgattgagtccttgggtttc

************************************************************

baochun2 cgaaatagtgtaatatagtgtaataaagaagtgcttcgaatcattgctatttgactcgga

baochun1 cgaaatagtgtaatatagtgtaataaagaagtgcttcgaatcattgctatttgactcgga

************************************************************

baochun2 cttgttctaaaaaagtcgaggtatttcgaattctttgttgacacggacaaagtcagggaa

baochun1 cctgttctaaaaaagtcgaggtatttcgaattctttgttgacacggacaaagtcagggaa

*.**********************************************************

baochun2 aacctctgaaattatttcaatattggaccttggacatataatagttccgaattgaatctc

baochun1 aacctctgaaattatttcaatattggaccttggacatataatagttccgaattgaatctc

************************************************************

baochun2 tttagaaagaagatcttttgtctcatggtagcctgctccagtccccttacgaaactttcg

baochun1 tttagaaagaagatcttttgtctcatggtagcctgctccagtccccttacgaaactttcg

************************************************************

baochun2 ttattgggttagctatacacttcacatgtttctagcgattcacatggcatcatcaaatga

baochun1 ttattgggttagctatacacttcacatgtttctagcgattcacatggcatcatcaaatga

************************************************************

baochun2 tacaagtcttggataagaatctacaacgcactagaacgcccttgttgacgatcttttact

baochun1 tacaagtcttggataagaatctacaacgcactagaacgcccttgttgacgatcttttact

************************************************************

baochun2 ccgacagcatctagggttcctcgaacaatgtgatatctcacaccgggtaaatccttaacc

baochun1 ccgacagcatctagggttcctcgaacaatgtgatatctcacaccgggtaaatccttaacc

************************************************************

baochun2 cttccccctcttactaagactacagaatgttcttgtgaattatggccaataccaggtata

baochun1 cttccccctcttactaagactacagaatgttcttgtgaattatggccaataccaggtata

************************************************************

baochun2 taagcagtgatttcaaatccagaggttaatcgtactctggcaactttacgtaaggcagag

baochun1 taagcagtgatttcaaatccagaggttaatcgtactctggcaactttacgtaaggcagag

************************************************************

baochun2 tttggttttttgggggtgatagtggaaaagttgacagataagtcacccttactgccgctc

baochun1 tttggttttttgggggtgatagtggaaaagttgacagataagtcacccttactgccactc

********************************************************.***

baochun2 tacagaaccgtacatgagattttcaccgcatacggctcctcgttcaattctttcgaagtc

baochun1 tacagaaccgtacatgagattttcaccgcatacggctcctcgttcaattctttcgaagtc

************************************************************

baochun2 attggatccttttgctcgttcgagaatctcttcctttcttccactccatcccgaagagta

baochun1 attggatccttttgctcgttcgagaatctcttcctttcttccactccatcccgaagagta

************************************************************

baochun2 actaggaccaatttagtgacgttttcatatttcaattgaacactttccattttttttttt

baochun1 actaggaccaatttagtgacgttttcatgtttcaattgaacactttcca-tttttttttt

****************************.******************** **********

baochun2 cctctatcccatagagacagtgtttgaatcaatagagaaccttttcttctgtatgaatcg

baochun1 cctctatcccatagagacagtgtttgaatcaatagagaaccttttcttctgtatgaatcg

************************************************************

baochun2 atattattacattccaattccttcccgatacctcccaaggaaaatctcgaattggatccc

baochun1 atattattacattccaattccttcccgatacctcccaaggaaaatctcgaattggatccc

************************************************************

baochun2 aaattgacgggttagtgtgagcttatccatgcggttatgcactcttcgaataggaatcca

baochun1 aaattgacgggttagtgtgagcttatccatgcggttatgcactcttcgaataggaatcca

************************************************************

baochun2 ttttctgaaagattgtggctttcatgctttggtgggtctccgagatcctttcgatgacct

baochun1 ttttctgaaagattgtggctttcgtgctttggtgggtctccgagatcctttcgatgacct

***********************.************************************

baochun2 atgtttattgaagggatatctatataatccgattgattgcgtaaagcccgcgggcggtag

baochun1 atgtttattgaagggatatctatataatccgattgattgcgtaaagcccgcgggcggtag

************************************************************

baochun2 caacggaaccggggaaagtatacagaaaagacagttctattctattactagattagttag

baochun1 caacggaaccggggaaagtatacagaaaagacagttctattctattactagattagttag

************************************************************

baochun2 tgatcccgtcttagggagccctttcttccatgaagaaccgttggcatcagtcctaatttt

baochun1 tgatcccgtcttagggagccctttcttccatgaagaaccgttggcatcagtcctaatttt

************************************************************

baochun2 gtctctgtggaccgaggagaaagagggctcggcgggaagaggattgtaccatgagagaag

baochun1 gtctctgtggaccgaggagaaagagggctcggcgggaagaggattgtaccatgagagaag

************************************************************

baochun2 caaggaggtcaacctctttcaaatatacaacatggattctggcaatgcaatgtagttgga

baochun1 caaggaggtcaacctctttcaaatatacaacatggattctggcaatgcaatgtagttgga

************************************************************

baochun2 ctctcatgtcgatctgaatcaattatcctttccacggaggtaaatctttgcctgttcggc

baochun1 ctctcatgtcgatctgaatcaattatcctttccacggaggtaaatctttgcctgttcggc

************************************************************

baochun2 aagaggatagcaagttacaaattctgtctcggtaggacatgtatttctattactatgaaa

baochun1 aagaggatagcaagttacaaattctgtctcggtaggacatgtatttctattactatgaaa

************************************************************

baochun2 ttcataaatgaagtagttaatagtggggttaccattaccctttttgtagtgacgcatctt

baochun1 ttcataaatgaagtagttaatagtggggttaccattaccctttttgtagtgacgcatctt

************************************************************

baochun2 gtatgtgttgctaataaaaggaatttgtccatttttcggggtctcaaaggggcgtgaaaa

baochun1 gtatgtgttgctaataaaaggaatttgtccatttttcggggtctcaaaggggcgtgaaaa

************************************************************

baochun2 cacataagaactcttgaatggaaaagagatagaactccagttccttcagaaatggtaaga

baochun1 cacataagaactcttgaatggaaaagagatagaactccagttccttcagaaatggtaaga

************************************************************

baochun2 tctttggcgcaagaagagggggttgatccgtatcatcttgacttggttctgcttcctcta

baochun1 tctttggcgcaagaagagggggttgatccgtatcatcttgacttggttctgcttcctcta

************************************************************

baochun2 tttttttacgaatactgagtcgggttcttctcctacctgtatcaaatagaacatgctgag

baochun1 tttttttacgaatactgagtcgggttcttctcctacctgtatcaaatagaacatgctgag

************************************************************

baochun2 ctaaatcttcttcatggaaaacctgcttgatttagatcgggaaaatcgtgcggttttatg

baochun1 ctaaatcttcttcatggaaaacctgcttgatttagatcgggaaaatcgtgcggttttatg

************************************************************

baochun2 aaaccatgtgctatggctcgaatccgtagtcaaccctctttccgataggggcagttgaca

baochun1 aaaccatgtgctatggctcgaatccgtagtcaaccctctttccgataggggcagttgaca

************************************************************

baochun2 attgaatccgattttctcattattttcatatatgtaaaagtgcgaaaagaaggacccgct

baochun1 attgaatccgattttctcattattttcatatatgtaaaagtgcgaaaagaaggacccgct

************************************************************

baochun2 ccaagttgttcaagaatagtggcgttgagtttctagaccctttgccttaggattagtcag

baochun1 ccaagttgttcaagaatagtggcgttgagtttctagaccctttgccttaggattagtcag

************************************************************

baochun2 ttctatttctcgatggaggcagagaagggatataactcagtggtagagtgtcaccttgac

baochun1 ttctatttctcgatggaggcagagaagggatataactcagtggtagagtgtcaccttgac

************************************************************

baochun2 gtggtggaagtcatcagttcgagcctgtttatccctaaacccaatgtgagtttttcgatt

baochun1 gtggtggaagtcatcagttcgagcctgtttatccctaaacccaatgtgagtttttcgatt

************************************************************

baochun2 ttgacttgctcccccgccatgattgaatgcgaatggataagaggctcgtgggattgacgt

baochun1 ttgacttgctcccccgccatgattgaatgcgaatggataagaggctcgtgggattgacgt

************************************************************

baochun2 gagggggcagggatggctatatttctgggagcgaactccgggcgaatatgccttggaatg

baochun1 gagggggcagggatggctatatttctgggagcgaactccgggcgaatatgccttggaatg

************************************************************

baochun2 aaagaaaattacgaatccgctttgtctacgaacaaggaagctataagtaatgcaactatg

baochun1 aaagaaaattacgaatccgctttgtctacgaacaaggaagctataagtaatgcaactatg

************************************************************

baochun2 aatctcatggagagttcgatcctggctcaggatgaacgctggcggcatgcttaacacatg

baochun1 aatctcatggagagttcgatcctggctcaggatgaacgctggcggcatgcttaacacatg

************************************************************

baochun2 caagtcggacgggaagtggtgtttccagtggcggacgggtgagtaacgcgtaagaacctg

baochun1 caagtcggacgggaagtggtgtttccagtggcggacgggtgagtaacgcgtaagaacctg

************************************************************

baochun2 cccttgggaggggaacaacagctggaaacggctgctaataccccgtaggctgaggagcaa

baochun1 cccttgggaggggaacaacagctggaaacggctgctaataccccgtaggctgaggagcaa

************************************************************

baochun2 aaggaggaatccgcccgaggaggggctcgcgtctgattagctagttggtgaggcaatggc

baochun1 aaggaggaatccgcccgaggaggggctcgcgtctgattagctagttggtgaggcaatggc

************************************************************

baochun2 ttaccaaggcgatgatcagtagctggtccgagaggatgatcagccacactgggactgaga

baochun1 ttaccaaggcgatgatcagtagctggtccgagaggatgatcagccacactgggactgaga

************************************************************

baochun2 cacggcccagactcctacgggaggcagcagtggggaattttccgcaatgggcgaaagcct

baochun1 cacggcccagactcctacgggaggcagcagtggggaattttccgcaatgggcgaaagcct

************************************************************

baochun2 gacggagcaatgccgcgtggaggtagaaggcccacgggtcatgaacttcttttcccggag

baochun1 gacggagcaatgccgcgtggaggtagaaggcccacgggtcatgaacttcttttcccggag

************************************************************

baochun2 aagaagcaatgacggtatctggggaataagcatcggctaactctgtgccagcagccgcgg

baochun1 aagaagcaatgacggtatctggggaataagcatcggctaactctgtgccagcagccgcgg

************************************************************

baochun2 taatacagaggatgcaagcgttatccggaatgattgggcgtaaagcgtctgtaggtggct

baochun1 taatacagaggatgcaagcgttatccggaatgattgggcgtaaagcgtctgtaggtggct

************************************************************

baochun2 ttttaagtccgccgtcaaatcccagggctcaaccctggacaggcggtggaaactaccaag

baochun1 ttttaagtccgccgtcaaatcccagggctcaaccctggacaggcggtggaaactaccaag

************************************************************

baochun2 ctggagtacggtaggggtagagggaatttccggtggagcggtgaaatgcgtagagatcgg

baochun1 ctggagtacggtaggggtagagggaatttccggtggagcggtgaaatgcgtagagatcgg

************************************************************

baochun2 aaagaacaccaacggcgaaagcactctgctgggccgacactgacactgagagacgaaagc

baochun1 aaagaacaccaacggcgaaagcactctgctgggccgacactgacactgagagacgaaagc

************************************************************

baochun2 taggggagcgaatgggattagataccccagtagtcctagccgtaaacgatggatactagg

baochun1 taggggagcgaatgggattagataccccagtagtcctagccgtaaacgatggatactagg

************************************************************

baochun2 cgctgtgcgtatcgacccgtgcagtgctgtagctaacgcgttaagtatcccgcctgggga

baochun1 cgctgtgcgtatcgacccgtgcagtgctgtagctaacgcgttaagtatcccgcctgggga

************************************************************

baochun2 gtacgttcgcaagaatgaaactcaaaggaattgacgggggcccgcacaagcggtggagca

baochun1 gtacgttcgcaagaatgaaactcaaaggaattgacgggggcccgcacaagcggtggagca

************************************************************

baochun2 tgtggtttaattcgatgcaaagcgaagaaccttaccagggcttgacatgccgcgaatcct

baochun1 tgtggtttaattcgatgcaaagcgaagaaccttaccagggcttgacatgccgcgaatcct

************************************************************

baochun2 cttgaaagagaggggtgccttcgggaacgcggacacaggtggtgcatggctgtcgtcagc

baochun1 cttgaaagagaggggtgccttcgggaacgcggacacaggtggtgcatggctgtcgtcagc

************************************************************

baochun2 tcgtgccgtaaggtgttgggttaagtcccgcaacgagcgcaaccctcgtgtttagttgcc

baochun1 tcgtgccgtaaggtgttgggttaagtcccgcaacgagcgcaaccctcgtgtttagttgcc

************************************************************

baochun2 atcattgagtttggaaccctgaacagactgccggtgataagccggaggaaggtgaggatg

baochun1 atcattgagtttggaaccctgaacagactgccggtgataagccggaggaaggtgaggatg

************************************************************

baochun2 acgtcaagtcatcatgccccttatgccctgggcgacacacgtgctacaatggccgggaca

baochun1 acgtcaagtcatcatgccccttatgccctgggcgacacacgtgctacaatggccgggaca

************************************************************

baochun2 aagggtcgcgatcccgcgagggtgagctaactccaaaaacccgtcctcagttcggattgc

baochun1 aagggtcgcgatcccgcgagggtgagctaactccaaaaacccgtcctcagttcggattgc

************************************************************

baochun2 aggctgcaactcgcctgcatgaagccggaatcgctagtaatcgccggtcagccatacggc

baochun1 aggctgcaactcgcctgcatgaagccggaatcgctagtaatcgccggtcagccatacggc

************************************************************

baochun2 ggtgaattcgttcccgggccttgtacacaccgcccgtcacactatgggagctggccatgc

baochun1 ggtgaattcgttcccgggccttgtacacaccgcccgtcacactatgggagctggccatgc

************************************************************

baochun2 ccgaagtcgttaccttaaccgcaaggagggggatgccgaaggcagggctagtgactggag

baochun1 ccgaagtcgttaccttaaccgcaaggagggggatgccgaaggcagggctagtgactggag

************************************************************

baochun2 tgaagtcgtaacaaggtagccgtactggaaggtgcggctggatcacctccttttcaggga

baochun1 tgaagtcgtaacaaggtagccgtactggaaggtgcggctggatcacctccttttcaggga

************************************************************

baochun2 gagctaatgcttgttgggtattttggtttgacactgcttcacacccaaaaagaggggagc

baochun1 gagctaatgcttgttgggtattttggtttgacactgcttcacacccaaaaagaggggagc

************************************************************

baochun2 tacgtatgagttaaacttggagatggaagtcttctttcgtttctcgacggtgaagtaaga

baochun1 tacgtatgagttaaacttggagatggaagtcttctttcgtttctcgacggtgaagtaaga

************************************************************

baochun2 ccaagctcatgagcttattatcctaggtcggaacaagttgataggatcccctttttacgt

baochun1 ccaagctcatgagcttattatcctaggtcggaacaagttgataggatcccctttttacgt

************************************************************

baochun2 ccccatgccccccgtgtggcgacatgggggcgaaaaaaggaaagagagagatggggtttc

baochun1 ccccatgccccccgtgtggcgacatgggggcgaaaaaaggaaagagagagatggggtttc

************************************************************

baochun2 tctcgcttttggcatagcgggcccccagtggggggctcacacgacgggcttttagctcag

baochun1 tctcgcttttggcatagcgggcccccagtggggggctcacacgacgggcttttagctcag

************************************************************

baochun2 tggtagagcgcgcccctgataattgcgtcgttgtgcctgggctgtgagggctctcagcca

baochun1 tggtagagcgcgcccctgataattgcgtcgttgtgcctgggctgtgagggctctcagcca

************************************************************

baochun2 catggatagttcaatgtgctcatcagcgcctgaccttgagatgtggatcatccaaggcac

baochun1 catggatagttcaatgtgctcatcagcgcctgaccttgagatgtggatcatccaaggcac

************************************************************

baochun2 attagcatggcgtactcctcctgttcaaaccggggtttgaaaccaaactcctcctcagaa

baochun1 attagcatggcgtactcctcctgttcaaaccggggtttgaaaccaaactcctcctcagaa

************************************************************

baochun2 ggatagatggggcgattcaggtgagatccaatgtagatccaactttcgattcactcgtgg

baochun1 ggatagatggggcgattcaggtgagatccaatgtagatccaactttcgattcactcgtgg

************************************************************

baochun2 gatccgggcggtccgggggggaccaccgcggctcctctcttctcgagaatctatacatcc

baochun1 gatccgggcggtccgggggggaccaccgcggctcctctcttctcgagaatctatacatcc

************************************************************

baochun2 cttatcagtgtatggacagctatctctcgagcacaggtttaggttcgacctcaatgggaa

baochun1 cttatcagtgtatggacagctatctctcgagcacaggtttaggttcgacctcaatgggaa

************************************************************

baochun2 aaaaatggagcacctaacaacgcatcttcacagaccaaggactacgagatcacccctttc

baochun1 aaaaatggagcacctaacaacgcatcttcacagaccaagaactacgagatcacccctttc

***************************************.********************

baochun2 attctggggtgacggagggatcgtaccattcgagccgttttttttttcatgcttttcccg

baochun1 attctggggtgacggagggatcgtaccattcgagccattttttttttcatgcttttcccg

************************************.***********************

baochun2 gaagtctggagcaagactagttatctatctcttgactcgaaatgggagcaggtttgaaaa

baochun1 gaagtctggagcaagactagttatctatctcttgactcgaaatgggagcaggtttgaaaa

************************************************************

baochun2 aggatcttagagtgtctagggttggcccaggagggtctcttaaggccttcttttttcttc

baochun1 aggatcttagagtgtctagggttggcccaggagggtctcttaaggccttcttttttcttc

************************************************************

baochun2 tcatcggagttatttcacaaagacttgccgtggtaaggaagaagggaagaacaagcacac

baochun1 tcatcggagttatttcacaaagacttgccgtggtaaggaagaagggaagaacaagcacac

************************************************************

baochun2 ttggagagcgcagtacaacggagagttgtatgctgcgttcgggaaggatgaatcgctccc

baochun1 ttggagagcgcagtacaacggagagttgtatgctgcgttcgggaaggatgaatcgctccc

************************************************************

baochun2 gaaaaagaatctattgattctcttccaattggttggaccgtaggtgcgatgatttacttc

baochun1 gaaaaagaatctattgattctcttccaattggttggaccgtaggtgcgatgatttacttc

************************************************************

baochun2 acgggcgaggtctctggttcaagtccaggatggcccagctgcgccagggaaaagaataga

baochun1 acgggcgaggtctctggttcaagtccaggatggcccagctgcgccagggaaaagaataga

************************************************************

baochun2 agaagcatctgactccttcatgcatgctccacttggctcagggggatatagctcagttgg

baochun1 agaagcatctgactccttcatgcatgctccacttggctcagggggatatagctcagttgg

************************************************************

baochun2 tagagctccgctcttgcaattgggtcgttgcgattacgggttggatgtctaaatgtccag

baochun1 tagagctccgctcttgcaattgggtcgttgcgattacgggttggatgtctaaatgtccag

************************************************************

baochun2 gcggtaatgatagtatcttgtacctgaaccggtggctcactttttctaagtaatggggaa

baochun1 gcggtaatgatagtatcttgtacctgaaccggtggctcactttttctaagtaatggggaa

************************************************************

baochun2 gaggaccgaaacatgccactgaaagactctactgagacaaagatgggctgtcaagaacgt

baochun1 gaggaccgaaacatgccactgaaagactctactgagacaaagatgggctgtcaagaacgt

************************************************************

baochun2 agaggaggtaggatgggcagttggtcagatctagtatggatcgtacatggacggtagttg

baochun1 agaggaggtaggatgggcagttggtcagatctagtatggatcgtacatggacggtagttg

************************************************************

baochun2 gagtcggcggctctcctagggttccctcatctgggatccctggggaagaggatcaagttg

baochun1 gagtcggcggctctcctagggttccctcatctgggatccctggggaagaggatcaagttg

************************************************************

baochun2 gcccttgcgaacagcttgatgcactatctcccttcaaccctttgagcgaaatgcggcaat

baochun1 gcccttgcgaacagcttgatgcactatctcccttcaaccctttgagcgaaatgcggcaat

************************************************************

baochun2 aaggaaggaaaatccatggaccaaccccatcatctccaccccgtaggaactacgagatca

baochun1 aaggaaggaaaatccatggaccaaccccatcatctccaccccgtaggaactacgagatca

************************************************************

baochun2 ccccaaggacgccttcggcatccaggggtcacggaccgaccatagaaccctgctcaataa

baochun1 ccccaaggacgccttcggcatccaggggtcacggaccgaccatagaaccctgctcaataa

************************************************************

baochun2 gtggaacgcattagctgtccgctctcaggttgggcagtaagggtcggagaagggcaatca

baochun1 gtggaacgcattagctgtccgctctcaggttgggcagtaagggtcggagaagggcaatca

************************************************************

baochun2 ctcattcttaaaacccgcgttcttaagaccaaagagtcgggcggaaaaggggggaaagct

baochun1 ctcattcttaaaacccgcgttcttaagaccaaagagtcgggcggaaaaggggggaaagct

************************************************************

baochun2 ctccgttcctggttctcctgtagctggatcctccggaaccataagaatccttagttagaa

baochun1 ctccgttcctggttctcctgtagctggatcctccggaaccataagaatccttagttagaa

************************************************************

baochun2 tgggattccaactcagtaccccttgagtgagattttgagaagagttgctctttggagagc

baochun1 tgggattccaactcagtaccccttgagtgagattttgagaagagttgctctttggagagc

************************************************************

baochun2 acagtacgatgaaagttgtaagctgtgttcgggggggagttattgtctatcgttggcctc

baochun1 acagtacgatgaaagttgtaagctgtgttcgggggggagttattgtctatcgttggcctc

************************************************************

baochun2 tatggtagaataagtcgggggacctgagaggcggtggtttaccctgcggcggatgtcagc

baochun1 tatggtagaataagtcgggggacctgagaggcggtggtttaccctgcggcggatgtcagc

************************************************************

baochun2 ggttcgagtccgcttatctccaactcgtgaacttagccgatacaaagctagatgatagca

baochun1 ggttcgagtccgcttatctccaactcgtgaacttagccgatacaaagctagatgatagca

************************************************************

baochun2 acccatttttcagattcgtcggttcgatctatgatttctcatttatggacgttgataaga

baochun1 acccatttttcagattcggcggttcgatctatgatttatcatttatggacgttgataaga

****************** ****************** **********************

baochun2 tccttccatttagcagcaccttaggatggcatagccttaaagttcaaagtgaagggcgag

baochun1 tccttccatttagcagcaccttaggatggcatagccttaaagttcaaagtgaagggcgag

************************************************************

baochun2 gttcaaacgaggaaaggcttacggtggatacctaggcacccagagacgaggaagggcgta

baochun1 gttcaaacgaggaaaggcttacggtggatacctaggcacccagagacgaggaagggcgta

************************************************************

baochun2 gtaagcgacgaaatgcttcggggagttgaaaataagcatagatccggagattcccgaata

baochun1 gtaagcgacgaaatgcttcggggagttgaaaataagcatagatccggagattcccgaata

************************************************************

baochun2 ggtcaacctttcgaactgctgctgaatccatgggcagacaagagacaacctggcgaactg

baochun1 ggtcaacctttcgaactgctgctgaatccatgggcagacaagagacaacctggcgaactg

************************************************************

baochun2 aaacatcttagtagccagaggaaaagaaagcaaaagcgattcccgtagtagcggcgagcg

baochun1 aaacatcttagtagccagaggaaaagaaagcaaaagcgattcccgtagtagcggcgagcg

************************************************************

baochun2 aaatgggagcagcctaaaccgtgaaaacggggttgtgggagagcaatacaagcgtcgtgc

baochun1 aaatgggagcagcctaaaccgtgaaaacggggttgtgggagagcaatacaagcgtcgtgc

************************************************************

baochun2 tgctaggcgaagcggtggagtactgcaccctagatggcgaaagtccagtagccgaaagca

baochun1 tgctaggcgaagcggtggagtactgcaccctagatggcgaaagtccagtagccgaaagca

************************************************************

baochun2 tcactagcttatgctctgacccgagtagcatgggacacgtggaatcccgtgtgaatcagc

baochun1 tcactagcttatgctctgacccgagtagcatgggacacgtggaatcccgtgtgaatcagc

************************************************************

baochun2 aaggaccaccttgcaaggctaaatactcctgggtgaccgatagcgaagtagtaccgtgag

baochun1 aaggaccaccttgcaaggctaaatactcctgggtgaccgatagcgaagtagtaccgtgag

************************************************************

baochun2 ggaagggtgaaaagaacccccatcggggagtgaaatagaacatgaaaccgtaagctccca

baochun1 ggaagggtgaaaagaacccccatcggggagtgaaatagaacatgaaaccgtaagctccca

************************************************************

baochun2 agcagtgggaggagcccagggctctgaccgcgtgcctgttgaagaatgagccggcgactc

baochun1 agcagtgggaggagcccagggctctgaccgcgtgcctgttgaagaatgagccggcgactc

************************************************************

baochun2 ataggcagtggcttggttaagggaacccaccggagccgtagcgaaagcgagtcttcatag

baochun1 ataggcagtggcttggttaagggaacccaccggagccgtagcgaaagcgagtcttcatag

************************************************************

baochun2 ggcaattgtcactgcttatggacccgaacctgggtgatctatccatgaccaggatgaagc

baochun1 ggcaattgtcactgcttatggacccgaacctgggtgatctatccatgaccaggatgaagc

************************************************************

baochun2 ttgggtgaaactaagtggaggtccgaaccgactgatgttgaagaatcagcggatgagttg

baochun1 ttgggtgaaactaagtggaggtccgaaccgactgatgttgaagaatcagcggatgagttg

************************************************************

baochun2 tggttaggggtgaaatgccactcgaacccagagctagctggttctccccgaaatgcgttg

baochun1 tggttaggggtgaaatgccactcgaacccagagctagctggttctccccgaaatgcgttg

************************************************************

baochun2 aggcgcagcagttgactggacatctaggggtaaagcactgtttcggtgcgggccgcgaga

baochun1 aggcgcagcagttgactggacatctaggggtaaagcactgtttcggtgcgggccgcgaga

************************************************************

baochun2 gcggtaccaaatcgaggcaaactctgaatactagatatgacctcaaaataacaggggtca

baochun1 gcggtaccaaatcgaggcaaactctgaatactagatatgacctcaaaataacaggggtca

************************************************************

baochun2 aggtcggccagtgagacgatgggggataagcttcatcgtcgagagggaaacagcccggat

baochun1 aggtcggccagtgagacgatgggggataagcttcatcgtcgagagggaaacagcccggat

************************************************************

baochun2 caccagctaaggcccctaaatgatcgctcagtgataaaggaggtaggggtgcagagacag

baochun1 caccagctaaggcccctaaatgatcgctcagtgataaaggaggtaggggtgcagagacag

************************************************************

baochun2 ccaggaggtttgcctagaagcagccacccttgaaagagtgcgtaatagctcactgatcga

baochun1 ccaggaggtttgcctagaagcagccacccttgaaagagtgcgtaatagctcactgatcga

************************************************************

baochun2 gcgctcttgcgccgaagatgaacggggctaagcgatctgccgaagctgtgggatgtaaaa

baochun1 gcgctcttgcgccgaagatgaacggggctaagcgatctgccgaagctgtgggatgtaaaa

************************************************************

baochun2 atacatcggtaggggagcgttccgccttagagggaagtccccgcgcgagcgagggtggac

baochun1 atacatcggtaggggagcgttccgccttagagggaagtccccgcgcgagcgagggtggac

************************************************************

baochun2 gaagcggaagcgagaatgtcggcttgagtaacgcaaacattggtgagaatccaatgcccc

baochun1 gaagcggaagcgagaatgtcggcttgagtaacgcaaacattggtgagaatccaatgcccc

************************************************************

baochun2 gaaaacctaagggttcctccgcaaggttcgtccacggagggtgagtcagggcctaagatc

baochun1 gaaaacctaagggttcctccgcaaggttcgtccacggagggtgagtcagggcctaagatc

************************************************************

baochun2 aggccgaaaggcgtagtcgatggacaacaggtgaatattcctgtactaccccttgttggt

baochun1 aggccgaaaggcgtagtcgatggacaacaggtgaatattcctgtactaccccttgttggt

************************************************************

baochun2 cccgagggacggaggaagctaggttagccgaaagatggttatcggttcaaggacgtaagg

baochun1 cccgagggacggaggaggctaggttagccgaaagatggttatcggttcaaggacgtaagg

****************.*******************************************

baochun2 tgaccctgttttttcagggtaagaaggggtagagaaaatgcctcgagccaatgttcgagt

baochun1 tgaccctgttttttcagggtaagaaggggtagagaaaatgcctcgagccaatgttcgagt

************************************************************

baochun2 accaggcgctacggcgctgaagtaacccatgccatactcccaggaaaagctcgaacgacc

baochun1 accaggcgctacggcgctgaagtaacccatgccatactcccaggaaaagctcgaacgacc

************************************************************

baochun2 ttcaacaaaagggtacctgtacccgaaaccgacacaggtgggtaggtagagaatacctag

baochun1 ttcaacaaaagggtacctgtacccgaaaccgacacaggtgggtaggtagagaatacctag

************************************************************

baochun2 gggcgcgagacaactctctctaaggaactcggcaaaatagccccgtaacttcgggagaag

baochun1 gggcgcgagacaactctctctaaggaactcggcaaaatagccccgtaacttcgggagaag

************************************************************

baochun2 gggtgcctcctcacaaagggggtcgcagtgaccaggcccgggcgactgtttaccaaaaac

baochun1 gggtgcctcctcacaaagggggtcgcagtgaccaggcccgggcgactgtttaccaaaaac

************************************************************

baochun2 acaggtctccgcaaagtcgtaagaccatgtatgggggctgacgcctgcccagtgccggaa

baochun1 acaggtctccgcaaagtcgtaagaccatgtatgggggctgacgcctgcccagtgccggaa

************************************************************

baochun2 ggtcaaggaagttggtgacctgatgacaggggagccggcgaccgaagccccggtgaacgg

baochun1 ggtcaaggaagttggtgacctgatgacaggggagccggcgaccgaagccccggtgaacgg

************************************************************

baochun2 cggccgtaactataacggtcctaaggtagcgaaattccttgtcgggtaagttccgacccg

baochun1 cggccgtaactataacggtcctaaggtagcgaaattccttgtcgggtaagttccgacccg

************************************************************

baochun2 cacgaaaggcgtaacgatctgggcactgtctcggagagagactcggtgaaatagacatgt

baochun1 cacgaaaggcgtaacgatctgggcactgtctcggagagagactcggtgaaatagacatgt

************************************************************

baochun2 ctgtgaagatgcggactacctgcacctggacagaaagaccctatgaagctttactgttcc

baochun1 ctgtgaagatgcggactacctgcacctggacagaaagaccctatgaagctttactgttcc

************************************************************

baochun2 ctgggattggctttgggcctttcctgcgcagcttaggtggaaggcgaagaaggccccctt

baochun1 ctgggattggctttgggcctttcctgcgcagcttaggtggaaggcgaagaaggccccctt

************************************************************

baochun2 ccgggggggcctgagccatcagtgagataccactctggaagagctagaattctaaccttg

baochun1 ccgggggggcctgagccatcagtgagataccactctggaagagctagaattctaaccttg

************************************************************

baochun2 tgccaggacctacgggccaagggacattctcaggtagacagtttctatggggcgtaggcc

baochun1 tgccaggacctacgggccaagggacattctcaggtagacagtttctatggggcgtaggcc

************************************************************

baochun2 tcccaaaaggtaacggaggcgtgcaaaggtttcctcgggccggacggagattggccctcg

baochun1 tcccaaaaggtaacggaggcgtgcaaaggtttcctcgggccggacggagattggccctcg

************************************************************

baochun2 agtgcaaaggcagaagggagcttgactgcaagacccacccgtcgagcagggacgaaagtc

baochun1 agtgcaaaggcagaagggagcttgactgcaagacccacccgtcgagcagggacgaaagtc

************************************************************

baochun2 ggccttagtgatccgacggtgccgagtggaagggccgtcgctcaacggataaaagttact

baochun1 ggccttagtgatccgacggtgccgagtggaagggccgtcgctcaacggataaaagttact

************************************************************

baochun2 ctagggataacaggctgatcttccccaagagctcacatcgacgggaaggtttggcacctc

baochun1 ctagggataacaggctgatcttccccaagagctcacatcgacgggaaggtttggcacctc

************************************************************

baochun2 gatgtcggctcttcgccacctggggctgtagtatgttccaagggttgggctgttcgccca

baochun1 gatgtcggctcttcgccacctggggctgtagtatgttccaagggttgggctgttcgccca

************************************************************

baochun2 ttaaagcggtacgtgagctgggttcagaacgtcgtgagacagttcggtccatatccggtg

baochun1 ttaaagcggtacgtgagctgggttcagaacgtcgtgagacagttcggtccatatccggtg

************************************************************

baochun2 tgggcgttagagcattgagaggacctttccctagtacgagaggaccgggaaggacgcacc

baochun1 tgggcgttagagcattgagaggacctttccctagtacgagaggaccgggaaggacgcacc

************************************************************

baochun2 tctggtgtaccagttatcgtgcccacggtaaacgctgggtagccaagtgcggagcggata

baochun1 tctggtgtaccagttatcgtgcccacggtaaacgctgggtagccaagtgcggagcggata

************************************************************

baochun2 actgctgaaagcatctaagtagtaagcccaccccaagatgagtgctctcctattccgact

baochun1 actgctgaaagcatctaagtagtaagcccaccccaagatgagtgctctcctattccgact

************************************************************

baochun2 tccccagagcctccggtagcacagccgagacagcgacgggttctctgcccctgcggggat

baochun1 tccccagagcctccggtagcacagccgagacagcgacgggttctctgcccctgcggggat

************************************************************

baochun2 ggagcgacagaagttttgagaattcaagagaaggtcacggcgagacgagccgtttatcat

baochun1 ggagcgacagaagttttgagaattcaagagaaggtcacggcgagacgagccgtttatcat

************************************************************

baochun2 tacgataggtgtcaagtggaagtgcagtgatgtatgcagctgaggcatcctaacagaccg

baochun1 tacgataggtgtcaagtggaagtgcagtgatgtatgcagctgaggcatcctaacagaccg

************************************************************

baochun2 gtagacttgaaccttgttcctacatgatccgatcaattcgatcaggtactcgccatctat

baochun1 gtagacttgaaccttgttcctacatgatccgatcaattcgatcaggtactcgccatctat

************************************************************

baochun2 tttcattgttcaactctttgacaacacgaaaaaaccaaaatatctgccctccctctctat

baochun1 tttcattgttcaactctttgacaacacgaaaaaaccaaaatatctgccctccctctctat

************************************************************

baochun2 ccatccaaggctagggatggaagggcagaggcctttggtgtcccttccagtcaagaattg

baochun1 ccatccaaggctagggatggaagggcagaggcctttggtgtcccttccagtcaagaattg

************************************************************

baochun2 gggcctcagaatcactagccaatatgcttttctctcatgcctttcttcgttcatagttcg

baochun1 gggcctcagaatcactagccaatatgcttttctctcatgcctttcttcgttcatagttcg

************************************************************

baochun2 atattctggtgtcctaggcgtagaggaaccacaccaatccatcccgaacttggtggttaa

baochun1 atattctggtgtcctaggcgtagaggaaccacaccaatccatcccgaacttggtggttaa

************************************************************

baochun2 actctactgcggtgacgatactgtaggggaggtcctgcggaaaaatagctcgacgccagg

baochun1 actctactgcggtgacgatactgtaggggaggtcctgcggaaaaatagctcgacgccagg

************************************************************

baochun2 atgataaaaagcttaacaccccccattcttattactttttcaatatgaaaagaaaaaaca

baochun1 atgataaaaagcttaacaccccccattcttattactttttcaatatgaaaagaaaaaaca

************************************************************

baochun2 aatgaaaaaagaaagggtcgtcttattcaaaaccccaatcatgatatcccctctctccca

baochun1 aatgaaaaaagaaagggtcgtcttattcaaaaccccaatcatgatatcccctctctccca

************************************************************

baochun2 ctccacacctcggaacgcaccgttcttatagagagaaacgcgctttcacatcttcttaat

baochun1 ctccacacctcggaacgcaccgttcttatagagagaaacgcgctttcacatcttcttaat

************************************************************

baochun2 ccgaaataatgggaaataatggctggggagagaaaggttctttttttgagggtactcccg

baochun1 ccgaaataatgggaaataatggctggggagagaaaggttctttttttgagggtactcccg

************************************************************

baochun2 ggaacagatccagtggagacggggtggggcctgtagctcagaggattagagcacgtggct

baochun1 ggaacagatccagtggagacggggtggggcctgtagctcagaggattagagcacgtggct

************************************************************

baochun2 acgaaccacggtgtcgggggttcgaatccctcctcgcccacaaccggcccaaaggggaag

baochun1 acgaaccacggtgtcgggggttcgaatccctcctcgcccacaaccggcccaaaggggaag

************************************************************

baochun2 gccctttccctctgagggtaggaaaatcatgatcgggatagcggaccaaaagctatggaa

baochun1 gccctttccctctgagggtaggaaaatcatgatcgggatagcggaccaaaagctatggaa

************************************************************

baochun2 cttgggtataggtcttttgtcgaaatggaatggccctactttgtctttttatttatcgtt

baochun1 cttgggtataggtcttttgtcgaaatggaatggccctactttgtctttttatttatcgtt

************************************************************

baochun2 gatgttagaatcattacacatagtatgccctaaacgtccgcgtatttttttgttttacgt

baochun1 gatgttagaatcattacacatagtatgccctaaacgtccgcgtatttttttgttttacgt

************************************************************

baochun2 cccgcaacccttcctcagcccggcttggacagaatagcagagcaagtacaagtattagta

baochun1 cccgcaacccttcctcagcccggcttggacagaatagcagagcaagtacaagtattagta

************************************************************

baochun2 gcataaaaacaatgccttcctcgtcattaatatgtttgctcgcggtaattgtggcctctc

baochun1 gcataaaaacaatgccttcctcgtcattaatatgtttgctcgcggtaattgtggcctctc

************************************************************

baochun2 gggagaatcgatgactgcatctttgatgcactgctagtactagtacatctgagaattctt

baochun1 gggagaatcgatgactgcatctttgatgcactgctagtactagtacatctgagaattctt

************************************************************

baochun2 aattggctagttgtaaatagccccaaggctatggaacaaagaattatcccagacctacac

baochun1 aattggctagttgtaaatagccccaaggctatggaacaaagaattatcccagacctacac

************************************************************

baochun2 cgaggttctgaattctttcattcagaatgaatcaaatctccccaagtaggattcgaacct

baochun1 cgaggttctgaattctttcattcagaatgaatcaaatctccccaagtaggattcgaacct

************************************************************

baochun2 acgaccaatcagttaacagccgaccgctctaccactgagctactgaggaacaacgggaga

baochun1 acgaccaatcagttaacagccgaccgctctaccactgagctactgaggaacaacgggaga

************************************************************

baochun2 ttaaatctcatagagttcaattcccgttctcaacctatgaccaatatgagctcgaagctt

baochun1 ttaaatctcatagagttcaattcccgttctcaacctatgaccaatatgagctcgaagctt

************************************************************

baochun2 ccttcgtaactccaggaacttcttcgtagtggctcccttccatgcctcatttcataggga

baochun1 ccttcgtaactccaggaacttcttcgtagtggctcccttccatgcctcatttcataggga

************************************************************

baochun2 acttcaaagtgaccctatttcattatattccatccatatcccaattccattcatttacta

baochun1 acttcaaagtgaccctatttcattatattccatccatatcccaattccattcatttacta

************************************************************

baochun2 tccctttggtgtcattgacataatagatgtcgtttctagtctatctctttatatggaaag

baochun1 tccctttggtgtcattgacataatagatgtcgtttctagtctatctctttatatggaaag

************************************************************

baochun2 ttcaaaaatcatcatataataatccagaaattgcaatagaaaagaaaaagggaggtttgt

baochun1 ttcaaaaatcatcatataataatccagaaattgcaatagaaaagaaaaagggaggtttgt

************************************************************

baochun2 gatgattttaaaatcttttctactaggtaatctagtatccttatgcctgaagataatcaa

baochun1 gatgattttaaaatcttttctactaggtaatctagtatccttatgcctgaagataatcaa

************************************************************

baochun2 ttcggtcgttgtggtcggactctattatggatttctgaccacattctctatagggccctc

baochun1 ttcggtcgttgtggtcggactctattatggatttctgaccacattctctatagggccctc

************************************************************

baochun2 ttatctctttcttctccgagctcaggttatggaagaaggaaccgagaagaaggtatcagc

baochun1 ttatctctttcttctccgagctcaggttatggaagaaggaaccgagaagaaggtatcagc

************************************************************

baochun2 aacaactggttttattacgggacagctcatgatgttcatatcgatctattatgcgccaat

baochun1 aacaactggttttattacgggacagctcatgatgttcatatcgatctattatgcgccaat

************************************************************

baochun2 gcatctagcattgggtagacctcatacaataactgtcctagctctaccctatcttttgtt

baochun1 gcatctagcattgggtagacctcatacaataactgtcctagctctaccctatcttttgtt

************************************************************

baochun2 tcatttcttctggaacaatcacaaaaacttttttgattatggatctactaccagaaattc

baochun1 tcatttcttctggaacaatcacaaaaacttttttgattatggatctactaccagaaattc

************************************************************

baochun2 aatgcgtaatctcagcattcaatgtgtattcctgaataatcttatttttcaattattcaa

baochun1 aatgcgtaatctcagcattcaatgtgtattcctgaataatcttatttttcaattattcaa

************************************************************

baochun2 ccatttcattttaccaagttcaatgttagccagattagtcaacatttatatgtttcgatg

baochun1 ccatttcattttaccaagttcaatgttagccagattagtcaacatttatatgtttcgatg

************************************************************

baochun2 caacaacaagatgttatttgtaacaagtagttttgttggttggttaattggtcacatttt

baochun1 caacaacaagatgttatttgtaacaagtagttttgttggttggttaattggtcacatttt

************************************************************

baochun2 attcatgaaatggcttggattggtattagtctggatacggcaaaatcgttctattagatc

baochun1 attcatgaaatggcttggattggtattagtctggatacggcaaaatcgttctattagatc

************************************************************

baochun2 aaatgtacttattcgatctaataagtaccttgtgtcagaattgagaaattctatggctag

baochun1 aaatgtacttattcgatctaataagtaccttgtgtcagaattgagaaattctatggctag

************************************************************

baochun2 gatctttagtattctcttatttattacctgtgtctactatttaggcagaataccgtcacc

baochun1 gatctttagtattctcttatttattacctgtgtctactatttaggcagaataccgtcacc

************************************************************

baochun2 cattcttactaagaaactgaaagaaacctcaaaaacggaagaaagagatgtagaaataga

baochun1 cattcttactaagaaactgaaagaaacctcaaaaacggaagaaagagatgtagaaataga

************************************************************

baochun2 aacaacttacgaaacgaagaggactaaacaggaacaagagggatccaccgaagaagatac

baochun1 aacaacttacgaaacgaagaggactaaacaggaacaagagggatccaccgaagaagatac

************************************************************

baochun2 ttctccttcccttttttcggaagaaaaggaggatacggacaaaatcgatcaaacgaaaga

baochun1 ttctccttcccttttttcggaagaaaaggaggatacggacaaaatcgatcaaacgaaaga

************************************************************

baochun2 gatccgagtgaatagaaaagaaaaaaaagaaatacaaaaaaagattaatacataagataa

baochun1 gatccgagtgaatagaaaagaaaaaaaataaatacaaaaaaagattaatacataagataa

**************************** *******************************

baochun2 atacaaaaaaagataagatgagatgcgccccccccctacatatttgatgccttctcctag

baochun1 atacaaaaaaagataagatgagatgcgccccccccctacatatttgatgccttctcctag

************************************************************

baochun2 aaagaaacttataataccaactccattcgtaattccatcaattatccgtctatcaataaa

baochun1 aaagaaacttataataccaactccattcgtaattccatcaattatccgtctatcaataaa

************************************************************

baochun2 atcggtgaattctgccaatactcttatacctctagttaagaatgttgcataaaaagcatc

baochun1 atcggtgaattctgccaatactcttatacctctagttaagaatgttgcataaaaagcatc

************************************************************

baochun2 aatataaccacgattatctgaccaattatatatgatatttattgttttatccaaaaagtt

baochun1 aatataaccacgattatctgaccaattatatatgatatttattgttttatccaaaaagtt

************************************************************

baochun2 tcttttagatctgattttaccaaatgaattaattaaatctaaatttttaaaagatgaata

baochun1 tcttttagatctgattttaccaaatgaattaattaaatctaaatttttaaaagatgaata

************************************************************

baochun2 aacaggtttatataaaaatagcgctataaatattcctaaataggctatactgactgaaaa

baochun1 aacaggtttatataaaaatagcgctataaatattcctaaataggctatactgactgaaaa

************************************************************

baochun2 aattgcatcttttccaaattcataccaatccatagactttttcaaattttgatgtaaaag

baochun1 aattgcatcttttccaaattcataccaatccatagactttttcaaattttgatgtaaaag

************************************************************

baochun2 atttattgaaggagttaaccatttggataatatatctaaatccattccttcttgattgaa

baochun1 atttattgaaggagttaaccatttggataatatatctaaatccattccttcttgattgaa

************************************************************

baochun2 aggaattcctatagatccaacacacaaagtaaataagaacaatacaagtagaggaaataa

baochun1 aggaattcctatagatccaacacacaaagtaaataagaacaatacaagtagaggaaataa

************************************************************

baochun2 catagtattatccgattcataaggatatgagtaatttattttattgccaaaattagaaat

baochun1 catagtattatccgattcataaggatatgagtaatttattttattgccaaaattagaaat

************************************************************

baochun2 agtaataaaaggtcgtgtcatctctcttatattctcgtcaatttgatatgtcgtctttgt

baochun1 agtaataaaaggtcgtgtcatctctcttatattctcatcaatttgatatgtcgtctttgt

************************************.***********************

baochun2 ttttaacaaagtactttcattattattcattaataaccaaatatttttgttaatattttt

baochun1 ttttaacaaagtactttcattattattcattaataaccaaatatttttgttaatattttt

************************************************************

baochun2 tgaatttcctttaccccatagagaaattgactgagtacttgttttaccatcgtaattttt

baochun1 tgaatttcctttaccccatagagaaattgactgagtacttgttttaccatcgtaattttt

************************************************************

baochun2 aaaataaatatttaaatgtccctcaaaagtaagtaaatagatcctaaacatataaaatgc

baochun1 aaaataaatatttaaatgtccctcaaaagtaagtaaatagatcctaaacatataaaatgc

************************************************************

baochun2 agttaatcctgccgtggaccaagctattaatgcgaaagtaggtgaatacaaccaagtatc

baochun1 agttaatcctgccgtggaccaagctattaatgcgaaagtaggtgaatacaaccaagtatc

************************************************************

baochun2 attaagaatctcatccttggaccaaaaacaagcaaggggcggaataccacaaagagacag

baochun1 attaagaatctcatccttggaccaaaaacaagcaaggggcggaataccacaaagagacag

************************************************************

baochun2 tgtacctaacaaaaaagaagtttttgtaattggcagatgttttgttaaacctcccataag

baochun1 tgtacctaacaaaaaagcagtttttgtaattggcagatgttttgttaaacctcccataag

***************** ******************************************

baochun2 aaccatattttgacttttttcgggagagtatccaacaacagtttccattgcatgaataac

baochun1 aaccatattttgacttttttcgggagagtatccaacaacagtttccattgcatgaataac

************************************************************

baochun2 tgatccagatcctaaaaacaataatgcttttgaataagcatgagtaattaaatgaaataa

baochun1 tgatccagatcctaaaaacaataatgcttttgaataagcatgagtaattaaatgaaataa

************************************************************

baochun2 agcaattcgataagaccctatacctagagctaacatcatatatcccaattgagacatggt

baochun1 agcaattcgataagaccccatacctagagctaacatcatatatcccaattgagacatggt

******************.*****************************************

baochun2 agaataagctaaacctctcttaatgtctttttgagctatagctaaagtagctcctaataa

baochun1 agaataagctaaacctctcttaatgtctttttgagctatagctaaagtagctcctaataa

************************************************************

baochun2 tactgttattatacctatgaaagagattaaattcattatataaggtataactgtgaaaag

baochun1 tactgttattatacctatgaaagagattaaattcattatataaggtataactgtgaaaag

************************************************************

baochun2 aggaagaagccgagcgacaagaaagatgccagcggctaccatagtagcagcatggataag

baochun1 aggaagaagccgagcgacaagaaagatgccagcggctaccatagtagcagcatggataag

************************************************************

baochun2 agcggaaataggagtgggcccctccatggcatcaggtaaccatacatgaagagggaattg

baochun1 agcggaaataggagtgggcccctccatggcatcaggtaaccatacatgaagagggaattg

************************************************************

baochun2 tgcggatttagcaactgcgccggcaaataatagaacagcaaaaaaaataacaaataaagc

baochun1 tgcggatttagcaactgcgccggcaaataatagaacagcaaaaaaaataacaaataaagc

************************************************************

baochun2 attgacttcattattataaatgaggttattaactatttcgaacatatcccgaaattcgaa

baochun1 attgacttcattattataaatgaggttattaactatttcgaacatatcccgaaattcgaa

************************************************************

baochun2 actacccgttatccaataaaaacctaaaattcctaataataaaccaaaatctcctactcg

baochun1 actacccgttatccaataaaaacctaaaattcctaataataaaccaaaatctcctactcg

************************************************************

baochun2 attagttacaaacgccttttgacaagcatttgccgcaataggccgtgtaaaccaaaaacc

baochun1 attagttacaaacgccttttgacaagcatttgccgcaataggccgtgtaaaccaaaaacc

************************************************************

baochun2 tattaacagatatgaacacattccaactaattcccaaaaaatataaatttgtatcaaatt

baochun1 tattaacagatatgaacacattccaactaattcccaaaaaatataaatttgtatcaaatt

************************************************************

baochun2 cgaactagtaactaatcctaacatggaagtactaaaaaaactcatataagcaaaaaatcg

baochun1 cgaactagtaactaatcctaacatggaagtactaaaaaaactcatataagcaaaaaatcg

************************************************************

baochun2 caaatatccttggtcatgagacatataattatcactataaataagaaccataattccaac

baochun1 caaatatccttggtcatgagacatataattatcactataaataagaaccataattccaac

************************************************************

baochun2 cgtagtgattaatattaacataatagaagtaagtggatcgatcaaataaccgagttctaa

baochun1 cgtagtgattaatattaacataatagaagtaagtggatcgatcaaataaccgagttctaa

************************************************************

baochun2 agaaaaatcattagtgatgatccaagaccatacatattgatatatggaactgctatttat

baochun1 agaaaaatcattagtgatgatccaagaccatacatattgatatatggaactgctatttat

************************************************************

baochun2 ttgctgaatagacagatttatcgacaaaatcatgactatacttaacaataaaccactctt

baochun1 ttgctgaatagacagatttatcgacaaaatcatgactatacttaacaataaaacactctt

**************************************************** *******

baochun2 aaaagcccacatacgacgaagcttttttgttgcgtttggaaaaagaagaagccccactcc

baochun1 aaaagcccacatacgacgaagcttttttgttgcgtttggaaaaagaagaagccccactcc

************************************************************

baochun2 tattaatacaggaactggaagtggaacaaaaggtattatccaggcatattcatatgtatg

baochun1 tattaatacaggaactggaagtggaacaaaaggtattatccaagcatattcatatgtatg

******************************************.*****************

baochun2 ttccataaaaataaaaaagtttttattctgaattgttttccaattaccgggccttttatc

baochun1 ttccataaaaataaaaaagtttttattctgaattgttttccaattaccgggccttttatc

************************************************************

baochun2 tttttaaaattggaaagggttaataaaaaaataaagatatgcattaatttaaactaacta

baochun1 tttttaaaattggaaagggttaataaaaaaataaagatatgcattaatttaaactaacta

************************************************************

baochun2 aaatcgaattttttattcttacttattaggtgtctttcttaaataaaaaaagattcaaat

baochun1 aaatcgaattttttattcttacttattatgtgtctttcttaaataaaaaaagattcaaat

**************************** *******************************

baochun2 taagaaggcacaattggtaaaataatatcactaatttataatttactagtataaaatatt

baochun1 taagaaggcacaattggtaaaataatatcactaatttataatttactagtataaaatatt

************************************************************

baochun2 agctatttactaaaactttttaggaagatattgaaaattacaattattattactcttaca

baochun1 agttatttactaaaactttttaggaagatgttgaaaattccaattattattactcttaca

**.**************************.********* ********************

baochun2 ataatttagatcaatacagcacaagcagaaataaaacactaaaaacaaaatattgaattt

baochun1 ataatttagatcaatacagcacaagcagaaataaaacactaaaaacaaaatattgaattt

************************************************************

baochun2 tgatataaatcatagtatgaactaagttaacttaatgaaataataactcccttttttctt

baochun1 tgatctaaattatagtatgaactaagttaacttaatgaaataataactcccttttttctt

**** *****.*************************************************

baochun2 tattatttcatttattcaaaataattaactagttcattctgaaattgattaattgatttc

baochun1 tattatttcatttattcaaaataattaactagttcattctgaaattgattaattgatttc

************************************************************

baochun2 tatctcaataaaatctcaataaaatacatttataggaaggaatctactacacgccgcttt

baochun1 tatctcaataaaatctcaataaaatacatttataggaaggaatctactacacgccgcttt

************************************************************

baochun2 attttcaattttagaaaactaaaaaaaaattactaaaaatattgtttataaagctatgtt

baochun1 attttcaattttagaaactaaagaaaaatttactaaaaatattgtttataaagctatgtt

***************** . **.***** *******************************

baochun2 atgtataattactaatttcattagaatttgtaaattacaattcagtgtatttttttctgg

baochun1 atgtataattactaatttcattagaatttgtaaattacaattcagtgtatttttttctgg

************************************************************

baochun2 atcttgatcaattaacaaatcacatgatgacatatcatatatatttttgatttaagtttg

baochun1 atcttgatcaattaacaaatcacatgatgacatatcatatatatttttgatttaagtttg

************************************************************

baochun2 aagaatgttttattctatttcgaatactattttttatgattttttttaagagaattctct

baochun1 aagaatgttttattctatttcgaatactattttttatgattttttttaagagaattctct

************************************************************

baochun2 taaaaaaaatcaataatgacatagcaaaaaaccacttcttttgaacaatagatgtctttc

baochun1 aaaaaaaaatcaataatgacatagcaaaaaaccacttcttttgaacaatagatgtctttc

***********************************************************

baochun2 acatccaatagaataaggagtaatctcttaattttcaaatggcagttccaaaaaaacgta

baochun1 acatccaatagaataaggagtaatctcttaattttcaaatggcagttccaaaaaaacgta

************************************************************

baochun2 cttctatatcaaaaaaacgcattcgtaaaaatagttggaaaaaaaaaggatattgggcgg

baochun1 cttctatatcaaaaaaacgcattcgtaaaaatagttggaaaaaaaaaggatattgggcgg

************************************************************

baochun2 ctttaaaagccttttccttgggtaaatctctttccaccgggcagtcaaaaagtttttttg

baochun1 ctttaaaagccttttccttgggtaaatctctttccaccgggcagtcaaaaagtttttttg

************************************************************

baochun2 ttcgacaaacaaataagtaataaaaaattgcgagaatctaaatttacatgactcaaaaag

baochun1 ttcgacaaacaaataagtaataaaaaattgcgagaatctaaatttacatgactcaaaaag

************************************************************

baochun2 ttgtcgaattttatatgtagactccatcgcctattcttttgaactaatcaatatgaaatt

baochun1 ttgtcgaattttatatgtagactccatcgcctattcttttgaactaatcaatatgaaatt

************************************************************

baochun2 ctttttgcatttgggtcttatgatttgcagaatgctaatttttgattattaaattcgtaa

baochun1 ctttttgcatttgggtcttatgatttgcagaatgctaatttttgattattaaattcgtaa

************************************************************

baochun2 aaaaaaagaaaagacttttttaagttctatccccctatccgggggtagaactagttagtt

baochun1 aaaaaaagaaaagacttttttaagttctatccccctatccgggggtagaactagttagtt

************************************************************

baochun2 ataataggtcaattgccgaagaggataaacttcacgaaaggccgaagggaaaaaaataaa

baochun1 ataataggtcaattgccgaagaggataaacttcacg------------------------

************************************

baochun2 aatgaatagtaatttaaagattcatttttattcatcaattttattctttcttcaaaaata

baochun1 ---------------aaagattcatttttattcatcaattttattctttcttcaaaaata

*********************************************

baochun2 ttctattgaattaacttttttagtctcgactattgaatagaaatacgctattataagttt

baochun1 ttctattgaattaacttttttagtctcgactattgaatagaaatacgctattataagttt

************************************************************

baochun2 gagcaagccgctatggtgaaattggtagacacgctgctcttaggaagcagtgctagagca

baochun1 gagcaagccgctatggtgaaattggtagacacgctgctcttaggaagcagtgctagagca

************************************************************

baochun2 tctcggttcgagtccgagtggcggcatgccctcttctaaaaaaaataaaatagattctat

baochun1 tctcggttcgagtccgagtggcggcatgccctcttctaaaaaaaataaaatagattctat

************************************************************

baochun2 aataaattcaattactgattttcccttcctaattaagggacccctttacttttttaaaaa

baochun1 aataaattcaattactgattttcccttcgtaattaagggacccctttactttttaaaaaa

**************************** ************************* *****

baochun2 tttttatgatatttttaactttagagcatatattaactcatatttccttttcgattattt

baochun1 tttttatgatatttttaactttagagcatatattaactcatatttccttttcgattgttt

********************************************************.***

baochun2 caattgtaattacaattcatttaataaccttattagtcgatgaaatcgtaaaactatctg

baochun1 caattgtaattacaattcatttaataaccttattagtcgatgaaatcgtaaaactatctg

************************************************************

baochun2 attcgtcaaaaaggggcatgatcgcggcctttttatgtataacaggattattagtaactc

baochun1 attcgtcaaaaaggggcatgatcgcggcctttttatgtataacaggattattagtaactc

************************************************************

baochun2 gttggatttattcgggacattttccattaagtaatttatatgaatcattaatctttcttt

baochun1 gttggatttattcgggacattttccattaagtaatttatatgaatcattaatctttcttt

************************************************************

baochun2 catggagtttctccattattcatattgttccgtattttaaaaaaaagaaaaatgatttaa

baochun1 catggagtttctccattattcatattgttccgtattttaaaaaaaagaaaaatgatttaa

************************************************************

baochun2 gtacaataacggcaccaagtgttctttttacacaaggctttgctacttcaggtcttttaa

baochun1 gtacaataacggcaccaagtgttctttttacacaaggctttgctacttcaggtcttttaa

************************************************************

baochun2 ctgaaatacatcaatccgaaatattagtacctgctctccaatctgagtggttaataatgc

baochun1 ctgaaatacatcaatccgaaatattagtacctgctctccaatctgagtggttaataatgc

************************************************************

baochun2 acgtaagtatgatggtattggggtatgcagctcttttatgcggatcattattatcagtag

baochun1 acgtaagtatgatggtattggggtatgcagctcttttatgcggatcattattatcagtag

************************************************************

baochun2 cacttttagtcattacatttcgaaaagacataagacctttttataataaaaaccatgtat

baochun1 cacttttagtcattacatttcgaaaagacataagacctttttataataaaaaccatgtat

************************************************************

baochun2 tgaatttcaatgaatctttttcttttggtgaaattcaatacatgaataaaacaaacaatt

baochun1 tgaatttcaatgaatctttttcttttggtgaaattcaatacatgaataaaacaaacaatt

************************************************************

baochun2 tttttggaaatacttattttttttctgctaaaaattattacaggtcccagttgattcaac

baochun1 tttttggaaatacttattttttttctgctaaaaattattacaggtcccagttgattcaac

************************************************************

baochun2 aattggatcgctggggttatcgtgtcattagtataggttttatctttttaaccataggga

baochun1 aattggatcgctggggttatcgtgtcattagtataggttttatctttttaaccataggga

************************************************************

baochun2 ttctttcgggagccgtatgggctaatgaagcatggggttcatattggaattgggacccaa

baochun1 ttctttcgggagccgtatgggctaatgaagcatggggttcatattggaattgggacccaa

************************************************************

baochun2 aagaaatttgggcatttattacttggaccgtattcgcgatttatttacatagtcgaacaa

baochun1 aagaaatttgggcatttattacttggaccgtattcgcgatttatttacatagtcgaacaa

************************************************************

baochun2 ataaaaaatggccccctgcaaattctgcgattgtagcttctgttggcttttttataattt

baochun1 ataaaaaatggccccctgcaaattctgcgattgtagcttctgttggcttttttataattt

************************************************************

baochun2 ggatatgctactttggggtcaatctgttaggaatcggactgcatagttatggttcattta

baochun1 ggatatgctactttggggtcaatctgttaggaatcggactgcatagttatggttcattta

************************************************************

baochun2 tattaatgtctaaagaaagtctctaacaaatataaaaaaaatacaacctatataaaaaac

baochun1 tattaatgtctaaagaaagtctctaacaaatataaaaaaaatacaacctatataaaaaac

************************************************************

baochun2 tttgtgtaagccggcgaaaaccatatgaatcaaatagtgtagcgattcatatggttctca

baochun1 tttgtgtaagccgacgaaaaccatatgaatcaagtagtgtagcgattcatatggttctca

*************.*******************.**************************

baochun2 aaaaaccaaacgtatctggttcaaaactacttatttttttacttaaagaaaaaaataagt

baochun1 aaaaaccaaacgtatctggttcaaattgac---tttttttacttaaagaaaaaaataagt

************************* . ** ***************************

baochun2 agtttttcattctacagcgaacaattttgaaaatcatagttttttttatagatagtttgc

baochun1 agtttttcattctacagcgaacaattttgaaaatcatagttttttttatagatagtttgc

************************************************************

baochun2 atgcaaactatctataaaaaaaattagataaaataacttctaccttgtcaactgataatg

baochun1 atgcaaactatctataaaaaaaattagataaaataacttctaccttgtcaactgataatg

************************************************************

baochun2 aaagaacgaaatccgggtaaaaaccaatacctactattggtaaaaaaatagagatcgaaa

baochun1 aaagaacgaaatccgggtaaaaaccaatacctactattggtaaaaaaatagagatcgaaa

************************************************************

baochun2 caaataactctcgcggtccagaatcaaaaaaataagaggttggaacattaaaaaacttgt

baochun1 caaataactctcgcggtccagaatcaaaaaaataagaggttggaacattaaaaaacttgt

************************************************************

baochun2 atccatagaacatctgacgtgacatagacaataaataaataggagttaatatcattccaa

baochun1 atccatagaacatctgacgtgacatagacaataaataaataggagttaatatcattccaa

************************************************************

baochun2 ttgccattacaagaatgattagcatttttggcattaaaaaaaatttttggctggtaatta

baochun1 ttgccattacaagaatgattagcatttttggcattaaaaaaattttttggctggtaatta

****************************************** *****************

baochun2 ctccaaaaaagactagcaattctgcaacaaacccactcatacctggtaatgcaagagagg

baochun1 ctccaaaaaagactagcaattctgcaacaaacccactcatacctggtaatgcaagagagg

************************************************************

baochun2 ccattgaaaagctactgaacattgtgaatatttttggcattgggatagctattccgccca

baochun1 ccattgaaaagctactgaacattgtgaatatttttggcattgggatagctattccgccca

************************************************************

baochun2 tttcgtcgagataagcaagccgtattctatcataactcgttcctgccaagaaaaagagtg

baochun1 tttcgtcgagataagcaagccgtattctatcataactcgttcctgccaagaaaaagagtg

************************************************************

baochun2 cggcaccaataaatccatgagatattatttgtaaaatggctccattgagtcccatatctg

baochun1 cggcaccaataaatccatgagatattatttgtaaaatggctccattgagtcccgtatctg

*****************************************************.******

baochun2 ttatcgaacctattcctataattataaaacccatatgagatacagaagaatatgctattc

baochun1 ttatcgaacctattcctataattataaaacccatatgagatacagaagaatatgctattc

************************************************************

baochun2 ttttctttaaattacgttgaccagaagatgttaaagctgcatagattatttgcattgtgc

baochun1 ttttctttaaattacgttgaccagaagatgttaaagctgcatagattatttgcattgtgc

************************************************************

baochun2 ctattatcattaaccaaggagaaaatagggaatgcgcgtgaggcaataattccatattga

baochun1 ctattatcattaaccaaggagaaaatagggaatgcgcgtgaggcaataattccatattga

************************************************************

baochun2 tacgaatcaacccatatgctcccatttttaataaaattccagcgagaagcatacaagtac

baochun1 tacgaattaacccatatgctcccatttttaataaaattccagcgagaagcatacaagtac

*******.****************************************************

baochun2 tgtaatgtgcttctccatgagtatctggtaaccatgtatgtagaggtataatcggcaatt

baochun1 tgtaatgtgcttctccatgagtatctggtaaccatgtatgtagagggataatcggcaatt

********************************************** *************

baochun2 tgacagcaaaagcaagaaaaaagccaatatagaatattatttctaatgccacaggatagg

baochun1 tgacagcaaaagcaagaaaaaagccaatatagaatattatttctaatgccacaggatagg

************************************************************

baochun2 gttgattggctaatgtttcaaaatttaatattggttcattagaactatataaaccgatac

baochun1 gttgattggctaatgtttcaaaatttaatattggttcattagaactatataaaccgatac

************************************************************

baochun2 ccaaaactcccattaacaaaaaaatagacccgcccgcagtgtacaaaataaattttgtag

baochun1 ccaaaactcccattaacaaaaaaatagacccgcccgcagtgtacaaaataaattttgtag

************************************************************

baochun2 ctgaatacagacgtttctttccgccccacatggatagaagtaaataaacaggaattaatt

baochun1 ctgaatacagacgtttctttccgccccacatggatagaagtaaataaacaggaattaatt

************************************************************

baochun2 ctaactcccacatgataaaaaacagtaagagatcttgagaagaaaataatcctatttgag

baochun1 ctaactcccacatgatgaaaaacagtaagagatcttgagaagaaaataatcctatttgag

****************.*******************************************

baochun2 cactgtacattgctaacatcaagaaatggaataatcgagaatctcgagtaatcggaaagg

baochun1 cactgtacattgctaacatcaagaaatggaataatcgagaatctcgagtaatcggaaagg

************************************************************

baochun2 ctgctaacgtagctaaagtagtgatgaatcccgtgagtaaaatgggtcctatagaaagcc

baochun1 ctgctaacgtagctaaagtagtgatgaatcccgtgagtaaaatgggtccgatagaaagcc

************************************************* **********

baochun2 catctattcccaatttccagtggaaatgaaaaaaatcgatccatttataatcttcaacta

baochun1 catctattcccaatttccagtggaaatgaaaaaaatcgatccatttataatcttcaacta

************************************************************

baochun2 gttgaattaatggatcgtcccattggaaatgataacagaatgcataggtcgttaacagaa

baochun1 gttgaattaatggatcgtcccattggaaatgataacagaatgcataggtcgttaacagaa

************************************************************

baochun2 gttccaatatacatataccaatagtataccacctaattattctatttcctctatggggaa

baochun1 gttccaatatacatataccaatagtataccacctaattattctatttcctctatggggaa

************************************************************

baochun2 gaaagaaaatgaaagaacccaaaaatattggaaaaactacaattattgttaaccaaggaa

baochun1 gaaagaaaatgaaagaacccaaaaatattggaaaaactacaattattgttaaccaaggaa

************************************************************

baochun2 aatgattcgtggtaaagacaagatacacttagaccaaaaaaacccgtactcgaacaaaat

baochun1 aatgattcgtggtaaagacaagatacacttagaccaaaaaaacccgtactcgaacaaaat

************************************************************

baochun2 tgaaatattttattttgagcacgggtttttgtcgataaaaaaatcaaatgaattcaagtg

baochun1 tgaaatattttattttgagcacgggtttttgtcgataaaaaaatcaaatgaattcaagtg

************************************************************

baochun2 gtttttttttctggaacgtatcaataagccagacccatgctacgggttgtttcatgccat

baochun1 g--tttttttctggaacgtatcaataagctagacccatgctacgggttgtttcatgccat

* **************************.******************************

baochun2 aaataaactcgaacactcaagaaatccgttgggcaggcagattcacatctcttacaaccc

baochun1 aaataaactcgaacactcaagaaatccgttgggcaggcagattcacatctcttacaaccc

************************************************************

baochun2 acacaatcctctgttcttggagcagaagcaatttgtttagctttacagccgtcccaaggt

baochun1 acacaatcctctgttcttggagcagaagcaatttgtttagctttacagccgtcccaaggt

************************************************************

baochun2 atcatttctaatacatctgtggggcaagctcggacgcattgagtacacccaatacatgta

baochun1 atcatttctaatacatctgtggggcaagctcggacgcattgagtacacccaatacatgta

************************************************************

baochun2 tcataaatttttactgaatgtgacattggatctatacatttttggatgtcataaaatttc

baochun1 tcataaatttttactgaatgtgacattggatctatacatttttggatgtcataaaatttc

************************************************************

baochun2 tatctagtaaatcgagtaaacttaataaataaattatatttagttaccagacgaatcaag

baochun1 tatctagtaaatcgagtaaacttaataaataaattatatttagttaccagacgaatcaag

************************************************************

baochun2 aagttactacaaatttttgataatcaatctatttttagattgatttatgttttgagagag

baochun1 aagttactacaaatttttgataatcaatctatttttagattgatttatgttttgagagag

************************************************************

baochun2 agccaagatactttgagttctttatgtttttgtaaaaatgatttgatttctatatatttt

baochun1 agccaagatactttgagttctttatgtttttgtaaaaatgatttgatttctatatatttt

************************************************************

baochun2 tacgcgacaaatatattaagctacattaattaaatattaaatattatagtcaatttctct

baochun1 tacgcgacaaatatactaagctaaatta-------attaaatattatagtcaatttctct

***************.******* **** *************************

baochun2 aattaatattatttattcaacaaattcgattggttaatacgaattgattttctattacga

baochun1 aattaatattatttattcaacaaattcgattggttaatacgaattgattttctattacga

************************************************************

baochun2 taaattgacgaaacaatagccaatccaattgctgcttcagcagctgcgatagctataaca

baochun1 taaattgacgaaacaatagccaatccaattgctgcttcagcagctgcgatagctataaca

************************************************************

baochun2 aaaatcgaaaaaatagttccttttaattgacgattatcaaaaaaatcagaaaatgttaca

baochun1 aaaatcgaaaaaatagttccttttaattgacgattatcaaaaaaatcagaaaatgttaca

************************************************************

baochun2 aaattcatattaactgcattcaatataagttcaagacacataagtgctctaaccatattt

baochun1 aaattcatattaactgcattcaatataagttcaagacacataagtgctctaaccatattt

************************************************************

baochun2 cgactcgtgatcaatccataaataccgatagaaaataaacaggcactcaaaacaagtaca

baochun1 cgactcgtgatcaatccataaataccgatagaaaataaacaggcactcaaaacaagtaca

************************************************************

baochun2 tgttcgagcatgattaactaactccttatgaatctcaatttagttcaatatgaacaacaa

baochun1 tgttcgagcatgattaactaactccttatgaatctcaatttagttcaatatgaacaacaa

************************************************************

baochun2 ttcaaccaattaaattaactaaaataaaccaaatagggaatatattgtgaatagattgaa

baochun1 ttcaaccaattaaattaactaaaataaaccaaatagggaatatattgtgaatagattgaa

************************************************************

baochun2 gtaaagcatttctatttgaattttcggcacgaattcaaatagaatggaagagaaaaaaat

baochun1 gtaaagcatttctatttgaattttcggcacgaattcaaatagaatggaagagaaaaaaat

************************************************************

baochun2 atgaaaatctagaaaaattttgagttggattccaattttgaaagatttcctattgacgag

baochun1 atgaaaatctagaaaaattttgagttggattccaattttgaaagatttcctattgacgag

************************************************************

baochun2 ctacagcaattgcacctattaaagcaactaaaagaattattgaaatgagttcaaatggaa

baochun1 ctacagcaattgcacctattaaagcaactaaaagaattattgaaatgagttcaaatggaa

************************************************************

baochun2 gaaaaaaatctgttgataaatgaataccaatttgttgactattacttatcaaatcttgtt

baochun1 gaaaaaaatctgttgataaatgaataccaatttgttgactattacttatcaaatcttgtt

************************************************************

baochun2 ctagaatctgattggatcttgtagcccaaataatcccgtaccatgacgtatctagaatag

baochun1 ctagaatctgattggatcttgtagcccaaataatcccgtaccatgacgtatctagaatag

************************************************************

baochun2 tggtaattaaagaaattaaaatacttgtacaaatcagggaagtaattccatctccaacag

baochun1 tggtaattaaagaaattaaaatacttgtacaaatcagggaagtaattccatctccaacag

************************************************************

baochun2 tcgaaagatgaaaatctttgtaatattctgaaccattcatgaacatcacagcaaatagga

baochun1 tcgaaagatgaaaatctttgtaatattctgaaccattcatgaacatcacagcaaatagga

************************************************************

baochun2 ttaaaacgtttattgctcctacataaatcaggagctgcgccacagctacaaagtgggagt

baochun1 ttaaaacgtttattgctcctacataaatcaggagctgcgccgcagctacaaagtgggagt

*****************************************.******************

baochun2 ttgctagaatatagaataaggatatacaaaaaaaaaccaatcccaaagaaaaggcagaat

baochun1 ttgctaaaatatagaataaggatatacaaaaaaaaaccaatcccaaagaaaaggcagaat

******.*****************************************************

baochun2 aaattggattgggaagtaatatcactcctaggcttcctaatataagacccaatcccagga

baochun1 aaattggattgggaagtaatatcactcctaggcttcctaatataagacccaatcccagga

************************************************************

baochun2 agactaaaagaaagtcatgtattggtccaggtaaatccattctatagtaaaataataaga

baochun1 agactaaaagaaagtcatgtattggtccaggtaaatccattctat-gtaaaataataaga

********************************************* **************

baochun2 aaatagaaatatcatgactttgttgacctgaccaggcaaaaggcagttactttatttgtt

baochun1 aaatagaaatatcatgactttgttgacctgaccaggcaaaaggcagttactttatttgtt

************************************************************

baochun2 ttatcctatgttaattgaattcaatcctaataaatatggagtgatggagatataattatt

baochun1 ttatcctatgttaattgaattcaatcctaataaatatggagtgatggagatacaattatt
[truncated: 117,689 more chars]
